# Arylative Intramolecular Allylation of Ketones with 1,3‐Enynes Enabled by Catalytic Alkenyl‐to‐Allyl 1,4‐Rhodium(I) Migration

**DOI:** 10.1002/anie.201703155

**Published:** 2017-05-19

**Authors:** Benjamin M. Partridge, Michael Callingham, William Lewis, Hon Wai Lam

**Affiliations:** ^1^ School of ChemistryUniversity of NottinghamUniversity ParkNottinghamNG7 2RDUK; ^2^ The GSK Carbon Neutral Laboratories for Sustainable ChemistryUniversity of NottinghamJubilee Campus, Triumph RoadNG7 2TUUK; ^3^ Department of ChemistryUniversity of SheffieldSheffieldS3 7HFUK

**Keywords:** allylic compounds, cyclization, isomerization, reaction mechanisms, rhodium

## Abstract

Alkenyl‐to‐allyl 1,4‐rhodium(I) migration enables the generation of nucleophilic allylrhodium(I) species by remote C−H activation. This new mode of reactivity was employed in the diastereoselective reaction of arylboron reagents with substrates containing a 1,3‐enyne tethered to a ketone, to give products containing three contiguous stereocenters. The products can be obtained in high enantioselectivities using a chiral sulfur‐alkene ligand.

Catalytic C−H functionalizations have revolutionized chemical synthesis by providing powerful new tools for bond construction.^1^ However, a critical objective for the advancement of this field is its application to a more diverse range of transformations. Nucleophilic allylations^2^ are important reactions that could benefit from C−H functionalization principles. Most typically, these processes have employed allylmetal(loid) reagents such as allyltin, allylboron, or allylsilicon compounds.^2^ The generation of nucleophilic allylmetal species by the activation of allylic C−H bonds would bypass the need to prepare such reagents and potentially increase efficiency by streamlining synthetic sequences. This strategy would also be a valuable complement to nucleophilic allylations involving migratory insertions of allenes,^3^, ^4^ the use of simple π‐unsaturated compounds in hydrogenative or redox‐triggered additions,^5^, ^6^ hetero‐ene reactions,^7^ and Prins reactions.^8^

Although generating electrophilic allylmetal species by allylic C−H activation is well‐known,^9^, ^10^ there is, to our knowledge, limited precedent for corresponding processes that provide nucleophilic allylmetals.^11^ Very recently, the groups of Schneider,11a Kanai,11b and Mita and Sato11c described the formation and trapping of nucleophilic allylmetal species from simple hydrocarbons. In view of the nucleophilic character of allylrhodium(I) species,4a, 12 we envisaged that activation of a remote C−H bond by 1,4‐rhodium(I) migration12d, 13, 14 could also achieve this goal. Specifically, rhodium(I)‐catalyzed reaction of an arylboron reagent with the alkyne of a 1,3‐enyne would provide the alkenylrhodium species **A** (Scheme 1). This intermediate could then undergo a 1,4‐rhodium(I) shift to the *cis*‐allylic substituent to give the allylrhodium(I) species **B**, which could be trapped by an electrophile. This approach was expected to be challenging, given that there is only very limited precedent for rhodium(I) to migrate to C(sp^3^) centers.12d, 14k,14m Nevertheless, the generation of electrophilic allylrhodium(III) species by a similar strategy in our rhodium(III)‐catalyzed oxidative annulations of 1,3‐enynes provided some encouragement.^10^ Herein, we describe the implementation of this strategy in arylative intramolecular allylations of ketones to give stereochemically complex fused bicycles with high diastereoselectivities. Preliminary results of enantioselective reactions are also provided.

**Scheme 1:**
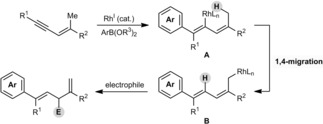
Proposed alkenyl‐to‐allyl 1,4‐rhodium(I) migration.

This study began with the reaction of the enynone **1 a** with 3,5‐dimethylphenyl pinacol boronate (1.3 equiv), [{Rh(cod)Cl}_2_] (1.5 mol %), and K_3_PO_4_ (0.3 equiv) at 65 °C for 16 hours in various solvents (Table 1). A 3,5‐disubstituted arylboron reagent was used to minimize 1,4‐rhodium(I) migration onto the aryl group as described previously,^15^ as it is well‐known that migration onto an aryl ring *ortho* to a substituent is unfavorable.14a,14i, 15a Pinacol boronates were used because 3,5‐disubstituted variants are easily accessed through iridium‐catalyzed C−H borylation.^16^ The reaction conducted in THF/MeOH (10:1) gave diastereomeric bicycles **2 aa** 17 and **2 ab** 18 in a 13:87 ratio (entry 1). After purification, **2 aa** and **2 ab** were isolated in 11 and 46 % yield, respectively. Traces of the diketone **3 a** were also formed, and resulted from arylrhodation of the alkyne of **1 a** with the regioselectivity opposite to that seen in the formation of **2 aa/2 ab**, followed by a cyclization‐fragmentation pathway.15a, 19 Notably, switching the solvent to MeCN/MeOH (10:1) reversed the sense of diastereoselectivity and gave **2 aa** and **2 ab** in 66 and 5 % yield, respectively (entry 2). Using TBME/*t*BuCN/MeOH (10:1.2:1) gave a further increase in diastereoselectivity (entry 3).

**Table 1:** Evaluation of solvents.^[a]^ 
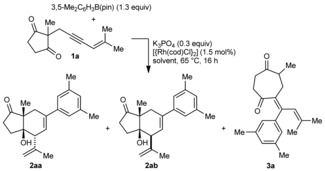

Entry	Solvent	d.r.	Yield [%]^[c]^		
		**2 aa**/**2 ab** ^[b]^	**2 aa**	**2 ab**	**3 a**
1	THF/MeOH (10:1)	17:83	11	46	n.d.^[d]^
2	MeCN/MeOH (10:1)	91:9	66	5	9
3	TBME/*t*BuCN/MeOH (10:1.2:1)	94:6	73	4	14

[a] Reactions employed 0.50 mmol of **1 a**. [b] Determined by ^1^H NMR analysis of the crude reaction mixtures. [c] Yield of the isolated product. [d] n.d.=not determined. cod=1,5‐cyclooctadiene, TBME=*tert*‐butyl methyl ether, THF=tetrahydrofuran.

In the proposed catalytic cycle (Scheme 2), transmetalation of the arylboronate with the rhodium methoxide **4** provides the arylrhodium species **5**, which undergoes migratory insertion with the alkyne of **1 a** to give alkenylrhodium species **6**. 1,4‐Rhodium migration gives the allylrhodium species (*Z*)‐**7**, which cyclizes onto a ketone to provide the rhodium alkoxide **8**. Methanolysis of **8** liberates the product **2 aa** or **2 ab** and regenerates **4**.

**Scheme 2:**
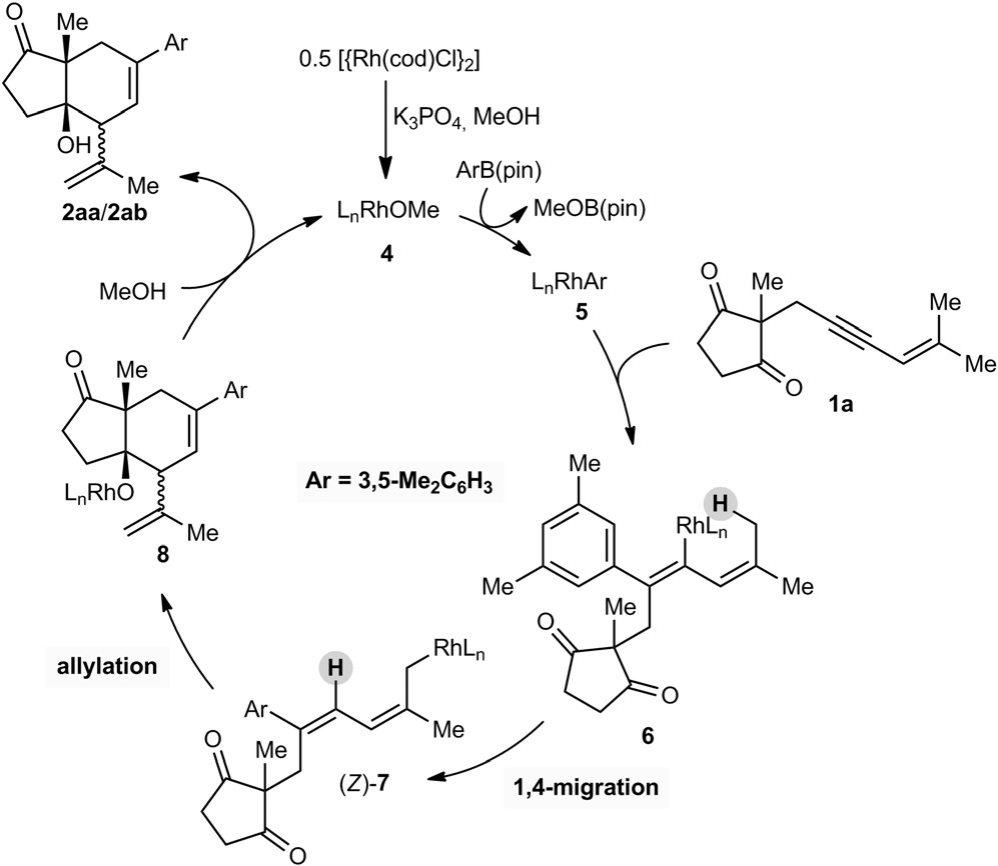
Proposed catalytic cycle.

Scheme 3 presents the reactions of **1 a** with various arylboronic acid pinacol esters. Products analogous to **3 a** were generally formed in up to 20 % yield (by ^1^H NMR analysis of the crude reaction mixtures) but were not isolated. The reaction is tolerant of halide (**2 ba**, **2 ea**, and **2 ha**), methoxy (**2 ca** and **2 fa**), trifluoromethyl (**2 da**), and carbomethoxy groups (**2 ea**) on the arylboronate. In addition, 3,5‐disubstituted (**2 aa**–**2 ea**), 3,4,5‐trisubstituted (**2 fa**), and 2,5‐disubstituted arylboronates (**2 ga** and **2 ha**) are tolerated. 2,5‐Disubstituted arylboronates gave lower yields (**2 ga** and **2 ha**), which is presumably a consequence of the steric hindrance of the *ortho*‐substituent. Finally, a heteroarylboronate is also tolerated (**2 ia**).

**Scheme 3:**
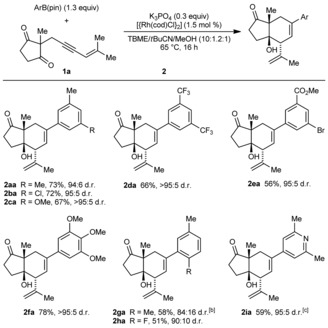
[a] Reaction of **1 a** with various arylboronates. Reactions employed 0.50 mmol of **1 a**. Diastereomeric ratios were determined by ^1^H NMR analysis of the crude reaction mixtures. Yields are of isolated, diastereomerically pure products. [b] Reaction performed with 2.5 mol % [{Rh(cod)Cl}_2_]. [c] Reaction employed 0.45 mmol of **1 a**.

Next, variation of the enynone was explored, and the substrates **1 b**–**f,** containing methyl groups *cis* to the alkyne, all reacted successfully with 3,5‐dimethylphenyl pinacol boronate (Table 2). Substrates containing hydrogen, phenyl, or alkyl groups *trans* to the alkyne are tolerated (entries 1–3). With the phenyl‐containing substrate **1 c**, however, application of the standard reaction conditions gave no diastereoselectivity (1:1 d.r.).^20^ Fortunately, switching the solvent to 2‐MeTHF/MeOH (10:1) gave the *syn*,*syn*‐diastereomer **9 cb** in greater than 95:5 d.r. and 62 % yield (entry 2).^17^ In contrast to our findings using rhodium(III) catalysis,^10^ substrates containing methylene groups (as opposed to methyl groups) *cis* to the alkyne are unreactive. Variation of the 1,3‐diketone is also possible. For example, the indane‐1,3‐dione **1 e** gave **9 ea** in 74 % yield and >95:5 d.r. (entry 4).^17^ Under the standard reaction conditions, the six‐membered cyclic 1,3‐diketone **1 f** underwent decomposition in competition with arylative allylation. However, by changing the arylboronate to the more reactive neopentyl glycol ester, and using K_2_CO_3_ and *t*AmOH in place of K_3_PO_4_ and MeOH, respectively, **9 fa** was formed in 67 % yield (entry 5).^17^ The process is not limited to cyclic 1,3‐diketones as the β‐ketoester **10** reacted smoothly using 2.5 mol % of [{Rh(cod)Cl}_2_] to give **11** in 62 % yield and 95:5 d.r. [Eq. 1].
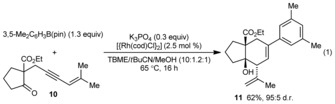



**Table 2:** Arylative allylation of various enynones.^[a]^ 
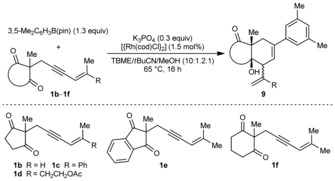

Entry	Enynone	Product (Ar=3,5‐Me_2_C_6_H_3_)		d.r.^[b]^	Yield [%]^[c]^
1^[d]^	**1 b**	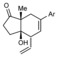	**9 ba**	n.d.^[e]^	50 (+7)^[f]^
2^[g]^	**1 c**	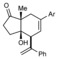	**9 cb**	>95:5	62
3	**1 d**	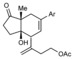	**9 da**	84:16	52
4	**1 e**	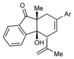	**9 ea**	>95:5	74
5^[h]^	**1 f**	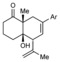	**9 fa**	84:16	67

[a] Reactions employed 0.50 mmol of **1 b**–**f**. [b] Determined by ^1^H NMR analysis of the crude reaction mixtures. [c] Yield of isolated, diastereomerically pure products. [d] Using 2.5 mol % of [{Rh(cod)Cl}_2_]. [e] The d.r. value could not be determined by ^1^H NMR analysis. [f] Yield of the isolated minor *syn*‐*syn* diastereomer **9 bb**. [g] Using 2‐MeTHF/MeOH (10:1) in place of TBME/*t*BuCN/MeOH (10:1.2:1). [h] Using 3,5‐Me_2_C_6_H_3_B(neo) (1.3 equiv), K_2_CO_3_ (1.3 equiv), and *t*AmOH (1.5 equiv) as the reagents in TBME/*t*BuCN (8.3:1). neo=neopentyl glycol.

Furthermore, the fully acyclic substrates **12 a** and **12 b** also underwent successful arylative intramolecular allylation [Eq. 2 and 3], although the diastereoselectivities were lower compared with substrates containing cyclic ketones. For acceptable yields, it was important to use neopentyl glycol boronate, K_2_CO_3_, and *t*AmOH. Under these reaction conditions, **12 a** reacted with 3,5‐dimethylphenyl neopentyl glycol boronate to give the diastereomeric products **13 aa** and **13 ab** in 28 and 27 % yield, respectively [Eq. (2)].^21^ Improved results were obtained with **12 b**, which contains a geminal dimethyl group in the tether, and **13 ba** and **13 bb** were obtained in 51 and 12 % yield, respectively [Eq. (3)].^21^ The same reactions conducted in 2‐MeTHF instead of TBME/*t*BuCN gave **13 ab** and **13 bb** as the major products, but were lower yielding.
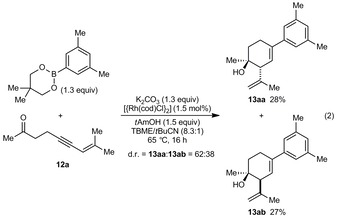


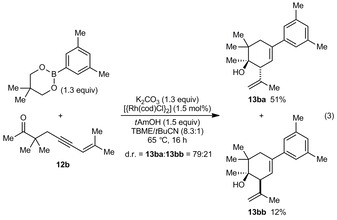



The substrate **14**, which contains an *E*‐1,3‐enyne, did not undergo the reaction, and only starting materials were recovered [Eq. 4]. This result confirms the requirement for *cis*‐allylic hydrogen atoms to be present in the enyne to allow 1,4‐rhodium(I) migration to occur (compare with Table 2, entry 1 using the *Z*‐isomer **1 b**). In addition, reaction of hexadeuterated enynone [D]_6_‐**1 a** with 3,5‐dimethylphenylboronic acid pinacol ester gave [D]_6_‐**2 aa** with greater than 95 % deuterium transfer to the alkene of the cyclohexene [Eq. 5]. This outcome is consistent with 1,4‐rhodium(I) migration occurring by a C−H oxidative addition/reductive elimination through a rhodium(III) hydride intermediate as hypothesized previously for alkenyl‐to‐aryl 1,4‐rhodium(I) migration.14j

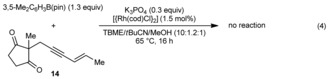


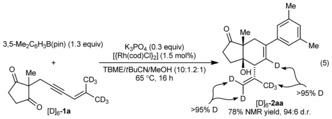



Up until this point, all of the arylboronates evaluated possess substitution patterns that disfavor 1,4‐rhodium(I) migration of intermediates such as **6** onto the aryl group. To assess whether alkenyl‐to‐allyl 1,4‐rhodium(I) migration would still be favored when a sterically more accessible site is available, **1 a** was reacted with phenylboronic acid (Scheme 4). The reaction in TBME/*t*BuCN (8:1) in the presence of *t*‐amyl alcohol (1.5 equiv) gave a 95:5 mixture of inseparable products, **15** and **2 ja**. The product **15** results from 1,4‐rhodium(I) migration onto the phenyl group followed by intramolecular ketone arylation,^15^ whereas **2 ja** is the arylative allylation product. When the solvent was changed to 2‐MeTHF, the allylation product **2 jb** was formed preferentially (36:64 ratio of **15**/**2 jb**) in 89:11 d.r., and was isolated as a single diastereomer in 45 % yield. The reasons for this switch in chemoselectivity are not currently known.

**Scheme 4:**
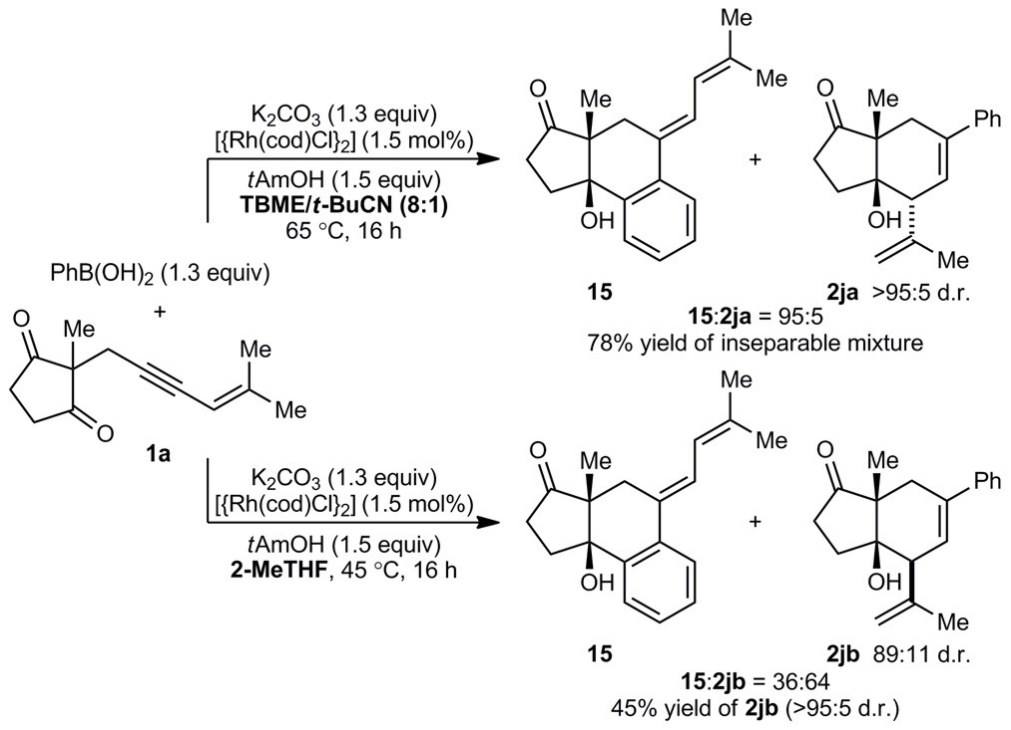
Reaction of **1 a** with PhB(OH)_2_.

Consistent with models proposed in prior rhodium‐catalyzed nucleophilic allylations,4a, 12b–12e we suggest that allylation occurs through cyclic six‐membered transition states (Scheme 5). In the absence of a nitrile in the reaction medium (Table 1, entry 1), we assume that (*Z*)‐**7**, formed from 1,4‐rhodium(I) migration of **6**, cyclizes through a chairlike arrangement (**TS1**) to give **2 aa** (Scheme 5). The boatlike structure **TS2** should be disfavored. However, when a coordinating nitrile is present (Table 1, entries 2 and 3), the rate of cyclization could be decreased, allowing isomerization of (*Z*)‐**7** into (*E*)‐**7**.^22^ Thereafter, we assume that cyclization of (*E*)‐**7** occurs through the chairlike conformation **TS5** to give **2 ab** (Scheme 5). The alternative conformation **TS3** is likely to be disfavored because of 1,3‐diaxial interactions and allylic 1,3‐strain. The boatlike structure **TS4** is also likely to be unfavorable. However, we do not exclude the possibility that when a nitrile is present, **2 aa** is formed by cyclization of (*E*)‐**7** through an open transition state because of preferential coordination of rhodium to the nitrile rather than the ketone.

**Scheme 5:**
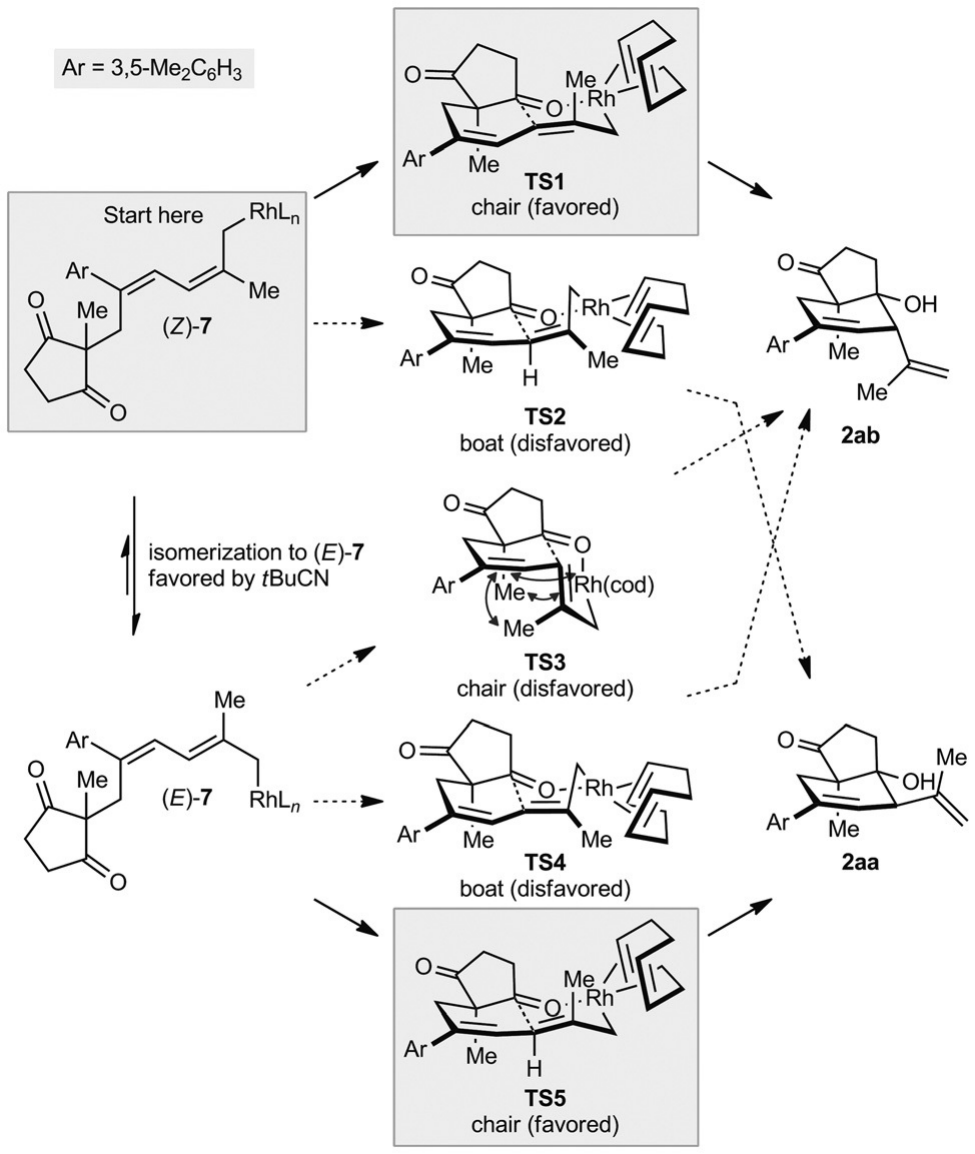
Possible stereochemical models.

Similar chairlike transition states can be used to explain the outcomes of the reactions **12 a** and **12 b** [Eqs. (2) and (3)], and the diastereomeric ratios observed may be a consequence of their more flexible nature (see the Supporting Information).

Finally, preliminary efforts at developing enantioselective reactions were conducted (Table 3).^23^ Only modest results were obtained with chiral diene ligands^24^ (see Supporting Information), while no reaction occurred when chiral bisphosphines were used. However, the reaction of **1 a** with 3,5‐dimethylphenylboronic acid (1.3 equiv) in the presence of [{Rh(C_2_H_4_)_2_Cl}_2_] (2.5 mol %), the sulfur‐alkene **L1** 25 (5.0 mol %), and KF (1.5 equiv) in TBME/*t*BuCN/MeOH (40:5:1) gave (+)‐**2 aa** 17 in 61 % yield and 91 % *ee* (entry 1). The diastereomeric product (+)‐**2 ab** was obtained in 11 % yield and 88 % *ee*. Similar results were obtained with 3‐chloro‐5‐methylphenylboronic acid (entry 2).

**Table 3:** Enantioselective reactions.^[a]^ 
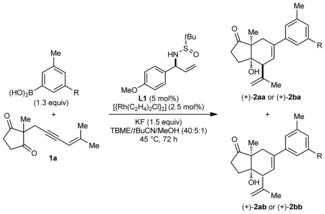

Entry	R	d.r.^[b]^	Major isomer^[c]^	Minor isomer^[c]^
1	Me	80:20	(+)‐**2 aa** 61 %, 91 % *ee*	(+)‐**2 ab** 11 %, 88 % *ee*
2	Cl	78:22	(+)‐**2 ba** 47 %, 90 % *ee*	(+)‐**2 bb** 10 %, 90 % *ee*

[a] Reactions employed 0.25 mmol of **1 a**. [b] Determined by ^1^H NMR analysis of the crude reaction mixture. [c] Yields are of isolated, diastereomerically pure products. Enantiomeric excesses were determined HPLC analysis on a chiral stationary phase.

In summary, we have reported the rhodium‐catalyzed arylative allylation of enynones with arylboron reagents. The key step of the reaction is the alkenyl‐to‐allyl 1,4‐rhodium(I) migration, a new mode of reactivity which enables the generation of nucleophilic allylrhodium(I) species without prefunctionalization of the allylic position. Cyclization of the allylrhodium species onto a pendant ketone leads to bicyclic products containing three contiguous stereocenters with high diastereoselectivities. The products can be obtained in high enantioselectivities using a chiral sulfur‐alkene ligand. Further applications of this promising platform for generating allylmetal species are in progress.

## Conflict of interest

The authors declare no conflict of interest.

## Supporting information

As a service to our authors and readers, this journal provides supporting information supplied by the authors. Such materials are peer reviewed and may be re‐organized for online delivery, but are not copy‐edited or typeset. Technical support issues arising from supporting information (other than missing files) should be addressed to the authors.

Supplementary
