# Supplementary material for: Arylative Intramolecular Allylation of Ketones with 1,3‐Enynes Enabled by Catalytic Alkenyl‐to‐Allyl 1,4‐Rhodium(I) Migration
Source: Angew Chem Int Ed Engl. 2017 May 19;56(25):7227–32. doi: 10.1002/anie.201703155 (PMC5488243; doi:10.1002/anie.201703155)
Supplement: Supplementary file 1 — Supplementary [file ANIE-56-7227-s001.pdf]

Angewandte Chemie
Eine Zeitschrift der Gesellschaft Deutscher Chemiker

Supporting Information

Arylative Intramolecular Allylation of Ketones with 1,3-Enynes Enabled by Catalytic Alkenyl-to-Allyl 1,4-Rhodium(I) Migration

*Benjamin M. Partridge, Michael Callingham, William Lewis, and Hon Wai Lam**

anie_201703155_sm_miscellaneous_information.pdf

Author Contributions

B.P. Conceptualization: Equal; Data curation: Lead; Formal analysis: Lead; Investigation: Lead; Methodology: Lead; Validation: Lead; Writing – original draft: Equal; Writing – review & editing: Equal
M.C. Data curation: Supporting; Formal analysis: Supporting; Investigation: Supporting; Methodology: Supporting; Validation: Supporting; Writing – review & editing: Supporting
W.L. Data curation: Supporting; Formal analysis: Supporting; Validation: Supporting
H.L. Conceptualization: Equal; Formal analysis: Supporting; Funding acquisition: Lead; Supervision: Lead; Validation: Supporting; Writing – original draft: Equal; Writing – review & editing: Lead.

Supporting Information

1. General Information.. 2
2. Synthesis of Substrates.. 3
3. Rhodium-Catalyzed Arylative Allylations.. 11
4. Investigation of Deuterium Transfer... 32
5. Enantioselective Reactions 34
6. NMR Spectra.. 39

1. General Information

All air-sensitive reactions were carried out under a nitrogen atmosphere using oven-dried apparatus. TBME, MeTHF, *t*-BuCN, and MeOH were dried over activated 4 Å molecular sieves and sparged with N_2 before use. All arylboronic esters were prepared using standard protocols either by either esterification of the corresponding arylboronic acid (**S1**, **S4**, **S6**, and **S10**),[1] or iridium-catalyzed C–H borylation of the appropriate arene (**S2**, **S3**, **S5**, **S7**, **S8**, and **S9**).[2]

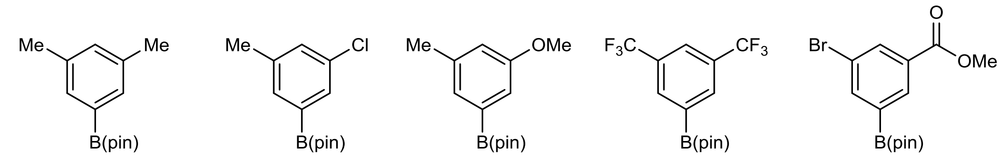

S1 **S2** **S3** **S4** **S5**

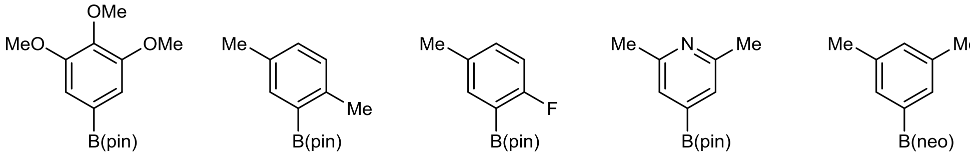

S6 **S7** **S8** **S9** **S10**

All commercially available reagents were used as received unless otherwise stated. All petroleum ether used was 40–60 °C petroleum ether. Thin layer chromatography (TLC) was performed on Merck DF-Alufoilien $60F_{254}$ 0.2 mm precoated plates. Compounds were visualized by exposure to UV light or by dipping the plates into solutions of potassium permanganate or vanillin followed by heating. Flash column chromatography was carried out using silica gel (Fisher Scientific 60Å particle size 35–70 micron). Melting points were recorded on a Gallenkamp melting point apparatus and are uncorrected. The solvent of recrystallization is reported in parentheses. Infra-red spectra were recorded on a Nicolet Avatar 360 FT instrument on the neat compound. NMR spectra were acquired on a Bruker AV500, AV(III)500HD, AV400, AV(III)400, AV(III)400HD, or DPX400 spectrometer. ^{1}H and ^{13}C NMR spectra were referenced to external tetramethylsilane *via* the residual protonated solvent (^{1}H) or the solvent itself (^{13}C). All chemical shifts are reported in parts per million (ppm). NMR data has been reported with the following abbreviations: s = singlet, d = doublet, t = triplet, q = quartet, quin = quintet, sept = septet, br = broad. For $CDCl_3$, the shifts are referenced to 7.27 ppm for ^{1}H NMR spectroscopy and 77.0 ppm for ^{13}C NMR spectroscopy. ^{2}H NMR spectra were referenced to the residual deuterated solvent (7.27 ppm). High-resolution mass spectra were recorded using electrospray ionization (ESI) or electron ionization (EI) techniques. X-ray diffraction data were collected at 120 K on an Agilent GV1000 using CuKα radiation, and refined in SHELXTL.

1. For a representative procedure, see: S. Morandi, E. Caselli, A. Forni, M. Bucciarelli, G. Torre, F. Prati, *Tetrahedron: Asymmetry* **2005**, *16*, 2918-2926.
2. (a) T. Ishiyama, J. Takagi, K. Ishida, N. Miyaura, N. R. Anastasi, J. F. Hartwig, *J. Am. Chem. Soc.* **2001**, *124*, 390-391. (b) J. M. Murphy, X. Liao, J. F. Hartwig, *J. Am. Chem. Soc.* **2007**, *129*, 15434-15435.

2. Synthesis of Substrates

General Procedure A: Preparation of Enynones

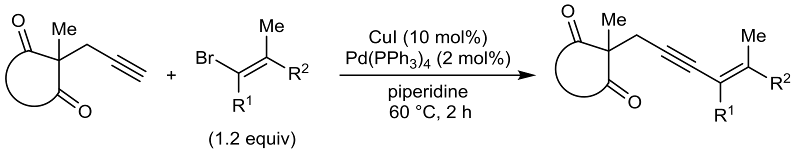

The appropriate vinyl bromide (1.2 equiv) was added to a solution of CuI (10 mol%) and $Pd(PPh_3)_4$ (2 mol%) in piperidine (0.33 M with respect to the alkyne). The appropriate alkyne (1 equiv) was added (neat) and the mixture was stirred at 60 °C for 2 h. The mixture was cooled to room temperature, and saturated aqueous NH_4Cl (30 mL) was added and the mixture was extracted with EtOAc (3 × 20 mL). The combined organic phases were washed with brine (20 mL), dried ($MgSO_4$), filtered, and concentrated *in vacuo*. The mixture was purified by column chromatography (20% EtOAc/petroleum ether) and the combined fractions were washed with activated charcoal, filtered through celite, and concentrated *in vacuo* to give the corresponding enynone.

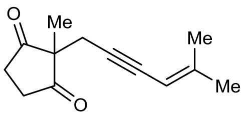

2-Methyl-2-(5-methylhex-4-en-2-yn-1-yl)cyclopentane-1,3-dione (1a). The title compound was prepared according to General Procedure A using 1-bromo-2-methyl-1-propene (2.5 mL, 24 mmol), CuI (381 mg, 2.00 mmol), $Pd(PPh_3)_4$ (462 mg, 0.400 mmol), 2-methyl-2-propargyl-1,3-cyclopentanedione[3] (3.00 g, 20.0 mmol) and piperidine (60 mL) to give a red oil (2.45 g, 60%). R_f = 0.28 (20% EtOAc/petroleum ether); IR 2909, 1727 (C=O), 1450, 1317, 1072 cm^{-1}; ^1H NMR (400 MHz, $CDCl_3$) δ 5.16–5.13 (1H, m, C=C**H**), 2.80 (4H, s, C**H₂**C**H₂**), 2.63 (2H, d, *J* = 2.0 Hz, C**H₂**C≡C), 1.81 (3H, s, C=CC**H₃**), 1.76 (3H, s, C=CC**H₃**), 1.14 (3H, s, O=CCC**H₃**); ^{13}C NMR (101 MHz, $CDCl_3$) δ 215.8 (2 × C), 148.8 (C), 104.5 (CH), 85.5 (C), 80.8 (C), 55.7 (C), 35.9 (2 × CH_2), 26.3 (CH_2), 24.7 (CH_3), 20.9 (CH_3), 18.8 (CH_3); HRMS (ESI) Exact mass calcd for $[C_{13}H_{16}NaO_2]^+$ $[M+Na]^+$: 227.1043, found: 227.1045.

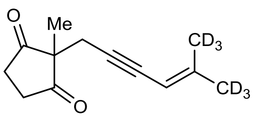

2-Methyl-2-(5-($^2H_3$)methyl(6,6,6-$^2H_3$)hex-4-en-2-yn-1-yl)cyclopentane-1,3-dione ([D₆]-1a). The title compound was prepared according to General Procedure A using 1-bromo-2-($^2H_3$)methyl(3,3,3-$^2H_3$)prop-1-ene[4] (215 mg, 1.53 mmol), CuI (24.2 mg, 0.127 mmol), $Pd(PPh_3)_4$ (29.4 mg, 0.0254 mmol), 2-methyl-2-propargyl-1,3-cyclopentanedione[3] (193 mg, 1.29 mmol) and piperidine (5 mL) to give a red oil (111

3. Prepared according to: P. T. Lansbury, A. K. Serelis, J. E. Hengeveld, D. G. Hangauer Jr, *Tetrahedron* **1980**, *36*, 2701-2710.
4. Prepared according to: D. J. Burns, H. W. Lam, *Angew. Chem., Int. Ed.* **2014**, *53*, 9931-9935.

mg, 41%). R_f = 0.32 (20% EtOAc/petroleum ether); IR 2933, 1727 (C=O), 1418, 1318, 1046 cm^{-1}; ^{1}H NMR (400 MHz, $CDCl_3$) δ 5.14 (1H, t, *J* = 2.2 Hz, C=C**H**), 2.80 (4H, s, C**H2**C**H2**), 2.62 (2H, d, *J* = 2.2 Hz, C**H2**C≡C), 1.14 (3H, s, O=CCC**H3**); ^{13}C NMR (101 MHz, $CDCl_3$) δ 215.8 (2 × C), 148.7 (C), 104.5 (CH), 85.4 (C), 80.8 (C), 55.7 (C), 35.9 (2 × CH_2), 26.3 (CH_2), 18.8 (CH_3); HRMS (ESI) Exact mass calcd for $C_{13}H_{10}D_6NaO_2$ [M+Na]$^+$: 233.1419, found: 223.1418.

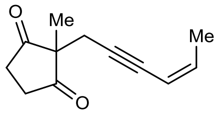

2-[(*Z*)-Hex-4-en-2-yn-1-yl]-2-methylcyclopentane-1,3-dione (1b). The title compound was prepared according to General Procedure A using (*Z*)-1-bromo-1-propene (0.85 mL, 10 mmol), CuI (127 mg, 0.667 mmol), $Pd(PPh_3)_4$ (154 mg, 0.133 mmol), 2-methyl-2-propargyl-1,3-cyclopentanedione[3] (1.00 g, 6.66 mmol) and piperidine (20 mL) to give an orange oil (803 mg, 63%). R_f = 0.29 (20% EtOAc/petroleum ether); IR 2935, 1726 (C=O), 1418, 1317, 1073 cm^{-1}; ^{1}H NMR (400 MHz, $CDCl_3$) δ 5.93 (1H, dq, *J* = 10.7, 6.8 Hz, CH=C**H**CH_3), 5.37 (1H, dtq, *J* = 10.7, 2.1, 1.7 Hz, C**H**=CHCH_3), 2.86–2.76 (4H, m, C**H2**C**H2**), 2.65 (2H, d, *J* = 2.1 Hz, C**H2**C≡C), 1.79 (3H, dd, *J* = 6.8, 1.7 Hz, CHC**H3**), 1.15 (3H, s, O=CCC**H3**); ^{13}C NMR (101 MHz, $CDCl_3$) δ 215.6 (2 × C), 138.8 (CH), 109.4 (CH), 88.3 (C), 79.4 (C), 55.6 (C), 35.8 (2 × CH_2), 26.0 (CH_2), 19.0 (CH_3), 15.8 (CH_3); HRMS (ESI) Exact mass calcd for $[C_{12}H_{14}NaO_2]^+$ [M+Na]$^+$: 213.0886, found: 213.0888.

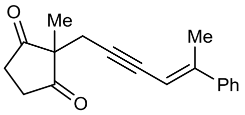

2-Methyl-2-[(*E*)-5-phenylhex-4-en-2-yn-1-yl]cyclopentane-1,3-dione (1c). The title compound was prepared according to General Procedure A using (*E*)-1-bromo-2-phenyl-1-propene[5] (2.00 g, 10.1 mmol), CuI (127 mg, 0.667 mmol), $Pd(PPh_3)_4$ (154 mg, 0.133 mmol), 2-methyl-2-propargyl-1,3-cyclopentanedione[3] (1.00 g, 6.66 mmol) and piperidine (20 mL) to give a brown solid (881 mg, 50%). R_f = 0.32 (20% EtOAc/petroleum ether); m.p. 68–69 °C (hexane); IR 2905, 1725 (C=O), 1415, 1312, 1073 cm^{-1}; ^{1}H NMR (400 MHz, $CDCl_3$) δ 7.42–7.37 (2H, m, Ar**H**), 7.36–7.27 (3H, m, Ar**H**), 5.78 (1H, tq, *J* = 2.3, 1.1 Hz, C=C**H**), 2.84 (4H, s, C**H2**C**H2**), 2.72 (2H, d, *J* = 2.3 Hz, C**H2**C≡C), 2.23 (3H, d, *J* = 1.1 Hz, C=CC**H3**), 1.18 (3H, s, O=CC**H3**); ^{13}C NMR (126 MHz, $CDCl_3$) δ 215.6 (2 × C), 148.6 (C), 140.6 (C), 128.4 (2 × CH), 128.1 (CH), 125.3 (2 × CH), 105.9 (CH), 89.6 (C), 81.2 (C), 55.7 (C), 35.9 (2 × CH_2), 26.2 (CH_2), 19.1 (CH_3), 18.5 (CH_3); HRMS (ESI) Exact mass calcd for $[C_{18}H_{18}NaO_2]^+$ [M+Na]$^+$: 289.1199, found: 289.1192.

5. Prepared according to: A. Spaggiari, D. Vaccari, P. Davoli, G. Torre, F. Prati, *J. Org. Chem.* **2007**, *72*, 2216-2219.

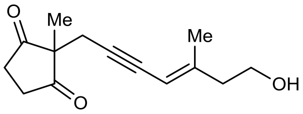

2-[(*E*)-7-Hydroxy-5-methylhept-4-en-2-yn-1-yl]-2-methylcyclopentane-1,3-dione (S11). The title compound was prepared according to General Procedure A using (*E*)-4-iodo-3-methylbut-3-en-1-ol[6] (1.45 g, 6.84 mmol), CuI (63.5 mg, 0.333 mmol), $Pd(PPh_3)_4$ (77.0 mg, 0.0666 mmol), 2-methyl-2-propargyl-1,3-cyclopentanedione[3] (500 mg, 3.33 mmol) and piperidine (20 mL). The crude mixture was purified by column chromatography (50% EtOAc/petroleum ether) to give a brown oil (539 mg, 69%). R_f = 0.13 (40% EtOAc/petroleum ether); IR 3478 (O–H), 2930, 1725 (C=O), 1419, 1317, 1048 cm^{-1}; ^1H NMR (400 MHz, $CDCl_3$) δ 5.25 (1H, tqt, *J* = 2.2, 1.2, 1.0 Hz, C=C**H**), 3.71 (2H, dt, *J* = 6.2, 5.0 Hz, C**H**$_2$OH), 2.89–2.73 (4H, m, C**H**$_2$C**H**$_2$), 2.64 (2H, d, *J* = 2.2 Hz, C**H**$_2$C≡C), 2.31 (2H, td, *J* = 6.2, 1.0 Hz, C=CC**H**$_2$), 1.84 (3H, d, *J* = 1.2 Hz, C=CC**H**$_3$), 1.36 (1H, t, *J* = 5.0 Hz, O**H**), 1.15 (3H, s, O=CCC**H**$_3$); ^{13}C NMR (126 MHz, $CDCl_3$) δ 215.5 (2 × C), 148.4 (C), 106.6 (CH), 86.8 (C), 80.2 (C), 60.3 (CH_2), 55.7 (C), 41.5 (CH_2), 35.8 (2 × CH_2), 26.0 (CH_2), 19.1 (CH_3), 19.0 (CH_3); HRMS (ESI) Exact mass calcd for $[C_{14}H_{18}NaO_3]^+$ $[M+Na]^+$: 257.1148, found: 257.1146.

(*E*)-3-Methyl-7-(1-methyl-2,5-dioxocyclopentyl)hept-3-en-5-yn-1-yl acetate (1d).

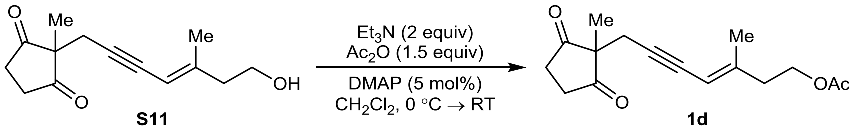

Acetic anhydride (0.27 mL, 2.9 mmol) was added dropwise to a solution of alcohol **S11** (445 mg, 1.90 mmol), DMAP (12.0 mg, 0.0982 mmol) and Et_3N (0.53 mL, 3.8 mmol) in CH_2Cl_2 (10 mL) at 0 °C. The mixture was warmed to room temperature and stirred for 1 h. Water (10 mL) and saturated aqueous NH_4Cl (2 mL) was added, and the mixture was extracted with CH_2Cl_2 (3 × 10 mL). The combined organic phases were dried ($MgSO_4$), filtered, and concentrated *in vacuo*. The mixture was purified by column chromatography (30% EtOAc/petroleum ether) to give *ester* **1d** (501 mg, 96%) as a pale yellow oil. R_f = 0.41 (30% EtOAc/petroleum ether); IR 2974, 1725 (C=O), 1364, 1241, 1047 cm^{-1}; ^1H NMR (500 MHz, $CDCl_3$) δ 5.21 (1H, tq, *J* = 2.2, 1.1 Hz, C=C**H**), 4.13 (2H, t, *J* = 6.7 Hz, C**H**$_2$O), 2.86–2.74 (4H, m, O=CC**H**$_2$C**H**$_2$), 2.63 (2H, d, *J* = 2.2 Hz, C**H**$_2$C≡C), 2.36 (2H, t, *J* = 6.7 Hz, C**H**$_2$CH$_2$O), 2.03 (3H, s, O=CC**H**$_3$), 1.84 (3H, d, *J* = 1.1 Hz, C=CC**H**$_3$), 1.14 (3H, s, O=CC**H**$_3$); ^{13}C NMR (101 MHz, $CDCl_3$) δ 215.6 (2 × C), 170.9 (C), 147.8 (C), 106.5 (CH), 87.0 (C), 80.2 (C), 62.0 (CH_2), 55.7 (C), 37.4 (CH_2), 35.8 (2 × CH_2), 26.0 (CH_2), 20.9 (CH_3), 19.2 (CH_3), 19.0 (CH_3); HRMS (ESI) Exact mass calcd for $[C_{16}H_{20}NaO_4]^+$ $[M+Na]^+$: 299.1254, found: 299.1261.

6. Prepared according to: M. Penner, V. Rauniyar, L. T. Kaspar, D. G. Hall, *J. Am. Chem. Soc.* **2009**, *131*, 14216-14217.

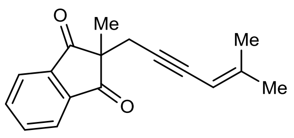

2-Methyl-2-(5-methylhex-4-en-2-yn-1-yl)-2,3-dihydro-1*H*-indene-1,3-dione (1e). The title compound was prepared according to General Procedure A using using 1-bromo-2-methyl-1-propene (0.37 mL, 3.6 mmol), CuI (57.6 mg, 0.302 mmol), $Pd(PPh_3)_4$ (70.0 mg, 0.0606 mmol), 2-methyl-2-(2-propynyl)-1,3-indandione[7] (600 mg, 3.03 mmol) and piperidine (10 mL) to give a yellow oil (462 mg, 60%). R_f = 0.45 (20% EtOAc/petroleum ether); IR 2910, 1710 (C=O), 1598, 1334, 1258 cm^{-1}; ^{1}H NMR (400 MHz, $CDCl_3$) δ 8.04–7.98 (2H, m, Ar**H**), 7.89–7.84 (2H, m, Ar**H**), 4.91 (1H, br s, C=C**H**), 2.84 (2H, d, J = 1.3 Hz, C**H₂**), 1.61 (3H, s, C=CC**H₃**), 1.38 (3H, s, C=CC**H₃**), 1.32 (3H, s, O=CCC**H₃**); ^{13}C NMR (101 MHz, $CDCl_3$) δ 203.0 (2 × C), 147.9 (C), 141.6 (2 × C), 135.7 (2 × CH), 123.4 (2 × CH), 104.5 (CH), 85.8 (C), 81.2 (C), 53.2 (C), 25.6 (CH_2), 24.5 (CH_3), 20.2 (CH_3), 18.8 (CH_3); HRMS (ESI) Exact mass calcd for $[C_{17}H_{17}O_2]^+$ $[M+H]^+$: 253.1223, found: 253.1236.

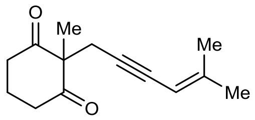

2-Methyl-2-(5-methylhex-4-en-2-yn-1-yl)cyclohexane-1,3-dione (1f). The title compound was prepared according to General Procedure A using using 1-bromo-2-methyl-1-propene (0.81 mL, 7.9 mmol), CuI (127 mg, 0.667 mmol), $Pd(PPh_3)_4$ (154 mg, 0.133 mmol), 2-methyl-2-propargyl-1,3-cyclohexanedione[8] (1.10 g, 6.70 mmol) and piperidine (22 mL) to give an orange oil (800 mg, 55%). R_f = 0.22 (15% EtOAc/petroleum ether); IR 2966, 1697 (C=O), 1312, 1205, 1027 cm^{-1}; ^{1}H NMR (400 MHz, $CDCl_3$) δ 5.16 (1H, br s, C=C**H**), 2.80 (2H, d, J = 1.4 Hz, C**H₂**C≡C), 2.71 (4H, t, J = 6.8 Hz, C**H₂**CH₂C**H₂**), 2.02 (1H, dquin, J = 13.9, 6.8, Hz, CH₂C**H₂**CH₂), 1.96 (1H, dquin, J = 13.9, 6.8 Hz, CH₂C**H₂**CH₂), 1.81 (3H, s, C=CC**H₃**), 1.76 (3H, s, C=CC**H₃**), 1.31 (3H, s, O=CCC**H₃**); ^{13}C NMR (101 MHz, $CDCl_3$) δ 209.3 (2 × C), 148.2 (C), 104.9 (CH), 86.9 (C), 81.1 (C), 64.3 (C), 38.4 (2 × CH_2), 27.0 (CH_2), 24.7 (CH_3), 21.2 (CH_3), 20.8 (CH_3), 17.3 (CH_2); HRMS (ESI) Exact mass calcd for $[C_{14}H_{18}NaO_2]^+$ $[M+Na]^+$: 241.1199, found: 241.1199.

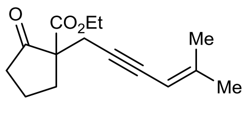

1-(5-Methylhex-4-en-2-yn-1-yl)-2-oxocyclopentane-1-carboxylate (10). The title compound was prepared according to General Procedure A using using 1-bromo-2-methyl-1-propene (0.63 mL, 6.2 mmol), CuI (98.0 mg, 0.515 mmol), $Pd(PPh_3)_4$ (119 mg, 0.103 mmol), 2-oxo-1-(prop-2-yn-1-yl)cyclopentane-1-carboxylate[9] (1.00 g, 5.15 mmol) and piperidine (15 mL) to give an orange oil (762 mg, 60%). R_f = 0.52 (20% EtOAc/petroleum ether); IR 2979, 1754 (C=O), 1727 (C=O), 1448, 1226, 1146 cm^{-1}; ^{1}H

7. Prepared according to: B. M. Partridge, J. Solana González, H. W. Lam, *Angew. Chem., Int. Ed.* **2014**, *53*, 6523-6527.
8. Prepared according to: K. Kato, C. Matsuba, T. Kusakabe, H. Takayama, S. Yamamura, T. Mochida, H. Akita, T. y. A. Peganova, N. V. Vologdin, O. V. Gusev, *Tetrahedron* **2006**, *62*, 9988-9999.
9. Prepared according to: F. Barabé, G. v. Bétournay, G. Bellavance, L. Barriault, *Org. Lett.* **2009**, *11*, 4236-4238.

NMR (400 MHz, $CDCl_3$) δ 5.19 (1H, s, C=C**H**), 4.18 (2H, q, *J* = 7.1 Hz, OC**H**$_2$), 2.90 (1H, dd, *J* = 17.1, 1.8 Hz, C**H**$_2$C≡C), 2.85 (1H, dd, *J* = 17.1, 2.0 Hz, C**H**$_2$C≡C), 2.56–2.43 (2H, m, C**H**$_2$CH$_2$C**H**$_2$), 2.38–2.23 (2H, m, C**H**$_2$CH$_2$C**H**$_2$), 2.16–1.98 (2H, m, CH$_2$C**H**$_2$CH$_2$), 1.84 (3H, s, C=CC**H**$_3$), 1.77 (3H, s, C=CC**H**$_3$), 1.26 (3H, t, *J* = 7.1 Hz, CH$_2$C**H**$_3$); ^{13}C NMR (101 MHz, $CDCl_3$) δ 214.0 (C), 170.5 (C), 148.0 (C), 105.0 (CH), 86.8 (C), 80.7 (C), 61.6 (CH_2), 59.2 (C), 38.4 (CH_2), 32.7 (CH_2), 24.6 (CH_3), 24.4 (CH_2), 20.8 (CH_3), 19.8 (CH_2), 14.0 (CH_3); HRMS (ESI) Exact mass calcd for $[C_{15}H_{20}NaO_3]^+$ $[M+Na]^+$: 271.1305, found: 271.1314.

8-Methylnon-7-en-5-yn-2-one (12a)

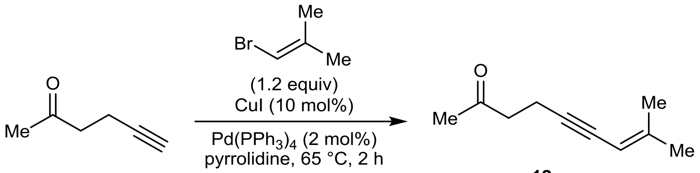

1-Bromo-2-methyl-1-propene (667 mg, 4.94 mmol) was added to a solution of CuI (94 mg, 0.49 mmol) and $Pd(PPh_3)_4$ (116 mg, 0.100 mmol) in pyrrolidine (15 mL) at room temperature. Hex-5-yn-2-one[10] (432 mg, 4.49 mmol) was added (neat, using *ca.* 1 mL of pyrrolidine to rinse) and the mixture was stirred at 60 °C for 2 h. The mixture was cooled to room temperature, saturated aqueous NH_4Cl solution (30 mL) added and the mixture extracted with EtOAc (3 × 20 mL). The combined organic phases were washed with brine (20 mL), dried (Na_2SO_4), filtered, and concentrated *in vacuo*. The mixture was purified by column chromatography (10% Et_2O/petroleum ether) to give *enynone* **12a** as a colorless oil (428 mg, 64%). R_f = 0.33 (10% Et_2O/petroleum ether); IR 2915, 1715 (C=O), 1377, 1364, 1161 cm^{-1}; ^{1}H NMR (400 MHz, $CDCl_3$) δ 5.22–5.19 (1H, m, C=C**H**), 2.72–2.66 (2H, m, C**H**$_2$C=O), 2.62–2.56 (2H, m, C**H**$_2$C≡C), 2.18 (3H, s, C**H**$_3$C=O), 1.86–1.84 (3H, m, C=CC**H**$_3$), 1.78–1.76 (3H, m, C=CC**H**$_3$); ^{13}C NMR (101 MHz, $CDCl_3$) δ 206.8 (C), 147.4 (C), 105.1 (CH), 90.1 (C), 78.9 (C), 42.9 (CH_2), 29.9 (CH_3), 24.6 (CH_3), 20.7 (CH_3), 14.2 (CH_2); HRMS (GC-MS, EI) Exact mass calcd for $[C_{10}H_{14}O]^+$ $[M]^+$: 150.1040, found: 150.1039.

10. Prepared according to: C. Görl, H. G. Alt, *J. Organomet. Chem.* **2007**, 692, 5727-5753, but using propargyl bromide (80% wt. in toluene) in place of propargyl chloride.

Preparation of Enynone 12b

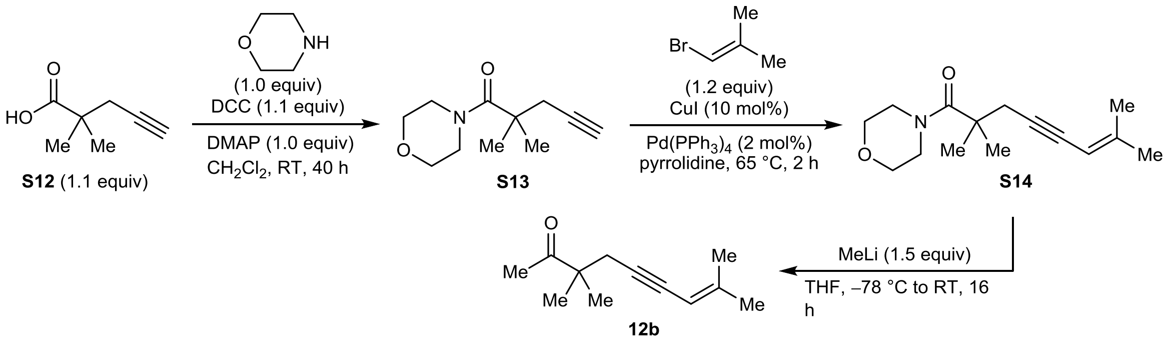

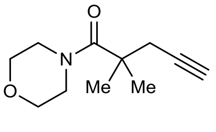

2,2-Dimethyl-1-(morpholin-4-yl)pent-4-yn-1-one (S13). DCC (4.56 g, 22.1 mmol) was added to a solution of terminal alkyne **S12**[11] (2.79 g, 22.1 mmol), morpholine (1.76 mL, 20.1 mmol), and DMAP (2.46 g, 20.1 mmol) in CH_2Cl_2 (60 mL) at 0 °C. The mixture was allowed to warm to room temperature and stirred for 40 h. The resulting suspension was filtered through a pad of silica using EtOAc as eluent, and concentrated *in vacuo*. The mixture was purified by column chromatography (40% EtOAc/petroleum ether) to give *alkyne* **S13** as a yellow solid (2.33 g, 59%). R_f = 0.36 (40% EtOAc/petroleum ether); m.p. 63–64 °C (Et_2O); IR 3226, 2930, 1611 (C=O), 1423, 1109 cm^{-1}; ^{1}H NMR (400 MHz, $CDCl_3$) δ 3.69–3.60 (8H, m, 2 × OC**H**$_2$C**H**$_2$N), 2.50 (2H, d, *J* = 2.6 Hz, C**H**$_2$C≡C), 2.01 (1H, t, *J* = 2.6, C≡C**H**), 1.36 (6H, s, 2 × C**H**$_3$); ^{13}C NMR (101 MHz, $CDCl_3$) δ 174.4 (C), 81.7 (C), 70.5 (CH), 66.7 (2 × CH_2), 45.7 (CH_2), 41.7 (C), 31.3 (2 × CH_2), 24.8 (2 × CH_3); HRMS (ESI) Exact mass calcd for $[C_{11}H_{18}NO_2]^+$ $[M+H]^+$: 196.1332, found: 196.1336.

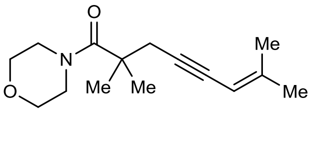

2,2,7-Trimethyl-1-(morpholin-4-yl)oct-6-en-4-yn-1-one (S14). 1-Bromo-2-methyl-1-propene (1.14 g, 8.45 mmol) was added to a solution of CuI (146 mg, 0.768 mmol) and $Pd(PPh_3)_4$ (178 mg, 0.154 mmol) in pyrrolidine (25 mL). Alkyne **S13** (1.50 g, 7.68 mmol) was added (neat, using *ca.* 1 mL of pyrrolidine to rinse) and the mixture was stirred at 60 °C for 2 h. The mixture was cooled to room temperature, saturated aqueous NH_4Cl solution (30 mL) added and the mixture extracted with EtOAc (3 × 20 mL). The combined organic phases were washed with brine (20 mL), dried (Na_2SO_4), filtered, and concentrated *in vacuo*. The mixture was purified by column chromatography (30% EtOAc/petroleum ether) to give *enyne* **S14** as a yellow oil (1.91 g, 99%). R_f = 0.35 (30% EtOAc/petroleum ether); IR 3226, 2964, 1611 (C=O), 1423, 1109, 981 cm^{-1}; ^{1}H NMR (400 MHz, $CDCl_3$) δ 5.26–5.23 (1H, m, C=C**H**), 3.73–3.62 (8H, m, 2 × OC**H**$_2$C**H**$_2$N), 2.70–2.65 (2H, br, s,

11. Prepared according to: R. G. Iafe, J. L. Kuo, D. G. Hochstatter, T. Saga, J. W. Turner, C. A. Merlic, *Org. Lett.* **2013**, 15, 582-585.

C**H**$_2$C≡C), 1.87 (3H, s, C=CC**H**$_3$), 1.78 (3H, s, C=CC**H**$_3$), 1.38 (6H, s, (C**H**$_3$)$_2$CC=O); ^{13}C NMR (101 MHz, CDCl$_3$) δ 174.8 (C), 147.3 (C), 105.3 (CH), 88.6 (C), 80.8 (C), 66.8 (2 × CH$_2$), 45.8 (CH$_2$), 42.2 (C), 32.0 (2 × CH$_2$), 25.3 (2 × CH$_3$), 24.6 (CH$_3$), 20.9 (CH$_3$); HRMS (ESI) Exact mass calcd for $[C_{15}H_{24}NO_2]^+$ $[M+H]^+$: 250.1802, found: 250.1790.

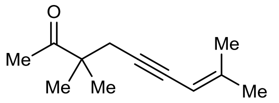

3,3,8-Trimethylnon-7-en-5-yn-2-one (12b). MeLi (1.15 M in Et$_2$O, 2.95 mL, 3.39 mmol) was added dropwise to a solution of morpholine amide **S14** (500 mg, 2.26 mmol) in THF (10 mL) at −78 °C. The solution was stirred for 16 h while being allowed to warm to room temperature. The reaction was quenched with saturated aqueous NH$_4$Cl solution (20 mL) and extracted with EtOAc (3 × 20 mL). The combined organic phases were washed with brine (20 mL), dried (Na$_2$SO$_4$), filtered, and concentrated *in vacuo*. The mixture was purified by column chromatography (10% Et$_2$O/petroleum ether) to give *enynone* **12b** as a colorless oil (295 mg, 73%). R_f = 0.29 (10% Et$_2$O/petroleum ether); IR 2971, 1706 (C=O), 1464, 1354, 1118 cm^{-1}; ^{1}H NMR (400 MHz, CDCl$_3$) δ 5.24–5.20 (1H, m, C=C**H**), 2.55–2.52 (2H, br s, C**H**$_2$C≡C), 2.21–2.18 (3H, m, C**H**$_3$C=O), 1.85 (3H, s, C=CC**H**$_3$), 1.78 (3H, s, C=CC**H**$_3$), 1.24–1.22 (6H, m, (C**H**$_3$)$_2$CC=O); ^{13}C NMR (101 MHz, CDCl$_3$) 212.4 (C), 147.5 (C), 105.1 (CH), 88.2 (C), 80.8 (C), 47.7 (C), 30.0 (CH$_2$), 25.3 (CH$_3$), 24.6 (CH$_3$), 24.0 (2 × CH$_3$), 20.8 (CH$_3$); HRMS (ESI) Exact mass calcd for $[C_{12}H_{19}O]^+$ $[M+H]^+$: 179.1430, found: 179.1423.

2-Methyl-2-[(*E*)-(5-methylhex-4-en-2-yn-1-yl)]cyclopentane-1,3-dione (14)

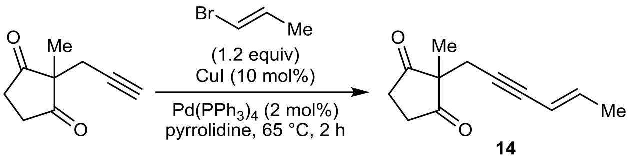

trans-1-Bromo-1-propene (443 mg, 3.66 mmol) was added to a solution of CuI (63 mg, 0.33 mmol) and Pd(PPh$_3$)$_4$ (77 mg, 0.067 mmol) in pyrrolidine (10 mL). 2-Methyl-2-propargyl-1,3-cyclopentanedione[3] (500 mg, 3.33 mmol) was added (neat, using *ca.* 1 mL of pyrrolidine to rinse) and the mixture was stirred at 60 °C for 2 h. The mixture was cooled to room temperature, saturated aqueous NH$_4$Cl solution (30 mL) added and the mixture extracted with EtOAc (3 × 20 mL). The combined organic phases were washed with brine (20 mL), dried (Na$_2$SO$_4$), filtered, and concentrated *in vacuo*. The mixture was purified by column chromatography (20% EtOAc/petroleum ether) to give *enynone* **14** as an orange oil (379 mg, 60%). R_f = 0.21 (20% EtOAc/petroleum ether); IR 2915, 1723 (C=O), 1317, 1072, 953 cm^{-1}; ^{1}H NMR (400 MHz, CDCl$_3$) δ 6.05 (1H, dq, J = 15.8, 6.7 Hz, =C**H**CH$_3$), 5.37 (1H, dq, J = 15.8, 2.1 Hz, C**H**=CHCH$_3$), 2.80 (4H, s, C**H**$_2$C**H**$_2$), 2.56 (2H, d, J = 2.1 Hz, C**H**$_2$C≡C), 1.74 (3H, dt, J = 6.7, 0.9 Hz, =CHC**H**$_3$), 1.12 (3H,

s, CCH_3); ^{13}C NMR (101 MHz, $CDCl_3$) δ 215.7 (2 × C), 139.9 (CH), 110.0 (CH), 81.9 (C), 81.6 (C), 55.5 (C), 35.9 (2 × CH_2), 26.1 (CH_2), 18.8 (CH_3), 18.5 (CH_3); HRMS (ESI) Exact mass calcd for $[C_{12}H_{15}O_2]^+$ $[M+H]^+$: 191.1067, found: 191.1061.

3. Rhodium-Catalyzed Arylative Allylations

(±)-(3a*R*,4*R*,7a*R*)-6-(3,5-Dimethylphenyl)-3a-hydroxy-7a-methyl-4-(prop-1-en-2-yl)-2,3,3a,4,7,7a-hexahydro-1*H*-inden-1-one (2aa), (±)-(3a*R*,4*S*,7a*R*)-6-(3,5-dimethylphenyl)-3a-hydroxy-7a-methyl-4-(prop-1-en-2-yl)-2,3,3a,4,7,7a-hexahydro-1*H*-inden-1-one (2ab), and (*Z*)-5-[1-(3,5-dimethylphenyl)-3-methylbut-2-en-1-ylidene]-7-methylcycloheptane-1,4-dione (3a)

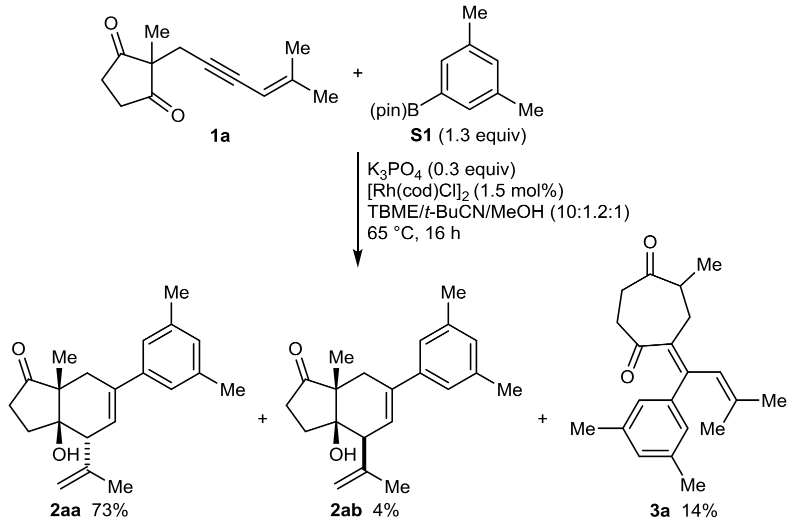

$[Rh(cod)Cl]_2$ (3.7 mg, 7.5 µmol) was added to an oven-dried microwave vial, and the vial was sealed with a septum and purged with nitrogen for 1 h. Degassed TBME (1 mL) and degassed *t*-BuCN (1 mL) were added and the mixture was stirred for 30 min at room temperature. Meanwhile, in a separate oven-dried microwave vial, enynone **1a** (102 mg, 0.499 mmol), 3,5-dimethylphenylboronic acid pinacol ester **S1** (151 mg, 0.650 mmol), and K_3PO_4 (31.8 mg, 0.150 mmol) were added. The vial was sealed with a septum-lined microwave vial cap and purged with nitrogen for 1 h. Degassed TBME (7.3 mL), the solution of catalyst (see above), and degassed MeOH (0.83 mL) were added, and the mixture was stirred at 65 °C for 16 h. The reaction was cooled to room temperature, water (10 mL) and saturated aqueous NH_4Cl solution (10 mL) were added, and the mixture was extracted with EtOAc (3 x 15 mL. The combined organic phases were washed with brine (30 mL), dried ($MgSO_4$), filtered, and concentrated *in vacuo*. The mixture was purified by column chromatography (94% $CHCl_3$, 5% EtOAc, 1% Et_3N) to give *ketone* **3a** (22.1 mg, 14%) as a pale yellow solid, followed by *alcohol* **2ab** (5.7 mg, 4%) as a yellow oil, followed by *alcohol* **2aa** (114 mg, 73%) as a white solid.

Data for **2aa**: R_f = 0.15 (95% $CHCl_3$, 5% Et_2O); m.p. 162–163 °C (Et_2O); IR 3501 (O–H), 2967, 1733 (C=O), 1599, 1457, 1373, 1060 cm^{-1}; ^{1}H NMR (400 MHz, $CDCl_3$) δ 6.98 (2H, s, Ar**H**), 6.93 (1H, s, Ar**H**), 5.95 (1H, t, *J* = 2.5 Hz, C=C**H**), 5.11 (1H, quin, *J* = 1.5 Hz, C=C**H**$_A$H$_B$), 4.95 (1H, br s, C=CH$_A$**H**$_B$), 3.18 (1H, dt, *J* = 3.9, 1.7 Hz, C=CC**H**), 2.57-2.39 (3H, m, O=CC**H**$_2$ and

C=CC**H$_A$**H$_B$), 2.32 (6H, s, 2 × ArC**H$_3$**), 2.23 (1H, dd, *J* = 18.0, 1.7 Hz, C=CCH$_A$**H$_B$**), 2.10 (1H, dt, *J* = 13.5, 9.8 Hz, HOCC**H$_A$**H$_B$), 1.91 (3H, br s, C=CC**H$_3$**), 1.81 (1H, s, O**H**), 1.76 (1H, ddd, *J* = 13.5, 8.1, 2.3 Hz, HOCCH$_A$**H$_B$**), 1.16 (3H, s, O=CCC**H$_3$**); ^{13}C NMR (101 MHz, $CDCl_3$) δ 218.6 (C), 144.3 (C), 140.4 (C), 137.9 (2 × C), 133.1 (C), 129.1 (CH), 124.4 (CH), 123.1 (2 × CH), 115.7 (CH_2), 79.5 (C), 52.9 (C), 50.6 (CH), 35.7 (CH_2), 32.8 (CH_2), 29.5 (CH_2), 22.9 (CH_3), 21.3 (2 × CH_3), 13.2 (CH_3); HRMS (ESI) Exact mass calcd for $C_{21}H_{26}NaO_2$ $[M+Na]^+$: 333.1825, found: 333.1818.

Cooling a solution of **2aa** in Et_2O at –20 °C provided crystals that were suitable for X-ray diffraction:

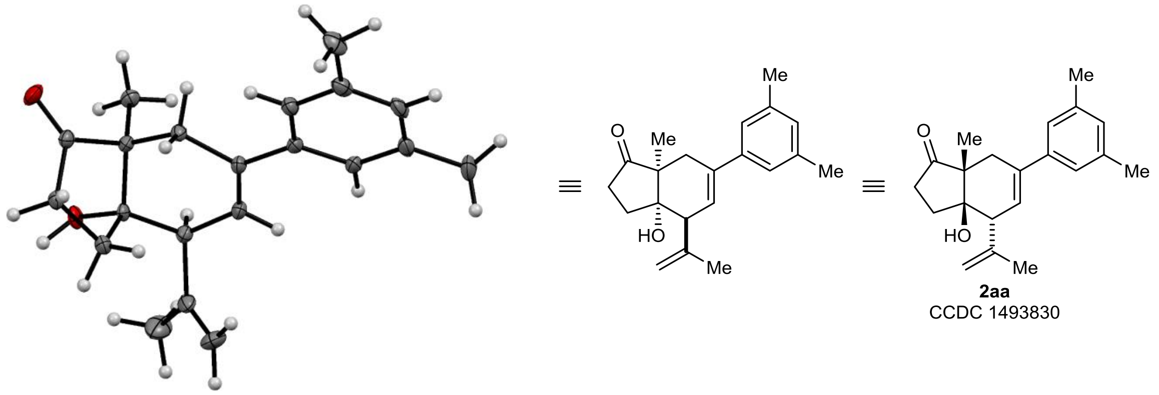

2aa
CCDC 1493830

Data for **2ab**: R_f = 0.53 (94% $CHCl_3$, 5% EtOAc, 1% Et_3N); IR 3477 (O–H), 2971, 1739 (C=O), 1374, 1046 cm^{-1}; ^{1}H NMR (400 MHz, $CDCl_3$) δ 7.00 (2H, s, Ar**H**), 6.93 (1H, s, Ar**H**), 5.93 (1H, t, *J* = 2.8 Hz, C=C**H**), 5.19 (1H, br s, C=C**H$_A$**H$_B$), 5.00 (1H, s, C=CH$_A$**H$_B$**), 3.13 (1H, br s, C=CC**H**), 2.64–2.51 (2H, m, O=CC**H$_A$**H$_B$ and C=CC**H$_A$**H$_B$), 2.43 (1H, ddd, *J* = 19.4, 9.0, 4.4 Hz, O=CCH$_A$**H$_B$**), 2.36–2.27 (1H, m, C=CCH$_A$**H$_B$**), 2.33 (6H, s, 2 × ArC**H$_3$**), 2.22 (1H, ddd, 13.4, 9.0, 8.1, HOCC**H$_A$**H$_B$), 2.13 (1H, ddd, *J* = 13.4, 9.0, 4.4 Hz, HOCCH$_A$**H$_B$**), 2.00 (1H, s, O**H**), 1.96 (3H, s, C=CC**H$_3$**), 1.18 (3H, s, O=CCC**H$_3$**); ^{13}C NMR (101 MHz, $CDCl_3$) δ 218.8 (C), 145.2 (C), 140.9 (C), 137.9 (2 × C), 134.3 (C), 129.0 (CH), 125.1 (CH), 123.2 (2 × CH), 117.0 (CH_2), 77.9 (C), 52.7 (C), 51.5 (CH), 34.2 (CH_2), 33.7 (CH_2), 33.4 (CH_2), 24.4 (CH_3), 21.3 (2 × CH_3), 16.7 (CH_3); HRMS (ESI) Exact mass calcd for $[C_{21}H_{26}NaO_2]^+$ $[M+Na]^+$: 333.1825, found: 333.1819.

The relative configuration of **2ab** could not be determined unambiguously by NOESY NMR spectroscopy (see page 57), but was assigned tentatively by analogy with **9cb** (NOESY NMR spectrum on page 82), the relative configuration of which was determined by X-ray crystallography (see page 21).

Data for **3a**: R_f = 0.61 (94% $CHCl_3$, 5% EtOAc, 1% Et_3N); m.p. 97–98 °C (EtOAc/petroleum ether); IR 2981, 1710 (C=O), 1688 (C=O), 1447, 1197, 1049 cm^{-1}; ^{1}H NMR (400 MHz, $CDCl_3$) δ

6.87 (1H, s, Ar**H**), 6.70 (2H, s, Ar**H**), 5.90 (1H, s, C=C**H**), 2.88–2.76 (3H, m, C**H**CH_3 and C$\mathbf{H_2}$C(O)CH), 2.69 (1H, dd, J = 15.1, 5.0 Hz, CHC$\mathbf{H_A}H_B$), 2.65–2.52 (2H, m, CC(O)C$\mathbf{H_2}$), 2.27 (6H, s, 2 × ArC$\mathbf{H_3}$), 2.24 (1H, m, J = 15.1, 11.7 Hz, CHC$H_A\mathbf{H_B}$), 1.84 (3H, d, J = 1.0 Hz, C=CC$\mathbf{H_3}$), 1.49 (3H, d, J = 0.8 Hz, C=CC$\mathbf{H_3}$), 1.16 (3H, d, J = 6.7 Hz, CHC$\mathbf{H_3}$); ^{13}C NMR (101 MHz, $CDCl_3$) δ 211.9 (C), 207.5 (C), 141.6 (C), 140.4 (C), 139.0 (C), 137.6 (2 × C), 137.3 (C), 129.2 (CH), 125.8 (2 × CH), 123.0 (CH), 44.8 (CH), 39.0 (CH_2), 37.5 (CH_2), 33.9 (CH_2), 26.1 (CH_3), 21.3 (2 × CH_3), 19.8 (CH_3), 15.7 (CH_3); HRMS (ESI) Exact mass calcd for $[C_{21}H_{27}O_2]^+$ $[M+H]^+$: 311.2006, found: 311.2007.

General Procedure B: Arylative Allylation Reaction

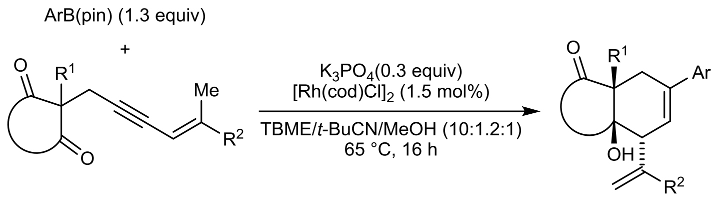

$[Rh(cod)Cl]_2$ (3.7 mg, 7.5 µmol) was added to an oven-dried microwave vial, and the vial was sealed with a septum and purged with nitrogen for 1 h. Degassed TBME (1 mL) and degassed *t*-BuCN (1 mL) were added and the mixture was stirred for 30 min at room temperature. Meanwhile, in a separate oven-dried microwave vial, enynone (0.50 mmol), arylboronic acid pinacol ester (0.65 mmol), and K_3PO_4 (31.8 mg, 0.150 mmol) were added. The vial was sealed with a septum-lined microwave vial cap and purged with nitrogen for 1 h. Degassed TBME (7.3 mL), the solution of catalyst (see above), and degassed MeOH (0.83 mL) were added, and the mixture was stirred at 65 °C for 16 h. The reaction was cooled to room temperature, water (10 mL) and saturated aqueous NH_4Cl solution (10 mL) were added, and the mixture was extracted with EtOAc (3 x 15 mL. The combined organic phases were washed with brine (30 mL), dried ($MgSO_4$), filtered, and concentrated *in vacuo*. The mixture was purified by column chromatography to give the corresponding alcohol.

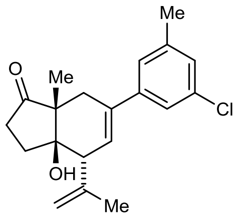

(±)-(3a*R*,4*R*,7a*R*)-6-(3-Chloro-5-methylphenyl)-3a-hydroxy-7a-methyl-4-(prop-1-en-2-yl)-2,3,3a,4,7,7a-hexahydro-1*H*-inden-1-one (2ba). General Procedure B was followed using enynone **1a** (102 mg, 0.498 mmol) and 3-chloro-5-methylphenylboronic acid pinacol ester **S2** (164 mg, 0.649 mmol). Column chromatography (94% $CHCl_3$, 5% EtOAc, 1% Et_3N) of the crude material gave *alcohol* **2ba** (118 mg, 72%) as a pale orange solid. R_f = 0.20 (94% $CHCl_3$, 5% EtOAc, 1% Et_3N); m.p. 141–142 °C (EtOAc/petroleum ether); IR 3495 (O–H), 2912, 1727 (C=O), 1597,

1434, 1374, 1078 cm^{-1}; ^{1}H NMR (400 MHz, $CDCl_3$) δ 7.14 (1H, t, *J* = 2.1 Hz, Ar**H**), 7.08 (1H, s, Ar**H**), 7.02 (1H, s, Ar**H**), 5.97 (1H, t, *J* = 2.3 Hz, C=C**H**), 5.13 (1H, quin, *J* = 1.5 Hz, C=C$\mathbf{H_A}H_B$), 4.95–4.93 (1H, m, C=C$H_A\mathbf{H_B}$), 3.18 (1H, ddd, *J* = 4.2, 2.3, 1.9 Hz, C=CC**H**), 2.57–2.37 (3H, m, O=CC$\mathbf{H_2}$ and C=CC$\mathbf{H_A}H_B$), 2.33 (3H, s, ArC$\mathbf{H_3}$), 2.19 (1H, dd, *J* = 17.9, 1.3 Hz, C=CC$H_A\mathbf{H_B}$), 2.08 (1H, ddd, *J* = 13.6, 9.8, 5.4 Hz, HOCC$\mathbf{H_A}H_B$), 1.92–1.91 (3H, m, C=CC$\mathbf{H_3}$), 1.77 (1H, ddd, *J* = 13.6, 7.9, 2.4 Hz, HOCC$H_A\mathbf{H_B}$), 1.77 (1H, s, O**H**), 1.16 (3H, s, O=CCC$\mathbf{H_3}$); ^{13}C NMR (101 MHz, $CDCl_3$) δ 218.2 (C), 144.0 (C), 142.1 (C), 139.8 (C), 134.2 (C), 132.2 (C), 128.1 (CH), 125.9 (CH), 124.2 (CH), 122.6 (CH), 115.9 (CH_2), 79.4 (C), 52.8 (C), 50.6 (CH), 35.5 (CH_2), 32.7 (CH_2), 29.5 (CH_2), 22.9 (CH_3), 21.2 (CH_3), 13.3 (CH_3); HRMS (ESI) Exact mass calcd for $[C_{20}H_{23}{}^{35}ClNaO_2]^+$ $[M+Na]^+$: 353.1284, found: 353.1275.

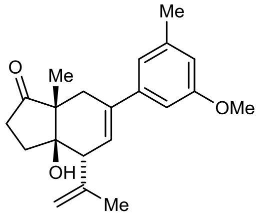

(±)-(3a*R*,4*R*,7a*R*)-3a-Hydroxy-6-(3-methoxy-5-methylphenyl)-7a-methyl-4-(prop-1-en-2-yl)-2,3,3a,4,7,7a-hexahydro-1*H*-inden-1-one (2ca). General Procedure B was followed using enynone **1a** (103 mg, 0.505 mmol) and 3-methyl-5-methoxyphenylboronic acid pinacol ester **S3** (161 mg, 0.649 mmol). Column chromatography (94% $CHCl_3$, 5% EtOAc, 1% Et_3N) of the crude material gave *alcohol* **2ca** (111 mg, 67%) as a pale yellow solid. R_f = 0.19 (94% $CHCl_3$, 5% EtOAc, 1% Et_3N); m.p. 139–140 °C (EtOAc/petroleum ether); IR 3495 (O–H), 2968, 1726 (C=O), 1591, 1158, 1061 cm^{-1}; ^{1}H NMR (400 MHz, $CDCl_3$) δ 6.77 (1H, s, Ar**H**), 6.70 (1H, t, *J* = 1.8 Hz, Ar**H**), 6.66 (1H, br s, Ar**H**), 5.97 (1H, t, *J* = 2.3 Hz, C=C**H**), 5.12 (1H, quin, *J* = 1.5 Hz, C=C$\mathbf{H_A}H_B$), 4.96–4.93 (1H, m, C=C$H_A\mathbf{H_B}$), 3.81 (3H, s, OC$\mathbf{H_3}$), 3.21–3.16 (1H, m, C=CC**H**), 2.57–2.39 (3H, m, O=CC$\mathbf{H_2}$ and C=CC$\mathbf{H_A}H_B$), 2.34 (3H, s, ArC$\mathbf{H_3}$), 2.23 (1H, dd, *J* = 18.0, 1.5 Hz, C=CC$H_A\mathbf{H_B}$), 2.10 (1H, dt, *J* = 13.5, 9.8 Hz, HOCC$\mathbf{H_A}H_B$), 1.92 (3H, dd, *J* = 1.4, 0.8 Hz, C=CC$\mathbf{H_3}$), 1.79 (1H, s, O**H**), 1.77 (1H, ddd, *J* = 13.5, 8.1, 2.3 Hz, HOCC$H_A\mathbf{H_B}$), 1.16 (3H, s, O=CCC$\mathbf{H_3}$); ^{13}C NMR (101 MHz, $CDCl_3$) δ 218.5 (C), 159.7 (C), 144.2 (C), 141.8 (C), 139.5 (C), 133.0 (C), 124.8 (CH), 118.6 (CH), 115.8 (CH_2), 113.6 (CH), 108.3 (CH), 79.5 (C), 55.2 (CH_3), 52.9 (C), 50.6 (CH), 35.6 (CH_2), 32.8 (CH_2), 29.5 (CH_2), 22.8 (CH_3), 21.6 (CH_3), 13.2 (CH_3); HRMS (ESI) Exact mass calcd for $[C_{21}H_{27}O_3]^+$ $[M+H]^+$: 327.1955, found: 327.1947.

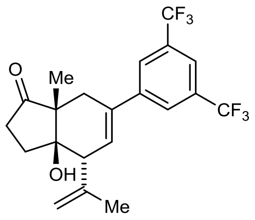

(±)-(3a*R*,4*R*,7a*R*)-6-[3,5-Bis(trifluoromethyl)phenyl]-3a-hydroxy-7a-methyl-4-(prop-1-en-2-yl)-2,3,3a,4,7,7a-hexahydro-1*H*-inden-1-one (2da). General Procedure B was followed using enynone **1a** (103 mg, 0.503 mmol) and 3,5-bis(trifluoromethyl)phenylboronic acid pinacol ester **S4** (203 mg, 0.597 mmol). Column chromatography (94% $CHCl_3$, 5% EtOAc, 1% Et_3N) of

the crude material gave *alcohol* **2da** (138 mg, 66%) as a pale yellow solid. R_f = 0.20 (94% $CHCl_3$, 5% EtOAc, 1% Et_3N); m.p. 139–140 °C (EtOAc); IR 3408 (O–H), 1985, 1727 (C=O), 1275, 1136 cm^{-1}; ^{1}H NMR (400 MHz, $CDCl_3$) δ 7.78 (1H, s, Ar**H**), 7.76 (2H, s, Ar**H**), 6.14 (1H, t, *J* = 2.5 Hz, C=C**H**), 5.17 (1H, quin, *J* = 1.4 Hz, C=C**H**$_A$H$_B$), 4.95 (1H, s, C=CH$_A$**H**$_B$), 3.24 (1H, ddd, *J* = 4.2, 2.5, 1.4 Hz, C=CC**H**), 2.62–2.44 (3H, m, O=CC**H**$_2$ and C=CC**H**$_A$H$_B$), 2.22 (1H, dd, *J* = 17.9, 1.4 Hz, C=CCH$_A$**H**$_B$), 2.10 (1H, ddd, *J* = 13.7, 10.0, 9.8 Hz, HOCC**H**$_A$H$_B$), 1.94 (3H, s, C=CC**H**$_3$), 1.83 (1H, ddd, *J* = 13.7, 8.0, 2.4 Hz, HOCCH$_A$**H**$_B$), 1.78 (1H, s, O**H**), 1.19 (3H, s, O=CCC**H**$_3$); ^{13}C NMR (101 MHz, $CDCl_3$) δ 217.8 (C), 143.4 (C), 142.5 (C), 131.8 (q, J_F = 33.0 Hz, 2 × C), 131.1 (C), 128.6 (CH), 125.2–125.1 (m, 2 × CH), 123.2 (q, J_F = 273.0 Hz, 2 × C), 121.1 (sept, J_F = 3.8 Hz, CH), 116.3 (CH_2), 79.2 (C), 52.8 (C), 50.7 (CH), 35.2 (CH_2), 32.7 (CH_2), 29.7 (CH_2), 23.1 (CH_3), 13.3 (CH_3); ^{19}F NMR (376 MHz, $CDCl_3$) δ –62.9; HRMS (ESI) Exact mass calcd for $[C_{21}H_{20}F_6NaO_2]^+$ $[M+Na]^+$: 441.1265, found: 441.1269.

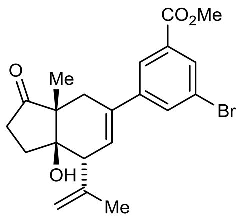

(±)-3-[(3a*R*,7*R*,7a*R*)-7a-Hydroxy-3a-methyl-3-oxo-7-(prop-1-en-2-yl)-2,3,3a,4,7,7a-hexahydro-1*H*-inden-5-yl]-5-bromobenzoate (2ea). General Procedure B was followed using enynone **1a** (102 mg, 0.497 mmol) and methyl 3-bromo-5-(tetramethyl-1,3,2-dioxaborolan-2-yl)benzoate **S5** (222 mg, 0.651 mmol). Column chromatography (94% $CHCl_3$, 5% EtOAc, 1% Et_3N) of the crude material gave *alcohol* **2ea** (117 mg, 56%) as a pale yellow solid. R_f = 0.16 (94% $CHCl_3$, 5% EtOAc, 1% Et_3N); m.p. 200–201 °C (EtOAc); IR 3503 (O–H), 1740 (C=O), 1705 (C=O), 1435, 1288, 1241, 1123 cm^{-1}; ^{1}H NMR (400 MHz, $CDCl_3$) δ 8.07 (1H, dd, *J* = 1.8, 1.4 Hz, Ar**H**), 7.93 (1H, dd, *J* = 1.8, 1.4 Hz, Ar**H**), 7.67 (1H, t, *J* = 1.8 Hz, Ar**H**), 6.07 (1H, t, *J* = 2.4 Hz, C=C**H**), 5.14 (1H, quin, *J* = 1.5 Hz, C=C**H**$_A$H$_B$), 4.94 (1H, s, C=CH$_A$**H**$_B$), 3.94 (3H, s, OC**H**$_3$), 3.21 (1H, ddd, *J* = 3.9, 2.4, 1.5 Hz, C=CC**H**) 2.59–2.41 (3H, m, O=CC**H**$_2$ and C=CC**H**$_A$H$_B$), 2.21 (1H, dd, *J* = 17.9, 1.5 Hz, C=CCH$_A$**H**$_B$), 2.08 (1H, dt, *J* = 13.6, 9.7 Hz, HOCC**H**$_A$H$_B$), 1.92 (3H, s, C=CC**H**$_3$), 1.80 (1H, ddd, *J* = 13.6, 8.0, 2.3 Hz, HOCCH$_A$**H**$_B$), 1.78 (1H, s, O**H**), 1.17 (3H, s, O=CCC**H**$_3$); ^{13}C NMR (101 MHz, $CDCl_3$) δ 217.9 (C), 165.6 (C), 143.6 (C), 142.6 (C), 132.5 (CH), 132.0 (C), 131.4 (C), 131.3 (CH), 127.3 (CH), 125.0 (CH), 122.7 (C), 116.2 (CH_2), 79.3 (C), 52.8 (C), 52.5 (CH_3), 50.6 (CH), 35.3 (CH_2), 32.7 (CH_2), 29.6 (CH_2), 23.0 (CH_3), 13.3 (CH_3); HRMS (ESI) Exact mass calcd for $[C_{21}H_{23}{}^{79}BrNaO_4]^+$ $[M+Na]^+$: 441.0672, found: 441.0675.

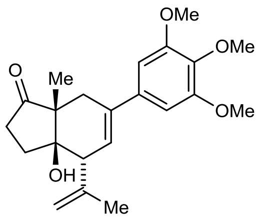

(±)-(3a*R*,4*R*,7a*R*)-3a-Hydroxy-7a-methyl-4-(prop-1-en-2-yl)-6-(3,4,5-trimethoxyphenyl)-2,3,3a,4,7,7a-hexahydro-1*H*-inden-1-one (2fa). General Procedure B was followed using enynone **1a** (103 mg, 0.502 mmol) and 3,4,5-trimethoxyphenylboronic acid pinacol ester **S6** (191 mg, 0.649 mmol). Column chromatography (94% $CHCl_3$, 5% EtOAc, 1% Et_3N) of the crude material gave *alcohol* **2fa** (146 mg, 78%) as a pale yellow solid. R_f = 0.11 (94% $CHCl_3$, 5% EtOAc, 1% Et_3N); m.p. 166–167 °C (EtOAc); IR 3519 (O–H), 2934, 1745 (C=O), 1580, 1413, 1243, 1119, 1002 cm^{-1}; ^1H NMR (400 MHz, $CDCl_3$) δ 6.55 (2H, s, Ar**H**), 5.93 (1H, t, *J* = 2.5 Hz, C=C**H**), 5.13 (1H, dq, *J* = 2.8, 1.5 Hz, C=C**H**$_A$H$_B$), 4.95 (1H, s, C=CH$_A$**H**$_B$), 3.88 (6H, d, *J* = 0.8 Hz, 2 × OC**H**$_3$), 3.85 (3H, d, *J* = 0.8 Hz, OC**H**$_3$), 3.19 (1H, br s, C=CC**H**), 2.60–2.39 (3H, m, O=CC**H**$_2$ and C=CC**H**$_A$H$_B$), 2.20 (1H, d, *J* =17.8 Hz, C=CCH$_A$**H**$_B$), 2.11 (1H, dt, *J* = 13.4, 9.8 Hz, HOCC**H**$_A$H$_B$), 1.92 (3H, s, C=CC**H**$_3$), 1.82 (1H, br s, O**H**), 1.78 (1H, ddd, *J* = 13.4, 8.0, 1.3 Hz, HOCCH$_A$**H**$_B$), 1.17 (3H, s, O=CCC**H**$_3$); ^{13}C NMR (101 MHz, $CDCl_3$) δ 218.6 (C), 153.1 (2 × C), 144.2 (C), 137.8 (C), 136.3 (C), 132.8 (C), 124.6 (CH), 115.8 (CH_2), 102.5 (2 × CH), 79.5 (C), 60.9 (CH_3), 56.2 (2 × CH_3), 52.9 (C), 50.6 (CH), 35.6 (CH_2), 32.8 (CH_2), 29.6 (CH_2), 22.9 (CH_3), 13.3 (CH_3); HRMS (ESI) Exact mass calcd for $[C_{22}H_{28}NaO_5]^+$ $[M+Na]^+$: 395.1829, found: 395.1841.

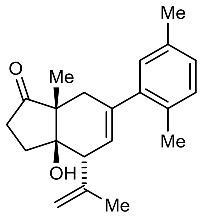

(±)-(3a*R*,4*R*,7a*R*)-6-(2,5-Dimethylphenyl)-3a-hydroxy-7a-methyl-4-(prop-1-en-2-yl)-2,3,3a,4,7,7a-hexahydro-1*H*-inden-1-one (2ga). General Procedure B was followed using enynone **1a** (102 mg, 0.499 mmol), 2,5-dimethylphenylboronic acid pinacol ester **S7** (151 mg, 0.650 mmol) and $[Rh(cod)Cl]_2$ (6.2 mg, 0.013 mmol, 2.5 mol%). Column chromatography (94% $CHCl_3$, 5% EtOAc, 1% Et_3N) of the crude material gave *alcohol* **2ga** (89.5 mg, 58%) as a yellow oil. R_f = 0.20 (94% $CHCl_3$, 5% EtOAc, 1% Et_3N); IR 3480 (O–H), 2925, 1734 (C=O), 1376, 1063 cm^{-1}; ^1H NMR (400 MHz, $CDCl_3$) δ 7.06 (1H, d, *J* = 7.8 Hz, Ar**H**), 6.99 (1H, dd, *J* = 7.8, 1.1 Hz, Ar**H**), 6.84 (1H, d, *J* = 1.1 Hz, Ar**H**), 5.46 (1H, t, *J* = 2.2 Hz, C=C**H**), 5.11 (1H, quin, *J* = 1.6 Hz, C=C**H**$_A$H$_B$), 4.96 (1H, br s, C=CH$_A$**H**$_B$), 3.19 (1H, ddd, *J* = 4.1, 2.2 1.6 Hz, C=CC**H**), 2.58–2.38 (2H, m, O=CC**H**$_2$), 2.33–2.15 (2H, m, C=CC**H**$_A$H$_B$ and HOCC**H**$_A$H$_B$), 2.31 (3H, s, ArC**H**$_3$), 2.25 (3H, s, ArC**H**$_3$), 2.00 (1H, d, *J* = 18.6 Hz, C=CCH$_A$**H**$_B$), 1.92 (3H, s, C=CC**H**$_3$), 1.84–1.77 (2H, m, HOCCH$_A$**H**$_B$ and O**H**), 1.23 (3H, s, O=CCC**H**$_3$); ^{13}C NMR (126 MHz, $CDCl_3$) δ 218.1 (C), 144.2 (C), 141.6 (C), 135.5 (C), 135.2 (C), 131.6 (C), 130.2 (CH), 128.9 (CH), 127.9 (CH), 126.1 (CH), 115.7 (CH_2), 79.3 (C), 53.0 (C), 50.2 (CH), 38.0 (CH_2), 32.7 (CH_2), 29.6 (CH_2), 22.8 (CH_3), 20.8 (CH_3), 19.3 (CH_3), 13.3 (CH_3); HRMS (ESI) Exact mass calcd for $[C_{21}H_{26}NaO_2]^+$ $[M+Na]^+$: 333.1825, found: 333.1828.

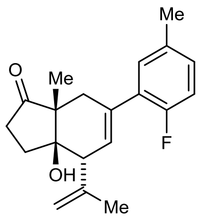

(±)-(3a*R*,4*R*,7a*R*)-6-(2-Fluoro-5-methylphenyl)-3a-hydroxy-7a-methyl-4-(prop-1-en-2-yl)-2,3,3a,4,7,7a-hexahydro-1*H*-inden-1-one (2ha). General Procedure B was followed using enynone **1a** (103 mg, 0.503 mmol) and 2-fluoro-5-methylphenylboronic acid pinacol ester **S8** (153 mg, 0.648 mmol). Column chromatography (94% $CHCl_3$, 5% EtOAc, 1% Et_3N) of the crude material gave *alcohol* **2ha** (80.2 mg, 51%) as a pale brown solid. R_f = 0.17 (94% $CHCl_3$, 5% EtOAc, 1% Et_3N); m.p. 87–88 °C (Et_2O); IR 3446 (O–H), 2974, 1726 (C=O), 1700, 1496, 1374, 1218, 1063 cm^{-1}; ^1H NMR (400 MHz, $CDCl_3$) δ 7.06–6.97 (2H, m, Ar**H**), 6.92 (1H, dd, J = 10.7, 8.3 Hz, Ar**H**), 5.78 (1H, t, J = 2.4 Hz, C=C**H**), 5.11 (1H, quin, J = 1.5 Hz, C=C**H$_A$**H$_B$), 4.95 (1H, s, C=CH$_A$**H$_B$**), 3.20–3.16 (1H, m, C=CC**H**), 2.58–2.37 (3H, m, O=CC**H$_2$** and C=CC**H$_A$**H$_B$), 2.31 (3H, s, ArC**H$_3$**), 2.22–2.11 (2H, m, HOCC**H$_A$**H$_B$ and C=CCH$_A$**H$_B$**), 1.92 (3H, s, C=CC**H$_3$**), 1.78 (1H, ddd, J = 13.4, 8.4, 1.8 Hz, HOCCH$_A$**H$_B$**), 1.78 (1H, s, O**H**), 1.19 (3H, s, O=CCC**H$_3$**); ^{13}C NMR (101 MHz, $CDCl_3$) δ 218.3 (C), 158.0 (d, J_F = 244.4 Hz, C), 144.0 (C), 133.5 (d, J_F = 2.9 Hz, C), 130.2 (C), 129.6 (d, J_F = 4.4 Hz, CH), 129.3 (d, J_F = 8.1 Hz, CH), 128.7 (d, J_F = 13.9 Hz, C), 128.2 (d, J_F = 2.9 Hz, CH), 115.7 (CH_2), 115.5 (d, J_F = 22.7 Hz, CH), 79.3 (C), 53.0 (C), 50.6 (CH), 36.5 (d, J_F = 2.9 Hz, CH_2), 32.7 (CH_2), 29.5 (CH_2), 22.9 (CH_3), 20.6 (CH_3), 13.0 (CH_3); ^{19}F NMR (376 MHz, $CDCl_3$) δ –120.6; HRMS (ESI) Exact mass calcd for $[C_{20}H_{23}FNaO_2]^+$ $[M+Na]^+$: 337.1574, found: 337.1573.

(±)-(3a*R*,4*R*,7a*R*)-6-(2,6-Dimethylpyridin-4-yl)-3a-hydroxy-7a-methyl-4-(prop-1-en-2-yl)-2,3,3a,4,7,7a-hexahydro-1*H*-inden-1-one (2ia)

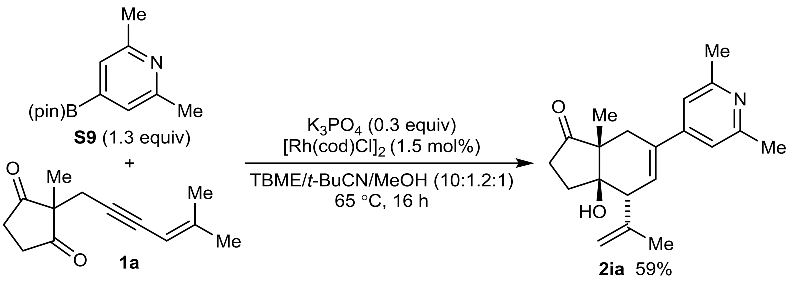

$[Rh(cod)Cl]_2$ (3.5 mg, 6.8 µmol) was added to an oven-dried microwave vial, and the vial was sealed with a septum and purged with nitrogen for 1 h. Degassed TBME (0.90 mL) and degassed *t*-BuCN (0.90 mL) were added and the mixture was stirred for 30 min at room temperature. Meanwhile, in a separate oven-dried microwave vial, enynone **1a** (92 mg, 0.450 mmol), 3,5-dimethylpyridineboronic acid pinacol ester **S9** (138 mg, 0.590 mmol), and K_3PO_4 (29.0 mg, 0.135 mmol) were added. The vial was sealed with a septum-lined microwave vial cap and purged with nitrogen for 1 h. Degassed TBME (6.6 mL), the solution of catalyst (see above), and degassed MeOH (0.75 mL) were added, and the mixture was stirred at 65 °C for 16 h. The reaction was cooled to room temperature, water (10 mL) and saturated aqueous ammonium chloride (10 mL)

were added, and the mixture was extracted with EtOAc (3 x 15 mL). The combined organic phases were washed with brine (30 mL), dried (Na_2SO_4), filtered, and concentrated in vacuo. The mixture was purified by column chromatography (twice, the first time using 70% $CHCl_3$, 29% EtOAc, 1% Et_3N; the second time using 50% EtOAc/petroleum ether) to give *alcohol* **2ia** (83.0 mg, 59%) as an off-white solid. R_f = 0.24 (50% EtOAc/petroleum ether); m.p. 202–204 °C (Et_2O); IR 3173 (O–H), 2963, 1732 (C=O), 1605, 1433, 1366, 1183 cm^{-1}; ^1H NMR (400 MHz, $CDCl_3$) δ 6.91 (2H, s, Ar**H**), 6.14 (1H, t, *J* = 2.5 Hz, C=C**H**), 5.13 (1H, t, *J* = 1.6 Hz, C=C**H$_A$**H$_B$), 4.94–4.91 (1H, m, C=CH$_A$**H$_B$**), 3.21-3.16 (1H, m, C=CC**H**), 2.54–2.36 (3H, m, O=CC**H$_2$** and C=CC**H$_A$**H$_B$), 2.52 (6H, s, 2 × ArC**H$_3$**), 2.20 (1H, dd, *J* = 18.0, 1.8 Hz, C=CCH$_A$**H$_B$**), 2.06 (1H, dt, *J* = 13.5, 9.8 Hz, HOCC**H$_A$**H$_B$), 1.93–1.90 (3H, m, C=CC**H$_3$**), 1.82 (1H, s, O**H**), 1.78 (1H, ddd, *J* = 13.5, 8.1, 2.8 Hz, HOCCH$_A$**H$_B$**), 1.15 (3H, s, O=CCC**H$_3$**); ^{13}C NMR (101 MHz, $CDCl_3$) δ 218.1 (C), 157.9 (2 × C), 148.0 (C), 143.7 (C), 131.3 (C), 127.7 (CH), 116.3 (2 × CH), 116.0 (CH_2), 79.3 (C), 52.7 (C), 50.5 (CH), 34.8 (CH_2), 32.7 (CH_2), 29.5 (CH_2), 24.5 (2 × CH_3), 23.0 (CH_3), 13.2 (CH_3); HRMS (ESI) Exact mass calcd for $[C_{20}H_{26}NO_2]^+$ $[M+H]^+$: 312.1958, found: 312.1961.

(±)-(3a*R*,4*S*,7a*R*)-6-(3,5-Dimethylphenyl)-4-ethenyl-3a-hydroxy-7a-methyl-2,3,3a,4,7,7a-hexahydro-1*H*-inden-1-one (9ba) and (±)-(3a*R*,4*R*,7a*R*)-6-(3,5-dimethylphenyl)-4-ethenyl-3a-hydroxy-7a-methyl-2,3,3a,4,7,7a-hexahydro-1*H*-inden-1-one (9bb).

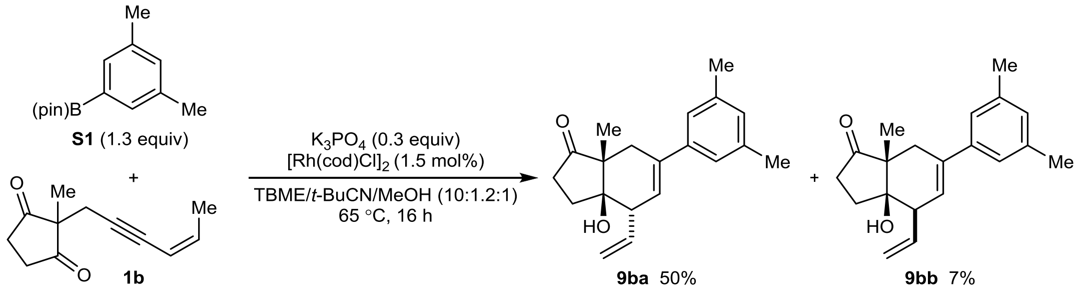

General Procedure B was followed using enynone **1b** (95.1 mg, 0.500 mmol), 3,5-dimethylphenylboronic acid pinacol ester **S1** (151 mg, 0.650 mmol) and $[Rh(cod)Cl]_2$ (6.2 mg, 0.013 mmol, 2.5 mol%). Column chromatography (94% $CHCl_3$, 5% EtOAc, 1% Et_3N) of the crude material gave *alcohol* **9bb** (10.1 mg, 7%) as a pale yellow oil, and *alcohol* **9ba** (73.7 mg, 50%) as a yellow oil which solidified into a pale yellow solid over time.

Data for **9ba**: R_f = 0.15 (94% $CHCl_3$, 5% EtOAc, 1% Et_3N); m.p. 89–90 °C (EtOAc); IR 3422 (O–H), 2927, 1725 (C=O), 1598, 1371, 1054 cm^{-1}; ^1H NMR (400 MHz, $CDCl_3$) δ 6.98 (2H, s, Ar**H**), 6.93 (1H, s, Ar**H**), 5.98–5.85 (2H, m, C**H**=CH$_2$ and CH$_2$C=C**H**), 5.39–5.35 (1H, m, C=C**H$_A$**H$_B$), 5.33 (1H, s, C=CH$_A$**H$_B$**), 3.16–3.08 (1H, m, C=CC**H**), 2.58–2.35 (3H, m, O=CC**H$_2$** and C=CC**H$_A$**H$_B$), 2.32 (6H, s, 2 × ArC**H$_3$**), 2.26 (1H, dd, *J* = 18.1, 0.8 Hz, C=CCH$_A$**H$_B$**), 2.06 (1H, dt, *J* = 13.7, 9.8 Hz, HOCC**H$_A$**H$_B$), 1.80 (1H, ddd, *J* = 13.7, 8.3, 2.1 Hz, HOCCH$_A$**H$_B$**), 1.67 (1H, s, O**H**),

1.16 (3H, s, O=CCC**H3**); ^{13}C NMR (101 MHz, $CDCl_3$) δ 218.4 (C), 140.2 (C), 138.0 (2 × C), 136.0 (CH), 133.4 (C), 129.2 (CH), 123.7 (CH), 123.1 (2 × CH), 119.2 (CH_2), 79.5 (C), 52.3 (C), 48.3 (CH), 36.0 (CH_2), 32.5 (CH_2), 28.8 (CH_2), 21.3 (2 × CH_3), 13.2 (CH_3); HRMS (ESI) Exact mass calcd for $[C_{20}H_{24}NaO_2]^+$ $[M+Na]^+$: 319.1669, found: 319.1666.

Data for **9bb**: R_f = 0.21 (94% $CHCl_3$, 5% EtOAc, 1% Et_3N); IR 3497 (O–H), 1925, 1742 (C=O), 1600, 1456, 1203, 1068 cm^{-1}; ^{1}H NMR (500 MHz, $CDCl_3$) δ 6.99 (2H, s, Ar**H**), 6.93 (1H, s, Ar**H**), 5.98 (1H, ddd, *J* = 4.6, 2.5, 0.6 Hz, C=C**H**), 5.92-5.84 (1H, m, C**H**=CH_2), 5.37-5.32 (2H, m, CH=C**H2**), 3.12 (1H, ddd, *J* = 9.0, 4.1, 3.0 Hz, C=CC**H**), 2.60-2.46 (3H, m, O=CC**H2** and C=CC**HA**HB), 2.35-2.28 (1H, m, C=CCHA**HB**), 2.32 (3H, s, ArC**H3**), 2.32 (3H, s, ArC**H3**), 2.19 (1H, dt, *J* = 13.3, 9.3 Hz, HOCC**HA**HB), 2.01 (1H, ddd, *J* = 13.2, 9.1, 3.4 Hz, HOCCHA**HB**), 1.17 (3H, s, O=CCC**H3**); ^{13}C NMR (126 MHz, $CDCl_3$) δ 218.4 (C), 140.5 (C), 137.9 (2 × C), 136.3 (CH), 133.7 (C), 129.2 (CH), 124.0 (CH), 123.3 (2 × CH), 120.5 (CH_2), 77.8 (C), 52.2 (C), 49.3 (CH), 35.3 (CH_2), 33.4 (CH_2), 32.5 (CH_2), 21.3 (2 × CH_3), 16.0 (CH_3); HRMS (ESI) Exact mass calcd for $[C_{20}H_{24}NaO_2]^+$ $[M+Na]^+$: 319.1669, found: 319.1668.

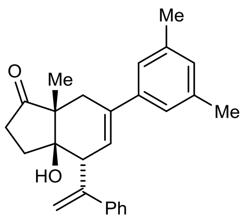

(±)-(3a*R*,4*R*,7a*R*)-6-(3,5-Dimethylphenyl)-3a-hydroxy-7a-methyl-4-(1-phenylethenyl)-2,3,3a,4,7,7a-hexahydro-1*H*-inden-1-one (9ca). General Procedure B was followed using enynone **1d** (134 mg, 0.503 mmol) and 3,5-dimethylphenylboronic acid pinacol ester **S1** (151 mg, 0.650 mmol). Column chromatography (94% $CHCl_3$, 5% EtOAc, 1% Et_3N) of the crude material gave *alcohol* **9ca** (40.3 mg, 22%) as a pale yellow solid. R_f = 0.41 (95% $CHCl_3$, 5% EtOAc); m.p. 138–139 °C (Et_2O); IR 3459 (O–H), 2925, 1743 (C=O), 1373, 1052 cm^{-1}; ^{1}H NMR (400 MHz, $CDCl_3$) δ 7.53–7.48 (2H, m, Ar**H**), 7.44–7.38 (2H, m, Ar**H**), 7.35 (1H, d, *J* = 7.2 Hz, Ar**H**), 7.04 (2H, s, Ar**H**), 6.96 (1H, s, Ar**H**), 6.15 (1H, t, *J* = 2.4 Hz, C=C**H**), 5.49 (1H, s, C=C**HA**HB), 5.20 (1H, s, C=CHA**HB**), 3.94 (1H, br s, C=CC**H**), 2.52–2.28 (3H, m, O=CC**H**2 and C=CC**HA**HB), 2.35 (6H, s, 2 × ArC**H3**), 2.24 (1H, dd, *J* = 17.9, 0.6 Hz, C=CCHA**HB**), 2.06 (1H, dt, *J* = 13.9, 9.7 Hz, HOCC**HA**HB), 1.69 (1H, ddd, *J* = 13.9, 9.1, 0.9 Hz, HOCCHA**HB**), 1.18 (3H, s, O=CCC**H3**), 1.04 (1H, s, O**H**); ^{13}C NMR (101 MHz, $CDCl_3$) δ 218.7 (C), 148.6 (C), 142.8 (C), 140.5 (C), 138.0 (2 × C), 133.6 (C), 129.2 (CH), 129.0 (2 × CH), 128.2 (CH), 126.9 (2 × CH), 125.0 (CH), 123.1 (2 × CH), 117.1 (CH_2), 80.4 (C), 53.4 (C), 48.2 (CH), 35.4 (CH_2), 32.5 (CH_2), 30.1 (CH_2), 21.4 (2 × CH_3), 13.1 (CH_3); HRMS (ESI) Exact mass calcd for $[C_{26}H_{29}O_2]^+$ $[M+H]^+$: 373.2162, found: 373.2163.

(±)-(3a*R*,4*S*,7a*R*)-6-(3,5-Dimethylphenyl)-3a-hydroxy-7a-methyl-4-(1-phenylethenyl)-2,3,3a,4,7,7a-hexahydro-1*H*-inden-1-one (9cb)

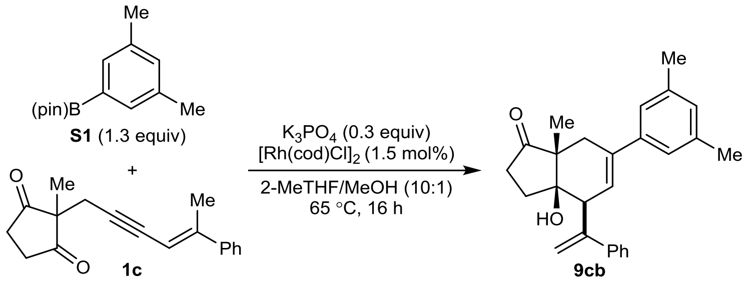

$[Rh(cod)Cl]_2$ (3.7 mg, 7.5 μmol) was added to an oven-dried microwave vial, and the vial was sealed with a septum and purged with nitrogen for 1 h. Degassed 2-MeTHF (1 mL) was added and the mixture was stirred for 30 min at room temperature. Meanwhile, in a separate oven-dried microwave vial, enynone **1c** (134 mg, 0.503 mmol), 3,5-dimethylphenylboronic acid pinacol ester **S1** (151 mg, 0.650 mmol), and K_3PO_4 (31.8 mg, 0.150 mmol) were added. The vial was sealed with a septum-lined microwave vial cap and purged under nitrogen for 1 h. Degassed 2-MeTHF (7.3 mL), the solution of catalyst (see above), and degassed MeOH (0.83 mL) were added, and the mixture was stirred at 65 °C for 16 h. The reaction was cooled to room temperature, water (10 mL) and saturated aqueous NH_4Cl solution (10 mL) were added, and the mixture was extracted with EtOAc (3 x 15 mL). The combined organic phases were washed with brine (30 mL), dried ($MgSO_4$), filtered, and concentrated *in vacuo*. The mixture was purified by column chromatography (25% EtOAc/petroleum ether) to give *alcohol* **9cb** (116 mg, 62%) as a pale yellow solid. R_f = 0.32 (25% EtOAc/petroleum ether); m.p. 122–123 °C (Et_2O); IR 3526 (O–H), 2989, 1733 (C=O), 1394, 1066 cm^{-1}; ^1H NMR (400 MHz, $CDCl_3$) δ 7.52–7.46 (2H, m, Ar**H**), 7.43–7.30 (3H, m, Ar**H**), 7.07 (2H, s, Ar**H**), 6.96 (1H, s, Ar**H**), 6.13–6.08 (1H, m, C=C**H**), 5.67 (1H, d, *J* = 0.6 Hz, C=C**H**$_A$H$_B$), 5.38 (1H, br s, C=CH$_A$**H**$_B$), 3.83 (1H, d, *J* = 1.4 Hz, C=CC**H**), 2.68 (1H, dt, *J* = 17.9, 2.6 Hz, C=CC**H**$_A$H$_B$), 2.47 (1H, dt, *J* = 19.2, 8.4 Hz, O=CC**H**$_A$H$_B$), 2.40–2.26 (2H, m, O=CCH$_A$**H**$_B$ and C=CCH$_A$**H**$_B$), 2.36 (6H, s, 2 × ArC**H**$_3$), 2.03 (2H, t, *J* = 7.7 Hz, HOCC**H**$_2$), 1.84 (1H, br s, O**H**), 1.20 (3H, s, O=CCC**H**$_3$); ^{13}C NMR (101 MHz, $CDCl_3$) δ 218.4 (C), 148.7 (C), 143.3 (C), 140.7 (C), 137.9 (2 × C), 134.6 (C), 129.1 (CH), 128.7 (2 × CH), 127.8 (CH), 126.3 (2 × CH), 125.2 (CH), 123.2 (2 × CH), 118.8 (CH_2), 78.0 (C), 52.8 (C), 48.1 (CH), 33.8 (CH_2), 33.3 (CH_2), 32.9 (CH_2), 21.3 (2 × CH_3), 17.0 (CH_3); HRMS (ESI) Exact mass calcd for $[C_{26}H_{29}O_2]^+$ $[M+H]^+$: 373.2162, found: 373.2155.

Slow diffusion of petroleum ether into a solution of **9cb** in EtOAc provided crystals that were suitable for X-ray diffraction:

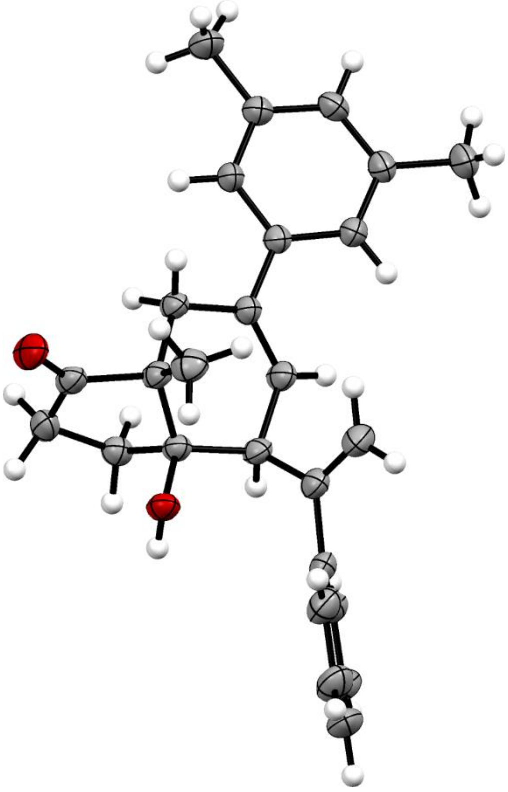

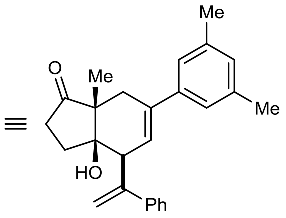

9cb
CCDC 1493831

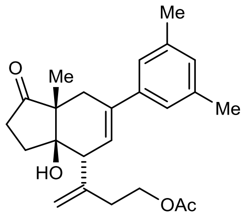

(±)-3-[(3a*R*,4*R*,7a*R*)-6-(3,5-Dimethylphenyl)-3a-hydroxy-7a-methyl-1-oxo-2,3,3a,4,7,7a-hexahydro-1*H*-inden-4-yl]but-3-en-1-yl acetate (9da). General Procedure B was followed using enynone **1d** (140 mg, 0.507 mmol) and 3,5-dimethylphenylboronic acid pinacol ester **S1** (151 mg, 0.650 mmol). Column chromatography (79% $CHCl_3$, 20% EtOAc, 1% Et_3N) of the crude material gave *alcohol* **9da** (102 mg, 52%) as a colorless oil. R_f = 0.28 (79% $CHCl_3$, 20% EtOAc, 1% Et_3N); IR 3508 (O–H), 2921, 1737 (C=O), 1244, 1037 cm^{-1}; ^{1}H NMR (400 MHz, $CDCl_3$) δ 6.98 (2H, s, Ar**H**), 6.93 (1H, s, Ar**H**), 5.91 (1H, t, *J* = 2.4 Hz, C=C**H**), 5.16 (1H, q, *J* = 1.3 Hz, C=C**H**$_\mathbf{A}$H$_B$), 5.05 (1H, s, C=CH$_A$**H**$_\mathbf{B}$), 4.31 (2H, t, *J* = 6.9 Hz, OC**H**$_\mathbf{2}$), 3.24 (1H, dt, *J* = 3.8, 2.0 Hz, C=CC**H**), 2.60 (2H, t, *J* = 6.9 Hz, OCH$_2$C**H**$_\mathbf{2}$), 2.57–2.38 (3H, m, O=CC**H**$_\mathbf{2}$ and C=CC**H**$_\mathbf{A}$H$_B$), 2.32 (6H, s, 2 × ArC**H**$_\mathbf{3}$), 2.24 (1H, dd, *J* = 18.0, 1.3 Hz, C=CCH$_A$**H**$_\mathbf{B}$), 2.09–1.99 (1H, m, HOCC**H**$_\mathbf{A}$H$_B$), 2.07 (3H, s, O=CC**H**$_\mathbf{3}$), 2.03 (1H, s, O**H**), 1.77 (1H, ddd, *J* = 13.7, 8.4, 1.8 Hz, HOCCH$_A$**H**$_\mathbf{B}$), 1.17 (3H, s, O=CCC**H**$_\mathbf{3}$); ^{13}C NMR (101 MHz, $CDCl_3$) δ 218.6 (C), 171.1 (C), 145.0 (C), 140.3 (C), 137.9 (2 × C), 133.3 (C), 129.1 (CH), 124.6 (CH), 123.1 (2 × CH), 115.8 (CH_2), 79.9 (C), 63.2 (CH_2), 53.4 (C), 49.6 (CH), 35.6 (CH_2), 35.3 (CH_2), 32.6 (CH_2), 29.1 (CH_2), 21.3 (2 × CH_3), 21.0 (CH_3), 13.2 (CH_3); HRMS (ESI) Exact mass calcd for $[C_{24}H_{30}NaO_4]^+$ $[M+Na]^+$: 405.2036, found: 405.2045.

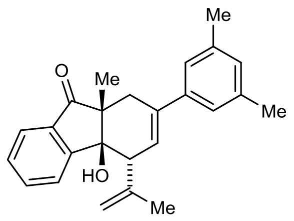

(±)-(4*R*,4a*S*,9a*R*)-2-(3,5-Dimethylphenyl)-4a-hydroxy-9a-methyl-4-(prop-1-en-2-yl)-4,4a,9,9a-tetrahydro-1*H*-fluoren-9-one (9ea). General Procedure B was followed using enynone **1e** (125 mg, 0.496 mmol) and 3,5-dimethylphenylboronic acid pinacol ester **S1** (151 mg, 0.650 mmol). Column chromatography (94% $CHCl_3$, 5% EtOAc, 1% Et_3N) of the crude material gave *alcohol* **9ea** (131 mg, 74%) as a pale yellow solid. R_f = 0.42 (94% $CHCl_3$, 5% EtOAc, 1% Et_3N); m.p. 152–153 °C (EtOAc); IR 3456 (O–H), 2973, 1701 (C=O), 1607, 1298, 1245, 1130 cm^{-1}; ^{1}H NMR (400 MHz, $CDCl_3$) δ 7.67–7.62 (2H, m, Ar**H**), 7.58 (1H, td, *J* = 7.5, 1.2 Hz, Ar**H**), 7.44–7.38 (1H, m, Ar**H**), 7.08 (2H, s, Ar**H**), 6.90 (1H, s, Ar**H**), 5.73–5.69 (1H, m, C=C**H**), 5.18–5.15 (1H, m, C=C$\mathbf{H_A}H_B$), 4.80 (1H, br s, CH, C=C$H_A\mathbf{H_B}$), 3.41 (1H, t, *J* = 2.8 Hz, C=CC**H**), 3.27 (1H, dd, *J* = 15.1, 1.0 Hz, O=CCC$\mathbf{H_A}H_B$), 2.44 (1H, s, O**H**), 2.43 (1H, dt, *J* = 15.1, 2.8 Hz, CC$H_A\mathbf{H_B}$), 2.32 (6H, s, 2 × ArC$\mathbf{H_3}$), 1.70 (3H, br s, C=CC$\mathbf{H_3}$), 1.26 (3H, s, O=CCC$\mathbf{H_3}$); ^{13}C NMR (101 MHz, $CDCl_3$) δ 207.1 (C), 153.9 (C), 144.2 (C), 142.2 (C), 140.1 (C), 137.8 (2 × C), 135.2 (C), 134.0 (CH), 129.2 (CH), 129.1 (CH), 126.7 (CH), 124.6 (CH), 123.6 (2 × CH), 122.7 (CH), 116.3 (CH_2), 83.3 (C), 57.1 (C), 55.6 (CH), 35.3 (CH_2), 23.7 (CH_3), 21.4 (2 × CH_3), 20.6 (CH_3); HRMS (ESI) Exact mass calcd for $[C_{25}H_{27}O_2]^+$ $[M+H]^+$: 359.2006, found: 359.2003.

Slow diffusion of petroleum ether into a solution of **9ea** in EtOAc provided crystals that were suitable for X-ray diffraction:

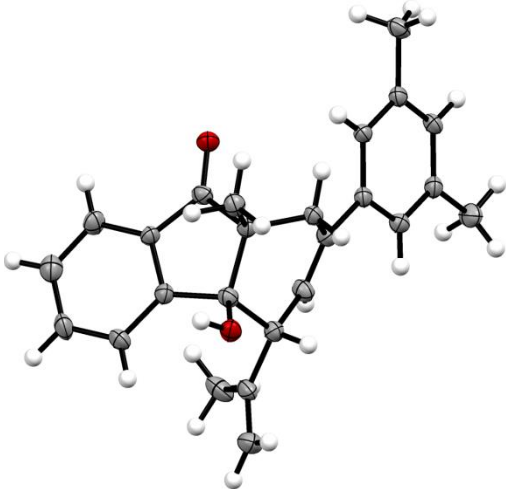

≡

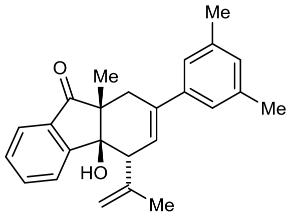

9ea
CCDC 1493832

(±)-(4a*R*,5*R*,8a*R*)-7-(3,5-Dimethylphenyl)-4a-hydroxy-8a-methyl-5-(prop-1-en-2-yl)-1,2,3,4,4a,5,8,8a-octahydronaphthalen-1-one (9fa)

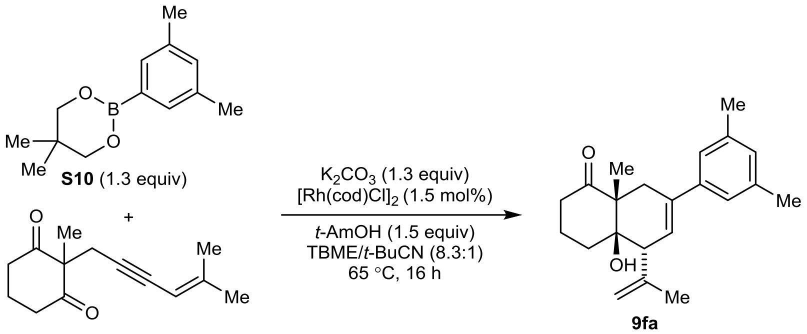

[Rh(cod)Cl]$_2$ (3.7 mg, 7.5 µmol, 1.5 mol%) was added to an oven-dried microwave vial, and the vial was sealed with a septum and purged with nitrogen for 1 h. Degassed TBME (1 mL) and degassed *t*-BuCN (1 mL) were added and the mixture was stirred for 30 min at room temperature. Meanwhile, in a separate oven-dried microwave vial, enynone **1f** (109 mg, 0.499 mmol), 3,5-dimethylphenylboronic acid neopentylglycol ester **S10** (142 mg, 0.651 mmol), and K_2CO_3 (89.8 mg, 0.650 mmol) were added. The vial was sealed with a septum-lined microwave vial cap and purged with nitrogen for 1 h. Degassed TBME (7.3 mL), the solution of catalyst (see above), and *t*-amyl alcohol (0.08 mL, 0.73 mmol) were added, and the mixture was stirred at 65 °C for 20 h. The reaction was cooled to room temperature, water (10 mL) and saturated aqueous NH_4Cl solution (10 mL) were added, and the mixture was extracted with EtOAc (3 x 15 mL. The combined organic phases were washed with brine (30 mL), dried ($MgSO_4$), filtered, and concentrated *in vacuo*. The mixture was purified by column chromatography (95% $CHCl_3$, 4% EtOAc, 1% Et_3N) to give *alcohol* **9fa** (108 mg, 67%) as a pale yellow solid. R_f = 0.37 (95% $CHCl_3$, 4% EtOAc, 1% Et_3N); m.p. 157–158 °C (Et_2O); IR 3408 (O–H), 2917, 1696 (C=O), 1376, 1243, 1084 cm^{-1}; ^{1}H NMR (400 MHz, $CDCl_3$) δ 7.00 (2H, s, Ar**H**), 6.94 (1H, s, Ar**H**), 5.99 (1H, t, *J* = 2.3 Hz, C=C**H**), 5.15–5.11 (1H, m, C=C**H**$_A$H$_B$), 4.93 (1H, s, C=CH$_A$**H**$_B$), 3.20–3.16 (1H, m, C=CC**H**), 3.04 (1H, dt, *J* = 17.4, 2.9 Hz, C=CC**H**$_A$H$_B$), 2.78 (1H, ddd, *J* = 15.1, 13.1, 7.2 Hz, O=CC**H**$_A$H$_B$), 2.41–2.29 (1H, m, O=CCH$_A$**H**$_B$), 2.33 (6H, s, 2 × ArC**H**$_3$), 2.29 (1H, dd, *J* = 17.4, 1.3 Hz, C=CCH$_A$**H**$_B$), 2.19–2.02 (1H, m, HOCC**H**$_A$H$_B$), 2.00–1.87 (2H, m, HOCCH$_A$**H**$_B$ and O=CCH$_2$C**H**$_A$H$_B$), 1.92 (3H, s, C=CC**H**$_3$), 1.67 (1H, s, O**H**), 1.63–1.53 (1H, m, O=CCH$_2$CH$_A$**H**$_B$), 1.25 (3H, s, O=CCC**H**$_3$); ^{13}C NMR (101 MHz, $CDCl_3$) δ 214.0 (C), 144.9 (C), 140.7 (C), 138.0 (2 × C), 133.4 (C), 129.1 (CH), 125.1 (CH), 123.1 (2 × CH), 115.9 (CH$_2$), 77.5 (C), 53.7 (C), 52.3 (CH), 38.6 (CH$_2$), 36.1 (CH$_2$), 28.3 (CH$_2$), 23.8 (CH$_3$), 21.4 (2 × CH$_3$), 20.1 (CH$_2$), 14.5 (CH$_3$); HRMS (ESI) Exact mass calcd for $[C_{22}H_{29}O_2]^+$ $[M+H]^+$: 325.2162, found: 325.2153.

Slow diffusion of petroleum ether into a solution of **9fa** in Et_2O provided crystals that were suitable for X-ray diffraction:

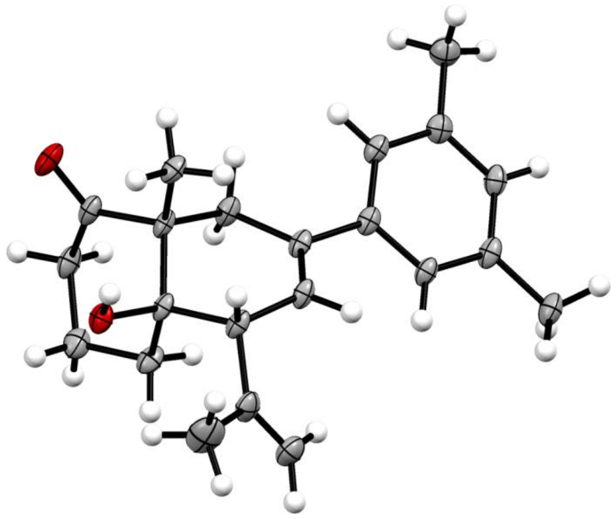

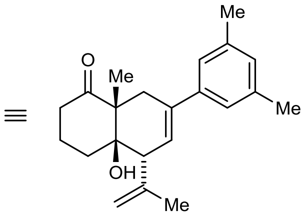

9fa
CCDC 1493833

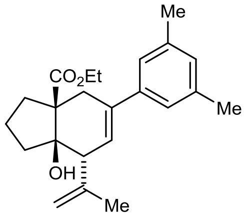

(±)-Ethyl (3a*S*,7*R*,7a*R*)-5-(3,5-dimethylphenyl)-7a-hydroxy-7-(prop-1-en-2-yl)-2,3,3a,4,7,7a-hexahydro-1*H*-indene-3a-carboxylate (11). A modification of General Procedure B was followed (a higher catalyst loading was used) using enynone **10** (124 mg, 0.498 mmol), 3,5-dimethylphenylboronic acid pinacol ester **S1** (151 mg, 0.650 mmol) and [Rh(cod)Cl]$_2$ (6.2 mg, 0.013 mmol, 2.5 mol%). Column chromatography (10% EtOAc/petroleum ether) of the crude material gave *alcohol* **11** (110 mg, 62%) as a 95:5 mixture of diastereomers as a pale yellow oil. R_f = 0.42 (10% EtOAc/petroleum ether); IR 3480 (O–H), 2977, 1702 (C=O), 1452, 1291, 1201 cm^{-1}; ^{1}H NMR (400 MHz, CDCl$_3$) δ 7.01 (2H, s, Ar**H**), 6.92 (1H, s, Ar**H**), 5.86 (1H, t, *J* = 2.8 Hz, C=C**H**), 4.95 (1H, quin, *J* = 1.5 Hz, C=C**H$_A$**H$_B$), 4.78 (1H, s, C=CH$_A$**H$_B$**), 4.19 (2H, q, *J* = 7.1 Hz, OC**H$_2$**), 3.99 (1H, d, *J* = 1.8 Hz, O**H**), 3.35 (1H, t, *J* = 3.0 Hz, C=CC**H**), 3.05 (1H, dd, *J* = 17.8, 0.8 Hz, C=CC**H$_A$**H$_B$), 2.46 (1H, dt, *J* = 17.8, 3.4 Hz, C=CCH$_A$**H$_B$**), 2.42–2.26 (1H, m, HOCCH$_2$CH$_2$C**H$_A$**H$_B$), 2.33 (6H, s, 2 × ArC**H$_3$**), 2.00 (3H, s, C=CC**H$_3$**), 1.99–1.90 (1H, m, HOCC**H$_A$**H$_B$) 1.89–1.73 (3H, m, HOCCH$_A$**H$_B$**C**H$_A$**H$_B$CH$_A$**H$_B$**), 1.68–1.58 (1H, m, HOCCH$_2$CH$_A$**H$_B$**CH$_2$), 1.26 (3H, t, *J* = 7.1 Hz, OCH$_2$C**H$_3$**); ^{13}C NMR (101 MHz, CDCl$_3$) δ 177.2 (C), 147.1 (C), 141.3 (C), 137.7 (2 × C), 133.5 (C), 128.7 (CH), 127.4 (CH), 123.2 (2 × CH), 113.0 (CH$_2$), 82.8 (C), 60.9 (CH$_2$), 55.3 (C), 51.3 (CH), 35.7 (CH$_2$), 34.9 (CH$_2$), 33.2 (CH$_2$), 24.6 (CH$_3$), 21.4 (2 × CH$_3$), 18.9 (CH$_2$), 14.1 (CH$_3$); HRMS (ESI) Exact mass calcd for $[C_{23}H_{30}NaO_3]^+$ $[M+Na]^+$: 377.2087, found: 377.2093.

(±)-(1*S*,2*R*)-4-(3,5-Dimethylphenyl)-1-methyl-2-(prop-1-en-2-yl)cyclohex-3-en-1-ol (13aa) and (±)-(1*S*,2*S*)-4-(3,5-dimethylphenyl)-1-methyl-2-(prop-1-en-2-yl)cyclohex-3-en-1-ol (13ab)

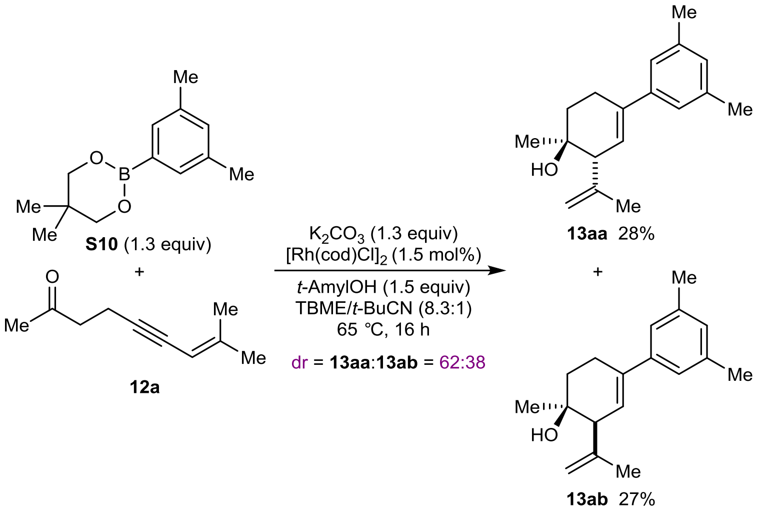

$[Rh(cod)Cl]_2$ (3.7 mg, 7.5 µmol) was added to an oven-dried microwave vial, and the vial was sealed with a septum and purged with nitrogen for 1 h. Degassed TBME (1 mL) and degassed *t*-BuCN (1 mL) were added and the mixture was stirred for 30 min at room temperature. Meanwhile, in a separate oven-dried microwave vial, enynone **12a** (75.1 mg, 0.500 mmol), 3,5-dimethylphenylboronic acid neopentylglycol ester **S10** (142 mg, 0.650 mmol), and K_2CO_3 (89.8 mg, 0.650 mmol) were added. The vial was sealed with a septum-lined microwave vial cap and purged with nitrogen for 1 h. Degassed TBME (7.3 mL), the solution of catalyst (see above), and *t*-amyl alcohol (0.08 mL, 0.73 mmol) were added, and the mixture was stirred at 65 °C for 16 h. The reaction was cooled to room temperature, water (10 mL) and saturated aqueous NH_4Cl solution (10 mL) were added, and the mixture was extracted with EtOAc (3 x 15 mL). The combined organic phases were washed with brine (30 mL), dried (Na_2SO_4), filtered, and concentrated in vacuo. ^{1}H NMR analysis indicated a 62:38 mixture of diastereomers **13aa** and **13ab**, respectively. The mixture was purified by column chromatography (twice, the first time using 10% EtOAc/*iso*-hexane; the second time using $CHCl_3$) to give *alcohol* **13ab** (35 mg, 27%) as a colorless oil followed by *alcohol* **13aa** (36 mg, 28%) as a colorless oil. The relative configurations of **13aa** and **13ab** could not be determined unambiguously and were assigned tentatively by analogy with the reaction producing **13ba** and **13bb**, on the assumption that the major products in each case possess the same relative configuration.

Data for **13aa**: R_f = 0.41 (10% EtOAc/petroleum ether); IR 3425 (O–H), 2920, 1599, 1447 1374, 1106, 846 cm^{-1}; ^{1}H NMR (400 MHz, $CDCl_3$) δ 7.05 (2H, s, Ar**H**), 6.92 (1H, s, Ar**H**), 5.92 (1H, dt, *J* = 3.6, 1.7 Hz, C=C**H**), 5.04–5.01 (1H, m, C=C**H**$_A$H$_B$), 4.88–4.85 (1H, m, C=CH$_A$**H**$_B$), 3.05–3.01 (1H, m, C=CC**H**), 2.68–2.46 (2H, m, C=CC**H**$_2$), 2.33 (6H, s, 2 × ArC**H**$_3$), 1.97–1.77 (6H, m, C=CCH$_2$C**H**$_2$, C=CC**H**$_3$ and O**H**), 1.20 (3H, s, C**H**$_3$COH); ^{13}C NMR (101 MHz, $CDCl_3$) δ 145.8

(C), 141.3 (C), 137.7 (2 × C), 135.8 (C), 128.7 (CH), 125.6 (CH), 123.1 (2 × CH), 115.0 (CH_2), 70.8 (C), 55.4 (CH), 34.9 (CH_2), 25.5 (CH_2), 24.9 (CH_3), 23.3 (CH_3), 21.4 (2 × CH_3); HRMS (ESI) Exact mass calcd for $[C_{18}H_{25}O]^+$ $[M+H]^+$: 257.1900, found: 257.1886.

Data for **13ab**: R_f = 0.67 (10% EtOAc/petroleum ether); IR 3466 (O–H), 2920, 1599, 1371, 1126, 846 cm^{-1}; ^1H NMR (400 MHz, $CDCl_3$) δ 7.06 (2H, s, Ar**H**), 6.92 (1H, s, Ar**H**), 5.86 (1H, dt, *J* = 3.4, 1.7 Hz, C=C**H**), 5.14–5.11 (1H, m, C=C**H**$_A$H$_B$), 4.89 (1H, app dt, *J* = 2.3, 0.8 Hz, C=CH$_A$**H**$_B$), 3.01–2.96 (1H, m, C=CC**H**), 2.74–2.63 (1H, m, C=CC**H**$_A$H$_B$), 2.50–2.40 (1H, m, C=CCH$_A$**H**$_B$), 2.34 (6H, s, 2 × ArC**H**$_3$), 2.01–1.90 (4H, m, C=CCH$_2$C**H**$_A$H$_B$ and C=CC**H**$_3$) 1.81–1.70 (2H, m, C=CCH$_2$CH$_A$**H**$_B$ and O**H**), 1.34 (3H, s, C**H**$_3$COH); ^{13}C NMR (101 MHz, $CDCl_3$) δ 146.5 (C), 141.5 (C), 137.7 (2 × C), 136.2 (C), 128.6 (CH), 125.9 (CH), 123.1 (2 × CH), 115.5 (CH_2), 69.3 (C), 53.9 (CH), 35.0 (CH_2), 28.5 (CH_3), 25.3 (CH_2), 24.6 (CH_3), 21.4 (2 × CH_3); HRMS (ESI) Exact mass calcd for $[C_{18}H_{24}NaO]^+$ $[M+Na]^+$: 279.1719, found: 279.1707.

(±)-(1*S*,2*R*)-4-(3,5-Dimethylphenyl)-1,6,6-trimethyl-2-(prop-1-en-2-yl)cyclohex-3-en-1-ol (13ba) and (±)-(1*S*,2*S*)-4-(3,5-dimethylphenyl)-1,6,6-trimethyl-2-(prop-1-en-2-yl)cyclohex-3-en-1-ol (13bb)

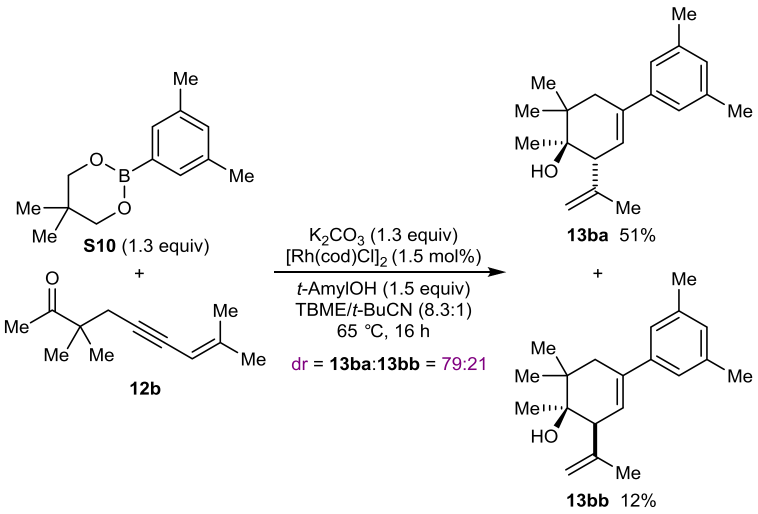

$[Rh(cod)Cl]_2$ (3.7 mg, 7.5 µmol) was added to an oven-dried microwave vial, and the vial was sealed with a septum and purged with nitrogen for 1 h. Degassed TBME (1 mL) and degassed *t*-BuCN (1 mL) were added and the mixture was stirred for 30 min at room temperature. Meanwhile, in a separate oven-dried microwave vial, enynone **12b** (89.1 mg, 0.500 mmol), 3,5-dimethylphenylboronic acid neopentylglycol ester **S10** (142 mg, 0.650 mmol), and K_2CO_3 (89.8 mg, 0.650 mmol) were added. The vial was sealed with a septum-lined microwave vial cap and purged with nitrogen for 1 h. Degassed TBME (7.3 mL), the solution of catalyst (see above), and *t*-amyl alcohol (0.08 mL, 0.73 mmol) were added, and the mixture was stirred at 65 °C for 16 h. The reaction was cooled to room temperature, water (10 mL) and saturated aqueous NH_4Cl solution (10

mL) were added, and the mixture was extracted with EtOAc (3 x 15 mL). The combined organic phases were washed with brine (30 mL), dried (Na_2SO_4), filtered, and concentrated *in vacuo*. ^{1}H NMR analysis indicated a 79:21 mixture of diastereomers **13ba** and **13bb**, respectively. The mixture was purified by column chromatography (10% EtOAc/*iso*-hexane) to give *alcohol* **13ba** (72 mg, 51%) as a pale yellow oil followed by *alcohol* **13bb** in *ca.* 85% purity as determined by ^{1}H NMR analysis (17 mg, *ca.* 12%, after a second column using 2% Et_2O/*iso*-hexane) as a pale yellow oil.

Data for **13ba**: R_f = 0.31 (10% EtOAc/petroleum ether); IR 3580 (O–H), 2962, 1599, 1372, 1092, 844 cm^{-1}; ^{1}H NMR (400 MHz, $CDCl_3$) δ 7.06 (2H, s, Ar**H**), 6.94 (1H, s, Ar**H**), 5.97 (1H, t, *J* = 2.4 Hz, C=C**H**), 5.12–5.08 (1H, m, C=C**H**$_{\mathbf{A}}$H$_B$), 4.89 (1H, dt, *J* = 1.9, 0.9 Hz, C=C**H**$_{\mathbf{A}}$H$_B$), 3.17 (1H, dt, *J* = 4.1, 1.9 Hz, C=CC**H**), 2.54–2.45 (1H, m, C=CC**H**$_{\mathbf{A}}$H$_B$), 2.36 (6H, s, 2 × ArC**H**$_{\mathbf{3}}$), 2.24 (1H, dd, *J* = 17.5, 1.8 Hz, C=CCH$_A$**H**$_{\mathbf{B}}$), 1.98–1.93 (3H, m, C=CC**H**$_{\mathbf{3}}$), 1.55 (1H, s, O**H**), 1.14 (3H, s, C(C**H**$_{\mathbf{3}}$)$_2$), 1.12 (3H, s, C(C**H**$_{\mathbf{3}}$)$_2$), 1.10 (3H, s, C**H**$_{\mathbf{3}}$COH); ^{13}C NMR (101 MHz, $CDCl_3$) δ 146.6 (C), 141.6 (C), 137.7 (2 × C), 135.3 (C), 128.6 (CH), 126.2 (CH), 123.0 (2 × CH), 114.9 (CH_2), 74.8 (C), 52.1 (CH), 41.9 (CH_2), 37.9 (C), 24.3 (CH_3), 24.1 (CH_3), 22.3 (CH_3), 21.4 (2 × CH_3), 19.5 (CH_3); HRMS (ESI) Exact mass calcd for $[C_{20}H_{28}NaO]^+$ $[M+Na]^+$: 307.2032, found: 307.2031.

Data for **13bb**: R_f = 0.35 (10% EtOAc/petroleum ether); IR 3559 (O–H), 2961, 1598, 1445, 1373, 1071, 844 cm^{-1}; ^{1}H NMR (400 MHz, $CDCl_3$) δ 7.05 (2H, s, Ar**H**), 6.90 (1H, s, Ar**H**), 5.78 (1H, t, *J* = 2.4 Hz, C=C**H**), 5.12-5.09 (1H, m, C=C**H**$_{\mathbf{A}}$H$_B$), 4.89–4.87 (1H, m, C=C**H**$_{\mathbf{A}}$H$_B$), 3.04 (1H, dt, *J* = 4.1, 1.9 Hz, C=CC**H**), 2.54–2.45 (1H, dt, *J* = 17.3, 3.4 Hz, C=CC**H**$_{\mathbf{A}}$H$_B$), 2.33 (6H, s, 2 × ArC**H**$_{\mathbf{3}}$), 2.00 (1H, dd, *J* = 17.3, 1.7 Hz, C=CCH$_A$**H**$_{\mathbf{B}}$), 1.95–1.92 (3H, m, C=CC**H**$_{\mathbf{3}}$), 1.66 (1H, s, O**H**), 1.20 (3H, s, C**H**$_{\mathbf{3}}$COH), 1.11 (3H, s, C(C**H**$_{\mathbf{3}}$)$_2$), 1.00 (3H, s, C(C**H**$_{\mathbf{3}}$)$_2$); ^{13}C NMR (101 MHz, $CDCl_3$) δ 147.0 (C), 141.7 (C), 137.7 (2 × C), 136.1 (C), 128.6 (CH), 125.3 (CH), 123.0 (2 × CH), 115.7 (CH_2), 73.5 (C), 51.6 (CH), 39.6 (CH_2), 37.7 (C), 24.6 (CH_3), 24.3 (2 × CH_3), 21.4 (2 × CH_3), 20.9 (CH_3); HRMS (ESI) Exact mass calcd for $[C_{20}H_{29}O]^+$ $[M+H]^+$: 285.2213, found: 285.2204.

Possible Stereochemical Models for the Cyclization of Fully Acyclic Substrates

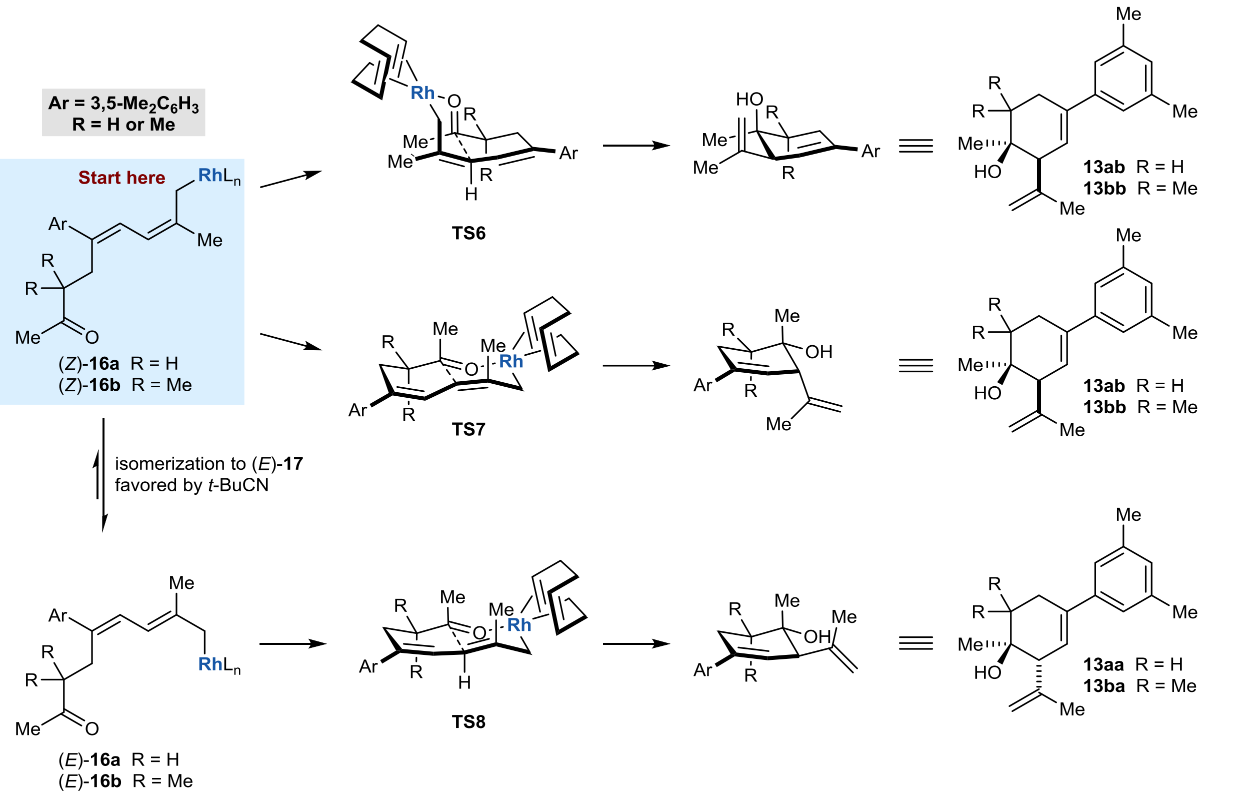

For the fully acyclic substrates, we suggest chairlike transition states similar to those invoked for the substrates containing cyclic ketones (see Scheme 5 of the paper) can be used to explain the diastereochemical outcomes. The formation of diastereomers **13ab** and **13bb** is likely to proceed from (*Z*)-**16a** or (*Z*)-**16b**, respectively, formed initially after 1,4-migration, through **TS6** and/or **TS7**. However, a competitive pathway is isomerization of (*Z*)-**16a** and (*Z*)-**16b** to give (*E*)-**16a** and (*E*)-**16b**, respectively. We assume that this isomerization is promoted by *t*-BuCN, which slows down the rate of allylation. Allylation through **TS8** would then give diastereomers **13aa** and **13ba**.

NB: The same reactions conducted in 2-MeTHF in place of TBME/*t*-BuCN gave **13ab** and **13bb** as the major products, but were lower yielding.

(±)-(3a*R*,5*E*,9b*R*)-9b-Hydroxy-3a-methyl-5-(3-methylbut-2-en-1-ylidene)-1*H*,2*H*,3*H*,3a*H*,4*H*,5*H*,9b*H*-cyclopenta[a]naphthalen-3-one (15) and (3a*R*,4*R*,7a*R*)-3a-hydroxy-7a-methyl-6-phenyl-4-(prop-1-en-2-yl)-2,3,3a,4,7,7a-hexahydro-1*H*-inden-1-one (2ja)

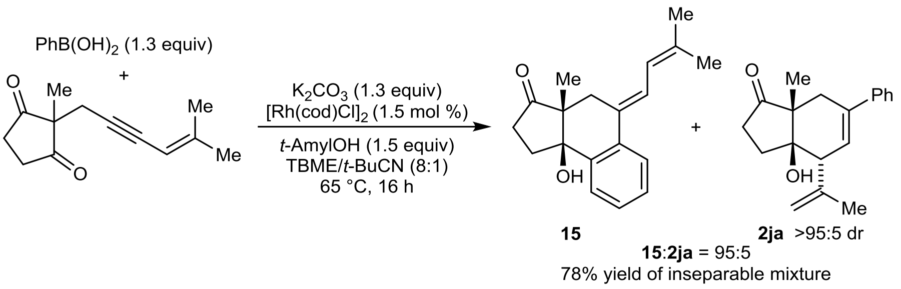

[Rh(cod)Cl]$_2$ (3.7 mg, 7.5 μmol) was added to an oven-dried microwave vial, and the vial was sealed with a septum and purged with nitrogen for 1 h. Degassed TBME (1 mL) and *t*-BuCN (1 mL) were added and the mixture was stirred for 30 min at room temperature. Meanwhile, in a separate oven-dried microwave vial, enynone **1a** (101 mg, 0.494 mmol), phenylboronic acid (79.3 mg, 0.650 mmol), and K_2CO_3 (89.8 mg, 0.650 mmol) were added. The vial was sealed with a septum-lined microwave vial cap and purged with nitrogen for 1 h. Degassed TBME (7 mL), the solution of catalyst (see above), and *t*-amyl alcohol (0.08 mL, 0.73 mmol) were added, and the mixture was stirred at 65 °C for 16 h. The reaction was cooled to room temperature, water (10 mL) and saturated aqueous NH_4Cl solution (10 mL) were added, and the mixture was extracted with EtOAc (3 x 15 mL). The combined organic phases were washed with brine (30 mL), dried ($MgSO_4$), filtered, and concentrated *in vacuo*. Column chromatography (95% $CHCl_3$, 4% EtOAc, 1% Et_3N) of the crude material gave a 95:5 mixture of *alcohol* **15** and *alcohol* **2ja** and (110 mg, 78%) as a pale yellow solid.

Data for **15**: R_f = 0.17 (94% $CHCl_3$, 5% EtOAc, 1% Et_3N); ^{1}H NMR (400 MHz, $CDCl_3$) δ 7.70-7.65 (1H, m, Ar**H**), 7.64-7.60 (1H, m, Ar**H**), 7.37-7.26 (2H, m, Ar**H**), 6.96 (1H, dd, *J* = 11.4, 1.3 Hz, ArC=C**H**), 6.18 (1H, ddt, *J* = 11.3, 2.7, 1.4 Hz, C**H**=C(CH_3)$_2$), 2.65 (1H, d, *J* = 14.3 Hz, C=CC**H$_A$**H$_B$), 2.63 (1H, ddd, *J* = 19.0, 10.5, 9.2 Hz, O=CC**H$_A$**H$_B$), 2.51-2.35 (3H, m, O=CCH$_A$**H$_B$**, C=CCH$_A$**H$_B$**, and HOCC**H$_A$**H$_B$), 2.27 (1H, ddd, *J* = 13.7, 10.5, 9.2 Hz, HOCCH$_A$**H$_B$**), 1.98 (1H, br s, O**H**), 1.90 (3H, s, C=CC**H$_3$**), 1.90 (3H, s, C=CC**H$_3$**), 1.05 (3H, s, O=CCC**H$_3$**) ; ^{13}C NMR (126 MHz, $CDCl_3$) δ 220.7 (C), 139.7 (C), 138.6 (C), 134.9 (C), 127.88 (CH), 127.87 (CH), 127.7 (C), 126.5 (CH), 123.3 (CH), 123.3 (CH), 120.9 (CH), 80.1 (C), 53.8 (C), 35.9 (CH_2), 35.2 (CH_2), 33.3 (CH_2), 26.7 (CH_3), 18.5 (CH_3), 14.1 (CH_3); HRMS (ESI) Exact mass calcd for $[C_{19}H_{22}NaO_2]^+$ $[M+Na]^+$: 305.1512, found: 305.1516.

Diagnostic signals for **2ja**: ^{1}H NMR (400 MHz, $CDCl_3$) δ 5.99 (1H, t, *J* = 2.4 Hz, C=C**H**), 5.12 (1H, quin, *J* = 1.5 Hz, C=C**H$_A$**H$_B$), 4.95 (1H, br s, C=CH$_A$**H$_B$**), 3.20 (1H, dt, *J* = 4.0, 2.0 Hz,

C=CC**H**), 1.16 (3H, s, O=CCC**H**$_3$).

(±)-(3a*R*,4*S*,7a*R*)-3a-Hydroxy-7a-methyl-6-phenyl-4-(prop-1-en-2-yl)-2,3,3a,4,7,7a-hexahydro-1*H*-inden-1-one (2jb)

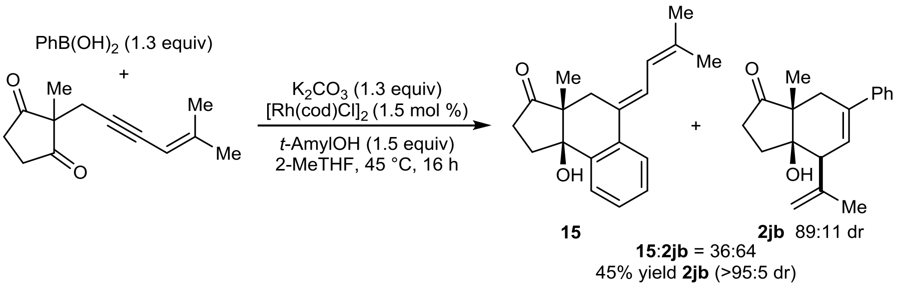

$[Rh(cod)Cl]_2$ (3.7 mg, 7.5 µmol) was added to an oven-dried microwave vial, and the vial was sealed with a septum and purged with nitrogen for 1 h. Degassed 2-MeTHF (2 mL) was added and the mixture was stirred for 30 min at room temperature. Meanwhile, in a separate oven-dried microwave vial, enynone **1a** (103 mg, 0.504 mmol), phenylboronic acid (79.3 mg, 0.650 mmol), and K_2CO_3 (89.8 mg, 0.650 mmol) were added. The vial was sealed with a septum-lined microwave vial cap and purged with nitrogen for 1 h. Degassed MeTHF (6 ml), the solution of catalyst (see above), and *t*-amyl alcohol (0.08 mL, 0.73 mmol) were added, and the mixture was stirred at 45 °C for 20 h. The reaction was cooled to room temperature, water (10 mL) and saturated aqueous NH_4Cl solution (10 mL) were added, and the mixture was extracted with EtOAc (3 x 15 mL. The combined organic phases were washed with brine (30 mL), dried ($MgSO_4$), filtered, and concentrated *in vacuo*. The mixture was purified by column chromatography (96% $CHCl_3$, 3% EtOAc, 1% Et_3N) to give *alcohol* **2jb** (64.5 mg, 45%) as a colorless oil. R_f = 0.20 (96% $CHCl_3$, 3% EtOAc, 1% Et_3N); IR 3504 (O–H), 2975, 1738 (C=O), 1446, 1198, 1069 cm^{-1}; ^{1}H NMR (400 MHz, $CDCl_3$) δ 7.41–7.31 (4H, m, Ar**H**), 7.30–7.25 (1H, m, Ar**H**), 5.95 (1H, ddq, *J* = 3.2, 2.8, 0.6 Hz, C=C**H**), 5.20 (1H, quin, *J* = 1.5 Hz, C=C**H**$_A$H$_B$), 5.00 (1H, br s, C=CH$_A$**H**$_B$), 3.14 (1H, ddd, *J* = 3.2, 2.8, 1.5 Hz, C=CC**H**), 2.61 (1H, dt, *J* = 17.6, 2.8 Hz, C=CC**H**$_A$H$_B$), 2.57 (1H, ddd, *J* = 19.2, 9.7, 7.7 Hz, O=CC**H**$_A$H$_B$), 2.43 (1H, ddd, *J* = 19.2, 9.0, 4.8 Hz, O=CCH$_A$**H**$_B$), 2.32 (1H, dd, *J* = 17.6, 0.6 Hz, C=CCH$_A$**H**$_B$), 2.23 (1H, ddd, *J* = 13.2, 9.0, 7.7 Hz, HOCC**H**$_A$H$_B$), 2.13 (1H, ddd, *J* = 13.2, 9.7, 4.8 Hz, HOCCH$_A$**H**$_B$), 1.96 (3H, dd, *J* = 1.5, 0.6 Hz, C=CC**H**$_3$), 1.86 (1H, br s, O**H**), 1.18 (3H, s, O=CCC**H**$_3$); ^{13}C NMR (126 MHz, $CDCl_3$) δ 218.6 (C), 145.2 (C), 140.8 (C), 134.2 (C), 128.4 (2 × CH), 127.4 (CH), 125.5 (CH), 125.3 (2 × CH), 117.0 (CH_2), 77.8 (C), 52.7 (C), 51.4 (CH), 33.9 (CH_2), 33.6 (CH_2), 33.4 (CH_2), 24.5 (CH_3), 16.8 (CH_3); HRMS (ESI) Exact mass calcd for $[C_{19}H_{22}NaO_2]^+$ $[M+Na]^+$: 305.1512, found: 305.1511.

The relative configuration of **2jb** could not be determined unambiguously by NOESY NMR spectroscopy (see page 99), but was assigned tentatively by analogy with **9cb** (NOESY NMR

spectrum on page 82), the relative configuration of which was determined by X-ray crystallography (see page 21).

4. Investigation of Deuterium Transfer

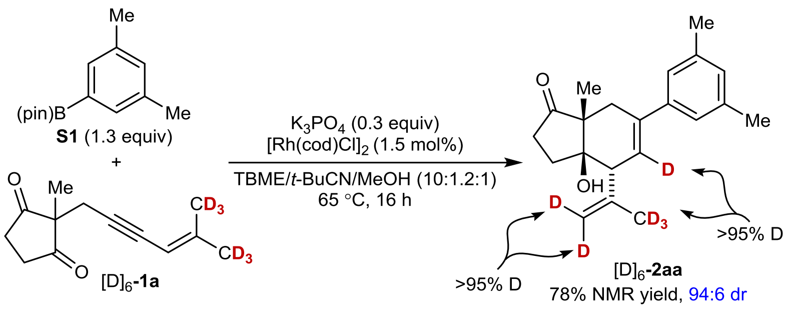

According to General Procedure B, enynone [D]$_6$-**1a** (10.1 mg, 0.0480mmol) was reacted with 3,5-dimethylphenylboronic acid pinacol ester **S1** (15.1 mg, 0.0650 mmol), K_3PO_4 (3.2 mg, 0.0151 mmol) and $[Rh(cod)Cl]_2$ (0.4 mg, 0.8 µmol) in TBME (0.80 mL), t-BuCN (0.10 mL) and MeOH (0.08 mL). After heating at 65 °C for 16 h, the mixture was cooled, 1,3,5-trimethoxybenzene (6.1 mg, 0.036 mmol) was added as an internal standard, and the mixture was partitioned between EtOAc (1 mL) and water (1 mL). The organic phase was concentrated *in vacuo* and analyzed by ^{1}H NMR spectroscopy ([D$_6$]-**2aa** was formed in 78% NMR yield in a d.r. of 94:6). Column chromatography (95% $CHCl_3$, 4% EtOAc, 1% Et_3N) gave a sample of *alcohol* [D$_6$]-**2aa** for characterization.

Expansion of crude ^{1}H NMR spectrum:

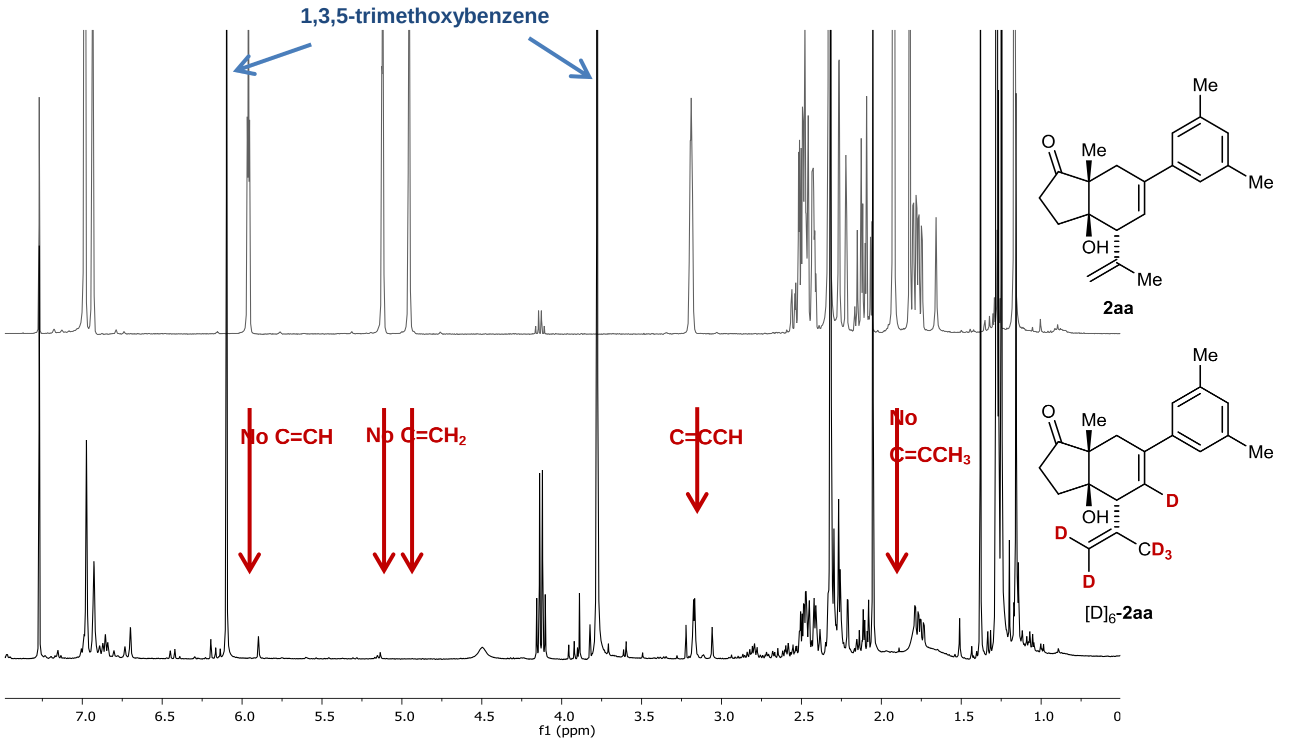

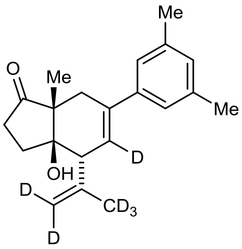

(±)-(3a*R*,4*R*,7a*R*)-6-(3,5-Dimethylphenyl)-3a-hydroxy-7a-methyl-4-[($^2H_5$)prop-1-en-2-yl]-2,3,3a,4,7,7a-hexahydro(5-^{2}H)-1*H*-inden-1-one ([D_6]-2aa). R_f = 0.23 (95% $CHCl_3$, 4% EtOAc, 1% Et_3N); IR 3452 (O–H), 2917, 1737 (C=O), 1599, 1374, 1207, 1065 cm^{-1}; ^{1}H NMR (400 MHz, $CDCl_3$) δ 6.98 (2H, s, Ar**H**), 6.93 (1H, s, Ar**H**), 3.17 (1H, dd, *J* = 4.1, 1.7 Hz, C=CC**H**), 2.57-2.40 (3H, m, O=CC**H₂** and C=CC**H**$_{\mathbf{A}}$H$_B$), 2.32 (3H, s, ArC**H**$_{\mathbf{3}}$), 2.32 (3H, s, ArC**H**$_{\mathbf{3}}$), 2.24 (1H, dd, *J* = 17.9, 1.7 Hz, C=CCH$_A$**H**$_{\mathbf{B}}$), 2.10 (1H, dt, *J* = 13.6, 9.8 Hz, HOC**H**$_{\mathbf{A}}$H$_B$), 1.76 (1H, ddd, *J* = 13.6, 8.2, 2.3 Hz, HOC**H**$_{\mathbf{A}}$**H**$_{\mathbf{B}}$), 1.72 (1H, br s, O**H**), 1.16 (3H, s, O=CCC**H**$_{\mathbf{3}}$); ^{13}C NMR (101 MHz, $CDCl_3$) δ 218.6 (C), 144.0 (C), 140.4 (C), 138.0 (2 × C), 133.0 (C), 129.1 (CH), 123.1 (2 × CH), 79.5 (C), 52.9 (CH), 50.4 (C), 35.6 (CH_2), 32.8 (CH_2), 29.5 (CH_2), 21.4 (2 × CH_3), 13.2 (CH_3); ^{2}H NMR (77 MHz, $CHCl_3$) δ 6.01 (1D, br s, C=C**D**), 5.16 (1D, br s, C=C**D**$_{\mathbf{A}}$D$_B$), 4.99 (1D, br s, C=CD$_A$**D**$_{\mathbf{B}}$), 1.89 (3D, br s, C**D**$_3$); HRMS (ESI) Exact mass calcd for $[C_{21}H_{20}D_6NaO_2]^+$ $[M+Na]^+$: 339.2202, found: 339.2200.

5. Enantioselective Reactions

Result Using a Chiral Diene Ligand

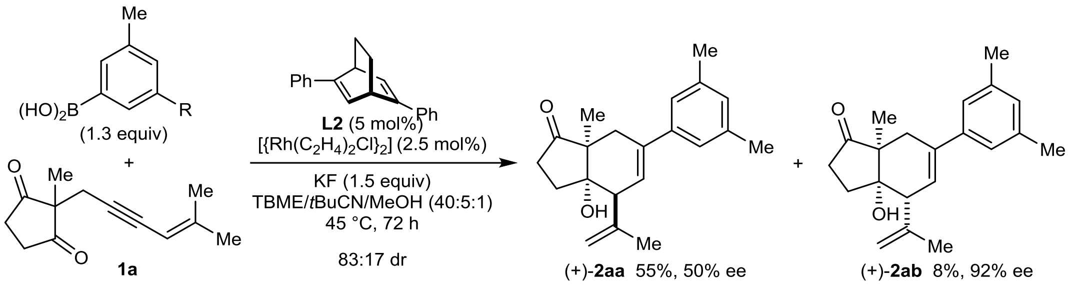

Reaction of **1a** with 3,5-dimethylphenylboronic acid using diene **L2**[12] gave diastereomeric products (+)-**2aa** and (+)-**2ab** in 55% and 8% yield, respectively. Although (+)-**2ab** was obtained in 92% ee, the major product (+)-**2aa** was formed in only 50% ee.

(+)-(3a*S*,4*S*,7a*S*)-6-(3,5-Dimethylphenyl)-3a-hydroxy-7a-methyl-4-(prop-1-en-2-yl)2,3,3a,4,7,7a-hexahydro-1*H*-inden-1-one (2aa) and (+)-(3a*S*,4*R*,7a*S*)-6-(3,5-dimethylphenyl)-3ahydroxy-7a-methyl-4-(prop-1-en-2-yl)-2,3,3a,4,7,7a-hexahydro-1*H*-inden-1-one (2ab)

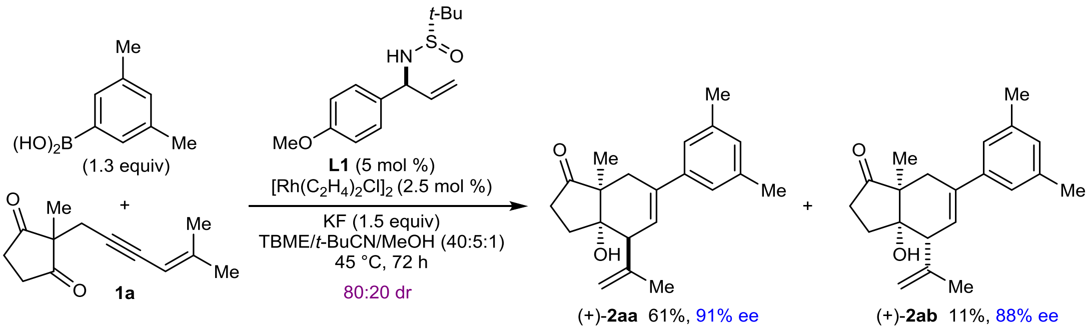

$[Rh(C_2H_4)Cl]_2$ (2.5 mg, 6.4 µmol) and **L1**[13] (3.4 mg, 13 µmol) were added to an oven-dried microwave vial, and the vial was sealed with a septum and purged with nitrogen for 1 h. Degassed TBME (1 mL) and degassed *t*-BuCN (0.5 mL) were added and the mixture was stirred for 30 min at room temperature. Meanwhile, in a separate oven-dried microwave vial, enynone **1a** (51.0 mg, 0.250 mmol), 3,5-dimethylphenylboronic acid (56.3 mg, 0.375 mmol), and KF (21.8 mg, 0.375 mmol) were added. The vial was sealed with a septum-lined microwave vial cap and purged with nitrogen for 1 h. Degassed TBME (3.2 mL), the solution of catalyst (see above), and MeOH (0.10 mL) were added, and the mixture was stirred at 45 °C for 72 h. The reaction was cooled to room temperature, water (10 mL) and saturated aqueous NH_4Cl solution (10 mL) were added, and

12. (a) N. Tokunaga, Y. Otomaru, K. Okamoto, K. Ueyama, R. Shintani, T. Hayashi, *J. Am. Chem. Soc.* **2004**, *126*, 13584–13585. (b) Y. Otomaru, K. Okamoto, R. Shintani, T. Hayashi, *J. Org. Chem.* **2005**, *70*, 2503–2508.
13. Prepared according to: X. Feng, Y. Wang, B. Wei, J. Yang, H. Du, *Org. Lett.* **2011**, *13*, 3300–3303.

the mixture was extracted with EtOAc (3 × 10 mL. The combined organic phases were washed with brine (15 mL), dried (Na_2SO_4), filtered, and concentrated *in vacuo*. ^{1}H NMR analysis indicated a 80:20 mixture of diastereomers (+)–**2aa** and (+)–**2ab**, respectively. The mixture was purified by column chromatography (twice, the first time using 94% $CHCl_3$, 5% EtOAc, 1% Et_3N; the second time using 20% EtOAc/petroleum ether) to give alcohol (+)–**2aa** (47 mg, 61%) as a white solid and alcohol (+)–**2ab** (8 mg, 11%) as a colorless oil. The spectroscopic data for these compounds matched those of the racemic compounds reported above. In addition:

Data for (+)–**2aa**: $[\alpha]_D^{21}$ +34.0 (*c* 0.47, $CHCl_3$). Enantiomeric excess was determined by HPLC using a Chiralcel IC column (95:5 *iso*-hexane:*i*-PrOH, 1.5 mL/min, 210 nm, 25 °C); t_r (major) = 14.6 min, t_r (minor) = 17.2 min, 91% ee.

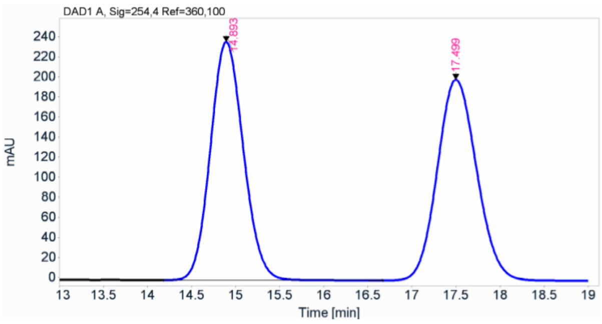

Signal: DAD1 A, Sig=254,4 Ref=360,100

RT [min]	Type	Width [min]	Area	Height	Area%
14.893	BB	0.4058	6186.391	237.5838	49.87
17.499	BBA	0.4839	6219.494	200.4773	50.13

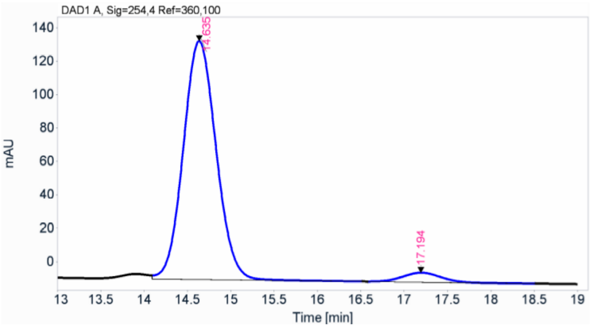

Signal: DAD1 A, Sig=254,4 Ref=360,100

RT [min]	Type	Width [min]	Area	Height	Area%
14.635	VB	0.4117	3764.122	142.7327	95.52
17.194	BBA	0.4735	176.462	5.7272	4.48

Slow diffusion of petroleum ether into a solution of (+)–**2aa** in EtOAc provided crystals that were suitable for X-ray diffraction:

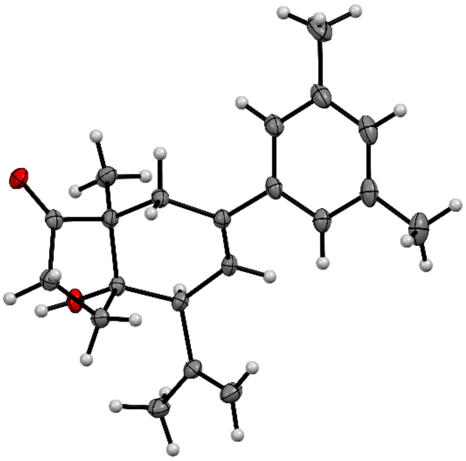

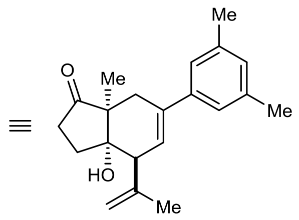

(+)–**2aa**
CCDC 1535509
Flack parameter = –0.05(11)

Data for (+)–**2ab**: $[\alpha]_D^{21}$ +63.2 (*c* 0.76, $CHCl_3$). Enantiomeric excess was determined by HPLC using a Chiralcel IC column (95:5 *iso*-hexane:*i*-PrOH, 1.5 mL/min, 254 nm, 25 °C); t_r (major) = 9.2

min, t_r (minor) = 12.6 min, 88% ee.

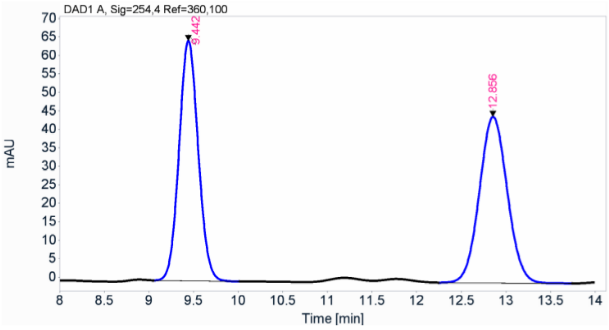

Signal:	DAD1 A, Sig=254,4 Ref=360,100				
RT [min]	Type	Width [min]	Area	Height	Area%
9.442	BB	0.2322	970.945	65.0271	49.77
12.856	BB	0.3401	980.072	44.9986	50.23

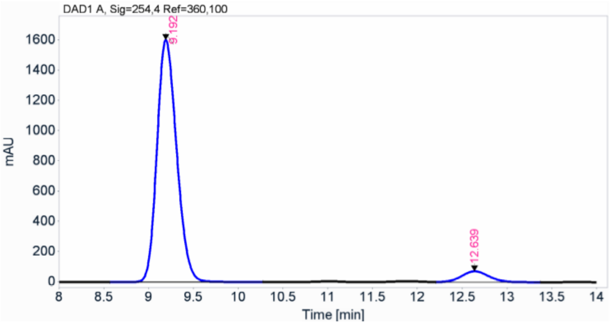

Signal:	DAD1 A, Sig=254,4 Ref=360,100				
RT [min]	Type	Width [min]	Area	Height	Area%
9.192	BB	0.2378	24413.246	1601.8511	94.08
12.639	VB	0.3307	1534.840	72.0049	5.92

(+)-(3a*S*,4*S*,7a*S*)-6-(3-Chloro-5-methylphenyl)-3a-hydroxy-7a-methyl-4-(prop-1-en-2-yl)-2,3,3a,4,7,7a-hexahydro-1*H*-inden-1-one (2ba) and (+)-(3a*S*,4*R*,7a*S*)-6-(3-chloro-5-methylphenyl)-3a-hydroxy-7a-methyl-4(prop-1-en-2-yl)-2,3,3a,4,7,7a-hexahydro-1*H*-inden-1-one (2bb)

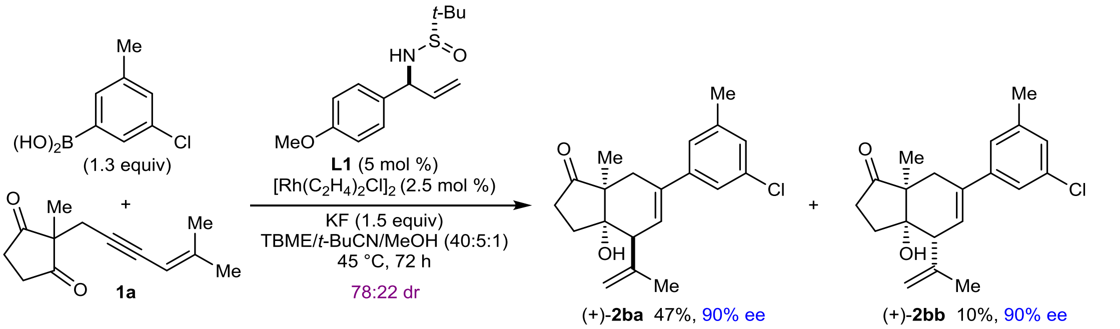

$[Rh(C_2H_4)Cl]_2$ (2.5 mg, 6.4 µmol) and **L1**[13] (3.4 mg, 13 µmol) were added to an oven-dried microwave vial, and the vial was sealed with a septum and purged with nitrogen for 1 h. Degassed TBME (1 mL) and degassed *t*-BuCN (0.5 mL) were added and the mixture was stirred for 30 min at room temperature. Meanwhile, in a separate oven-dried microwave vial, enynone **1a** (51.0 mg, 0.250 mmol), 3-chloro-5-methylphenylboronic acid (63.9 mg, 0.375 mmol), and KF (21.8 mg, 0.375 mmol) were added. The vial was sealed with a septum-lined microwave vial cap and purged with nitrogen for 1 h. Degassed TBME (3.2 mL), the solution of catalyst (see above), and MeOH (0.10 mL) were added, and the mixture was stirred at 45 °C for 72 h. The reaction was cooled to room temperature, water (10 mL) and saturated aqueous NH_4Cl solution (10 mL) were added, and the mixture was extracted with EtOAc (3 × 10 mL). The combined organic phases were washed with brine (15 mL), dried (Na_2SO_4), filtered, and concentrated *in vacuo*. ^{1}H NMR analysis indicated a 78:22 mixture of diastereomers (+)–**2ba** and (+)–**2bb**, respectively. The mixture was purified by column chromatography (94% $CHCl_3$, 5% EtOAc, 1% Et_3N) to give alcohol (+)–**2ba** (39 mg, 47%) as a beige solid and alcohol (+)–**2bb** (8 mg, 10%) as a colorless oil. The spectroscopic data for (+)–

2ba matched those of the racemic compound reported above. In addition:

Data for (+)–**2ba**: $[\alpha]_D^{20}$ +9.8 (*c* 0.41, $CHCl_3$). Enantiomeric excess was determined by HPLC using a Chiralcel IC column (95:5 *iso*-hexane:*i*-PrOH, 1.5 mL/min, 254 nm, 25 °C); t_r (major) = 14.7 min, t_r (minor) = 17.1 min, 90% ee.

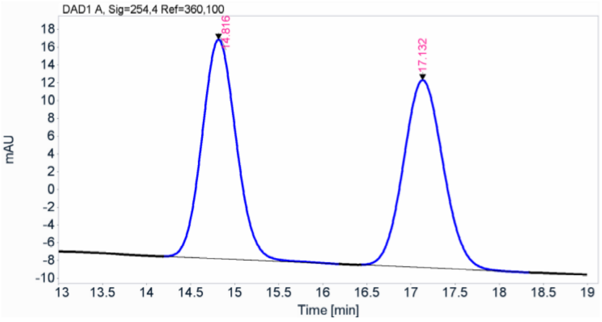

Signal: DAD1 A, Sig=254,4 Ref=360,100

RT [min]	Type	Width [min]	Area	Height	Area%
14.816	BB	0.4191	666.664	24.6811	50.12
17.132	BBA	0.4905	663.369	21.1173	49.88

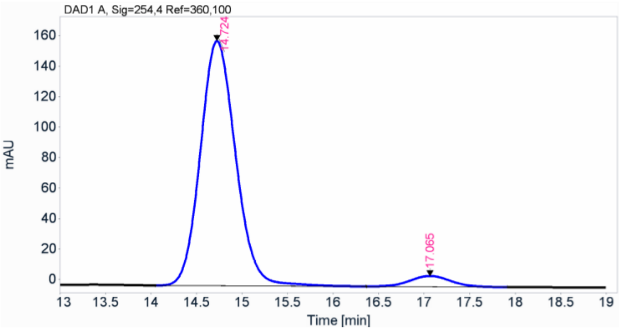

Signal: DAD1 A, Sig=254,4 Ref=360,100

RT [min]	Type	Width [min]	Area	Height	Area%
14.724	BB	0.4176	4315.103	160.5108	95.14
17.065	BB	0.4850	220.560	7.0889	4.86

Data for (+)–**2bb**: R_f = 0.43 (94% $CHCl_3$, 5% EtOAc, 1% Et_3N); $[\alpha]_D^{21}$ +50.0 (*c* 0.80, $CHCl_3$). IR 3445 (O–H), 2982, 1731 (C=O), 1599, 1436, 1374, 1036 cm^{-1}; ^{1}H NMR (400 MHz, $CDCl_3$) δ 7.16 (1H, t, *J* = 1.9 Hz, Ar**H**), 7.08 (1H, s, Ar**H**), 7.05 (1H, s, Ar**H**), 5.93 (1H, ddd, *J* = 3.4, 2.3, 0.9 Hz, C=C**H**), 5.22 (1H, t, *J* = 1.6 Hz, C=C$\mathbf{H_A}H_B$), 4.99–4.96 (1H, m, C=C$H_A\mathbf{H_B}$), 3.13-3.09 (1H, m, C=CC**H**), 2.63–2.52 (2H, m, O=CC$\mathbf{H_2}$), 2.47-2.37 (1H, m, C=CC$\mathbf{H_A}H_B$), 2.33 (3H, s, ArC$\mathbf{H_3}$), 2.29-2.09 (3H, m, C=CC$H_A\mathbf{H_B}$ and HOCC$\mathbf{H_2}$), 1.98–1.93 (4H, m, C=CC$\mathbf{H_3}$ and O**H**), 1.18 (3H, s, O=CCC$\mathbf{H_3}$); ^{13}C NMR (101 MHz, $CDCl_3$) δ 218.3 (C), 145.0 (C), 142.5 (C), 139.7 (C), 134.1 (C), 133.3 (C), 128.0 (CH), 126.4 (CH), 124.3 (CH), 122.7 (CH), 117.2 (CH_2), 77.6 (C), 52.7 (C), 51.3 (CH), 33.7 (CH_2), 33.6 (CH_2), 33.4 (CH_2), 24.5 (CH_3), 21.3 (CH_3), 17.0 (CH_3) HRMS (ESI) Exact mass calcd for $[C_{20}H_{23}{}^{35}ClNaO_2]^+$ $[M+Na]^+$: 353.1284, found: 353.1231. Enantiomeric excess was determined by HPLC using a Chiralcel IC column (95:5 *iso*-hexane:*i*-PrOH, 1.5 mL/min, 254 nm, 25 °C); t_r (major) = 9.3 min, t_r (minor) = 12.6 min, 90% ee.

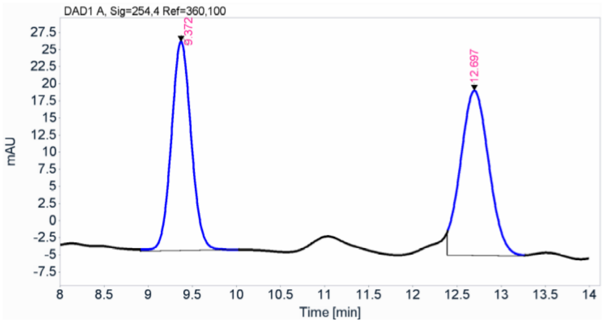

Signal: DAD1 A, Sig=254,4 Ref=360,100

RT [min]	Type	Width [min]	Area	Height	Area%
9.372	MM	0.2599	476.830	30.5807	47.05
12.697	MM	0.3712	536.611	24.0914	52.95

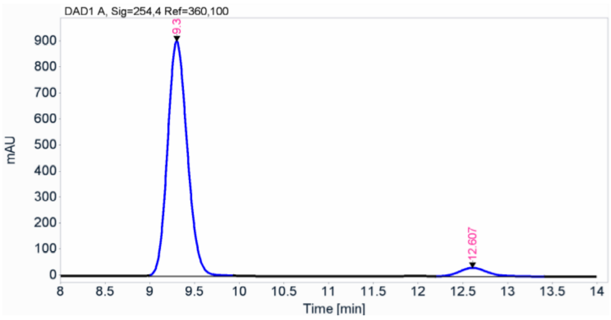

Signal: DAD1 A, Sig=254,4 Ref=360,100

RT [min]	Type	Width [min]	Area	Height	Area%
9.300	MM	0.2562	13899.492	904.0563	94.87
12.607	VB	0.3456	750.975	33.4901	5.13

6. NMR Spectra

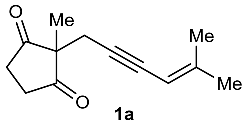

1a

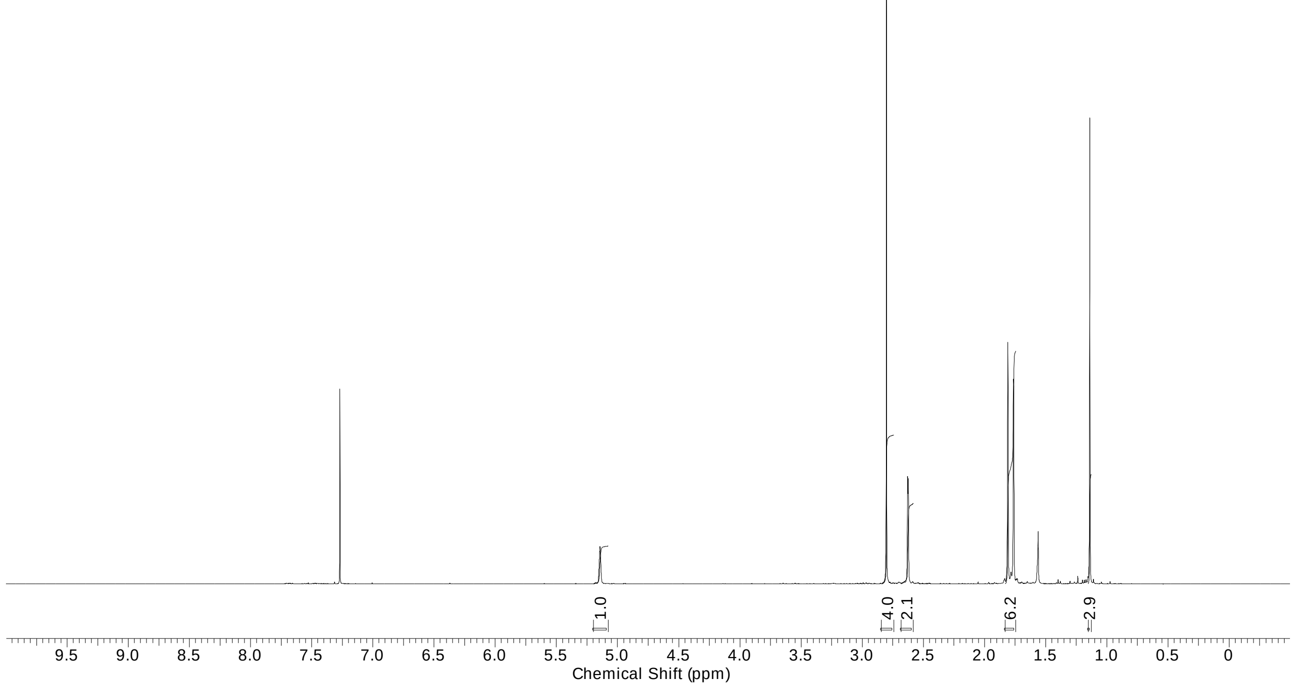

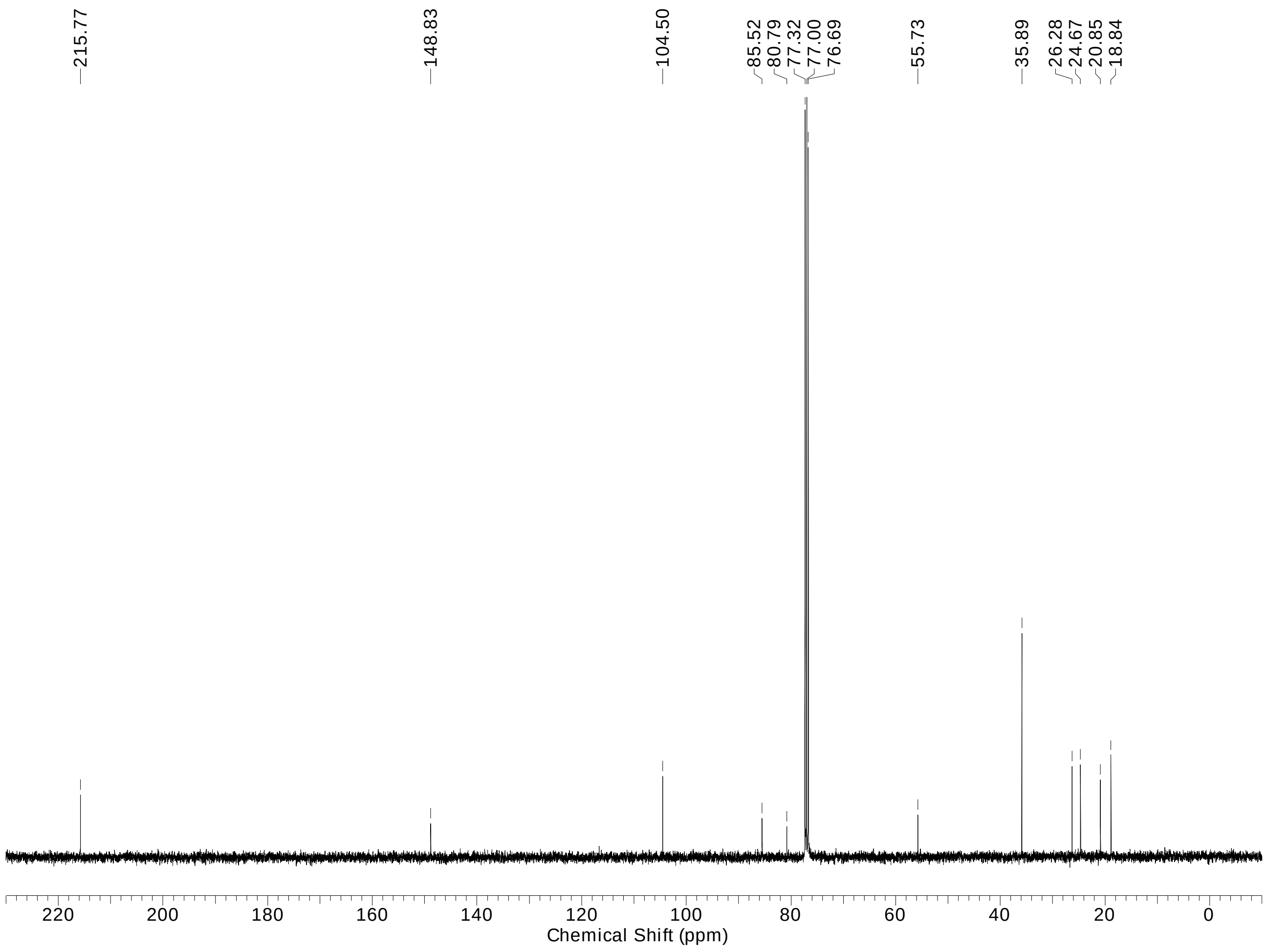

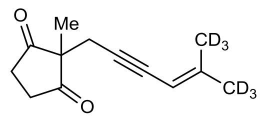
Me
CD3
CD3
O
O

[D]$_6$-**1a**

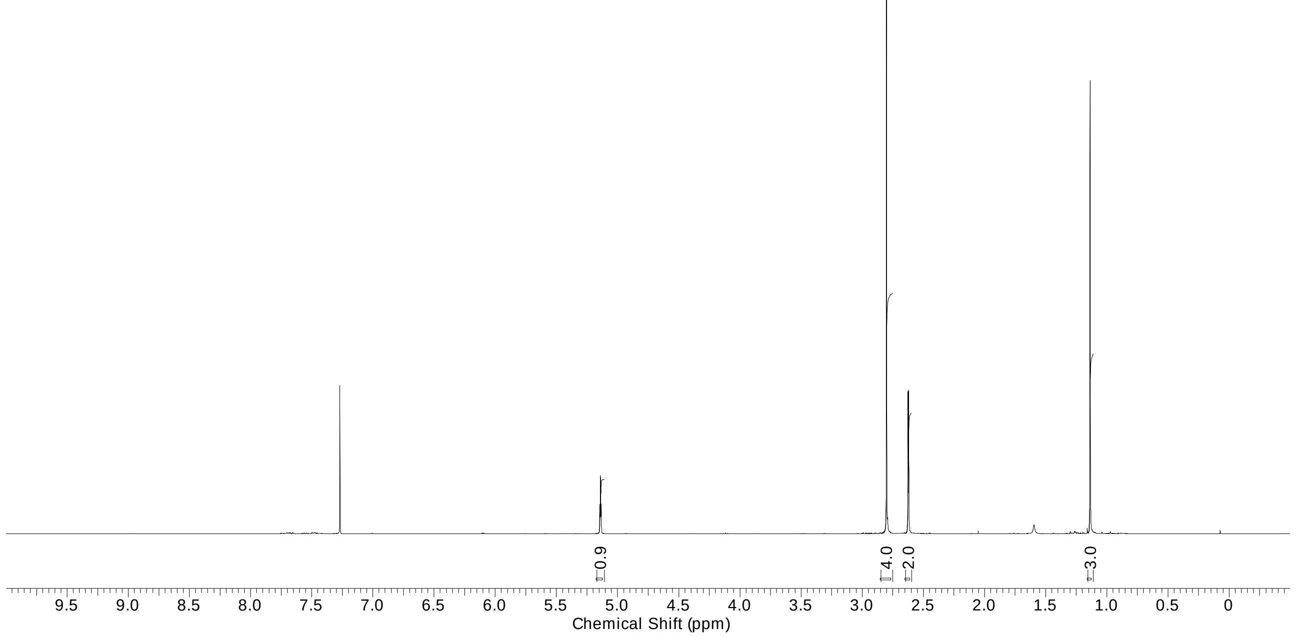
0.9
4.0
2.0
3.0
9.5 9.0 8.5 8.0 7.5 7.0 6.5 6.0 5.5 5.0 4.5 4.0 3.5 3.0 2.5 2.0 1.5 1.0 0.5 0
Chemical Shift (ppm)

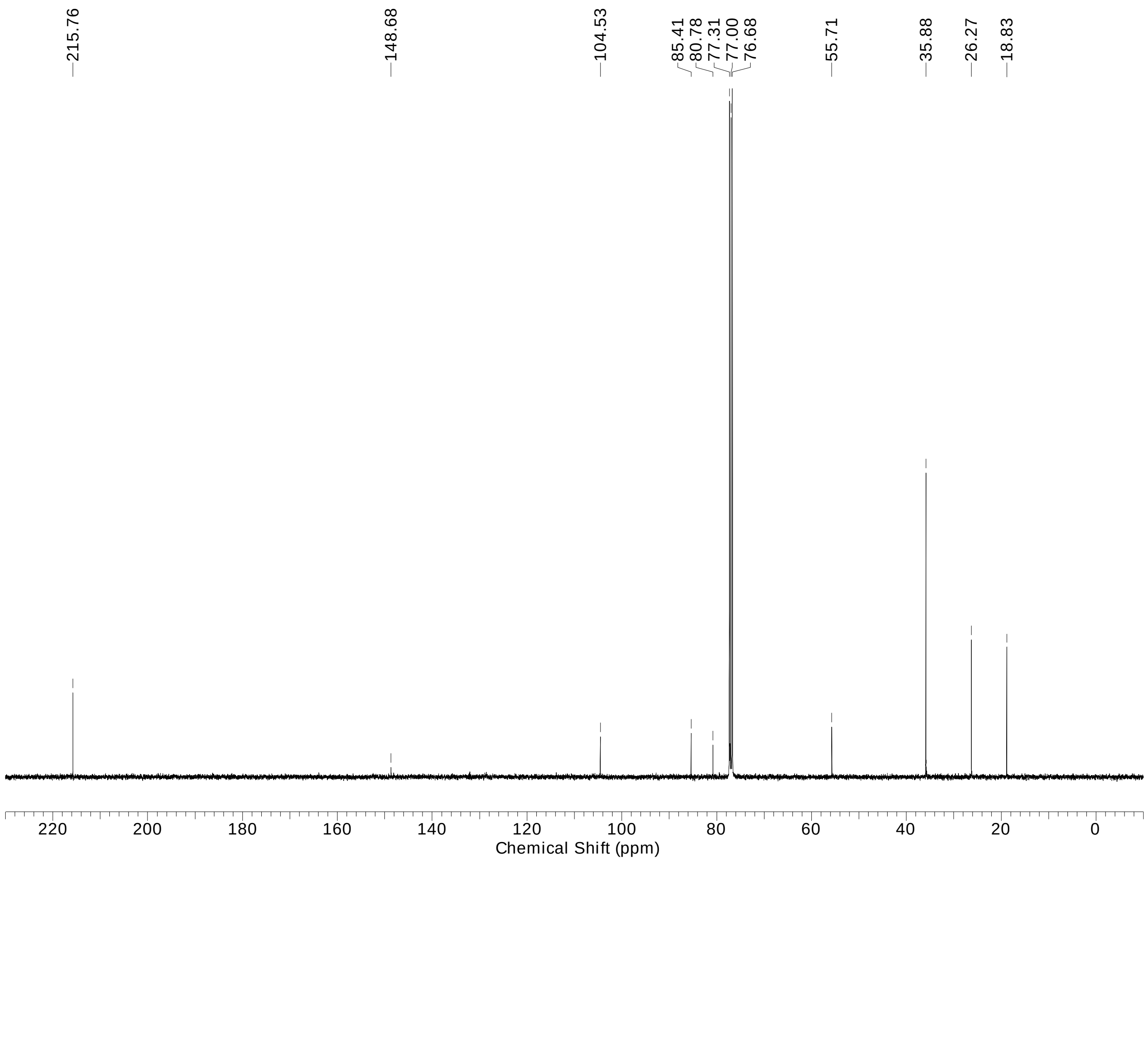
215.76
148.68
104.53
85.41
80.78
77.31
77.00
76.68
55.71
35.88
26.27
18.83
220 200 180 160 140 120 100 80 60 40 20 0
Chemical Shift (ppm)

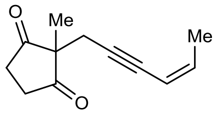
O
Me
Me
O

1b

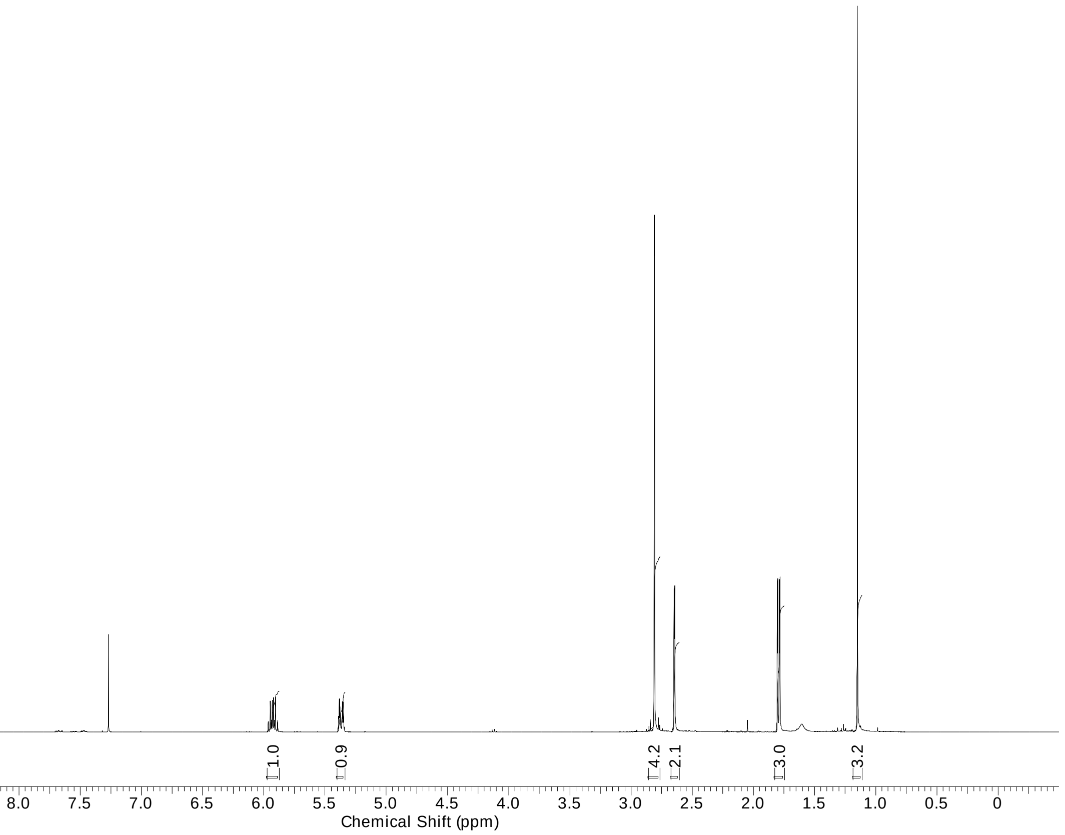
1.0
0.9
4.2
2.1
3.0
3.2
9.5 9.0 8.5 8.0 7.5 7.0 6.5 6.0 5.5 5.0 4.5 4.0 3.5 3.0 2.5 2.0 1.5 1.0 0.5 0
Chemical Shift (ppm)

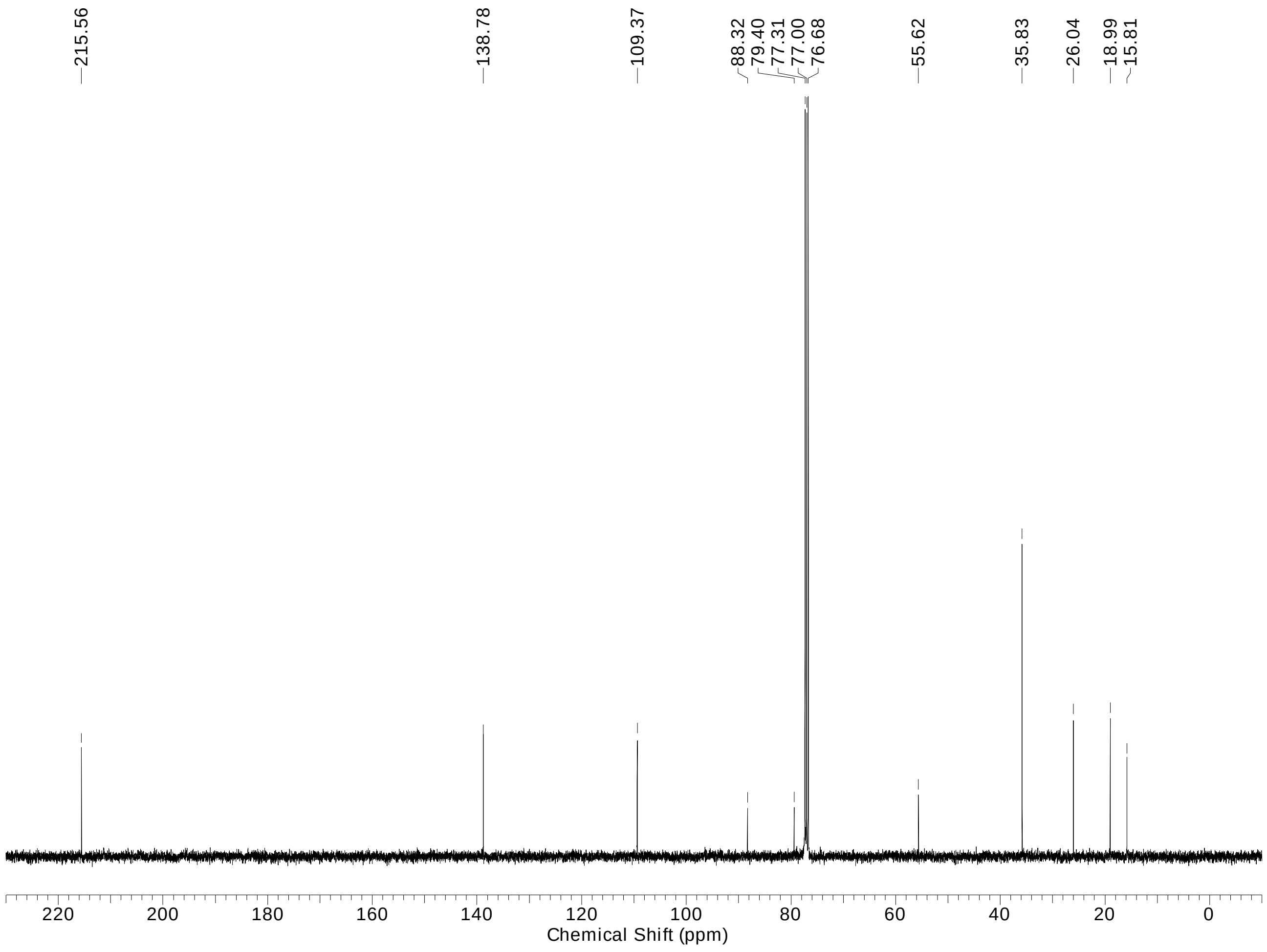
215.56
138.78
109.37
88.32
79.40
77.31
77.00
76.68
55.62
35.83
26.04
18.99
15.81
220 200 180 160 140 120 100 80 60 40 20 0
Chemical Shift (ppm)

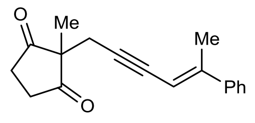
Me
Me
Ph
O
O

1c

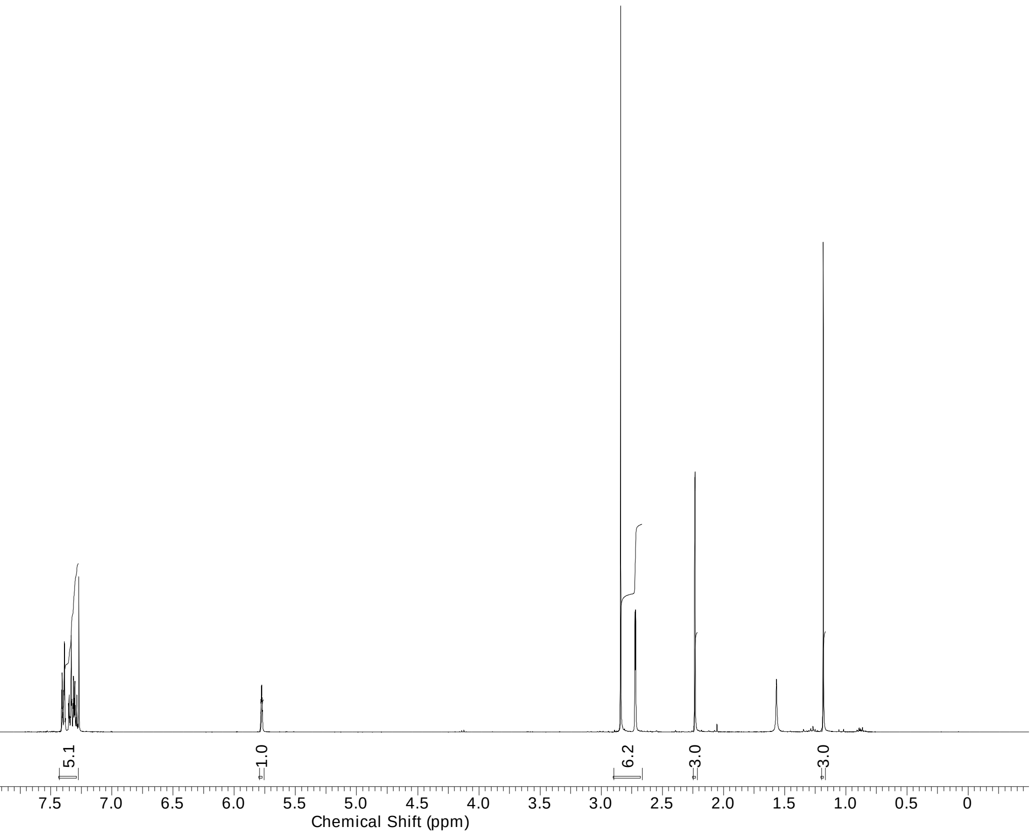
5.1
1.0
6.2
3.0
3.0
Chemical Shift (ppm)

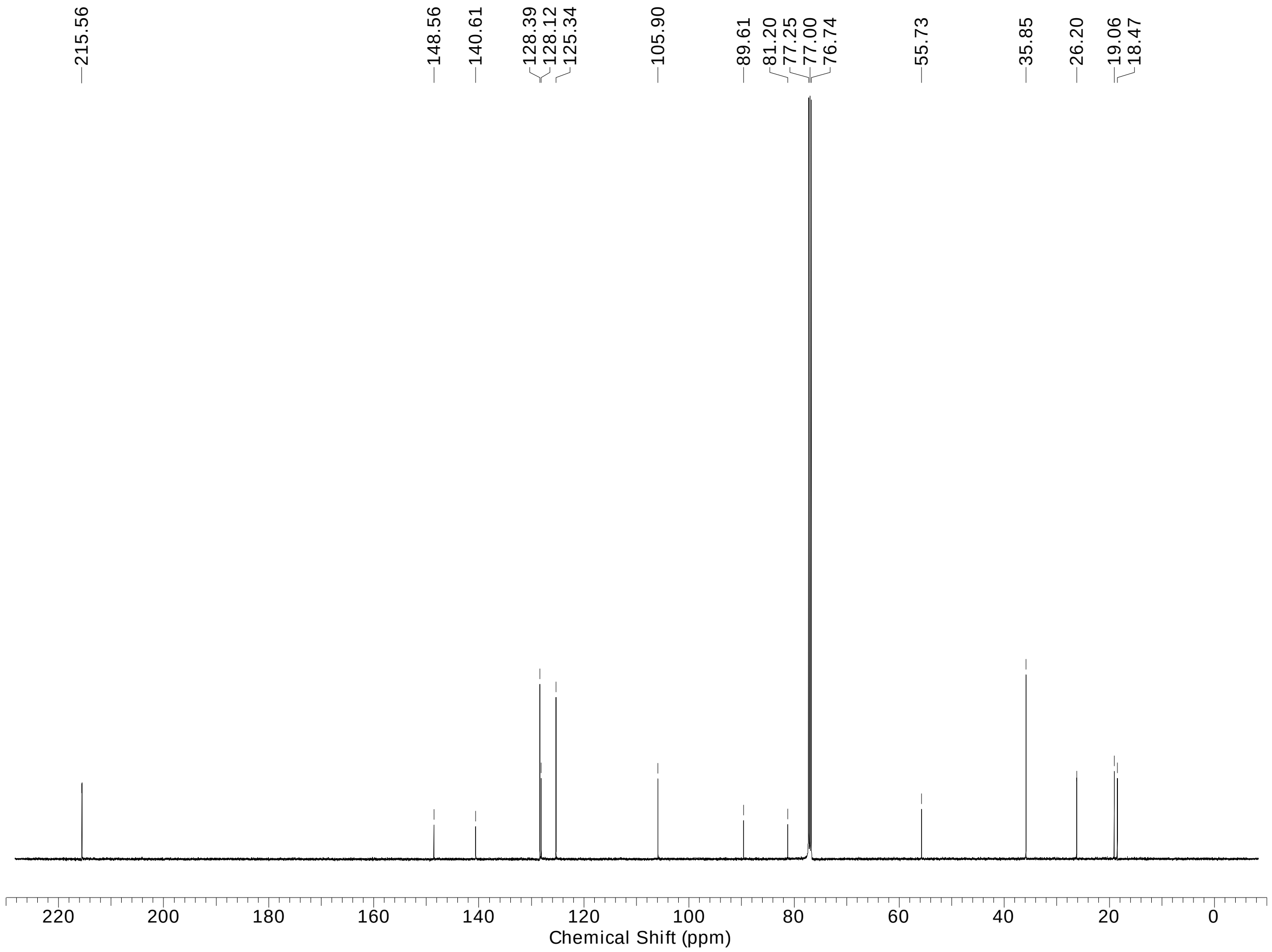
215.56
148.56
140.61
128.39
128.12
125.34
105.90
89.61
81.20
77.25
77.00
76.74
55.73
35.85
26.20
19.06
18.47
Chemical Shift (ppm)

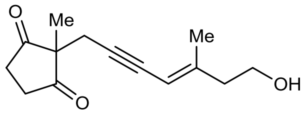

Me
Me
O
O
OH

S11

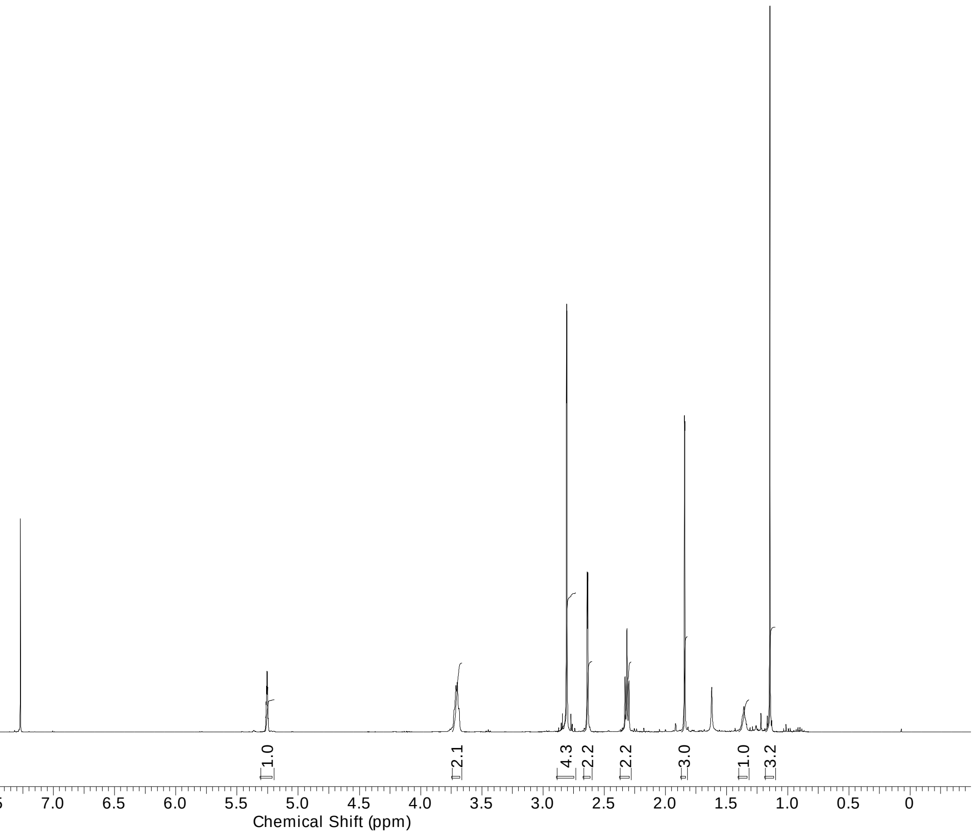

1.0
2.1
4.3
2.2
2.2
3.0
1.0
3.2
9.5 9.0 8.5 8.0 7.5 7.0 6.5 6.0 5.5 5.0 4.5 4.0 3.5 3.0 2.5 2.0 1.5 1.0 0.5 0
Chemical Shift (ppm)

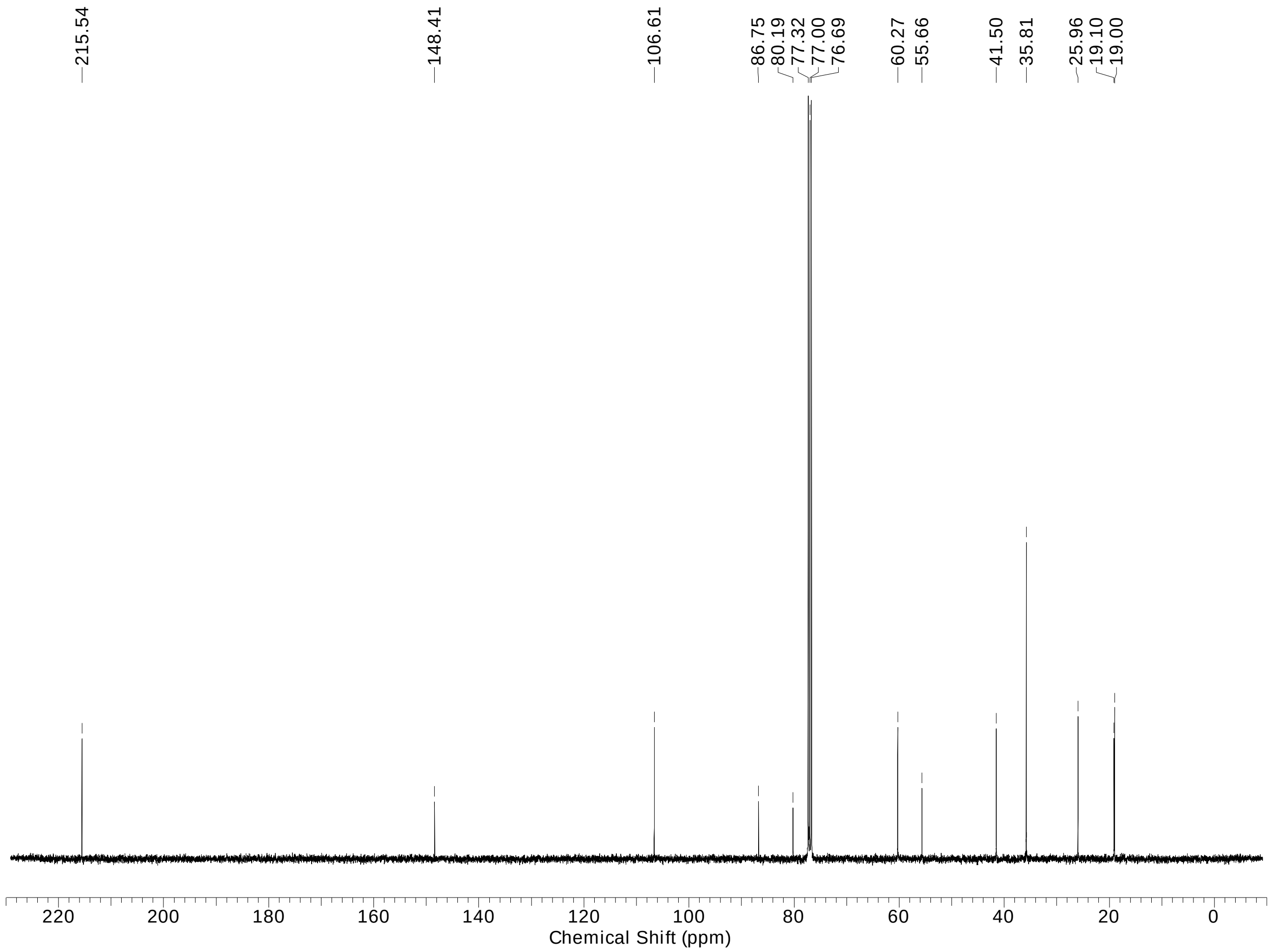

215.54
148.41
106.61
86.75
80.19
77.32
77.00
76.69
60.27
55.66
41.50
35.81
25.96
19.10
19.00
220 200 180 160 140 120 100 80 60 40 20 0
Chemical Shift (ppm)

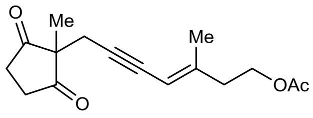
O
Me
Me
OAc
O

1d

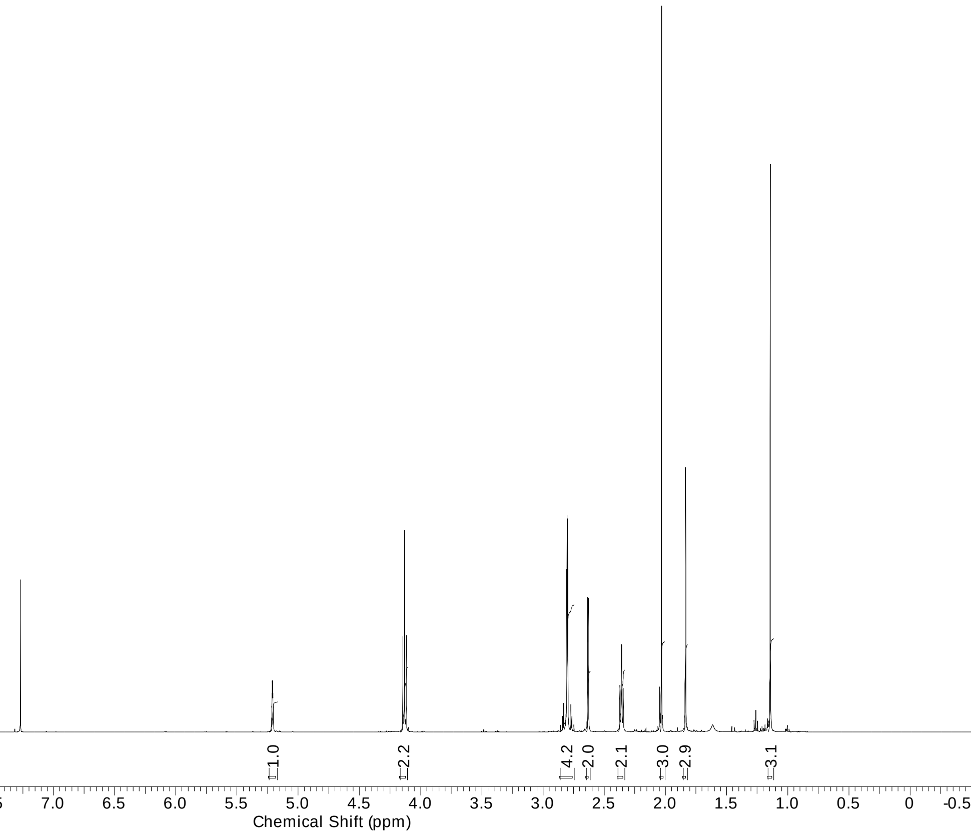
1.0
2.2
4.2
2.0
2.1
3.0
2.9
3.1
9.5 9.0 8.5 8.0 7.5 7.0 6.5 6.0 5.5 5.0 4.5 4.0 3.5 3.0 2.5 2.0 1.5 1.0 0.5 0 -0.5
Chemical Shift (ppm)

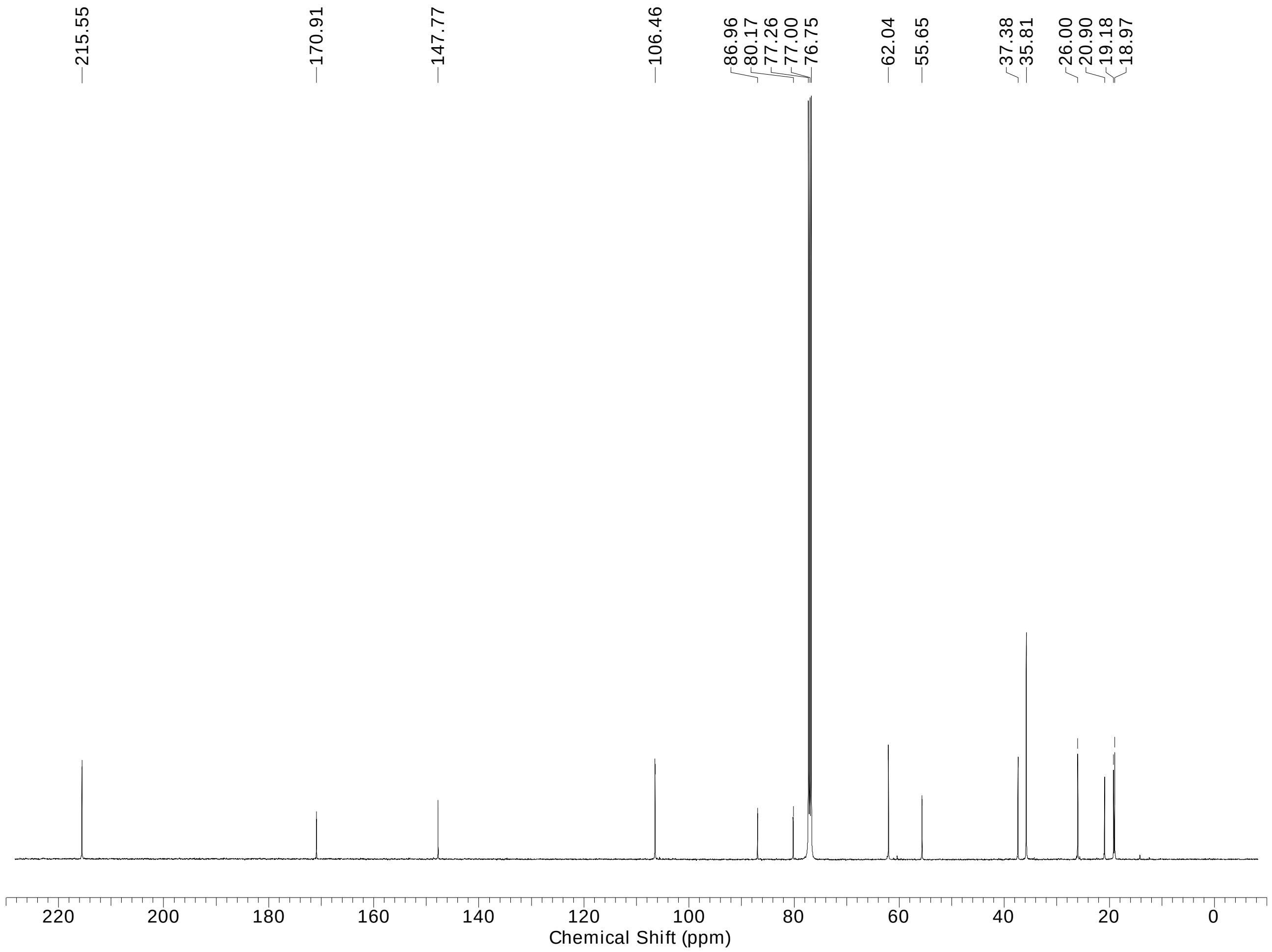
215.55
170.91
147.77
106.46
86.96
80.17
77.26
77.00
76.75
62.04
55.65
37.38
35.81
26.00
20.90
19.18
18.97
220 200 180 160 140 120 100 80 60 40 20 0
Chemical Shift (ppm)

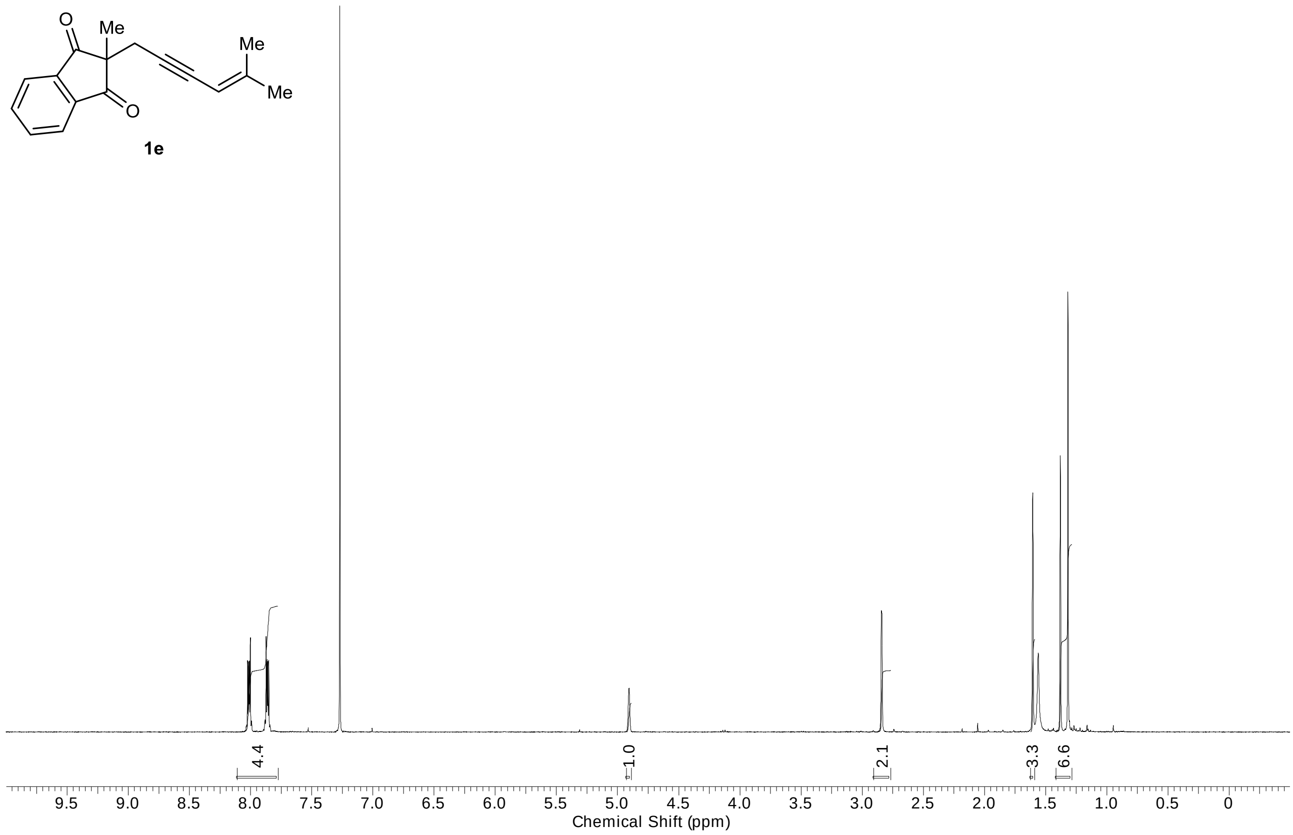
Me
Me
Me
O
O
1e
4.4
1.0
2.1
3.3
6.6
9.5 9.0 8.5 8.0 7.5 7.0 6.5 6.0 5.5 5.0 4.5 4.0 3.5 3.0 2.5 2.0 1.5 1.0 0.5 0
Chemical Shift (ppm)

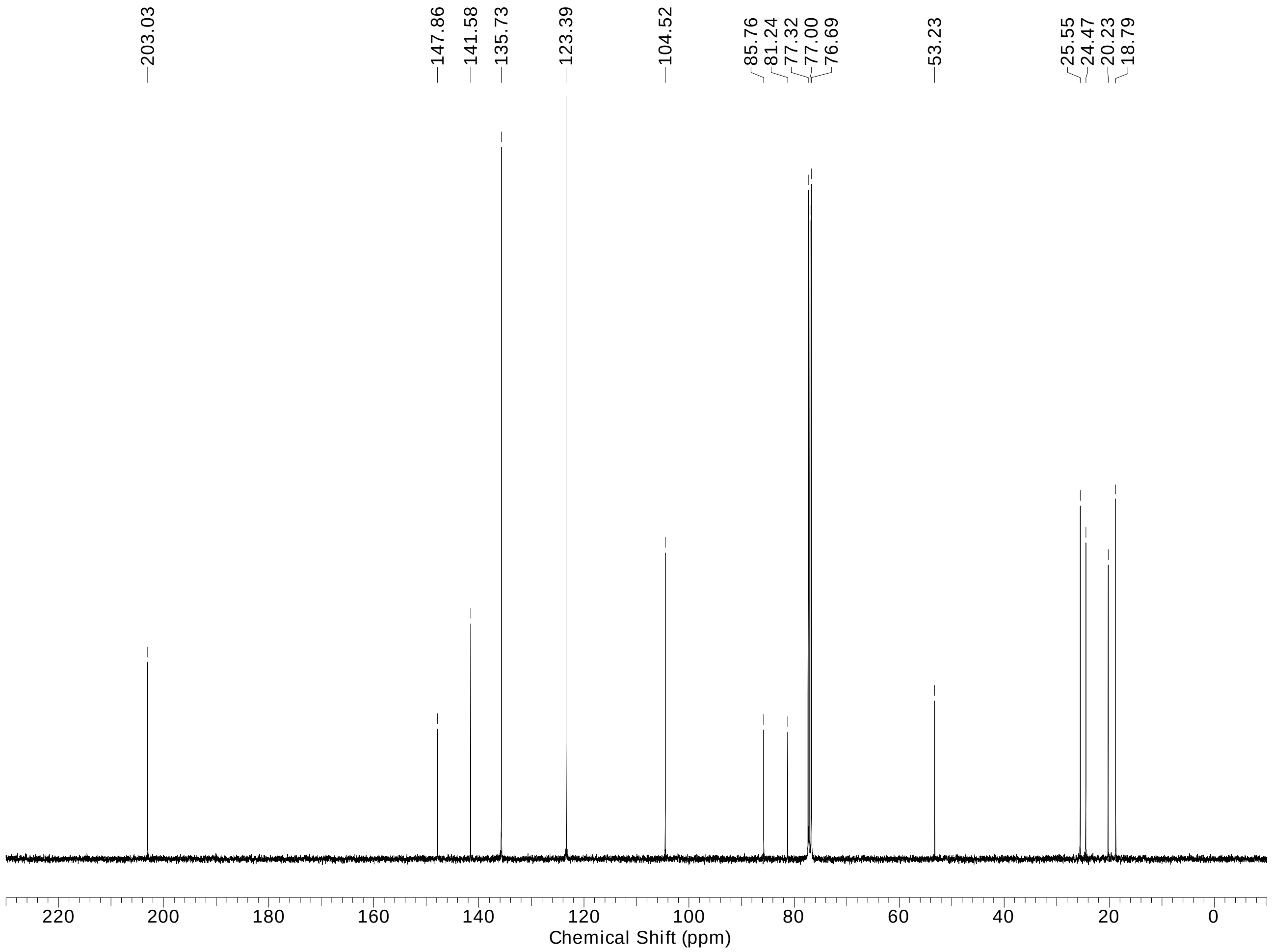
203.03
147.86
141.58
135.73
123.39
104.52
85.76
81.24
77.32
77.00
76.69
53.23
25.55
24.47
20.23
18.79
220 200 180 160 140 120 100 80 60 40 20 0
Chemical Shift (ppm)

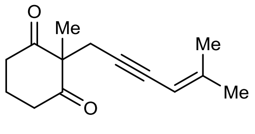
O
Me
Me
Me
O

1f

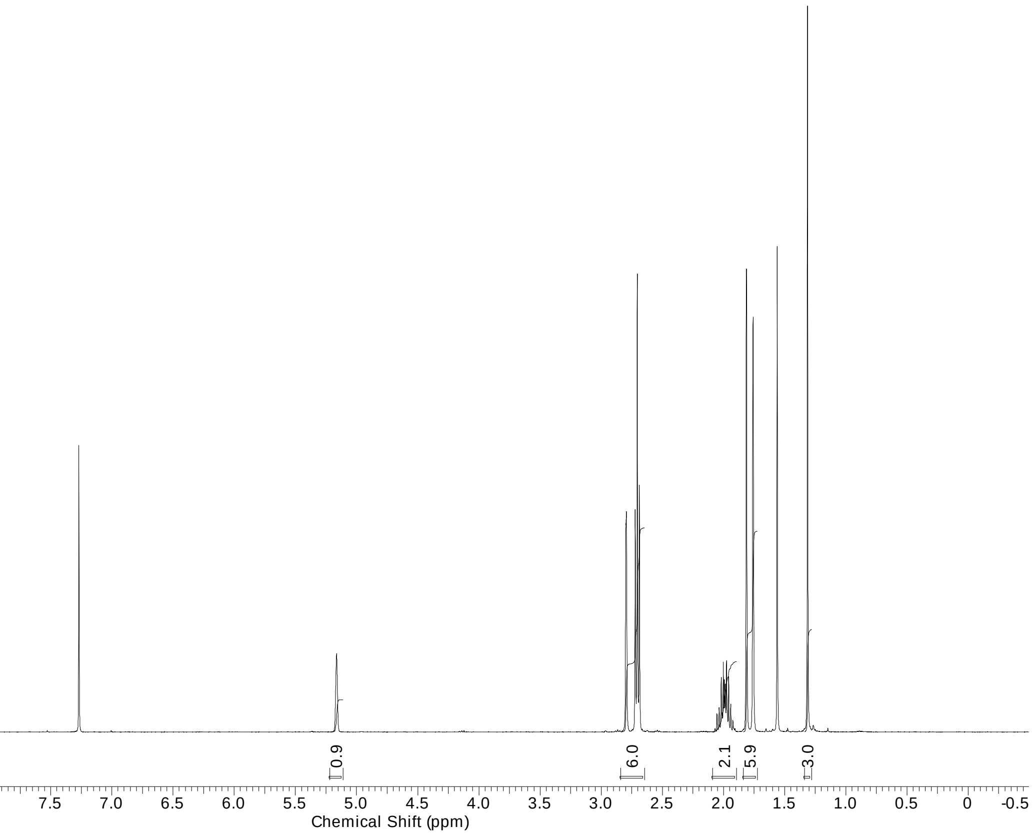
0.9
6.0
2.1
5.9
3.0
9.5 9.0 8.5 8.0 7.5 7.0 6.5 6.0 5.5 5.0 4.5 4.0 3.5 3.0 2.5 2.0 1.5 1.0 0.5 0 -0.5
Chemical Shift (ppm)

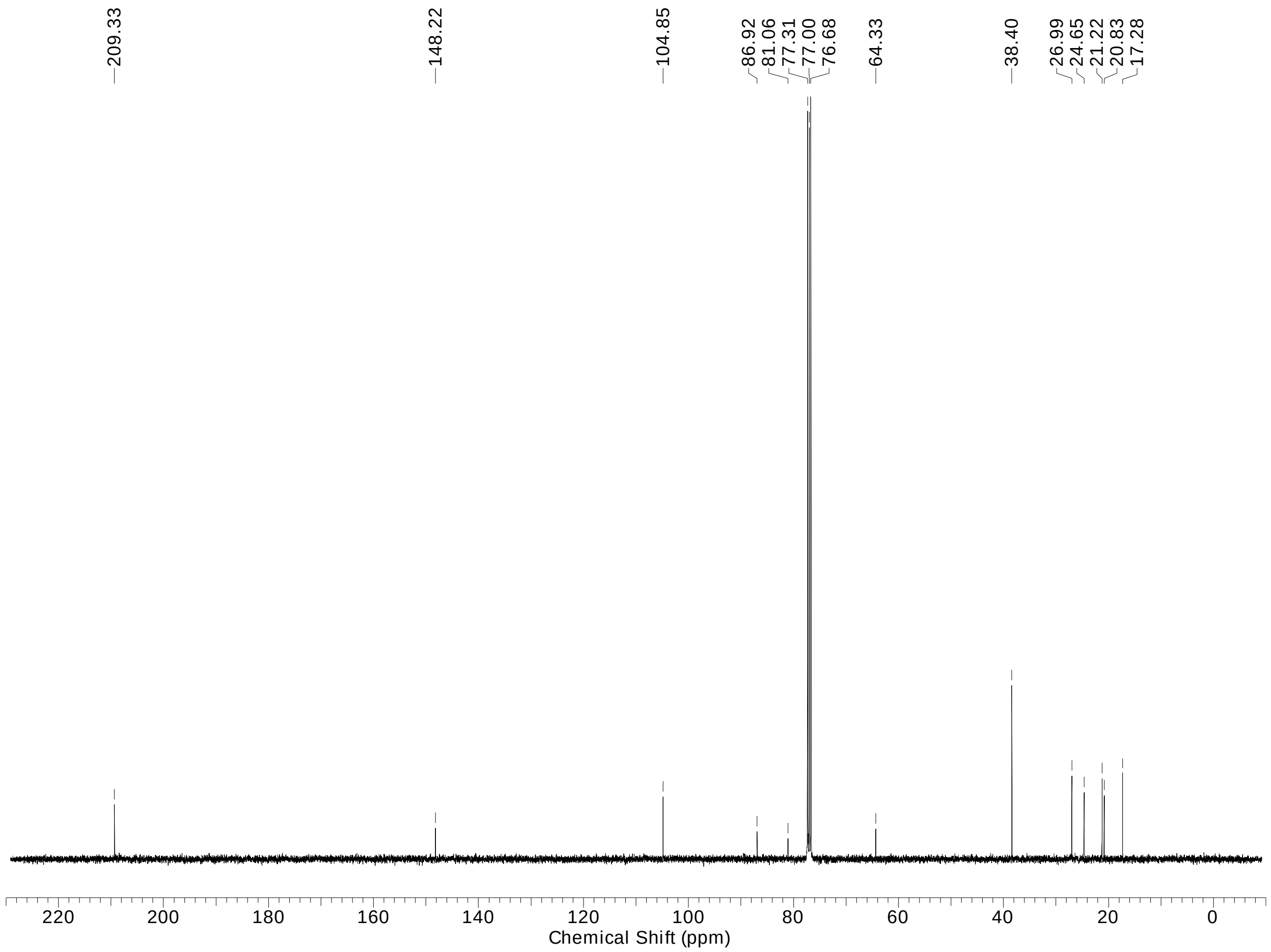
209.33
148.22
104.85
86.92
81.06
77.31
77.00
76.68
64.33
38.40
26.99
24.65
21.22
20.83
17.28
220 200 180 160 140 120 100 80 60 40 20 0
Chemical Shift (ppm)

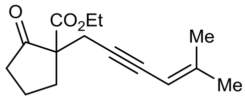
O
CO_2Et
Me
Me

10

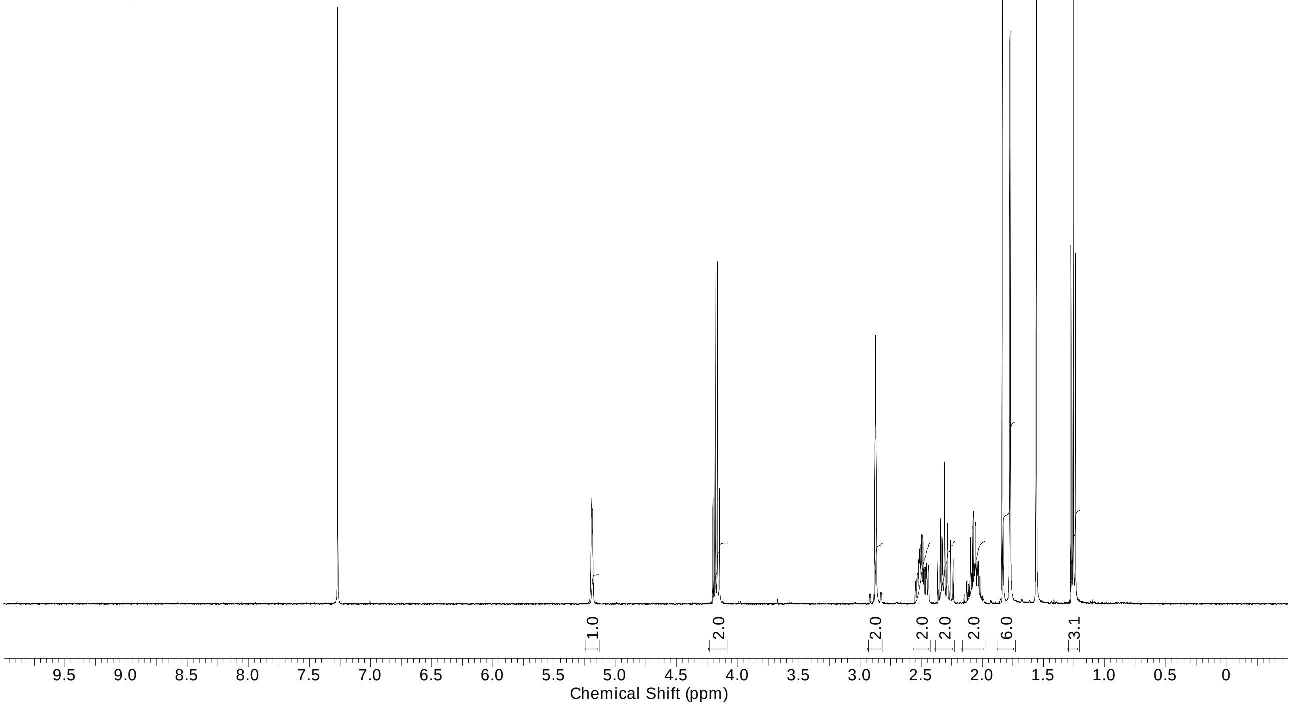
1.0
2.0
2.0
2.0
2.0
2.0
6.0
3.1
9.5 9.0 8.5 8.0 7.5 7.0 6.5 6.0 5.5 5.0 4.5 4.0 3.5 3.0 2.5 2.0 1.5 1.0 0.5 0
Chemical Shift (ppm)

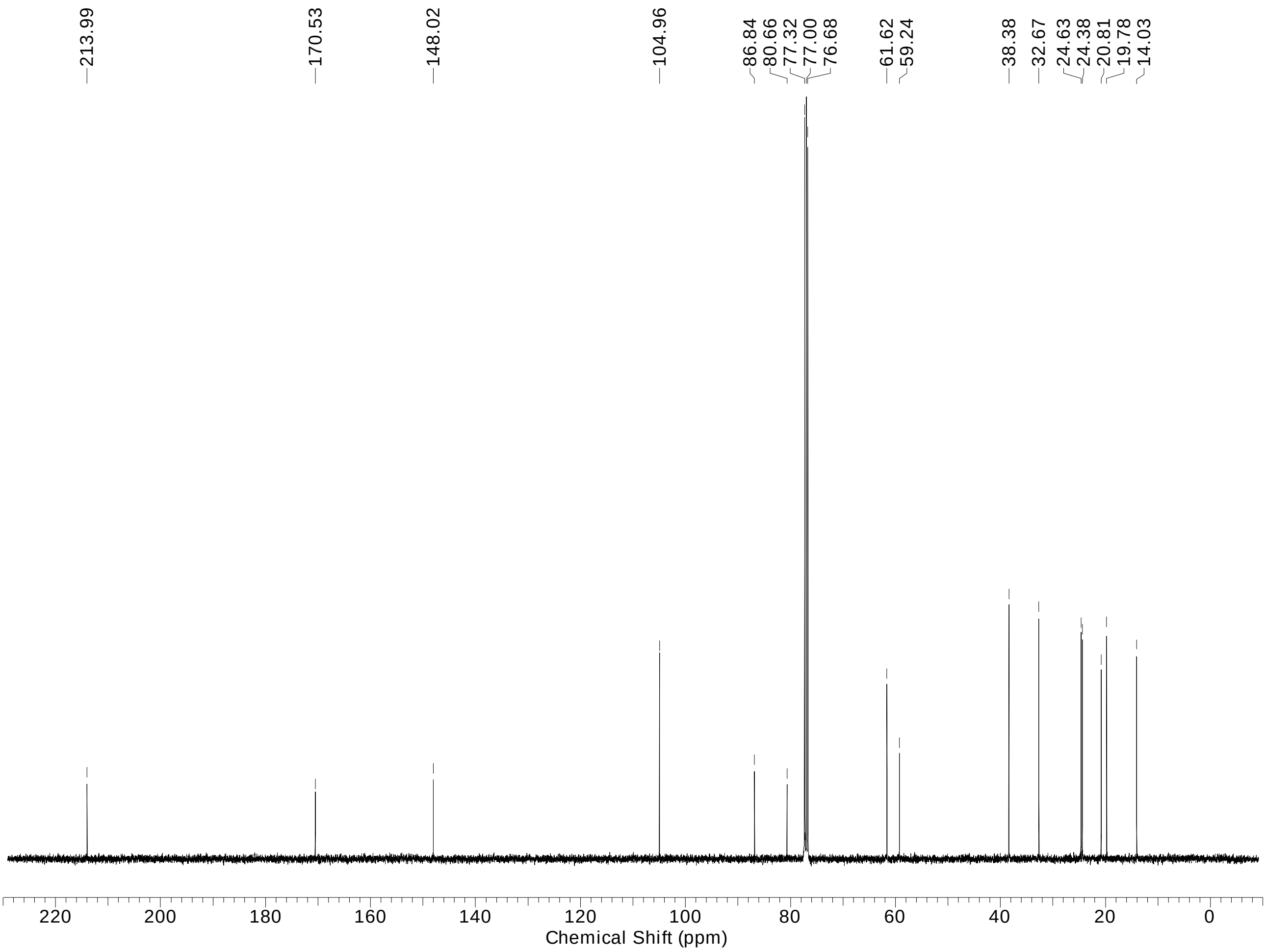
213.99
170.53
148.02
104.96
86.84
80.66
77.32
77.00
76.68
61.62
59.24
38.38
32.67
24.63
24.38
20.81
19.78
14.03
220 200 180 160 140 120 100 80 60 40 20 0
Chemical Shift (ppm)

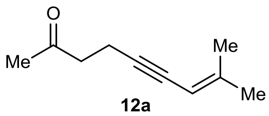
O
Me
Me
Me

12a

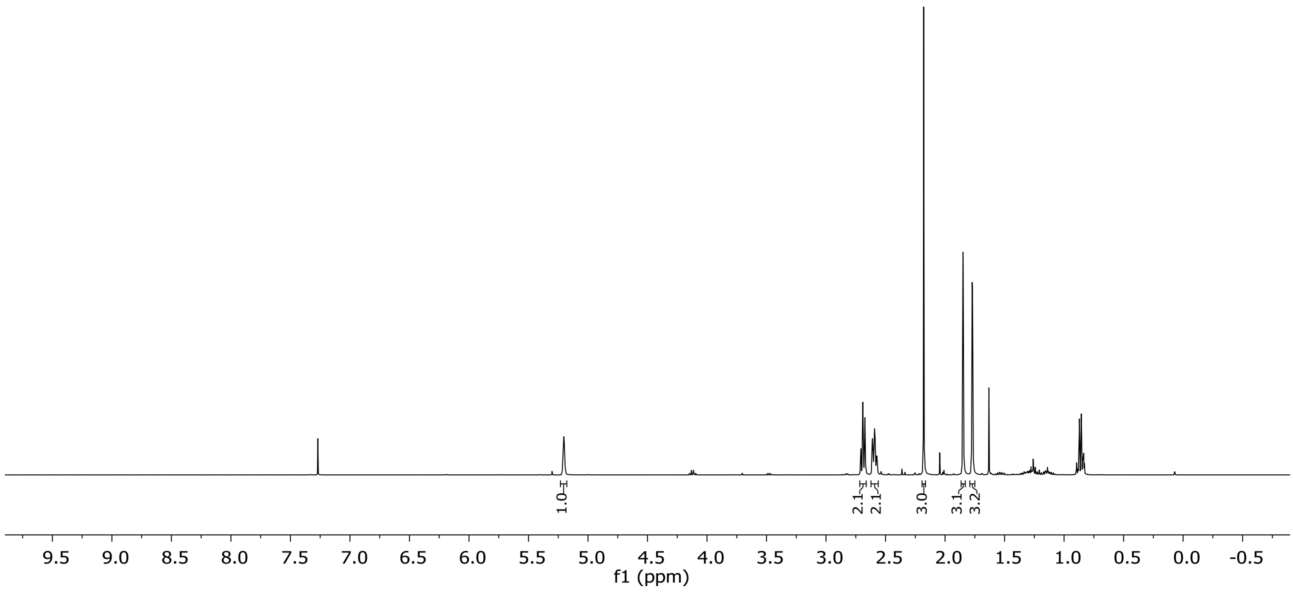
1.0
2.1
2.1
3.0
3.1
3.2
9.5 9.0 8.5 8.0 7.5 7.0 6.5 6.0 5.5 5.0 4.5 4.0 3.5 3.0 2.5 2.0 1.5 1.0 0.5 0.0 -0.5
f1 (ppm)

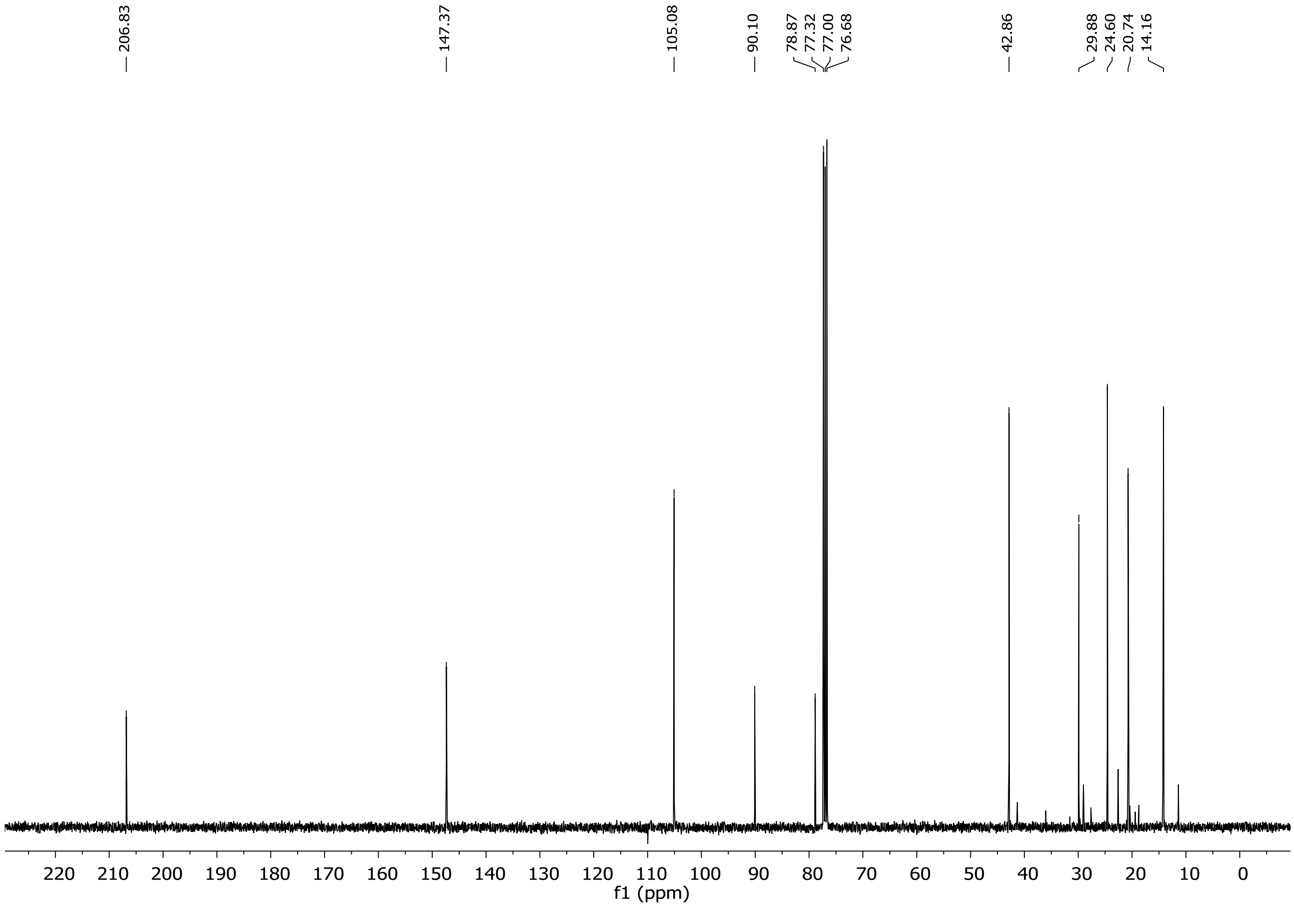
206.83
147.37
105.08
90.10
78.87
77.32
77.00
76.68
42.86
29.88
24.60
20.74
14.16
220 210 200 190 180 170 160 150 140 130 120 110 100 90 80 70 60 50 40 30 20 10 0
f1 (ppm)

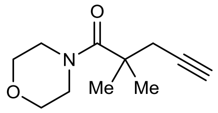
O
N
O
Me
Me

S13

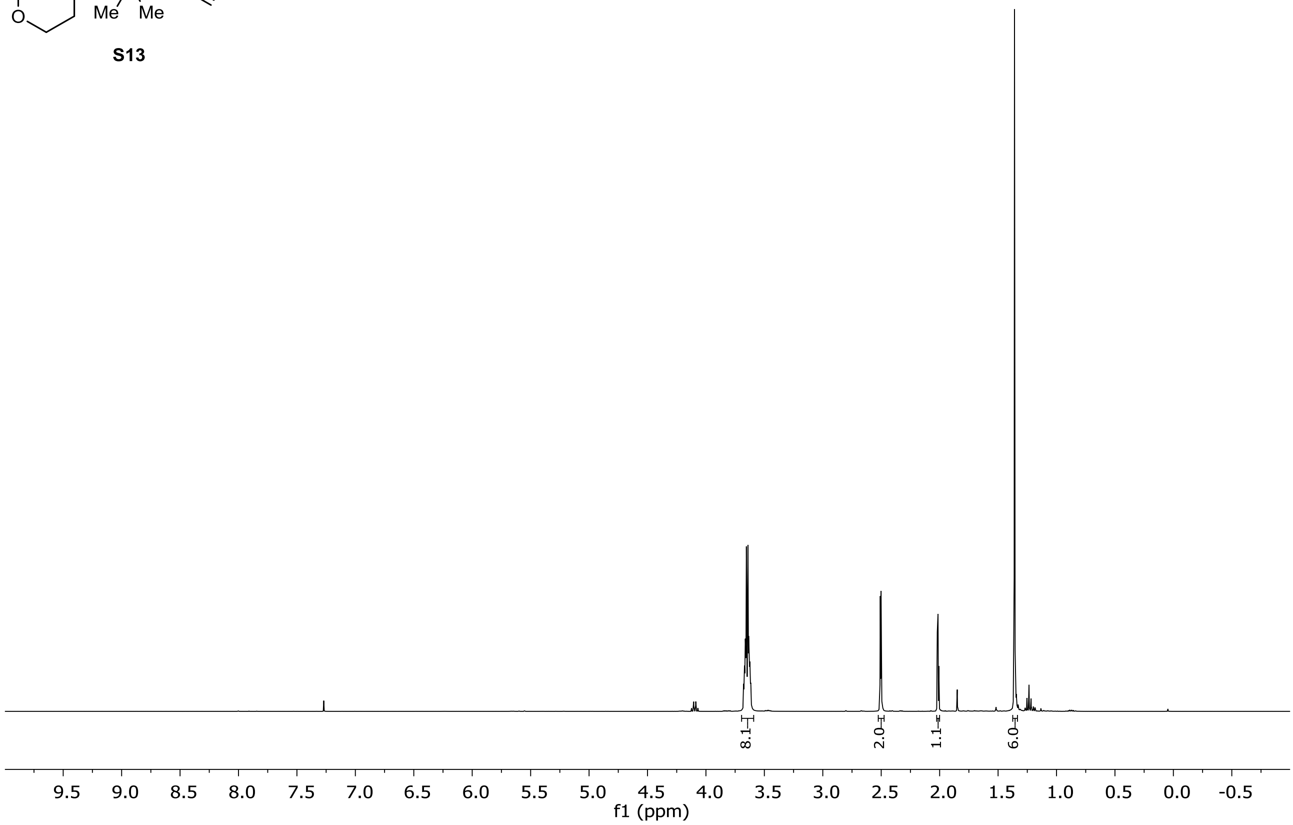
8.1
2.0
1.1
6.0
9.5 9.0 8.5 8.0 7.5 7.0 6.5 6.0 5.5 5.0 4.5 4.0 3.5 3.0 2.5 2.0 1.5 1.0 0.5 0.0 -0.5
f1 (ppm)

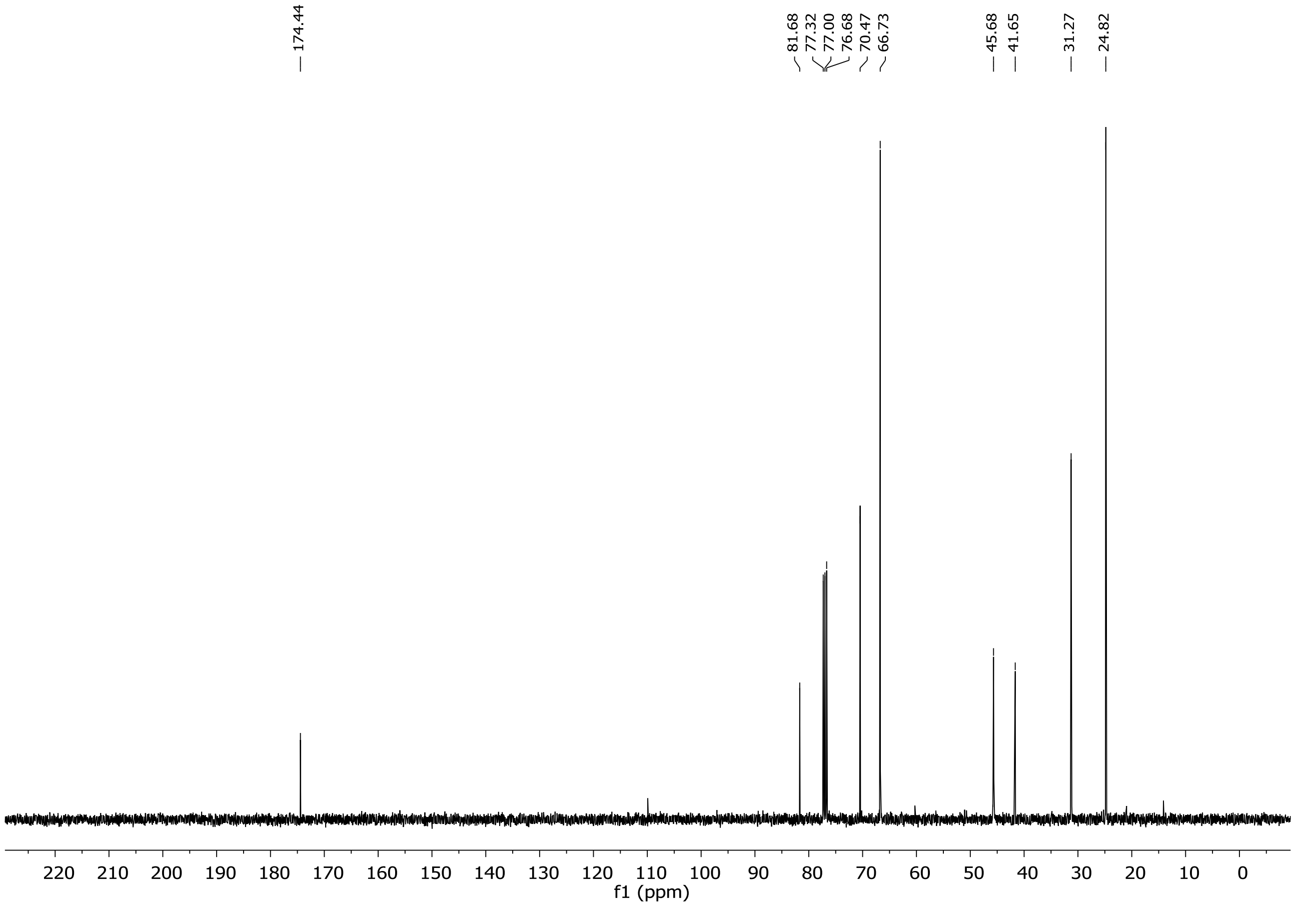
174.44
81.68
77.32
77.00
76.68
70.47
66.73
45.68
41.65
31.27
24.82
220 210 200 190 180 170 160 150 140 130 120 110 100 90 80 70 60 50 40 30 20 10 0
f1 (ppm)

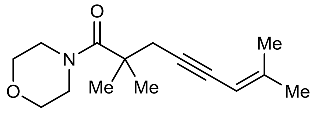
O
N
O
Me
Me
Me
Me

S14

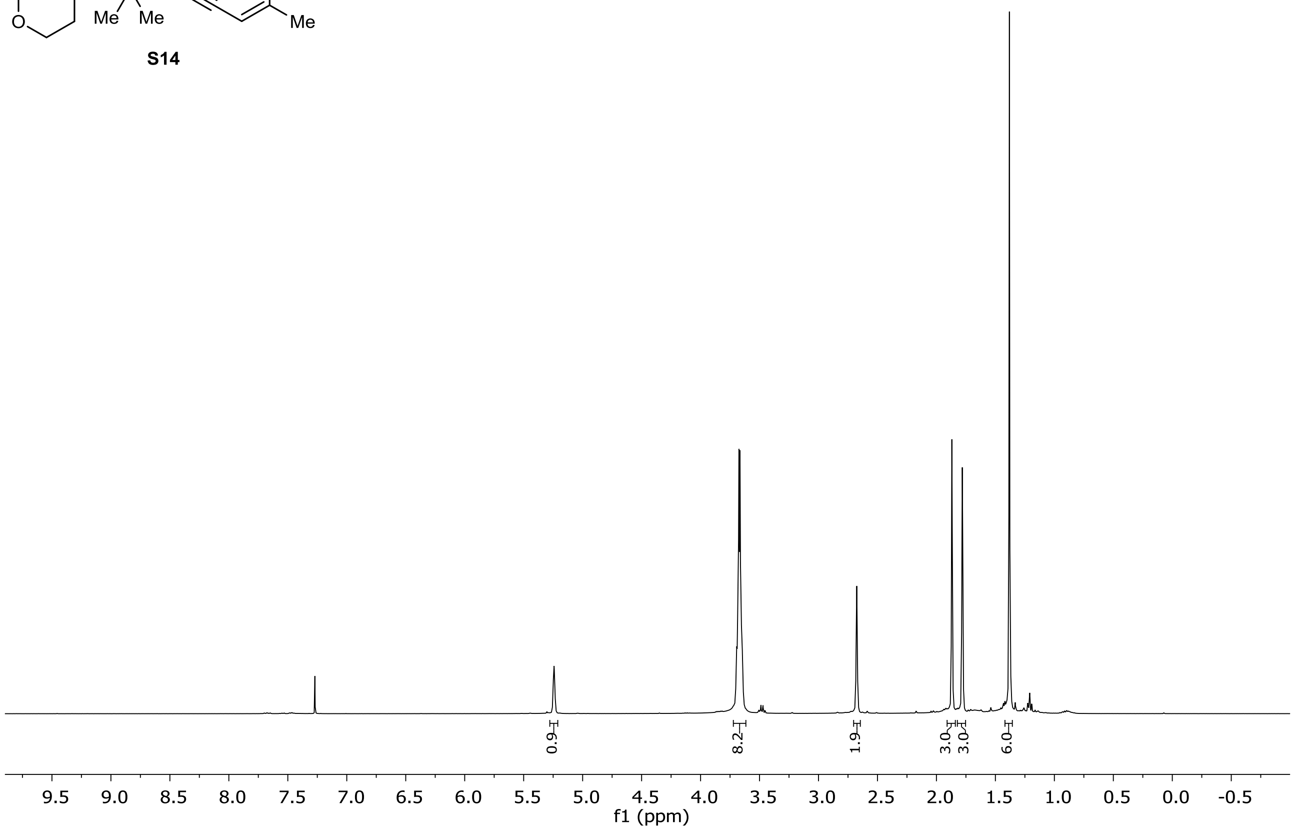
0.9
8.2
1.9
3.0
3.0
6.0
9.5 9.0 8.5 8.0 7.5 7.0 6.5 6.0 5.5 5.0 4.5 4.0 3.5 3.0 2.5 2.0 1.5 1.0 0.5 0.0 -0.5
f1 (ppm)

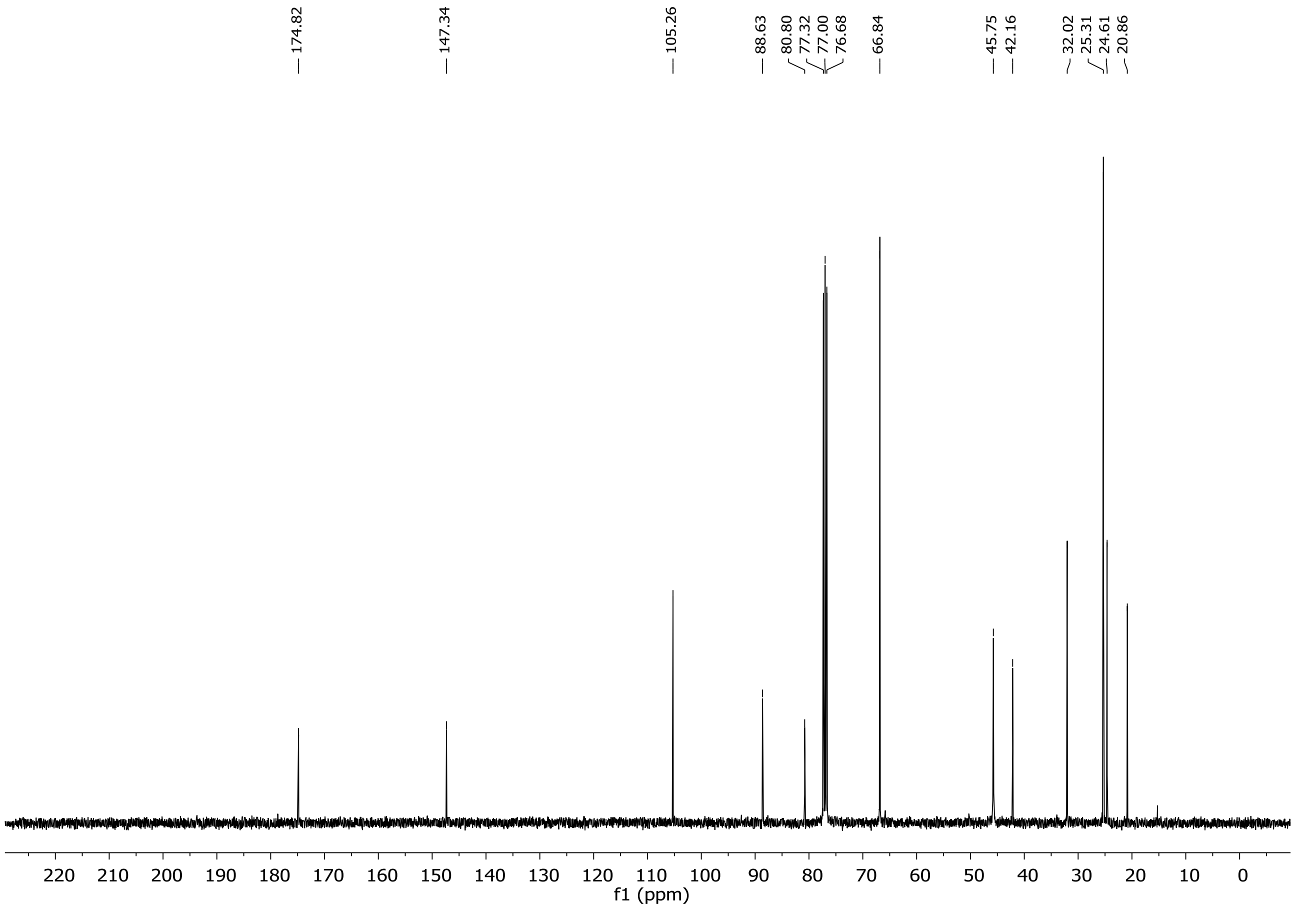
174.82
147.34
105.26
88.63
80.80
77.32
77.00
76.68
66.84
45.75
42.16
32.02
25.31
24.61
20.86
220 210 200 190 180 170 160 150 140 130 120 110 100 90 80 70 60 50 40 30 20 10 0
f1 (ppm)

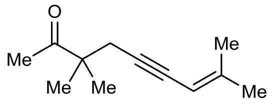
O
Me
Me
Me
Me
Me

12b

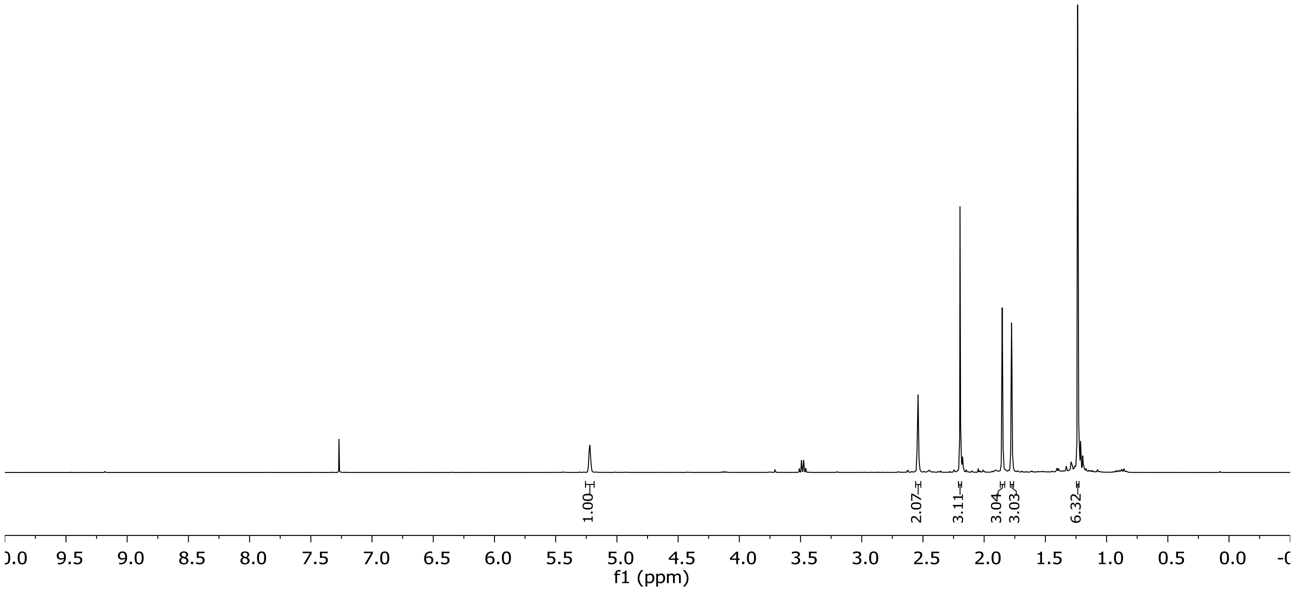
1.00
2.07
3.11
3.04
3.03
6.32
f1 (ppm)

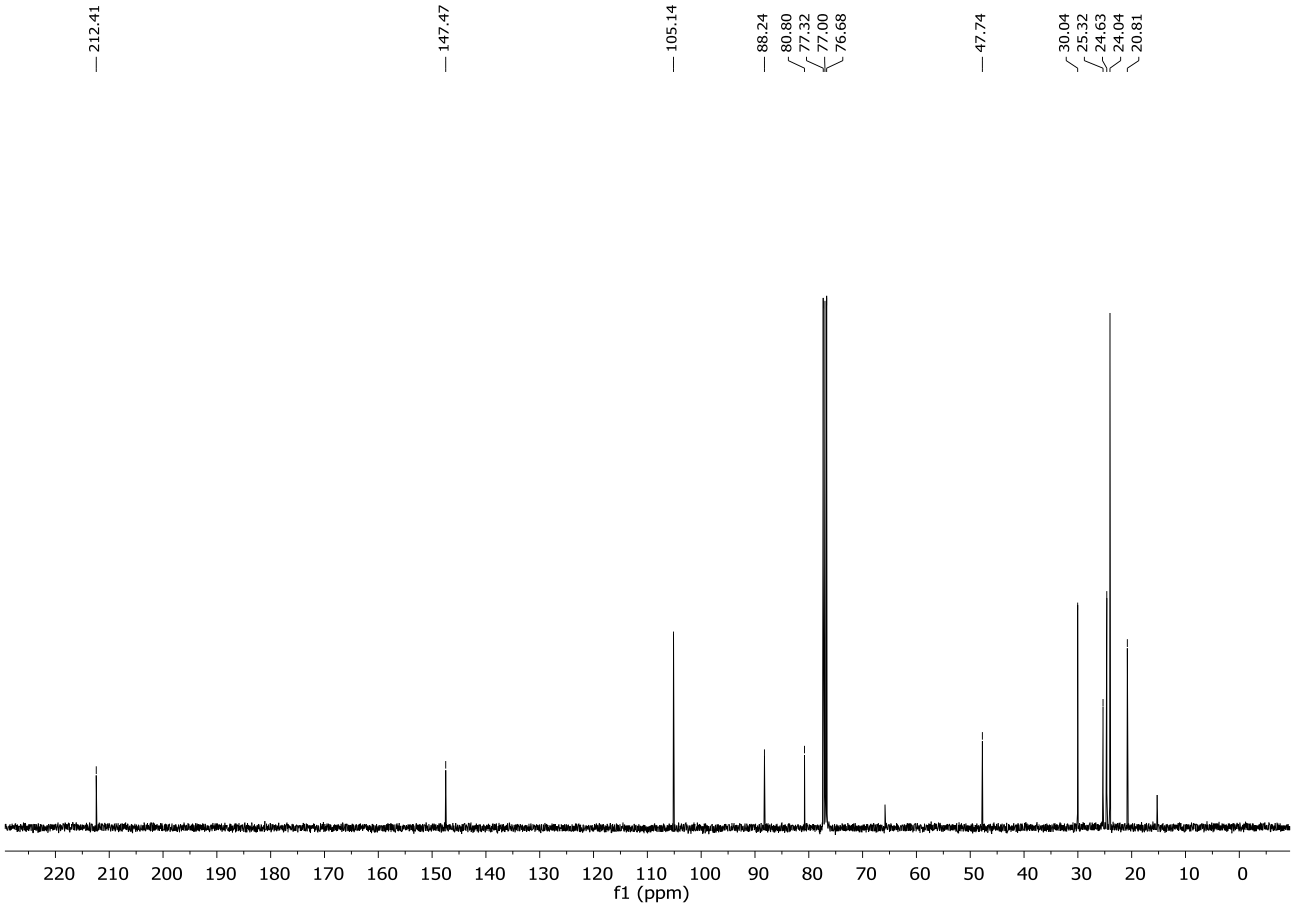
212.41
147.47
105.14
88.24
80.80
77.32
77.00
76.68
47.74
30.04
25.32
24.63
24.04
20.81
f1 (ppm)

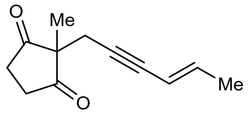
O
Me
Me
O

14

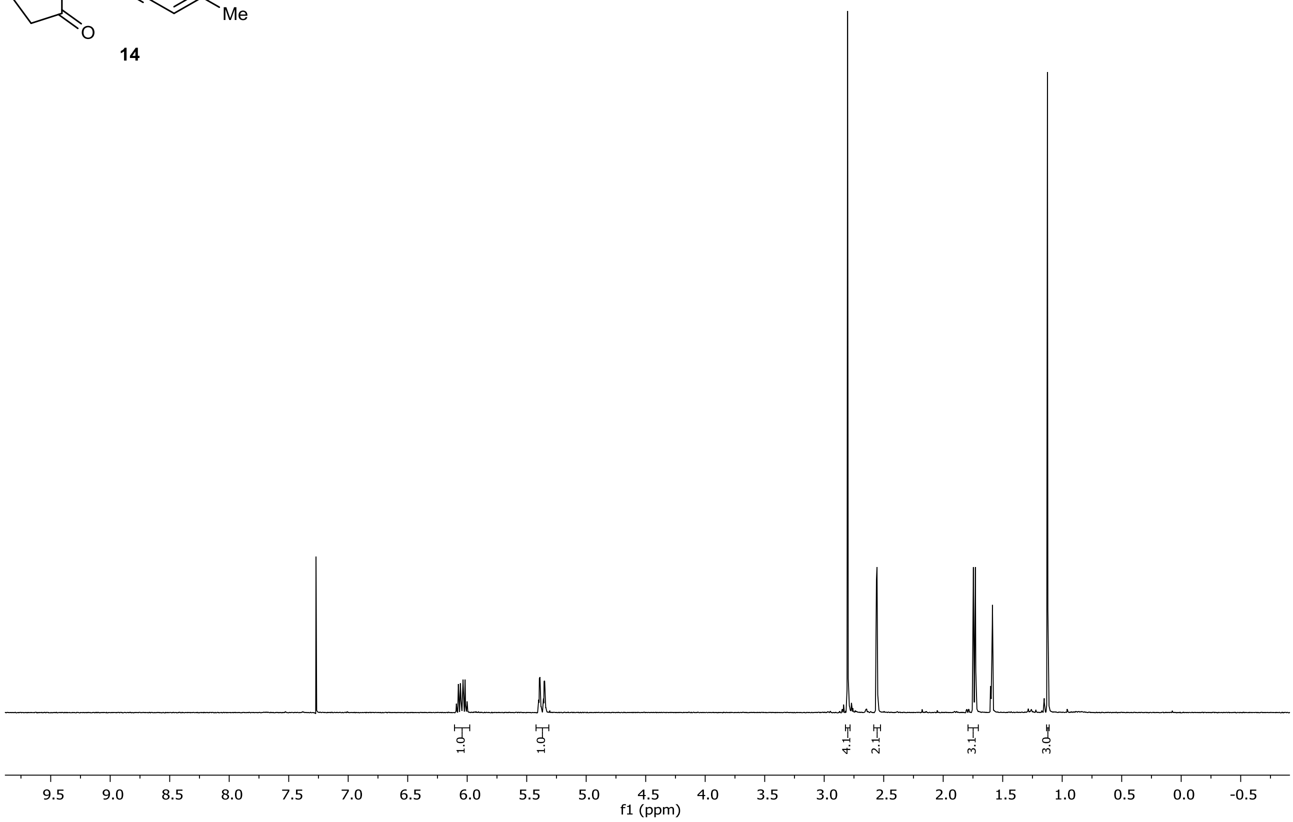
1.0
1.0
4.1
2.1
3.1
3.0
9.5 9.0 8.5 8.0 7.5 7.0 6.5 6.0 5.5 5.0 4.5 4.0 3.5 3.0 2.5 2.0 1.5 1.0 0.5 0.0 -0.5
f1 (ppm)

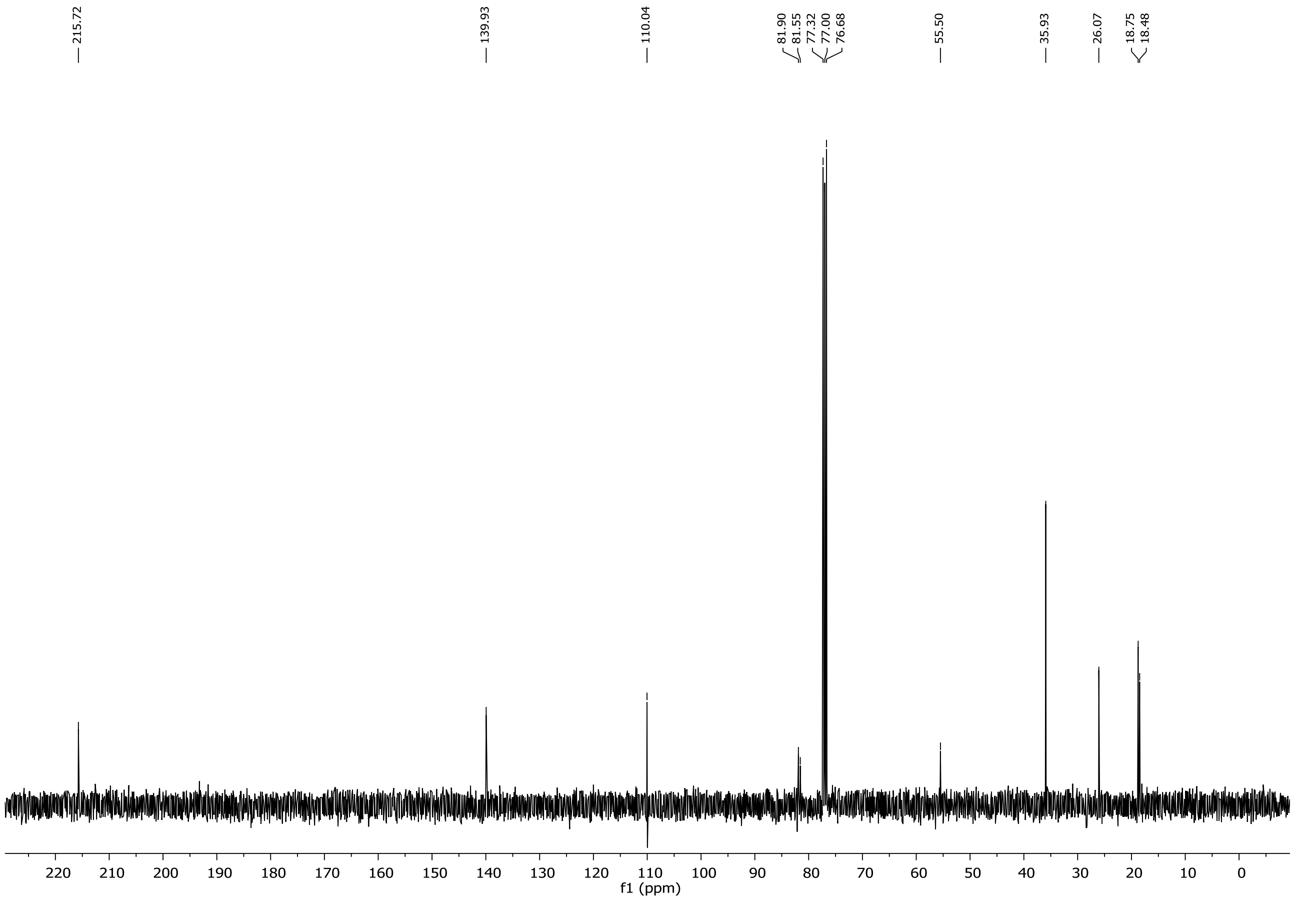
215.72
139.93
110.04
81.90
81.55
77.32
77.00
76.68
55.50
35.93
26.07
18.75
18.48
220 210 200 190 180 170 160 150 140 130 120 110 100 90 80 70 60 50 40 30 20 10 0
f1 (ppm)

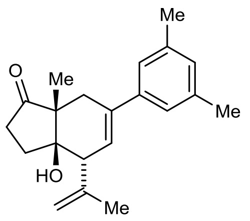
Me
O
Me
Me
HO
Me

2aa

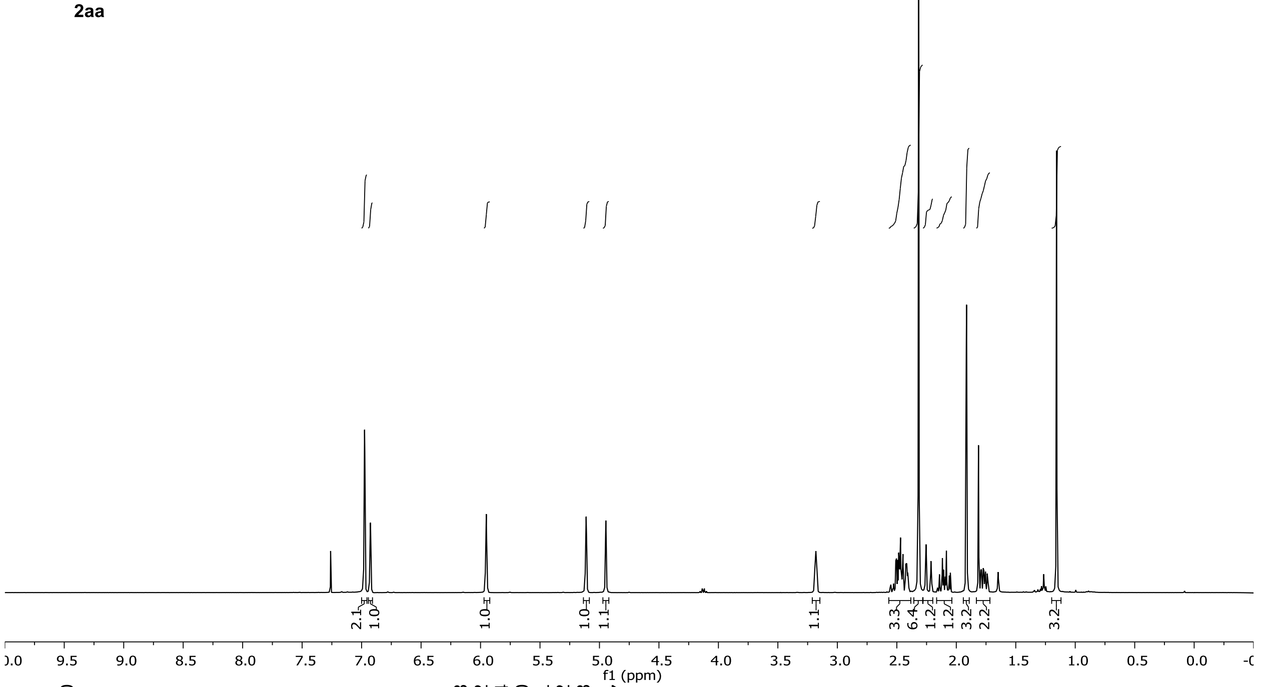
2.1
1.0
1.0
1.0
1.1
1.1
3.3
6.4
1.2
1.2
3.2
2.2
3.2
9.5
9.0
8.5
8.0
7.5
7.0
6.5
6.0
5.5
5.0
4.5
4.0
3.5
3.0
2.5
2.0
1.5
1.0
0.5
0.0
f1 (ppm)

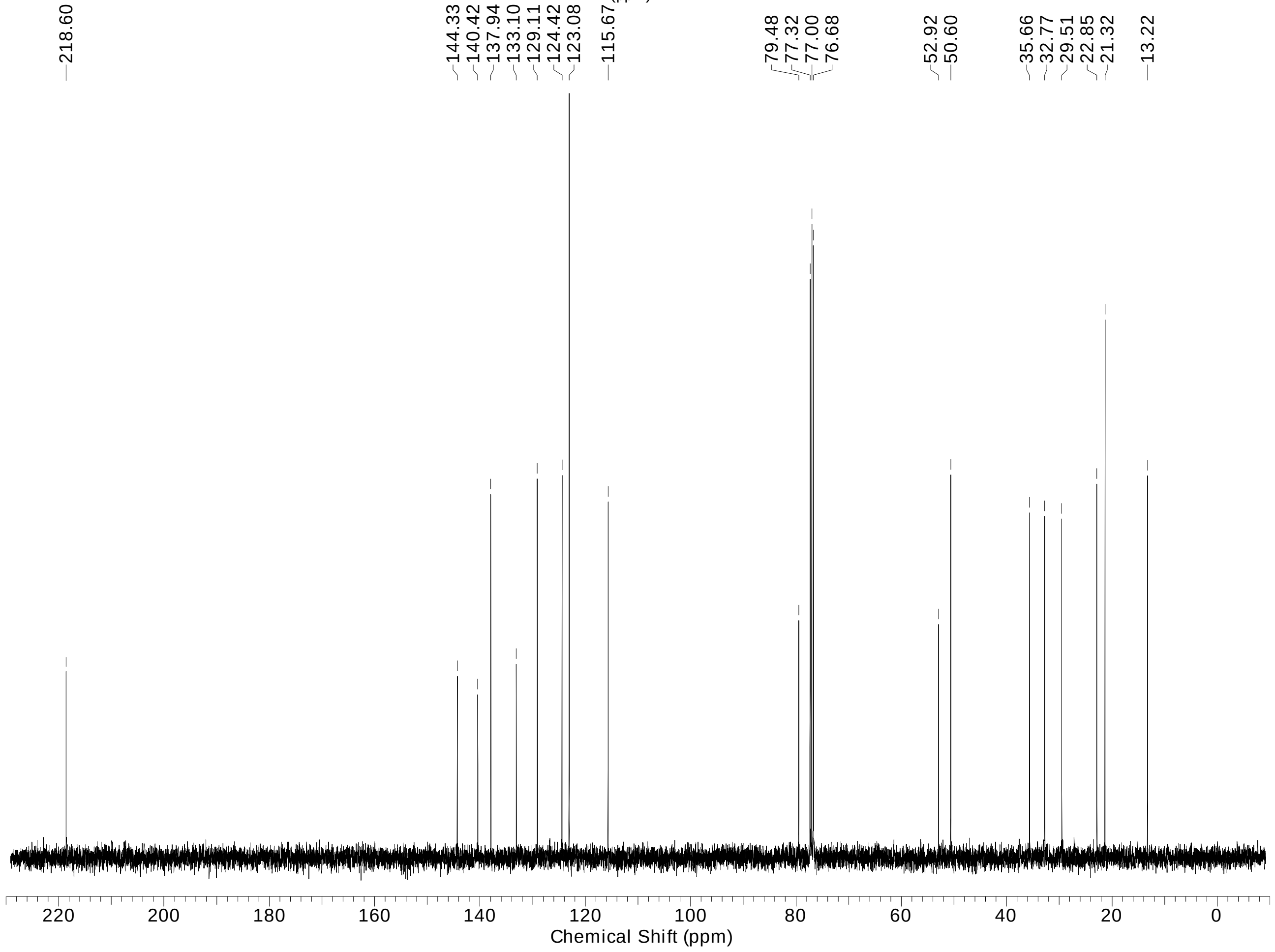
218.60
144.33
140.42
137.94
133.10
129.11
124.42
123.08
115.67
79.48
77.32
77.00
76.68
52.92
50.60
35.66
32.77
29.51
22.85
21.32
13.22
220
200
180
160
140
120
100
80
60
40
20
0
Chemical Shift (ppm)

HMBC

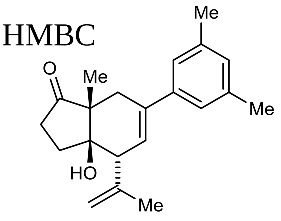

2aa

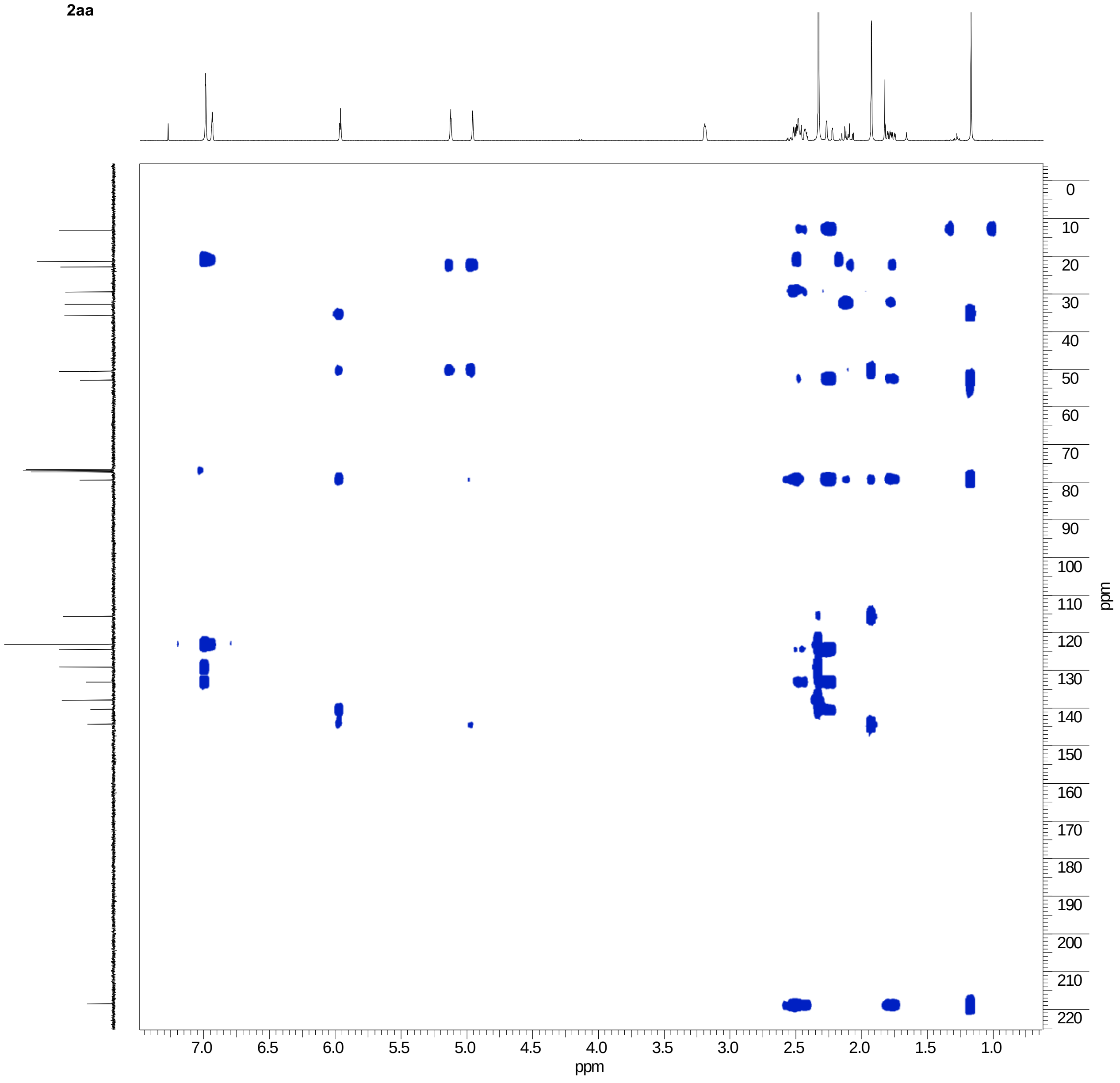

NOESY

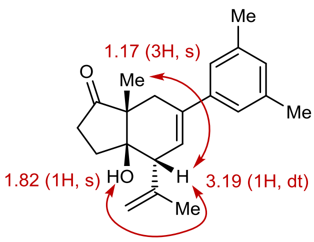

2aa

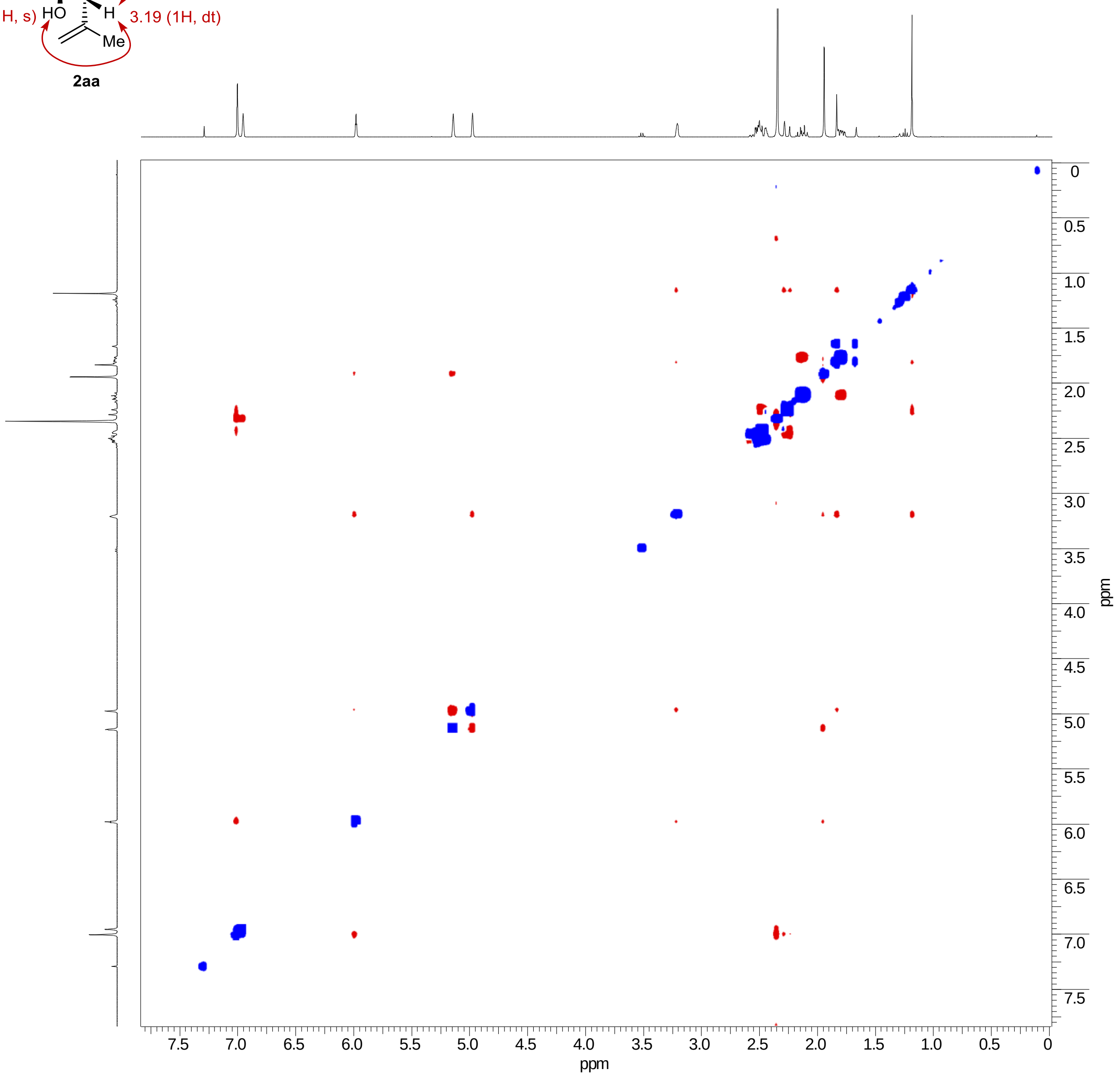

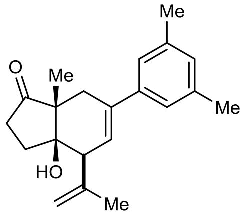
Me
O
Me
Me
HO
Me

2ab

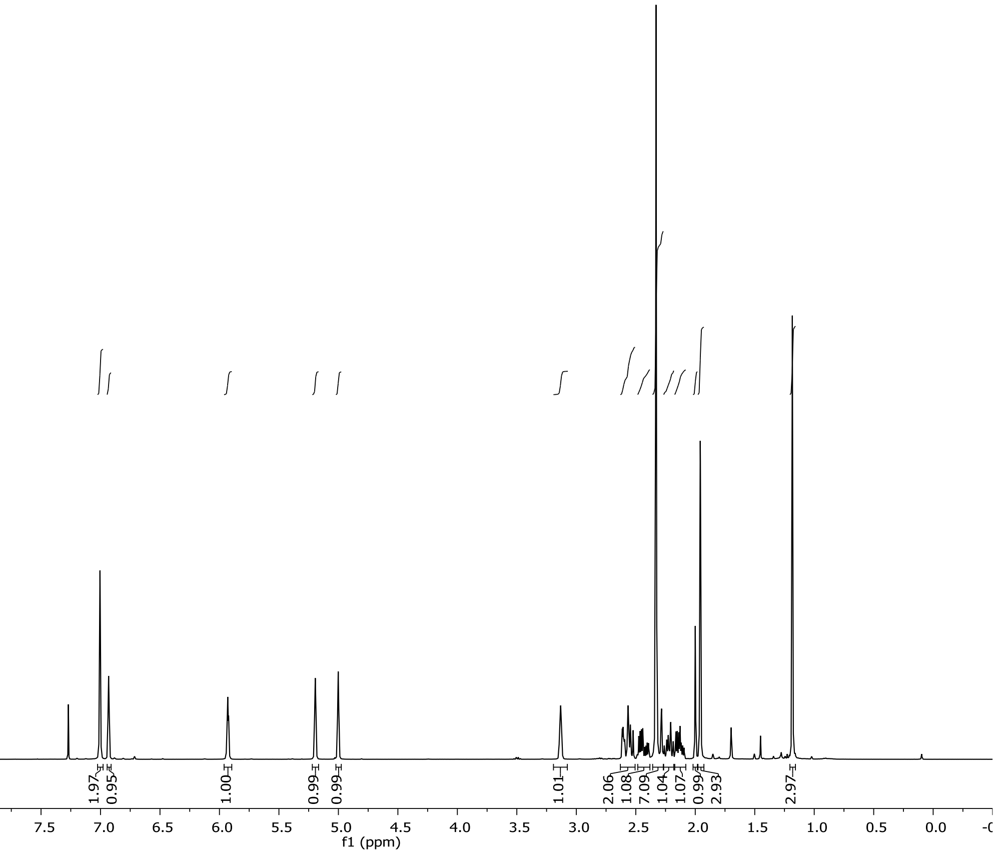
1.97
0.95
1.00
0.99
0.99
1.01
2.06
1.08
7.09
1.04
1.07
0.99
2.93
2.97
9.5 9.0 8.5 8.0 7.5 7.0 6.5 6.0 5.5 5.0 4.5 4.0 3.5 3.0 2.5 2.0 1.5 1.0 0.5 0.0
f1 (ppm)

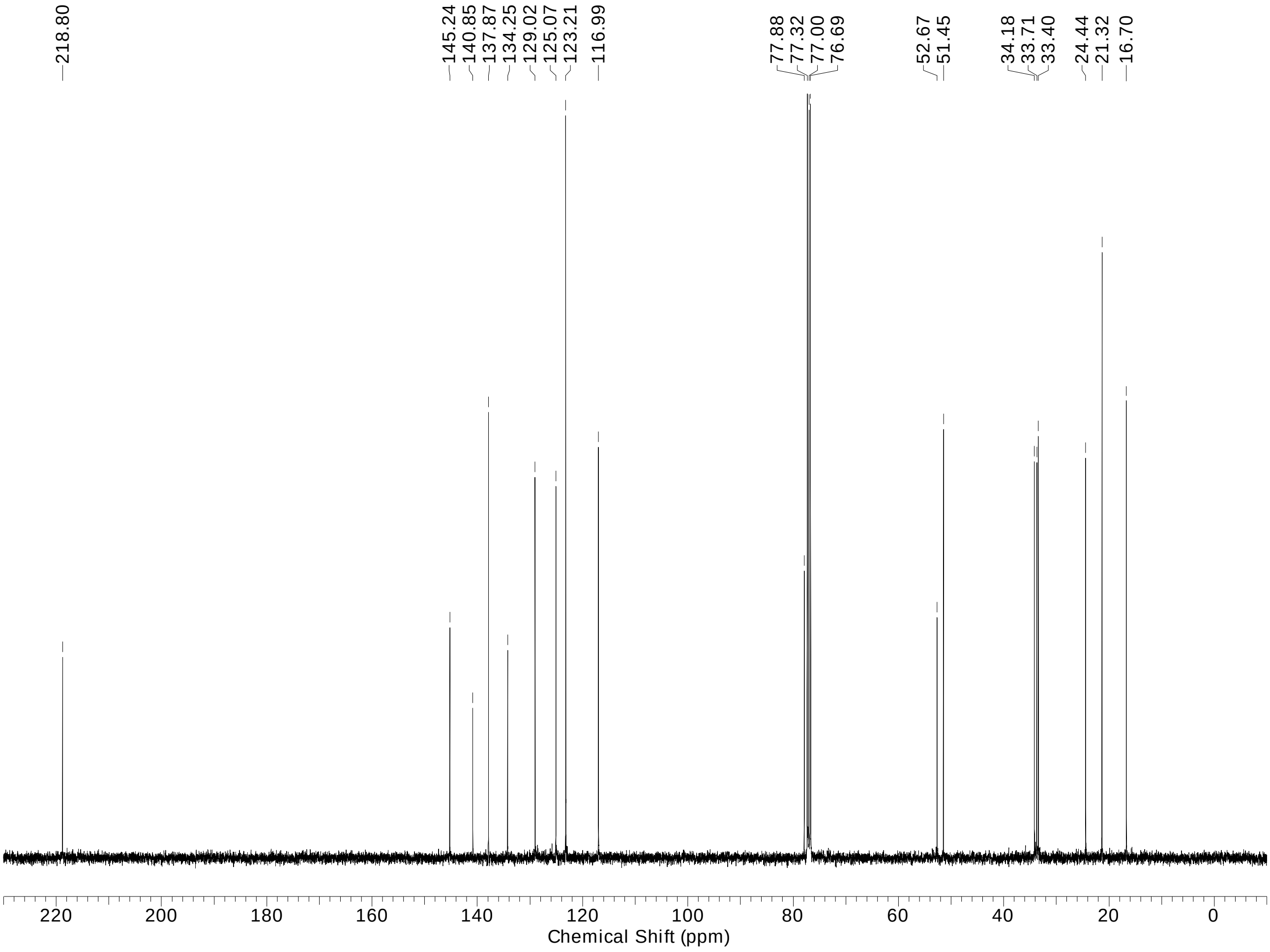
218.80
145.24
140.85
137.87
134.25
129.02
125.07
123.21
116.99
77.88
77.32
77.00
76.69
52.67
51.45
34.18
33.71
33.40
24.44
21.32
16.70
220 200 180 160 140 120 100 80 60 40 20 0
Chemical Shift (ppm)

NOESY

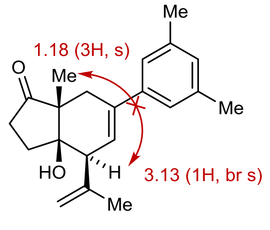

2ab

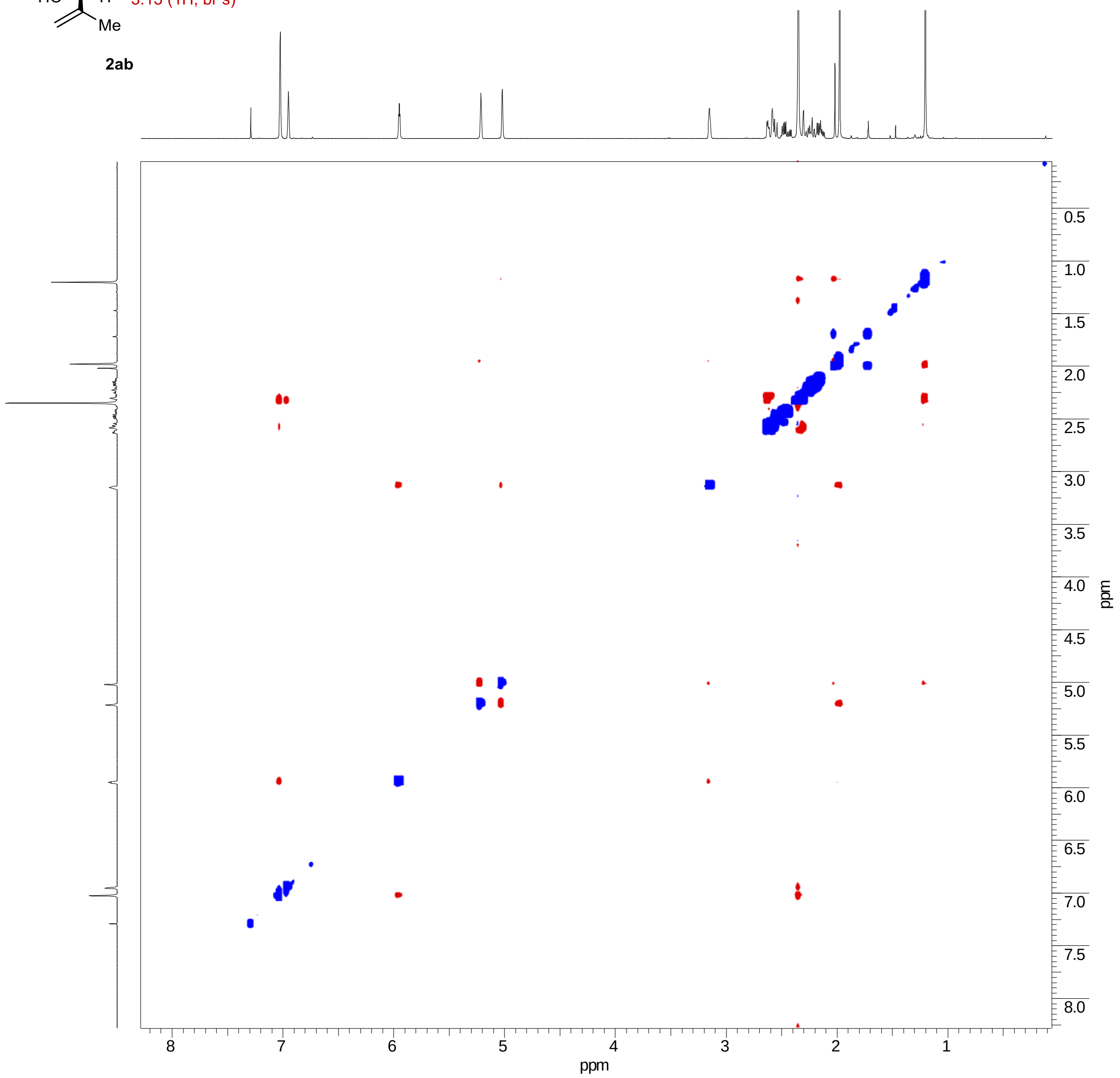

This NOESY NMR spectrum does not constitute strong proof of the relative configuration of **2ab**. The relative configuration of **2a** was assigned tentatively by analogy with **9cb** (NOESY NMR spectrum on page 82), the relative configuration of which was determined by X-ray crystallography (see page 21).

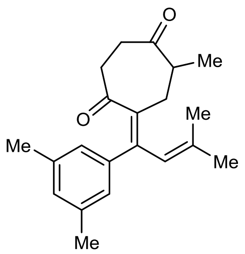
O
Me
O
Me
Me
Me
Me

3a

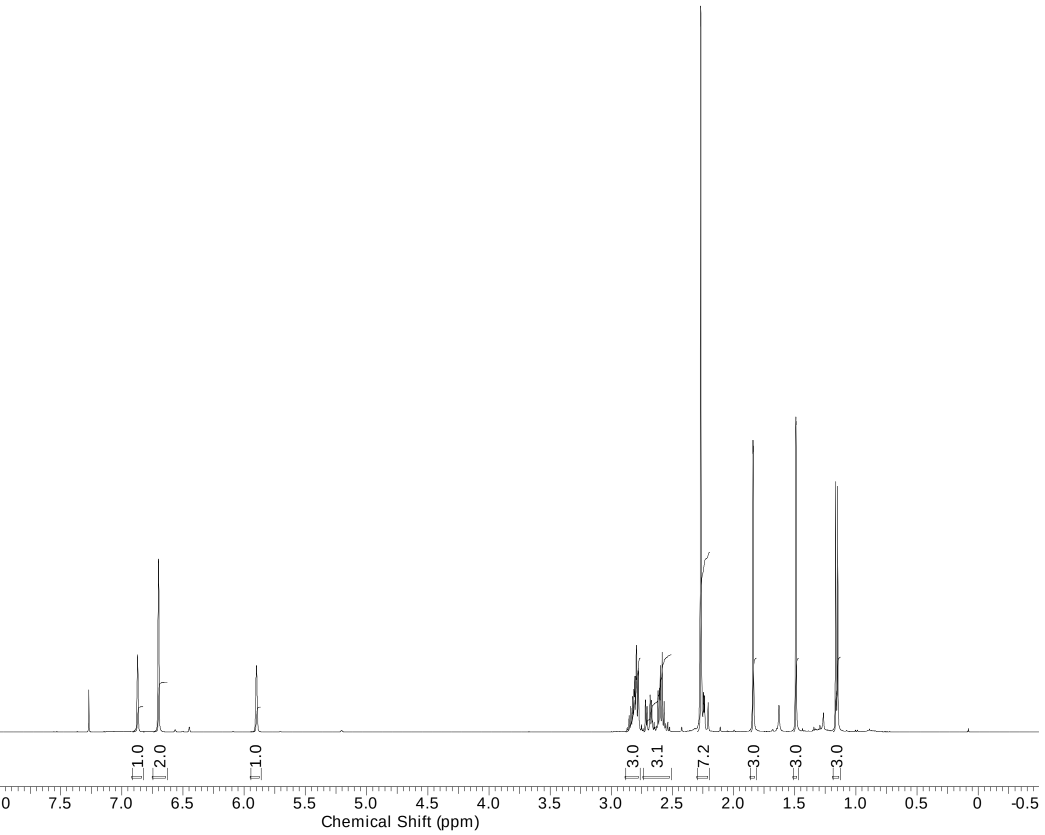
1.0
2.0
1.0
3.0
3.1
7.2
3.0
3.0
3.0
9.5 9.0 8.5 8.0 7.5 7.0 6.5 6.0 5.5 5.0 4.5 4.0 3.5 3.0 2.5 2.0 1.5 1.0 0.5 0 -0.5
Chemical Shift (ppm)

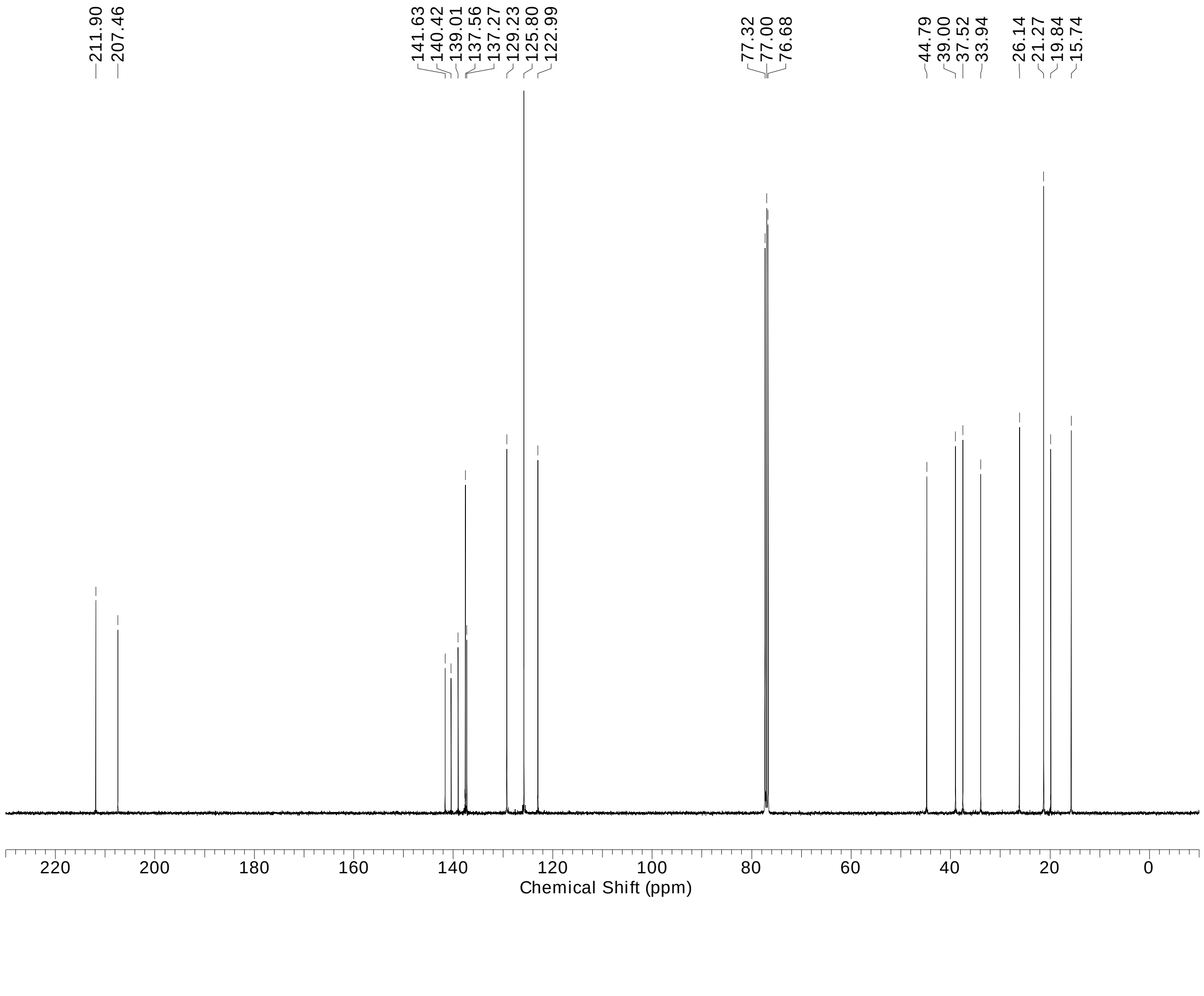
211.90
207.46
141.63
140.42
139.01
137.56
137.27
129.23
125.80
122.99
77.32
77.00
76.68
44.79
39.00
37.52
33.94
26.14
21.27
19.84
15.74
220 200 180 160 140 120 100 80 60 40 20 0
Chemical Shift (ppm)

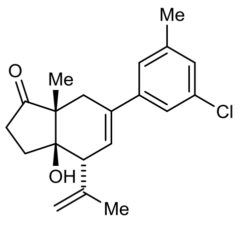
Me
O
Me
Cl
OH
Me

2ba

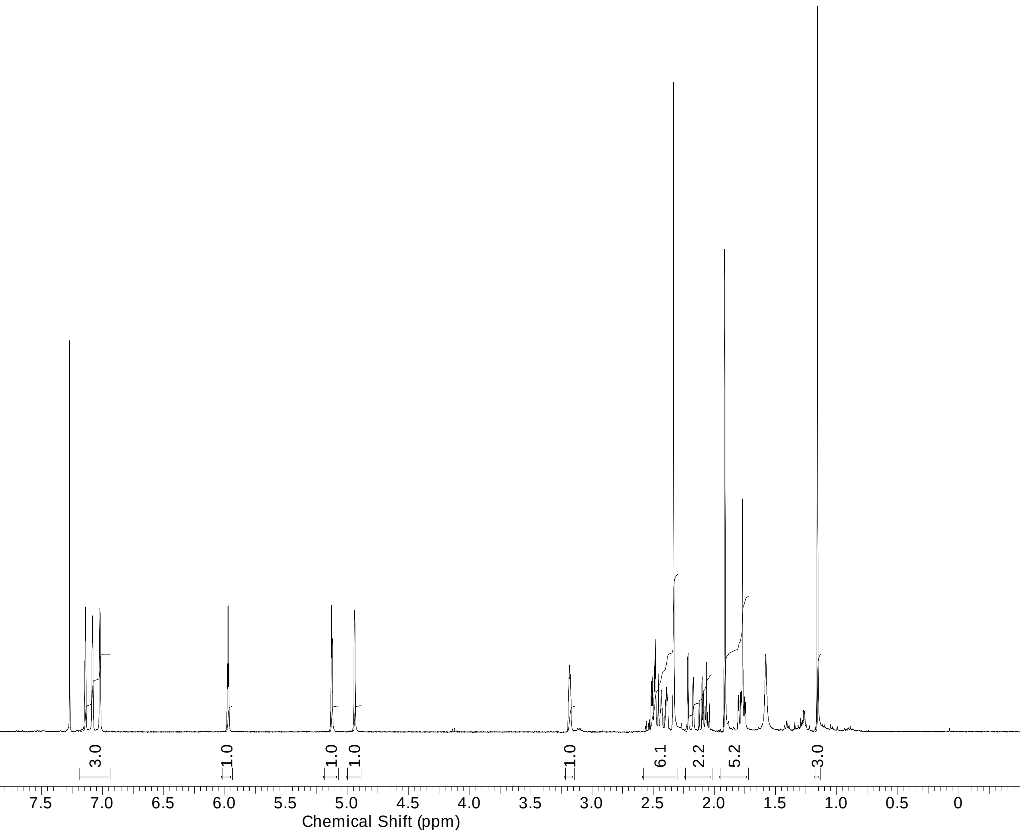
3.0
1.0
1.0
1.0
1.0
6.1
2.2
5.2
3.0
9.5
9.0
8.5
8.0
7.5
7.0
6.5
6.0
5.5
5.0
4.5
4.0
3.5
3.0
2.5
2.0
1.5
1.0
0.5
0
Chemical Shift (ppm)

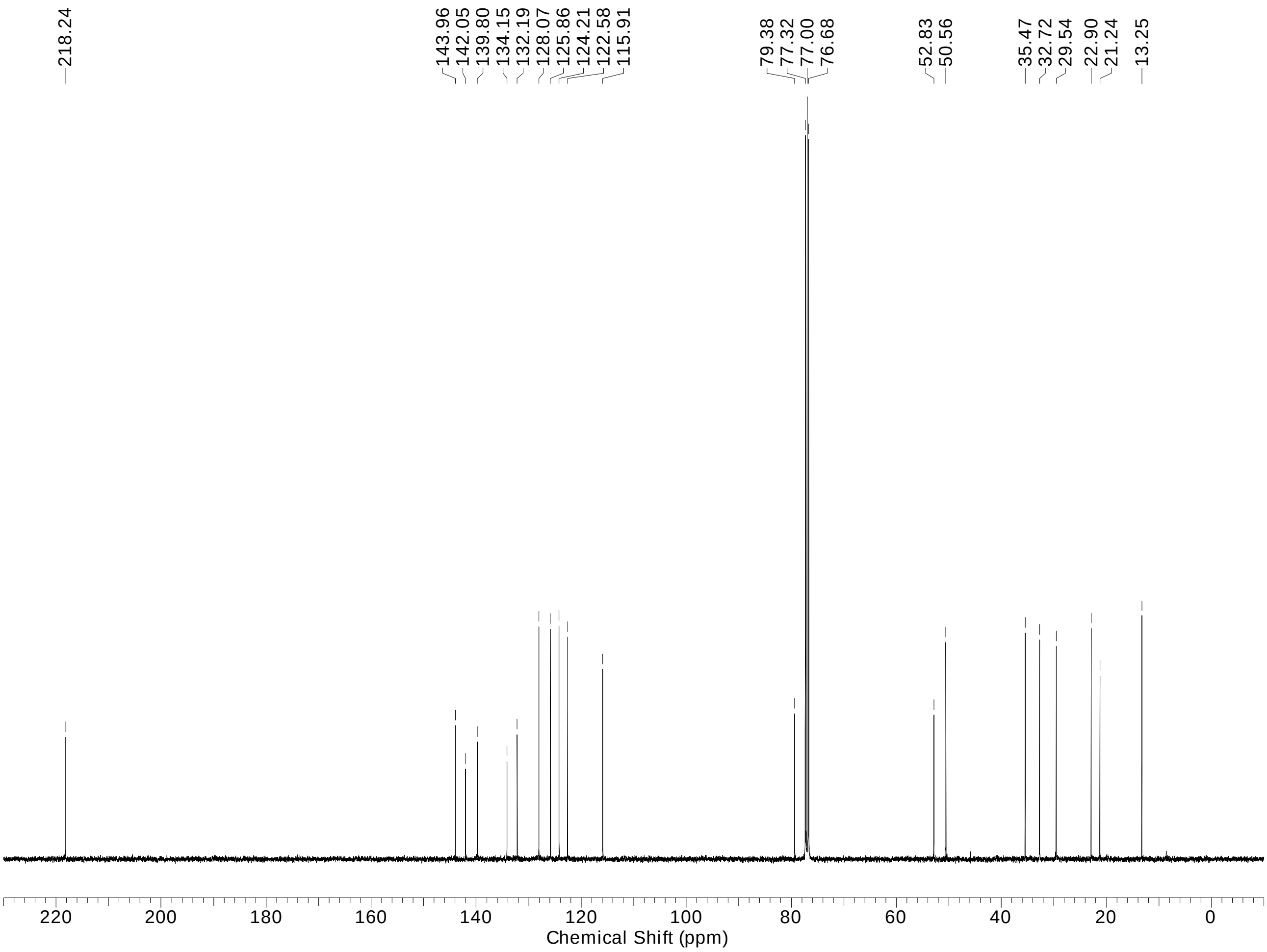
218.24
143.96
142.05
139.80
134.15
132.19
128.07
125.86
124.21
122.58
115.91
79.38
77.32
77.00
76.68
52.83
50.56
35.47
32.72
29.54
22.90
21.24
13.25
220
200
180
160
140
120
100
80
60
40
20
0
Chemical Shift (ppm)

NOESY

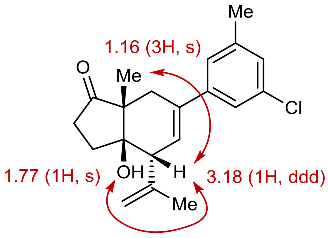

2ba

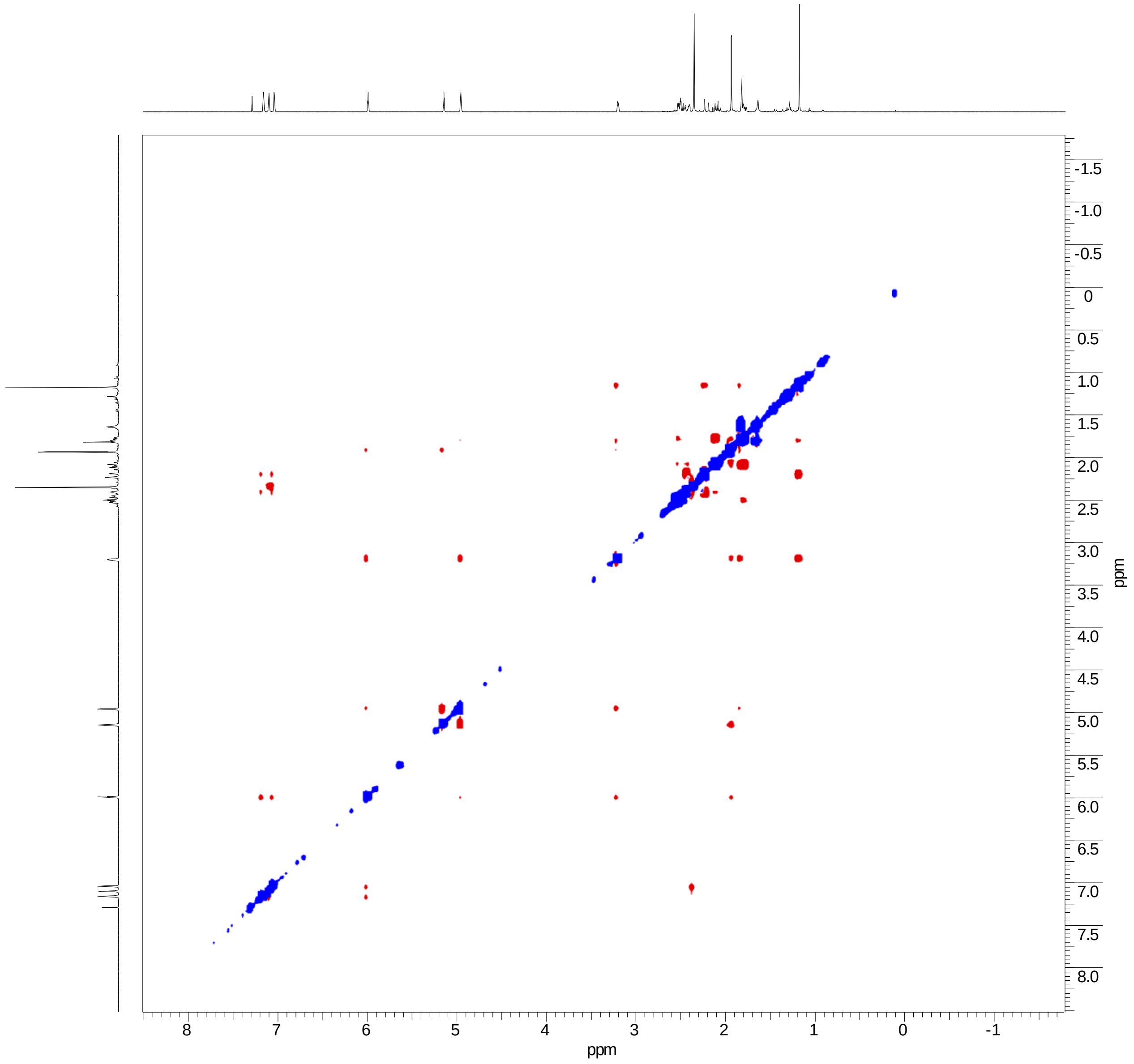

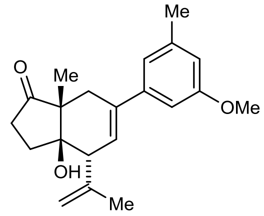
Me
O
Me
OMe
OH
Me

2ca

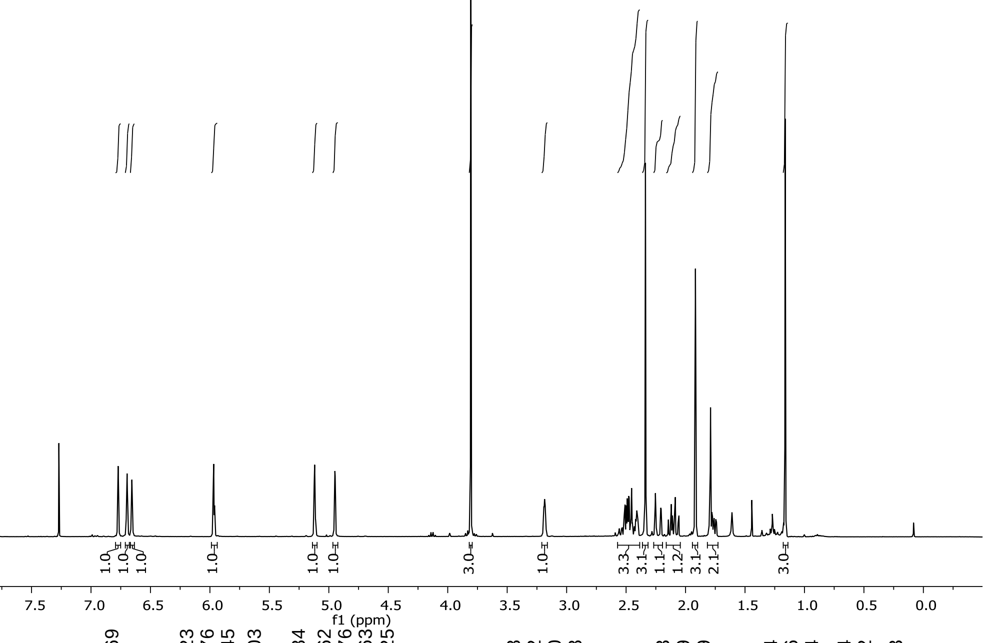
1.0
1.0
1.0
1.0
1.0
1.0
3.0
1.0
3.3
3.1
1.1
1.2
3.1
2.1
3.0
f1 (ppm)

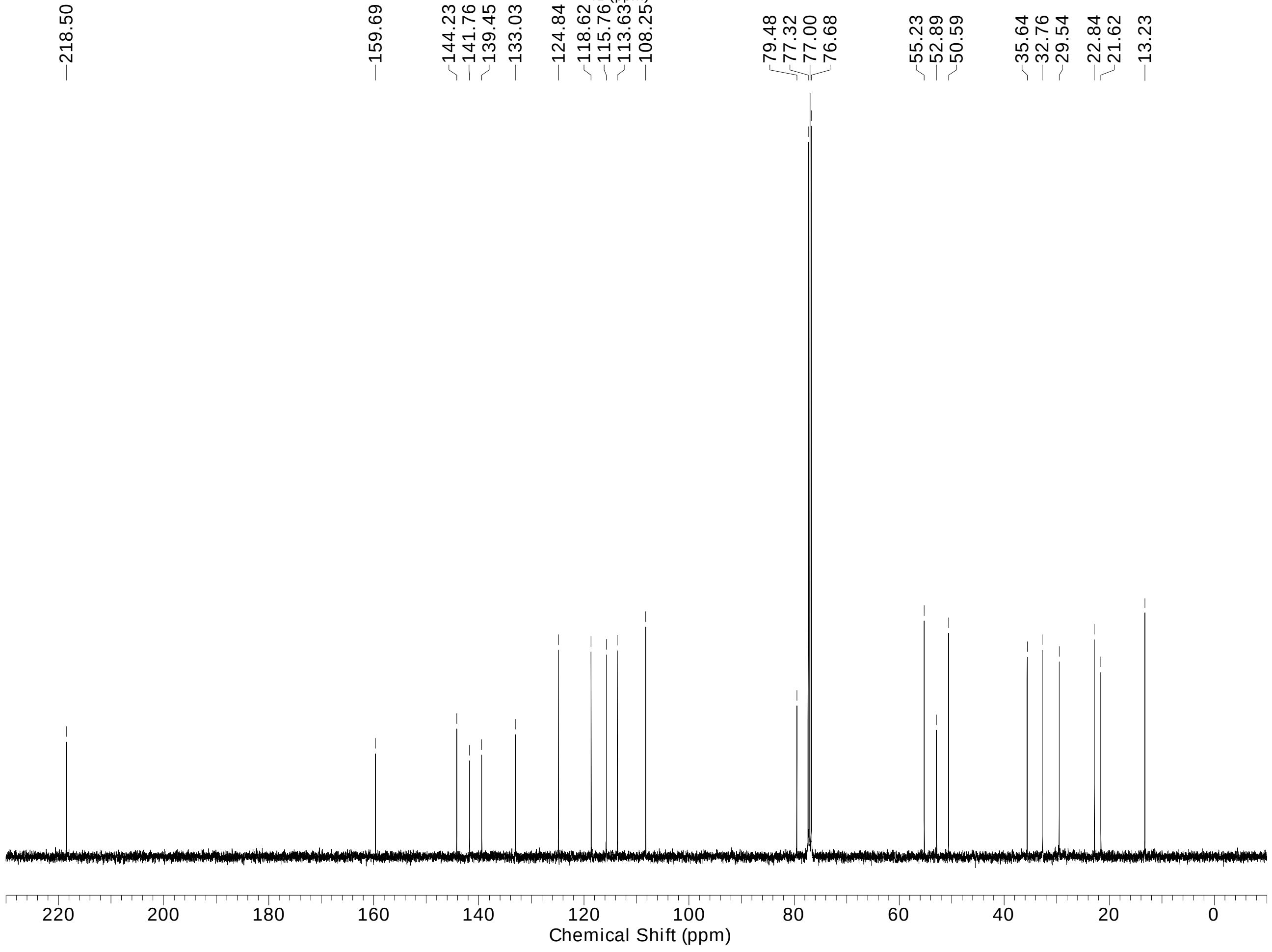
218.50
159.69
144.23
141.76
139.45
133.03
124.84
118.62
115.76
113.63
108.25
79.48
77.32
77.00
76.68
55.23
52.89
50.59
35.64
32.76
29.54
22.84
21.62
13.23
Chemical Shift (ppm)

NOESY

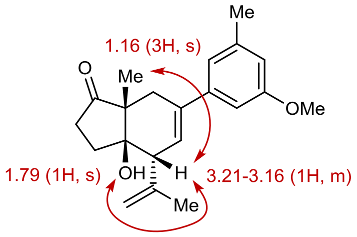
Me
1.16 (3H, s)
O
Me
OMe
1.79 (1H, s)
OH
H
3.21-3.16 (1H, m)
Me

2ca

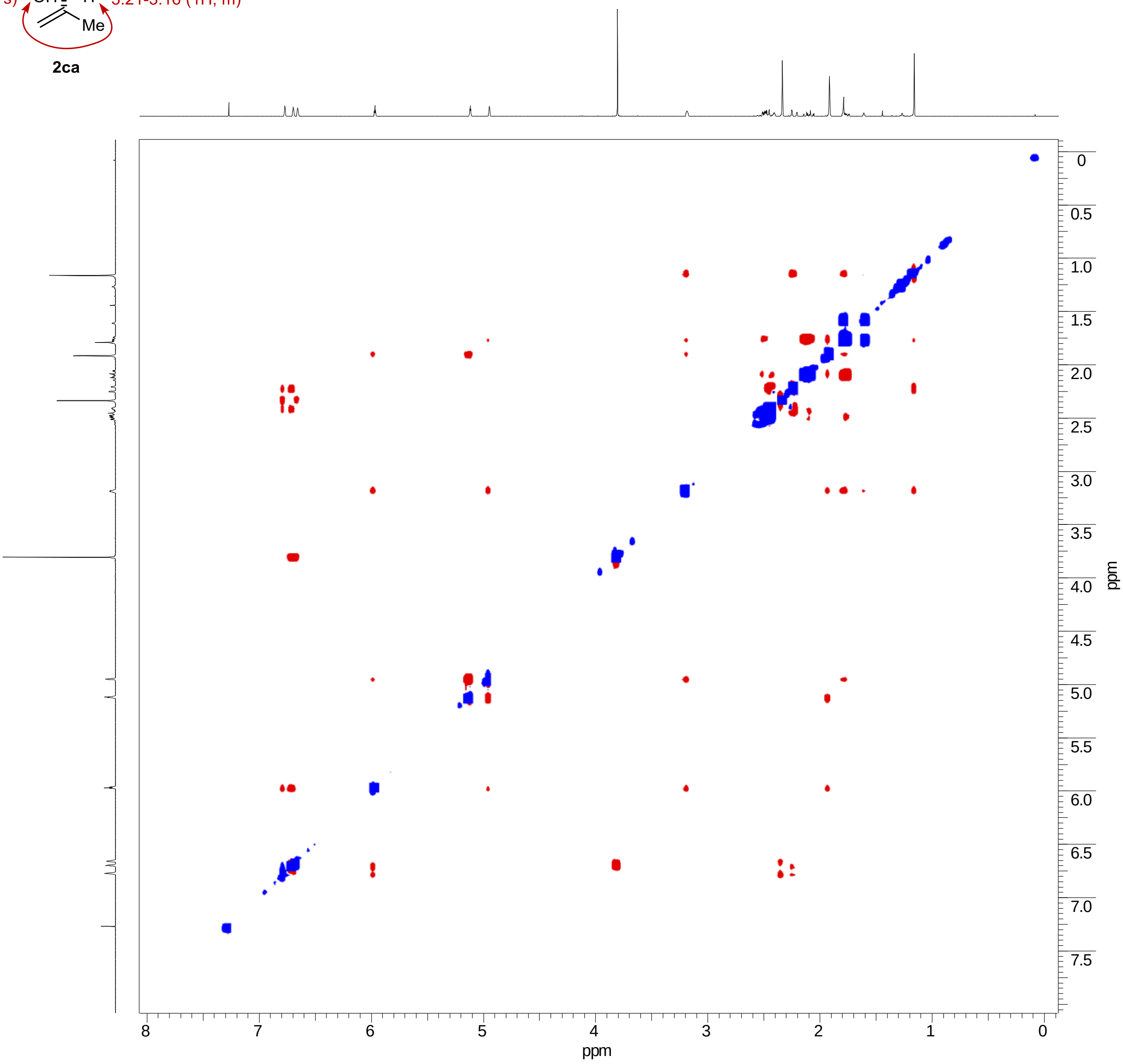
ppm
8
7
6
5
4
3
2
1
0
0.5
1.0
1.5
2.0
2.5
3.0
3.5
4.0
4.5
5.0
5.5
6.0
6.5
7.0
7.5

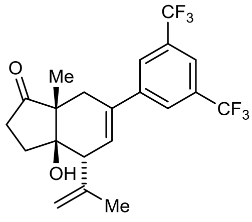
CF3
O
Me
CF3
OH
Me

2da

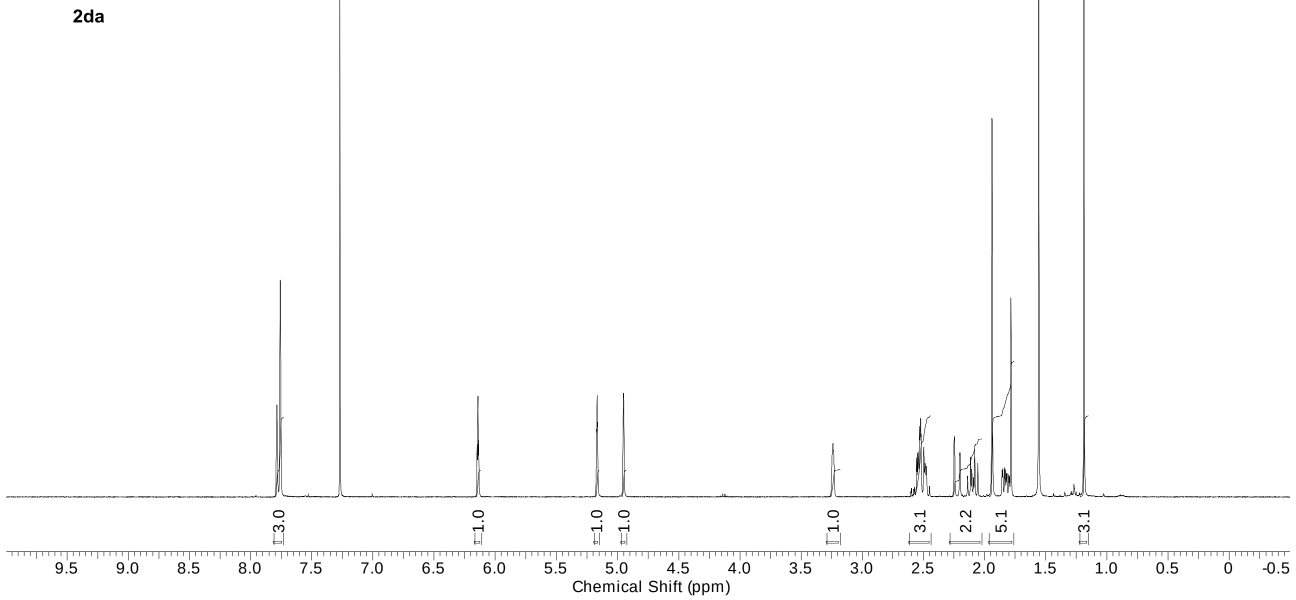
3.0
1.0
1.0
1.0
1.0
3.1
2.2
5.1
3.1
9.5 9.0 8.5 8.0 7.5 7.0 6.5 6.0 5.5 5.0 4.5 4.0 3.5 3.0 2.5 2.0 1.5 1.0 0.5 0 -0.5
Chemical Shift (ppm)

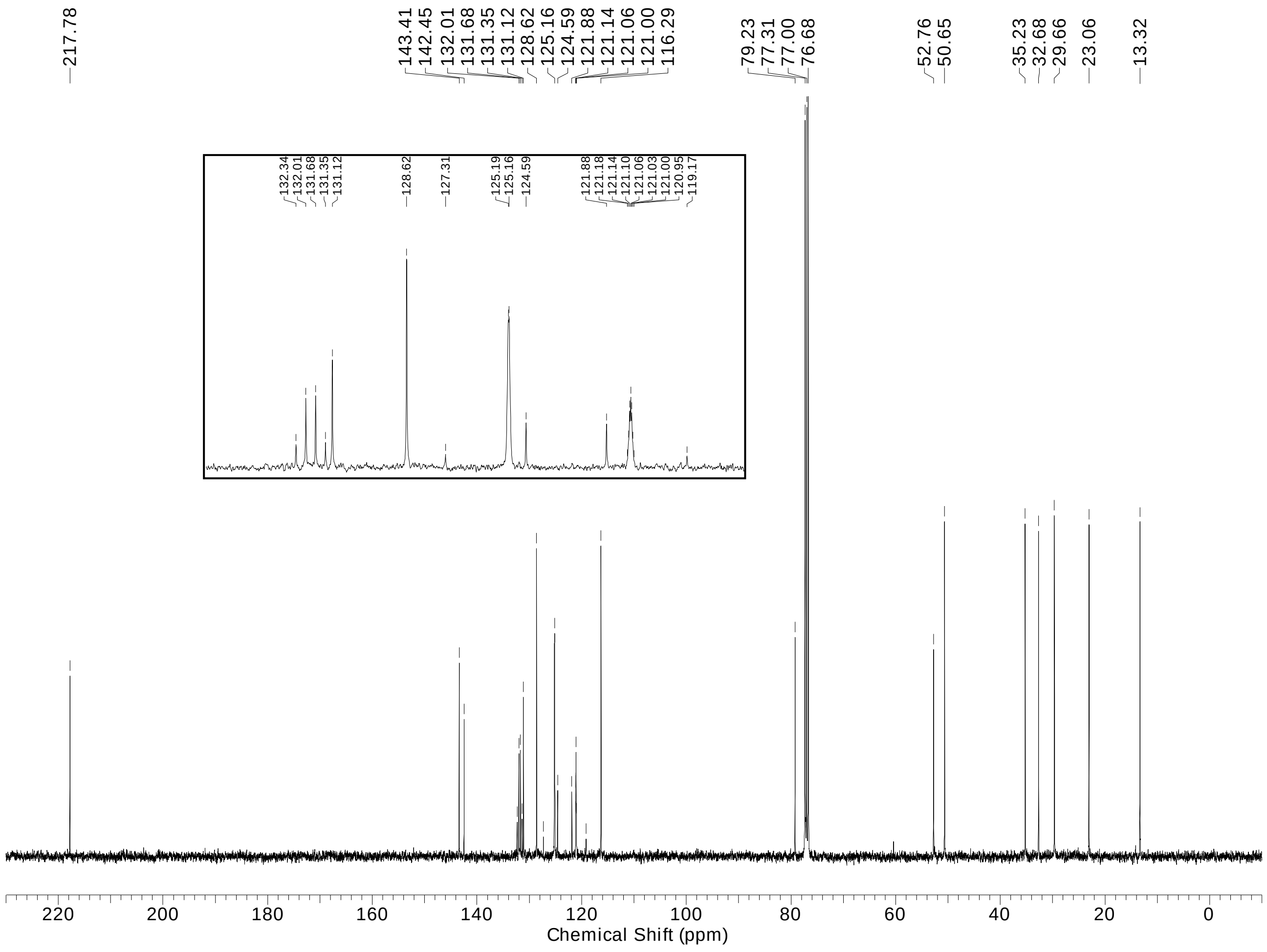
217.78
143.41
142.45
132.01
131.68
131.35
131.12
128.62
125.16
124.59
121.88
121.14
121.06
121.00
116.29
79.23
77.31
77.00
76.68
52.76
50.65
35.23
32.68
29.66
23.06
13.32
132.34
132.01
131.68
131.35
131.12
128.62
127.31
125.19
125.16
124.59
121.88
121.18
121.14
121.10
121.06
121.03
121.00
120.95
119.17
220 200 180 160 140 120 100 80 60 40 20 0
Chemical Shift (ppm)

^{19}F NMR Spectrum

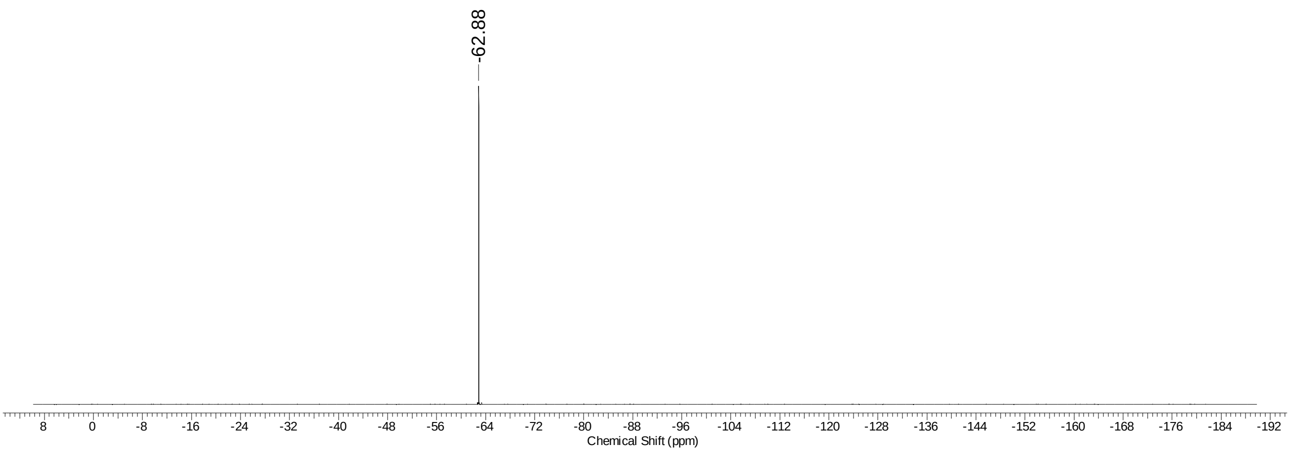

NOESY

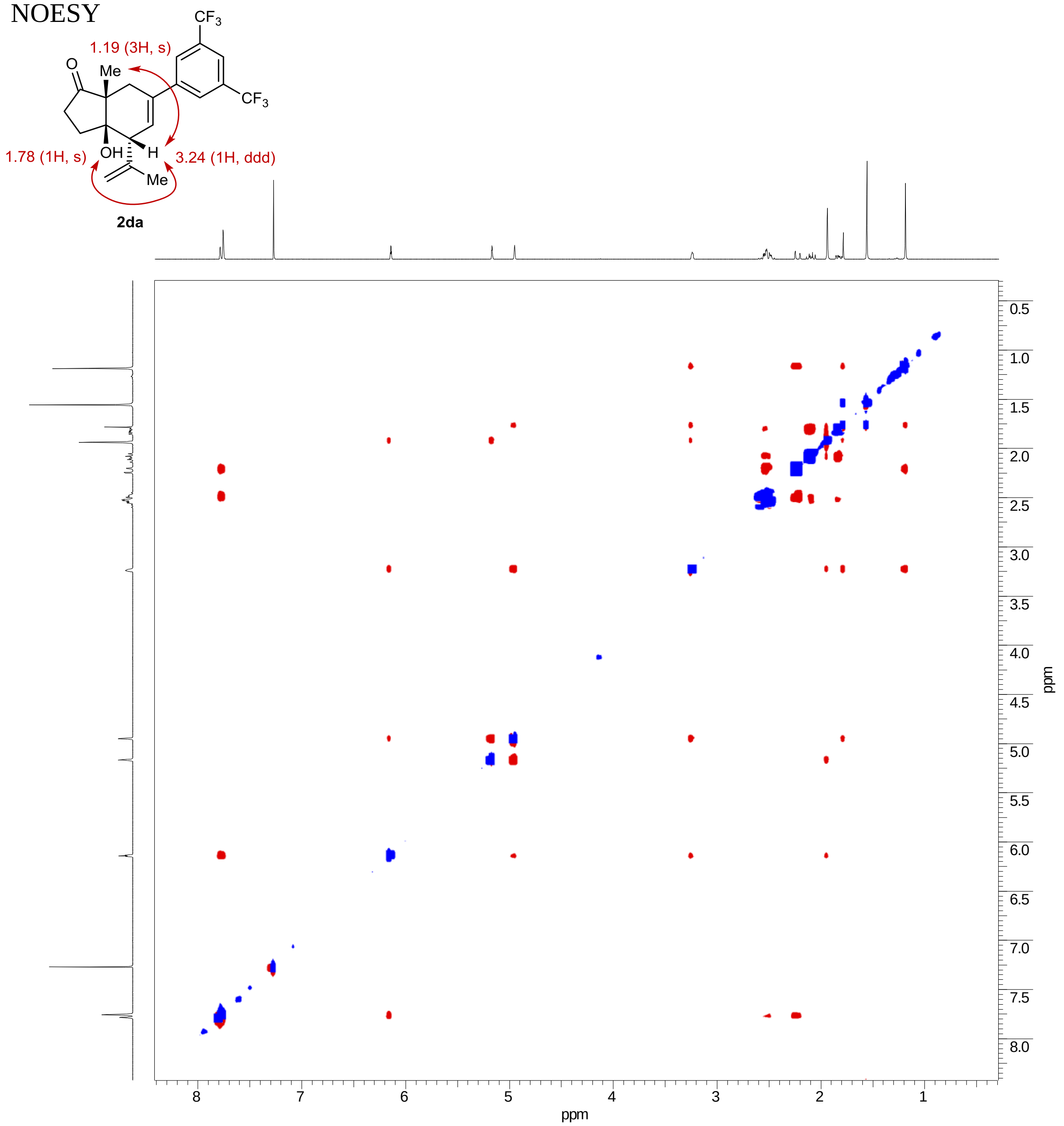

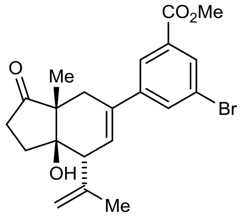
CO2Me
O
Me
Br
OH
Me

2ea

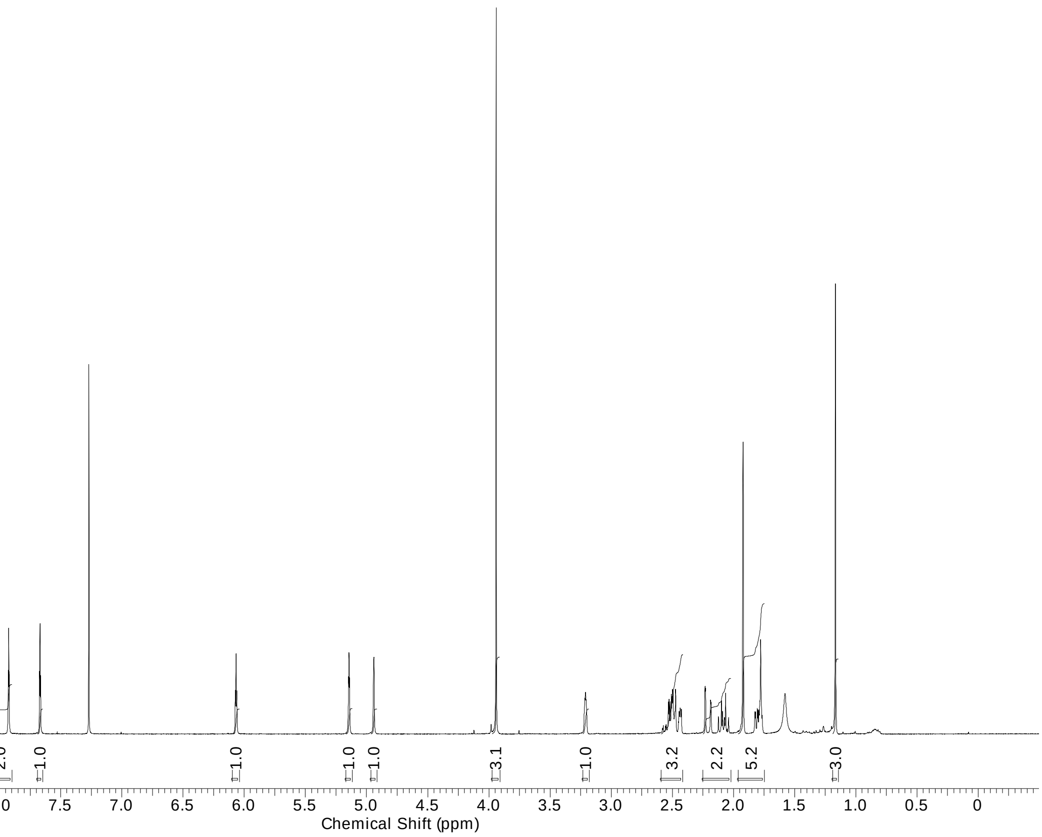
2.0
1.0
1.0
1.0
1.0
3.1
1.0
3.2
2.2
5.2
3.0
9.5
9.0
8.5
8.0
7.5
7.0
6.5
6.0
5.5
5.0
4.5
4.0
3.5
3.0
2.5
2.0
1.5
1.0
0.5
0
Chemical Shift (ppm)

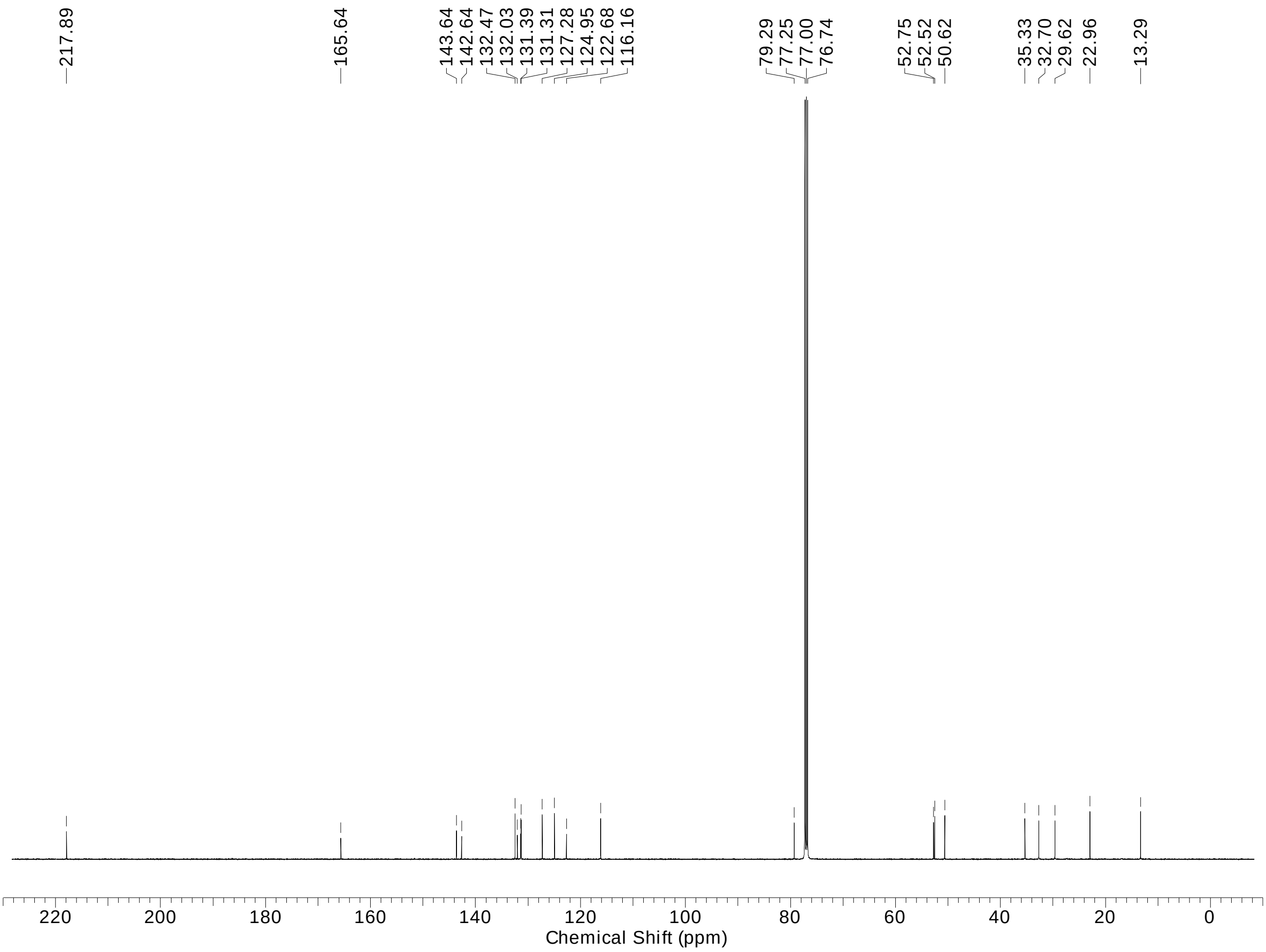
217.89
165.64
143.64
142.64
132.47
132.03
131.39
131.31
127.28
124.95
122.68
116.16
79.29
77.25
77.00
76.74
52.75
52.52
50.62
35.33
32.70
29.62
22.96
13.29
220
200
180
160
140
120
100
80
60
40
20
0
Chemical Shift (ppm)

NOESY

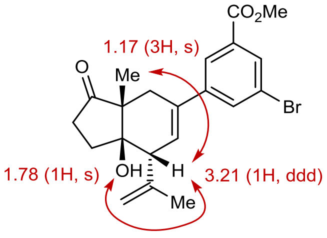

2ea

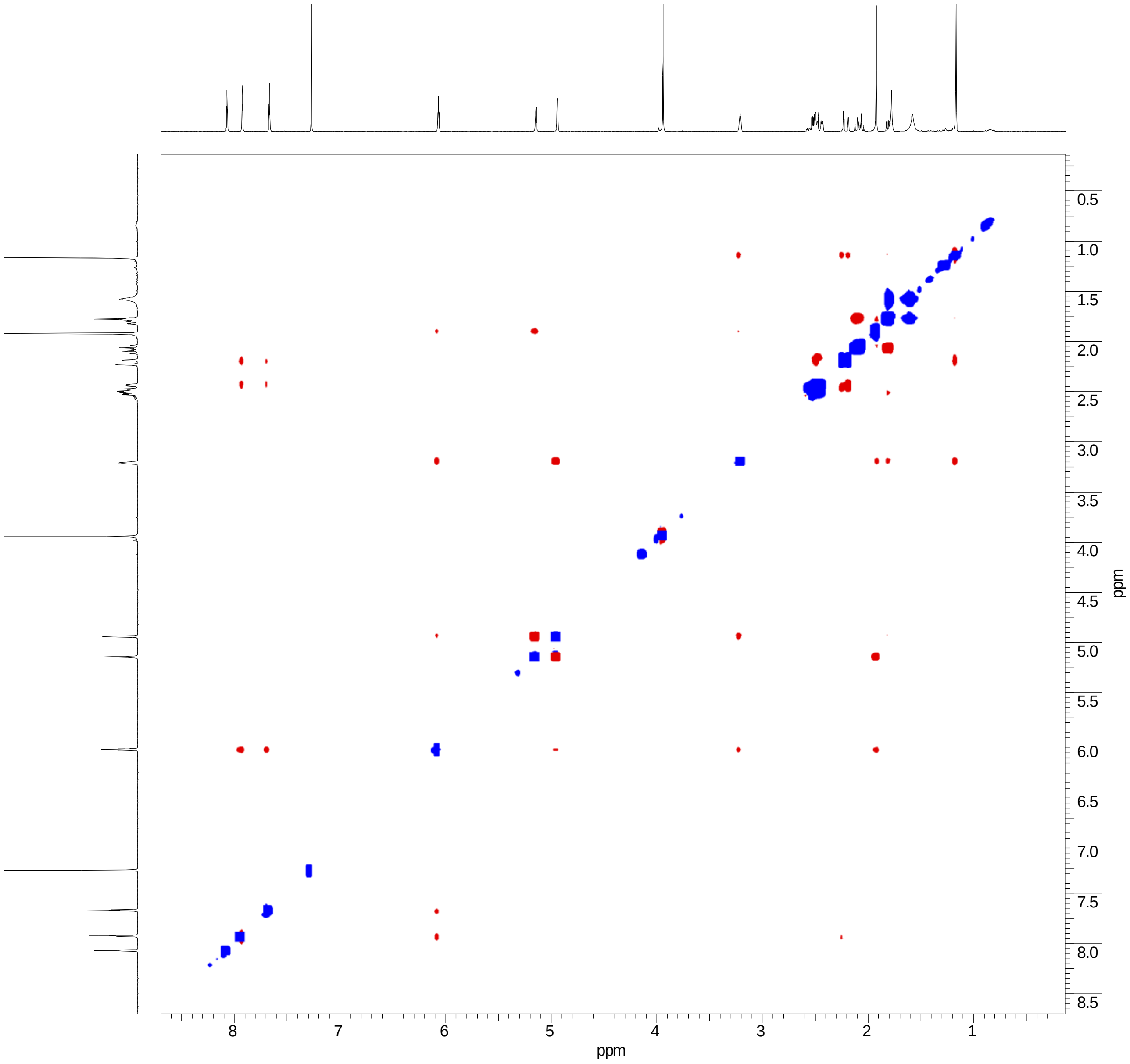

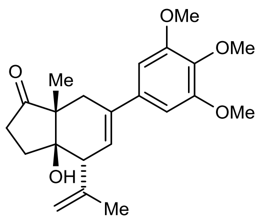
OMe
OMe
OMe
O
Me
OH
Me

2fa

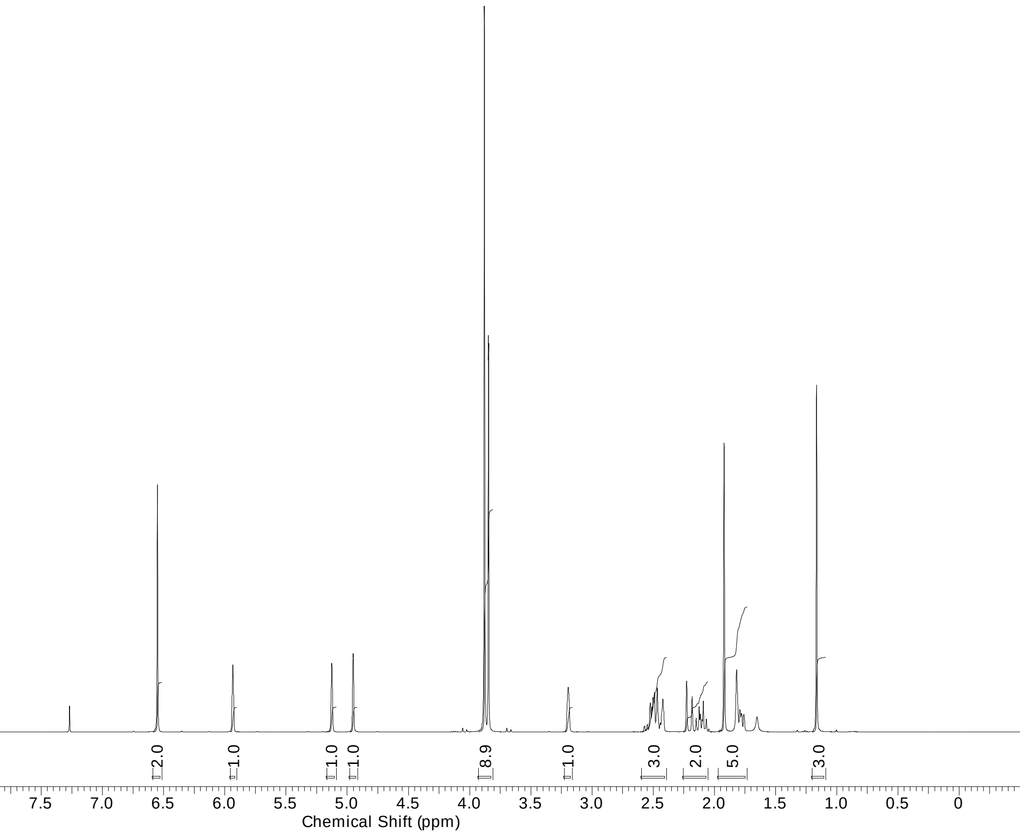
2.0
1.0
1.0
1.0
8.9
1.0
3.0
2.0
5.0
3.0
9.5 9.0 8.5 8.0 7.5 7.0 6.5 6.0 5.5 5.0 4.5 4.0 3.5 3.0 2.5 2.0 1.5 1.0 0.5 0
Chemical Shift (ppm)

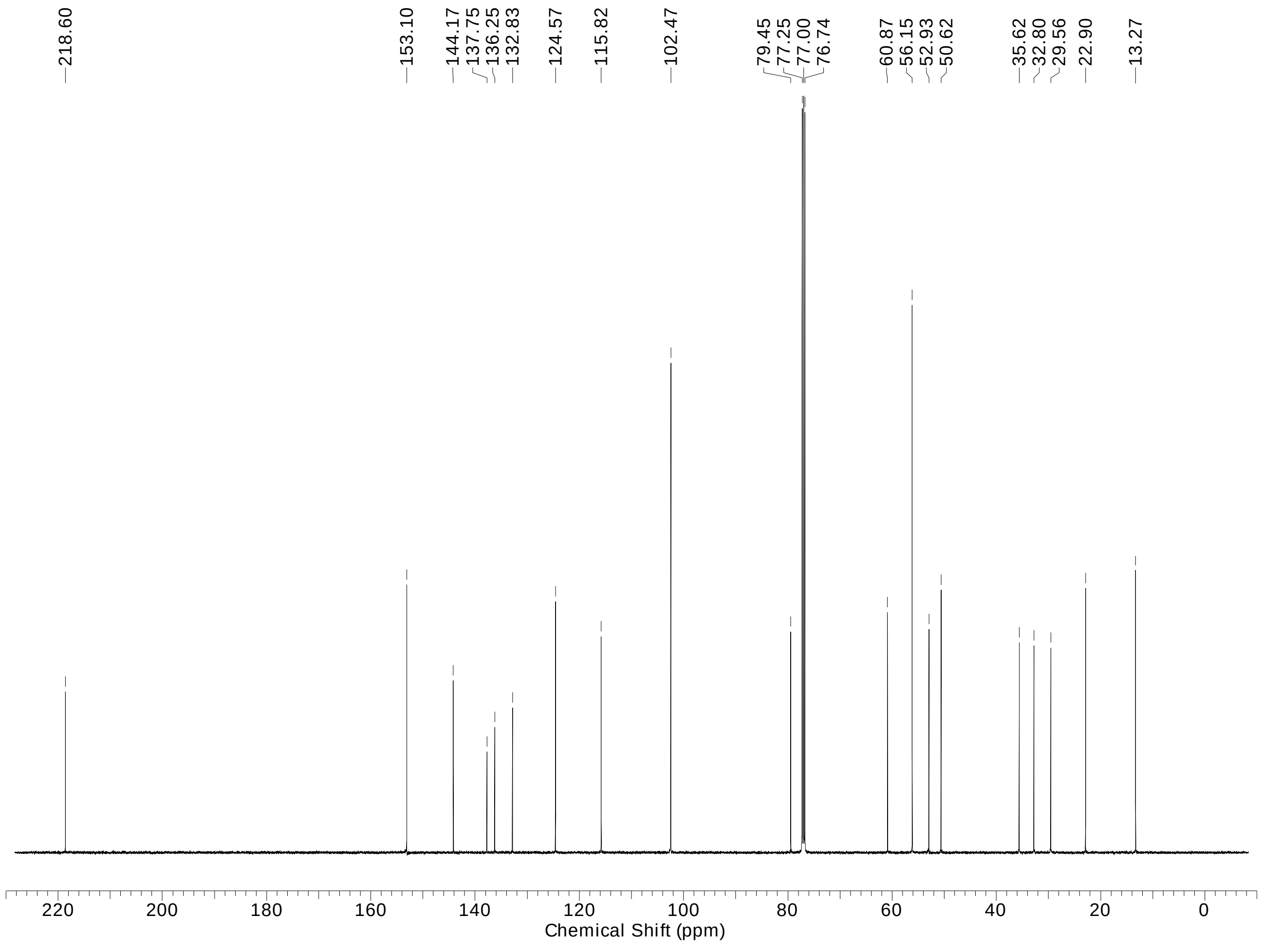
218.60
153.10
144.17
137.75
136.25
132.83
124.57
115.82
102.47
79.45
77.25
77.00
76.74
60.87
56.15
52.93
50.62
35.62
32.80
29.56
22.90
13.27
220 200 180 160 140 120 100 80 60 40 20 0
Chemical Shift (ppm)

NOESY

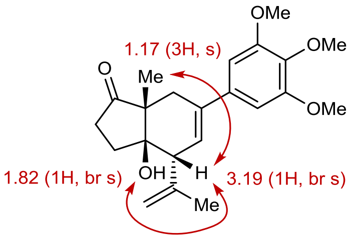
OMe
OMe
OMe
1.17 (3H, s)
Me
O
1.82 (1H, br s)
OH
H
3.19 (1H, br s)
Me

2fa

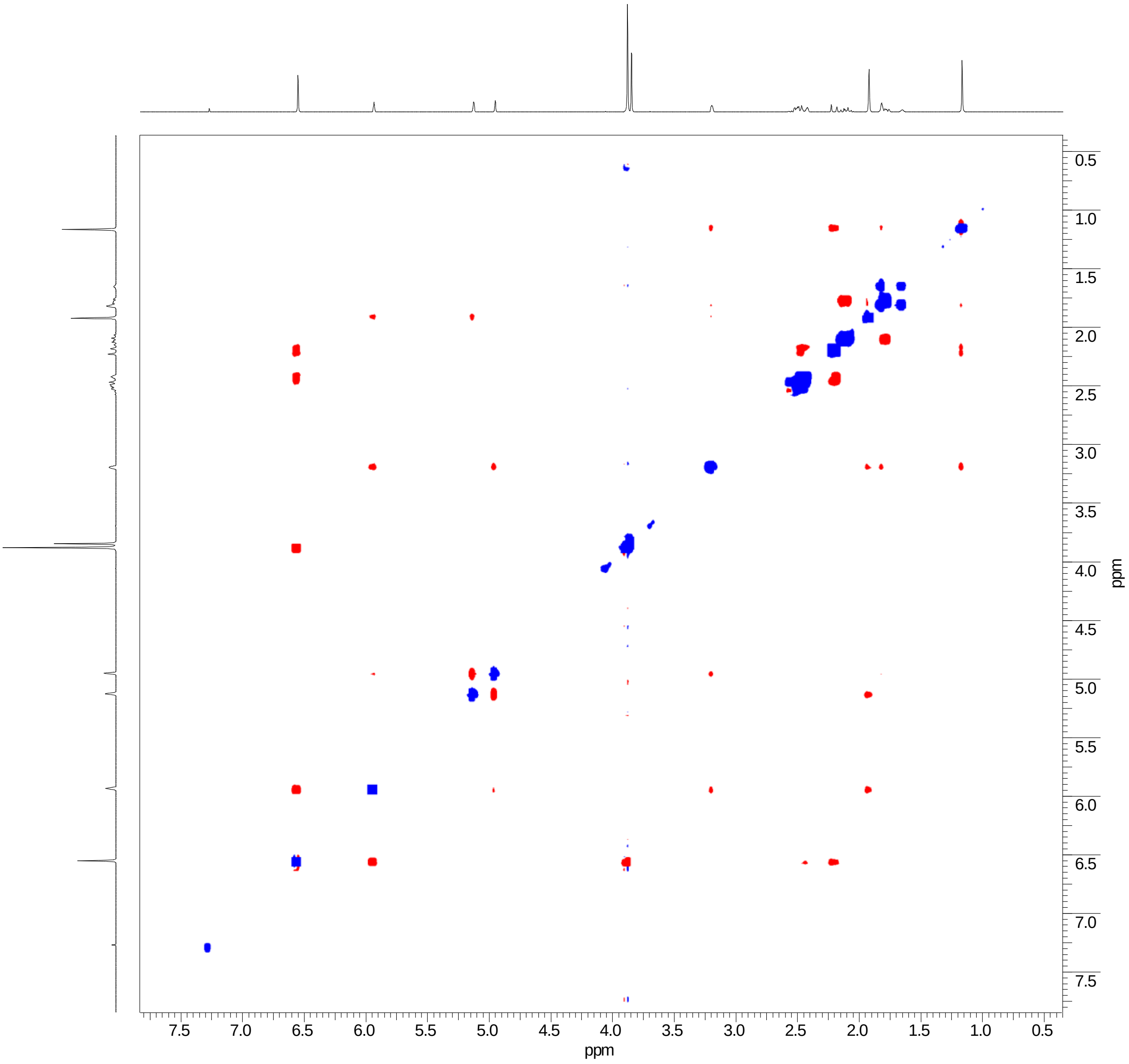
0.5
1.0
1.5
2.0
2.5
3.0
3.5
4.0
4.5
5.0
5.5
6.0
6.5
7.0
7.5
ppm
7.5
7.0
6.5
6.0
5.5
5.0
4.5
4.0
3.5
3.0
2.5
2.0
1.5
1.0
0.5
ppm

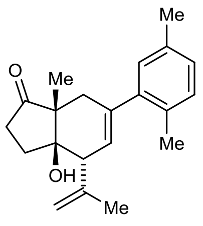
O
Me
Me
Me
OH
Me

2ga

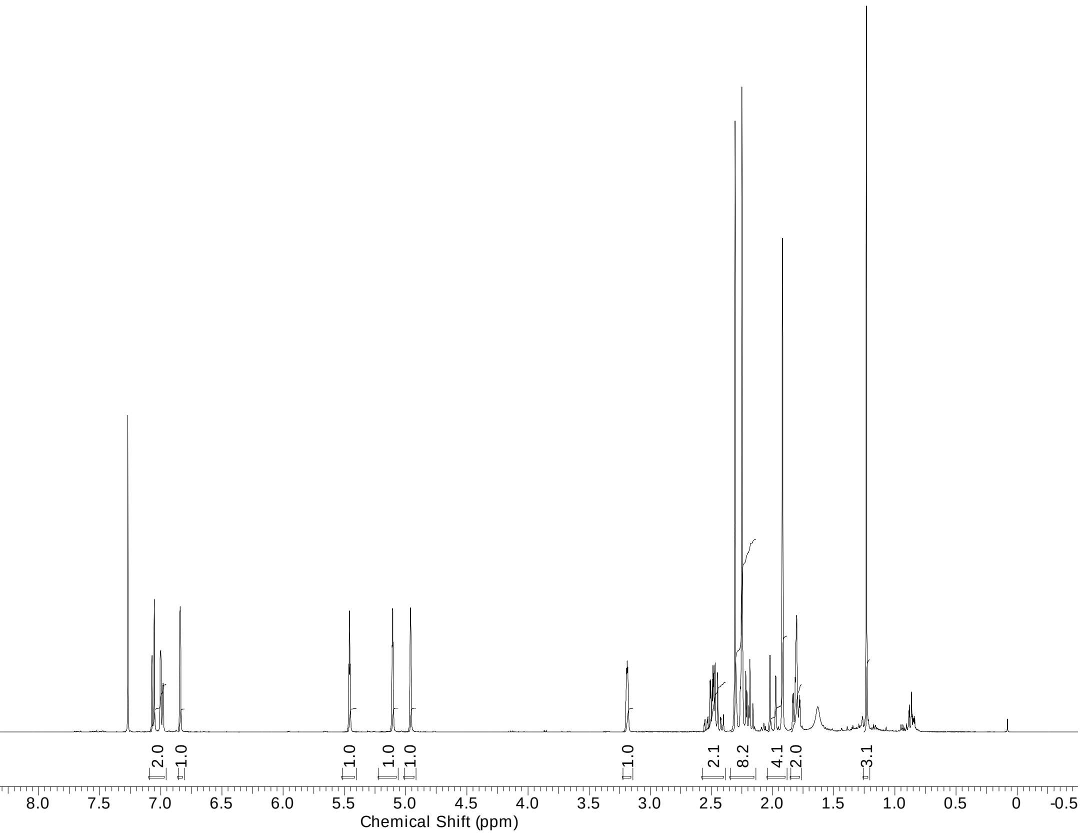
2.0
1.0
1.0
1.0
1.0
1.0
2.1
8.2
4.1
2.0
3.1
9.5 9.0 8.5 8.0 7.5 7.0 6.5 6.0 5.5 5.0 4.5 4.0 3.5 3.0 2.5 2.0 1.5 1.0 0.5 0 -0.5
Chemical Shift (ppm)

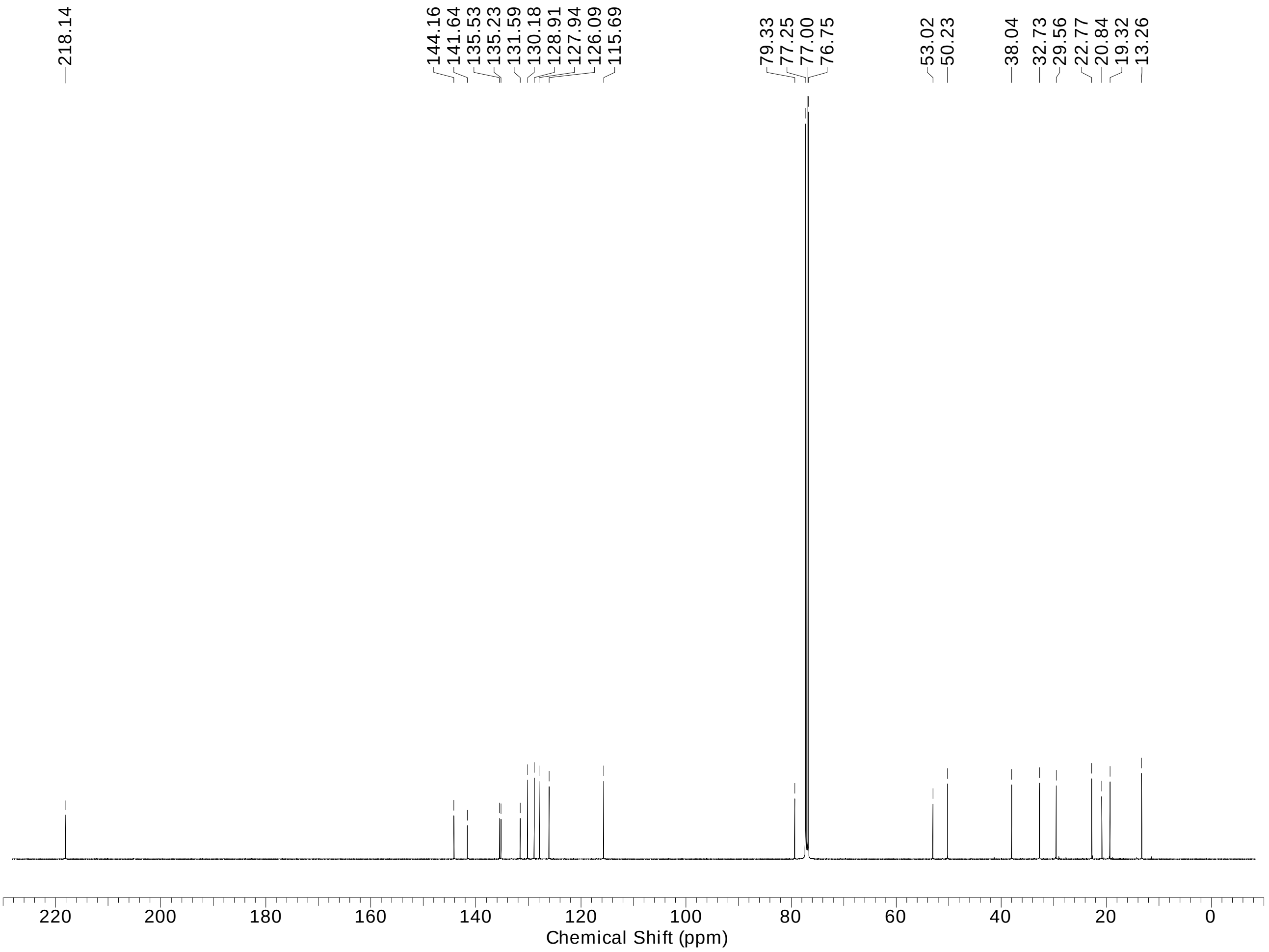
218.14
144.16
141.64
135.53
135.23
131.59
130.18
128.91
127.94
126.09
115.69
79.33
77.25
77.00
76.75
53.02
50.23
38.04
32.73
29.56
22.77
20.84
19.32
13.26
220 200 180 160 140 120 100 80 60 40 20 0
Chemical Shift (ppm)

NOESY

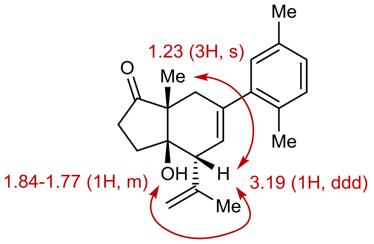

2ga

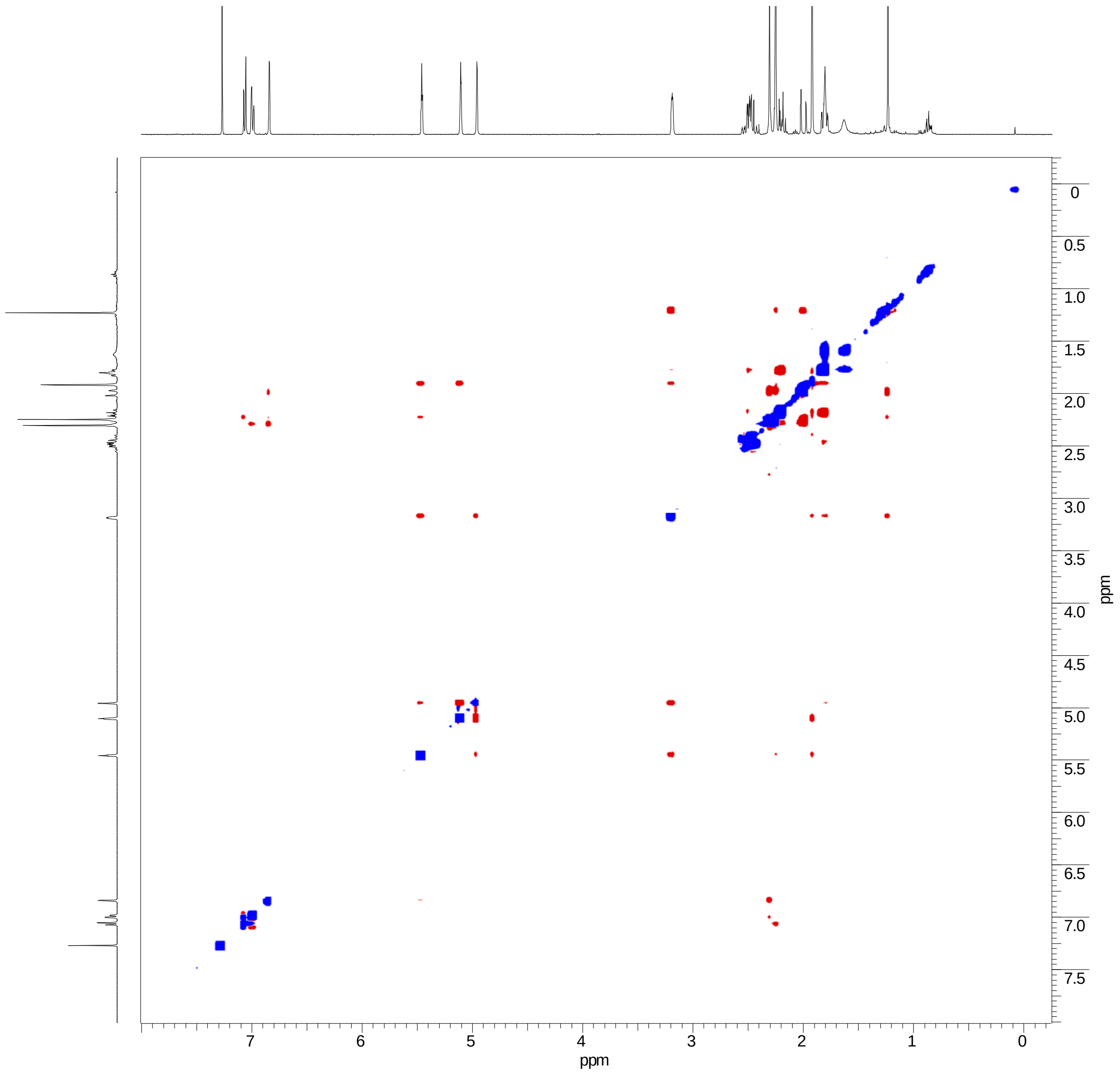

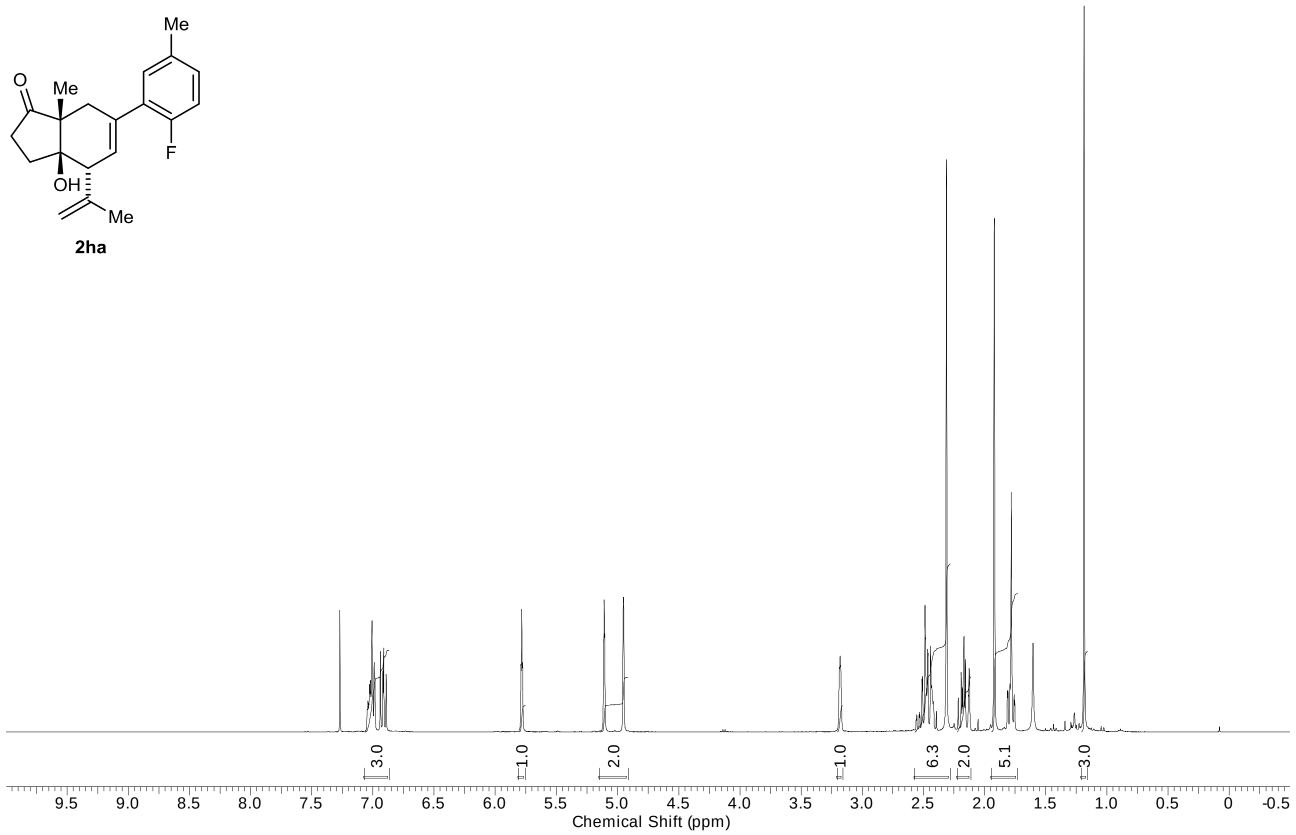
Me
O
Me
F
OH
Me
2ha
3.0
1.0
2.0
1.0
6.3
2.0
5.1
3.0
9.5 9.0 8.5 8.0 7.5 7.0 6.5 6.0 5.5 5.0 4.5 4.0 3.5 3.0 2.5 2.0 1.5 1.0 0.5 0 -0.5
Chemical Shift (ppm)

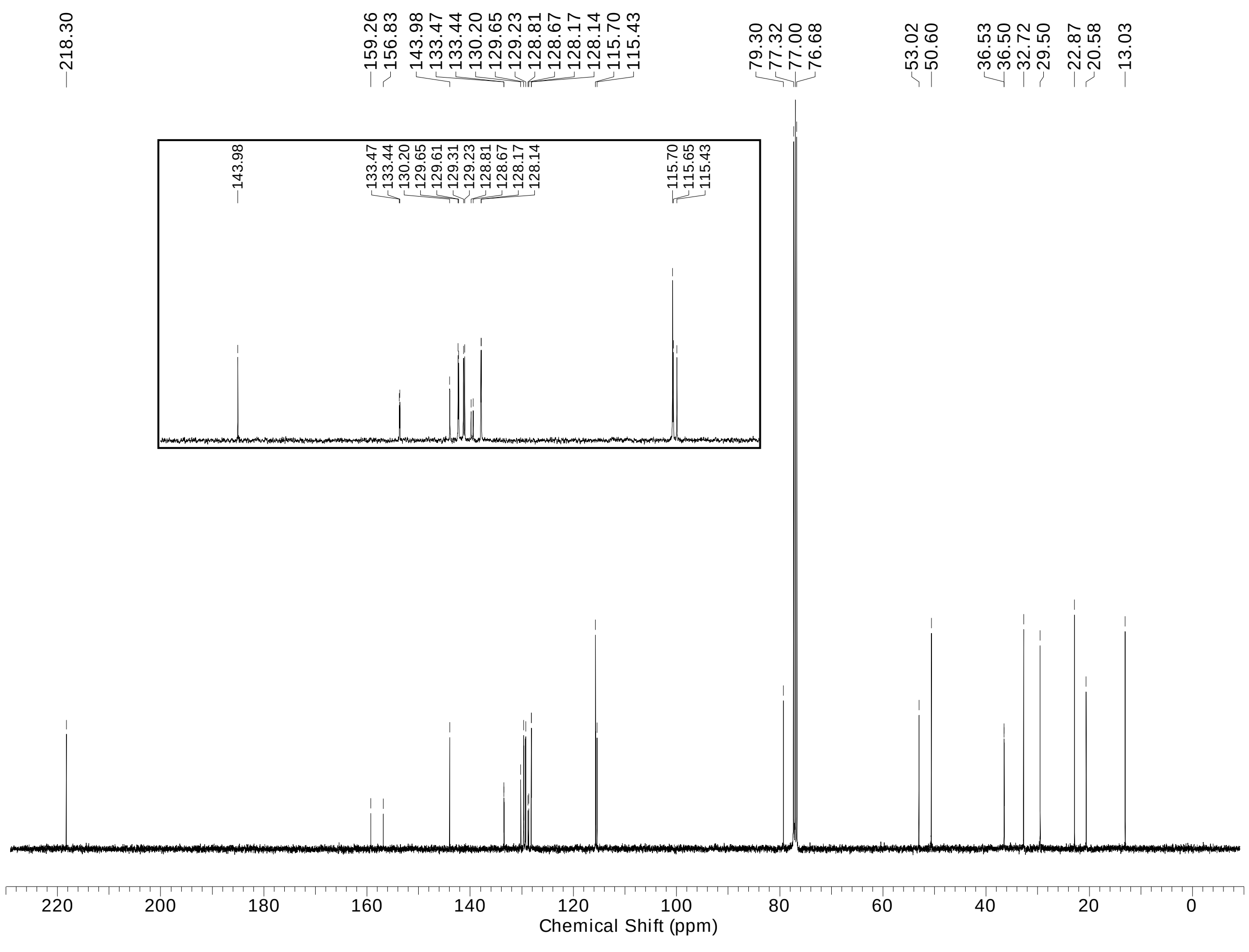
218.30
159.26
156.83
143.98
133.47
133.44
130.20
129.65
129.23
128.81
128.67
128.17
128.14
115.70
115.43
79.30
77.32
77.00
76.68
53.02
50.60
36.53
36.50
32.72
29.50
22.87
20.58
13.03
143.98
133.47
133.44
130.20
129.65
129.61
129.31
129.23
128.81
128.67
128.17
128.14
115.70
115.65
115.43
220 200 180 160 140 120 100 80 60 40 20 0
Chemical Shift (ppm)

^{19}F NMR spectrum

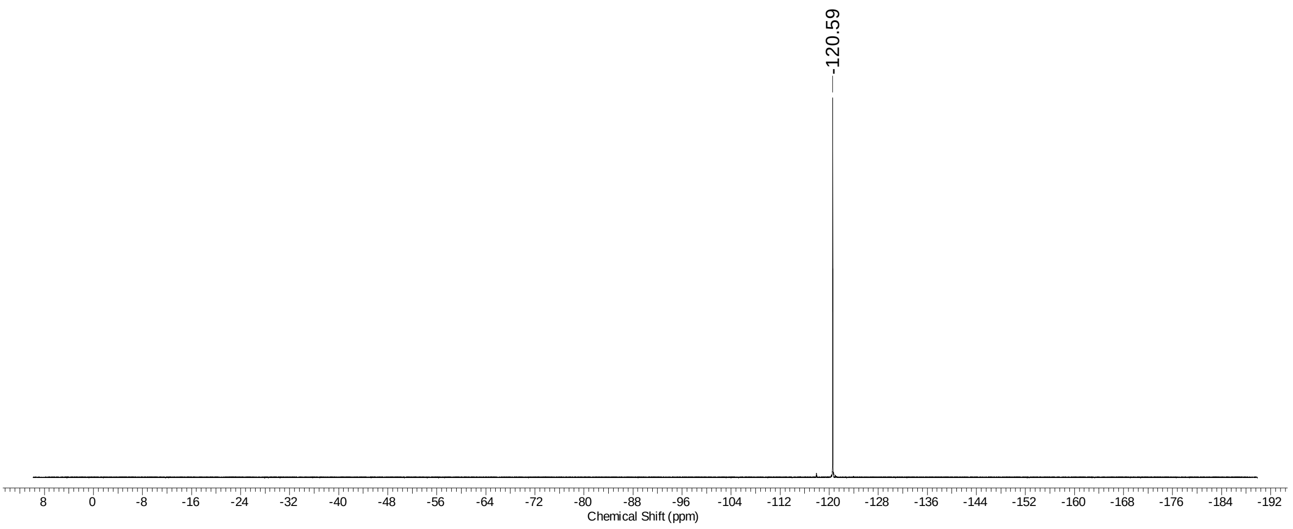

NOESY

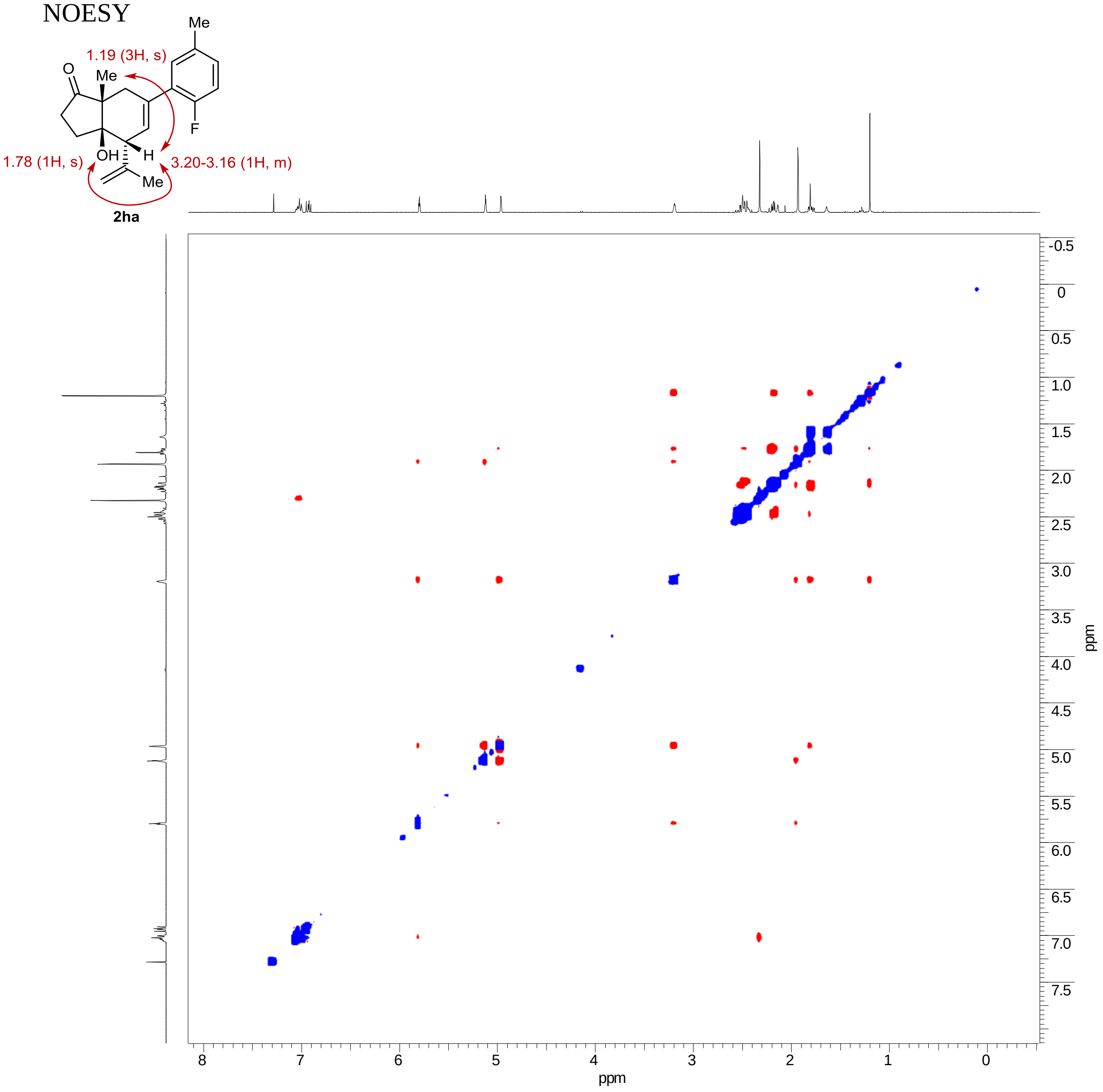

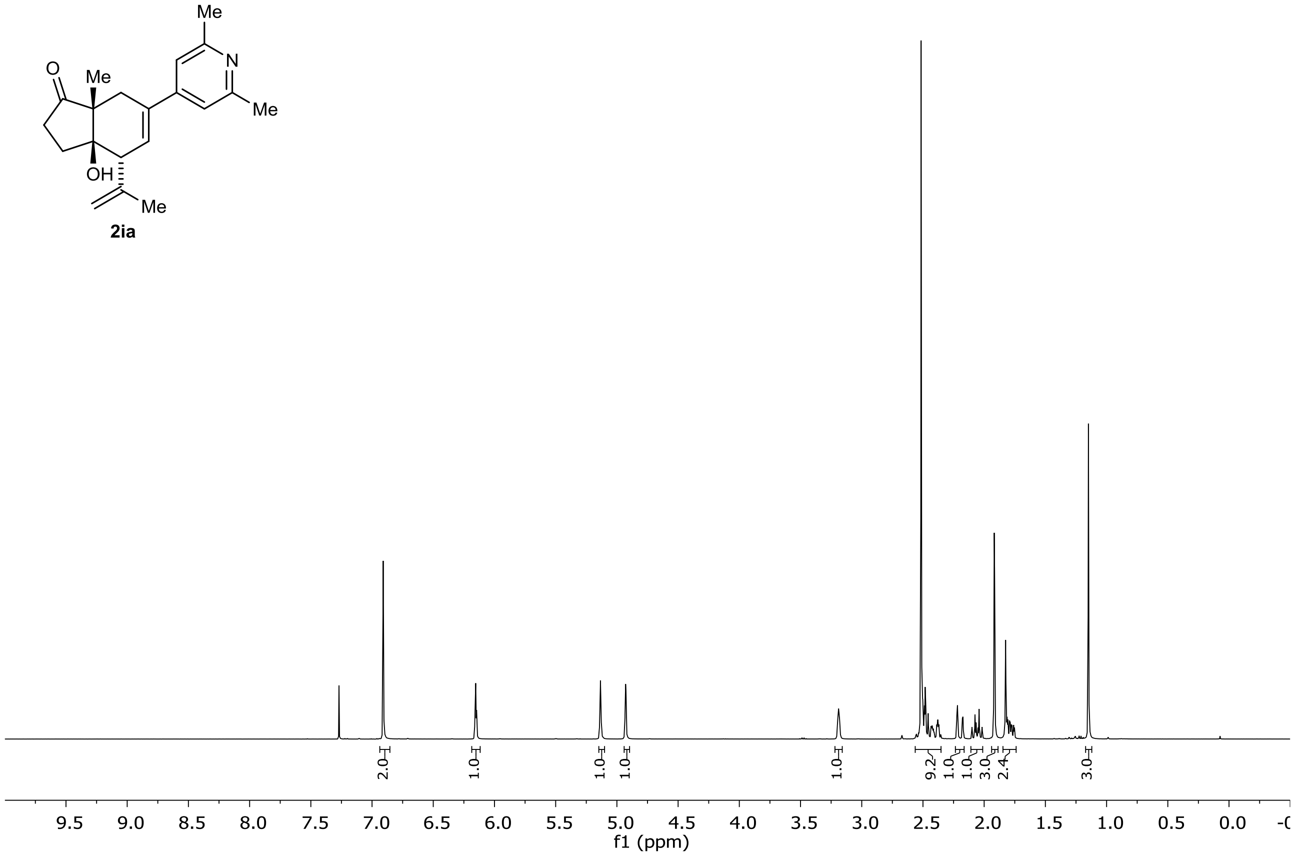
Me
N
O
Me
Me
OH
Me
2ia
2.0
1.0
1.0
1.0
1.0
9.2
1.0
1.0
3.0
2.4
3.0
9.5 9.0 8.5 8.0 7.5 7.0 6.5 6.0 5.5 5.0 4.5 4.0 3.5 3.0 2.5 2.0 1.5 1.0 0.5 0.0
f1 (ppm)

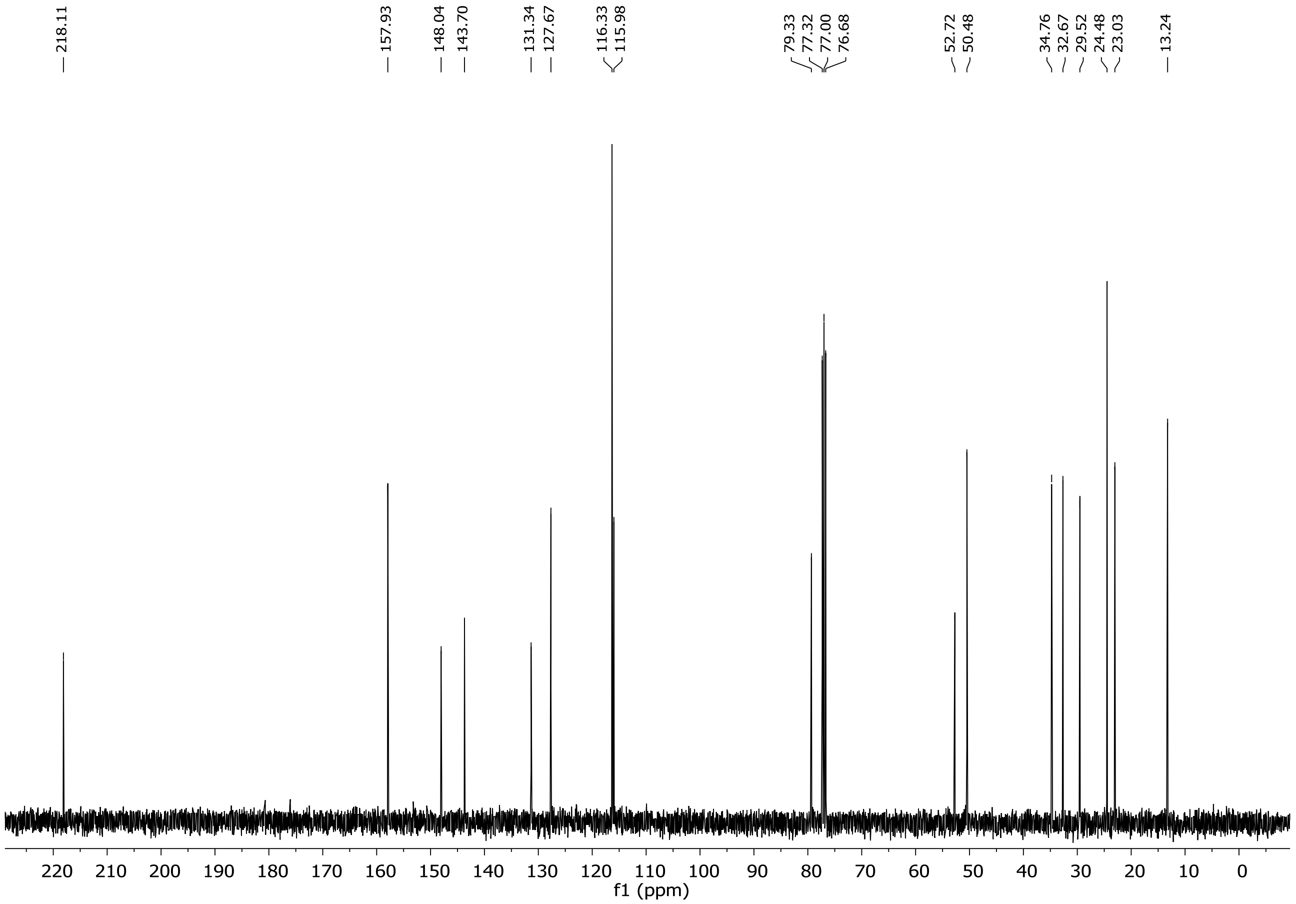
218.11
157.93
148.04
143.70
131.34
127.67
116.33
115.98
79.33
77.32
77.00
76.68
52.72
50.48
34.76
32.67
29.52
24.48
23.03
13.24
220 210 200 190 180 170 160 150 140 130 120 110 100 90 80 70 60 50 40 30 20 10 0
f1 (ppm)

NOESY

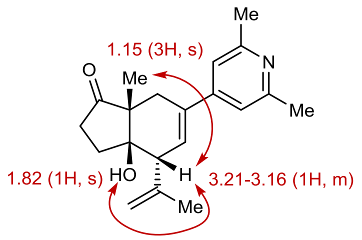

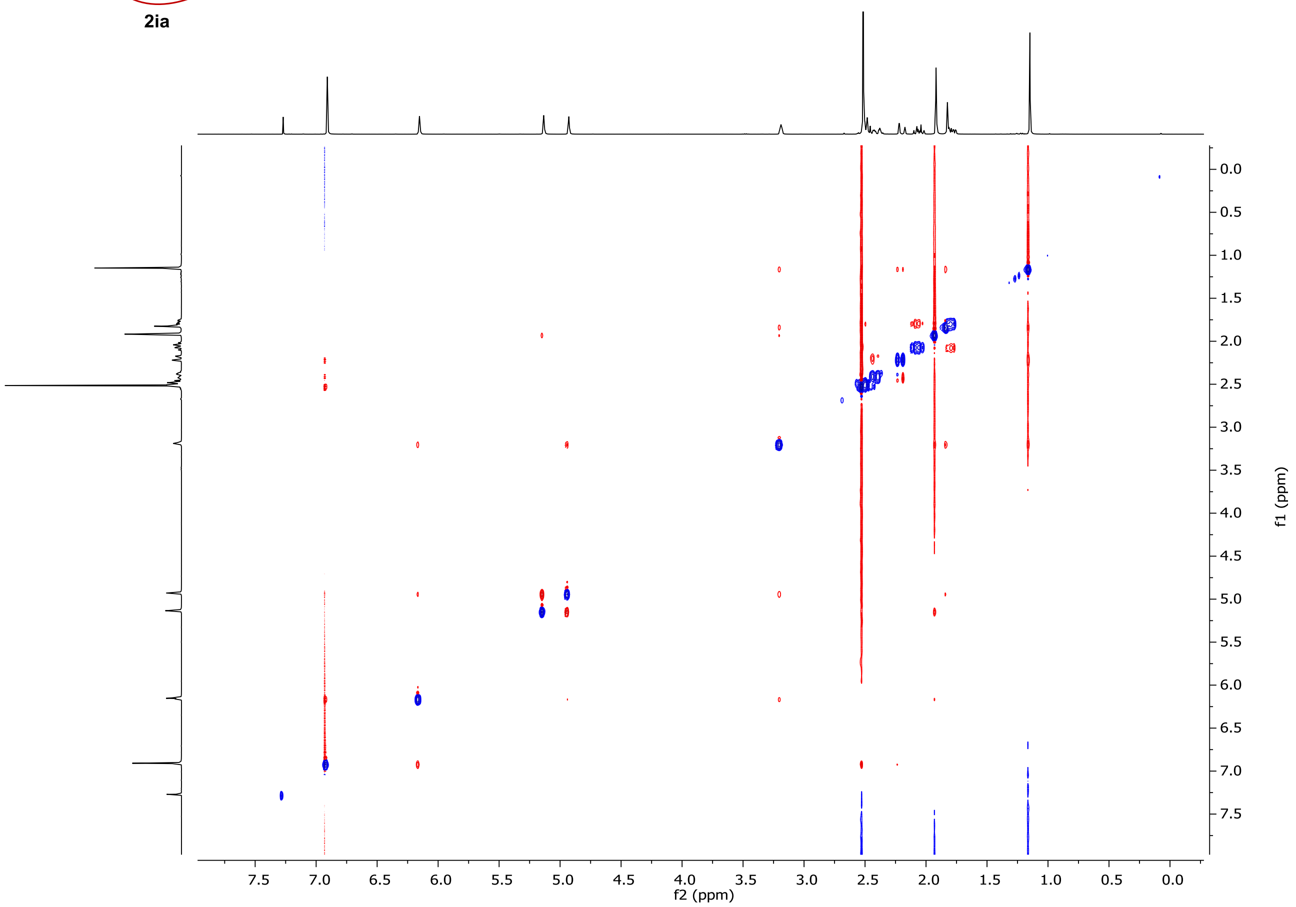

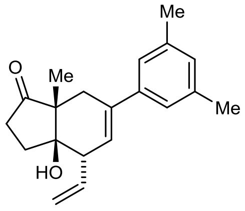
Me
O
Me
Me
HO

9ba

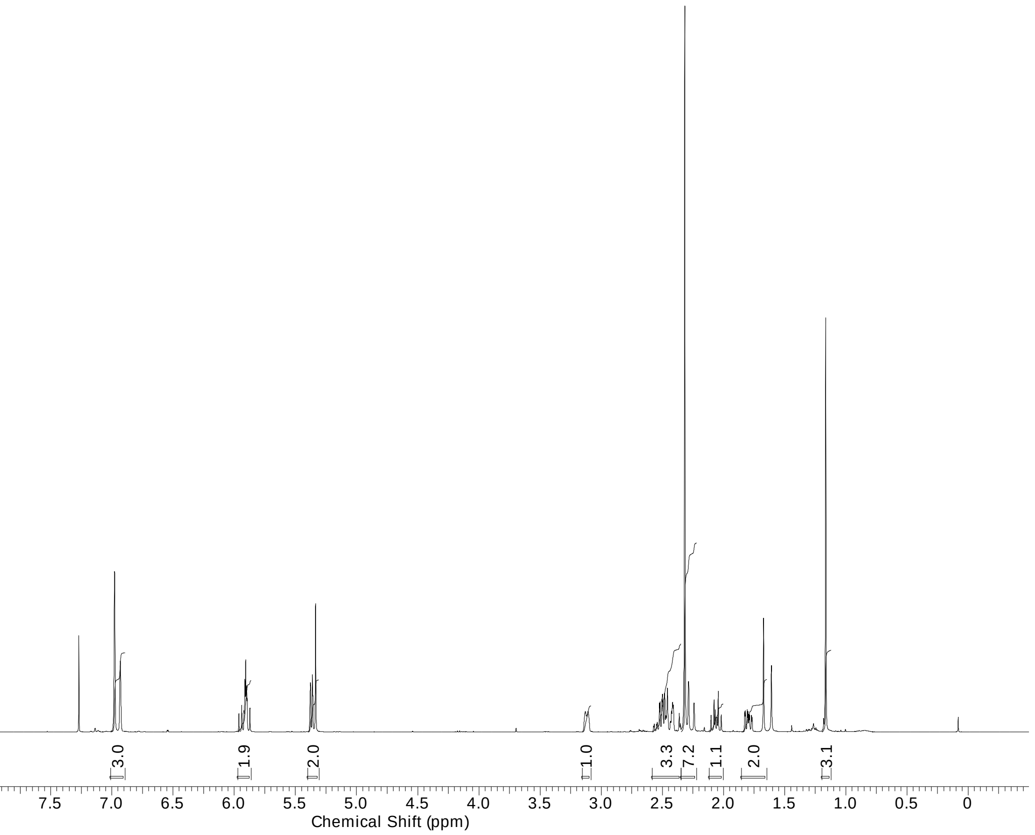
3.0
1.9
2.0
1.0
3.3
7.2
1.1
2.0
3.1
9.5 9.0 8.5 8.0 7.5 7.0 6.5 6.0 5.5 5.0 4.5 4.0 3.5 3.0 2.5 2.0 1.5 1.0 0.5 0
Chemical Shift (ppm)

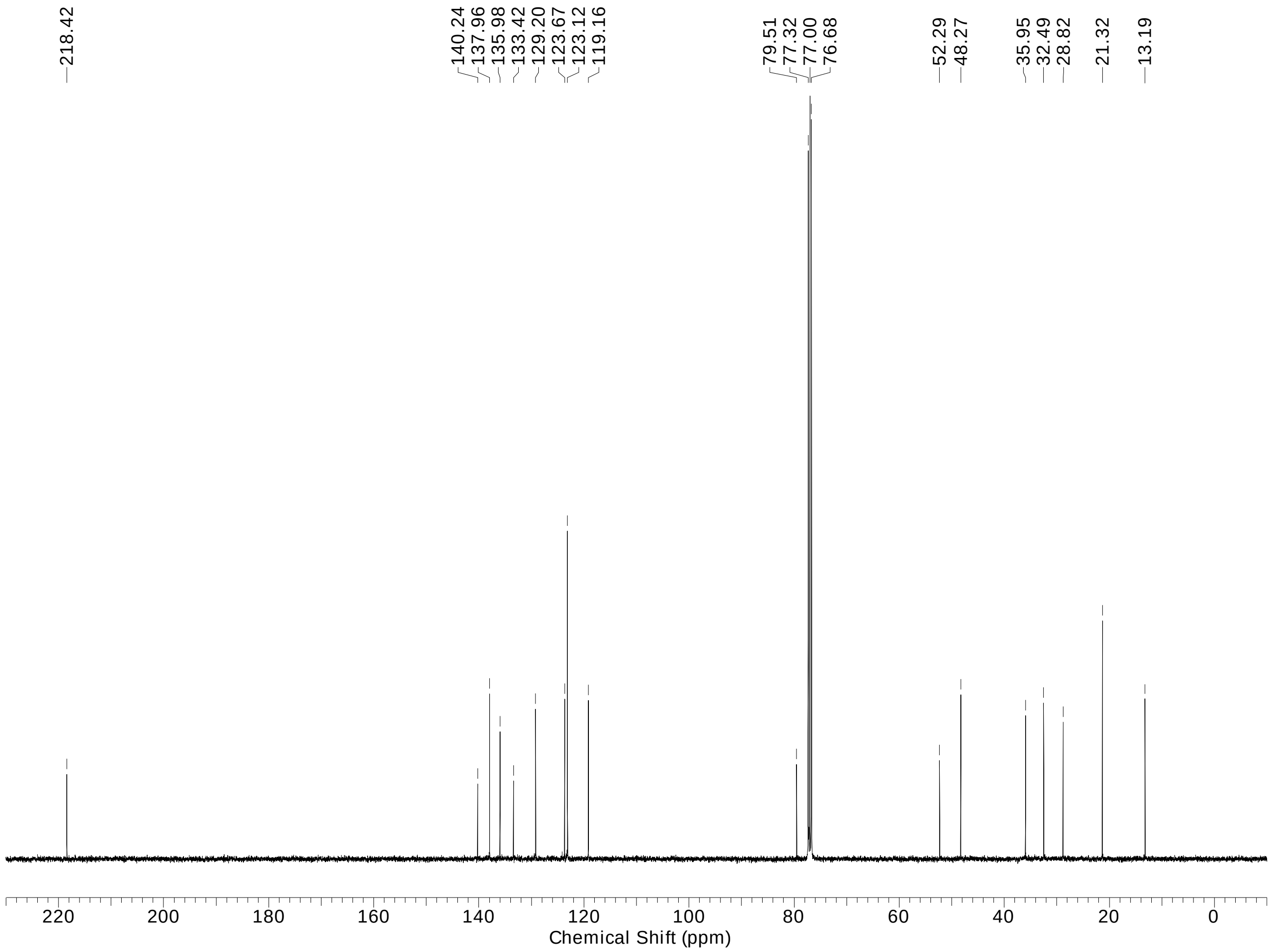
218.42
140.24
137.96
135.98
133.42
129.20
123.67
123.12
119.16
79.51
77.32
77.00
76.68
52.29
48.27
35.95
32.49
28.82
21.32
13.19
220 200 180 160 140 120 100 80 60 40 20 0
Chemical Shift (ppm)

NOESY

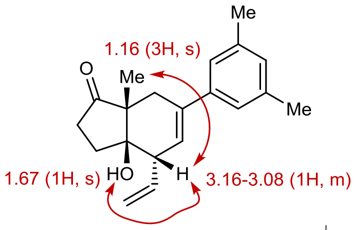

9ba

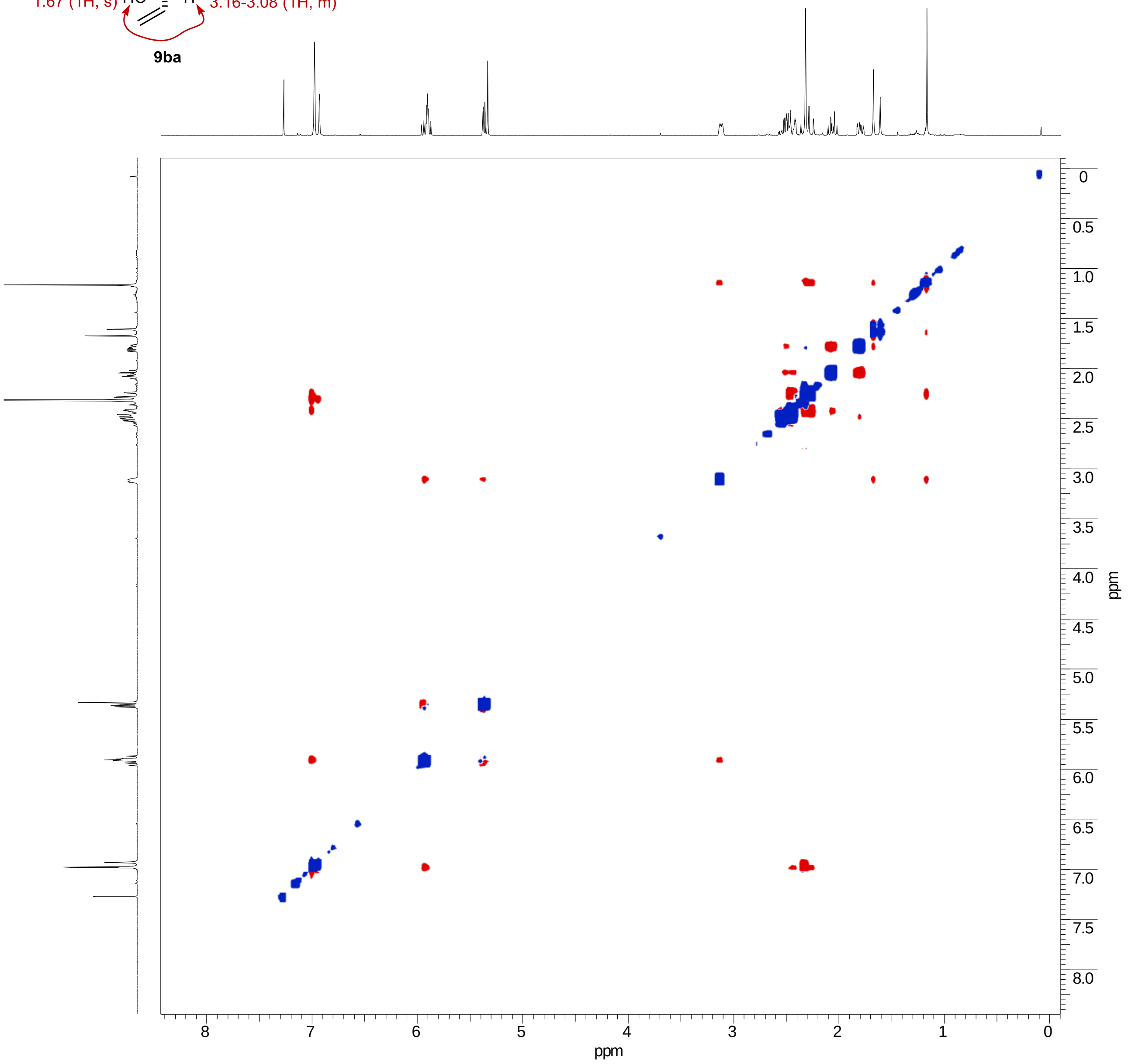

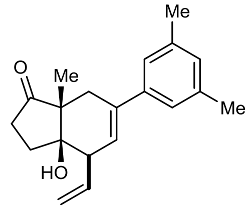
Me
O
Me
Me
HO

9bb

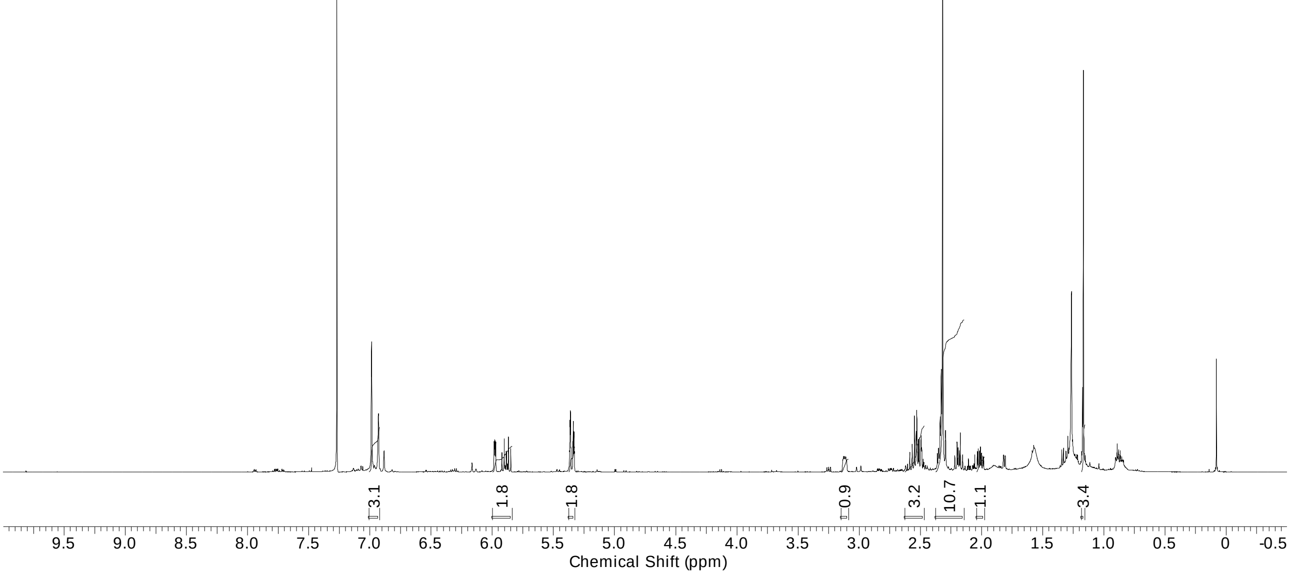
3.1
1.8
1.8
0.9
3.2
10.7
1.1
3.4
9.5 9.0 8.5 8.0 7.5 7.0 6.5 6.0 5.5 5.0 4.5 4.0 3.5 3.0 2.5 2.0 1.5 1.0 0.5 0 -0.5
Chemical Shift (ppm)

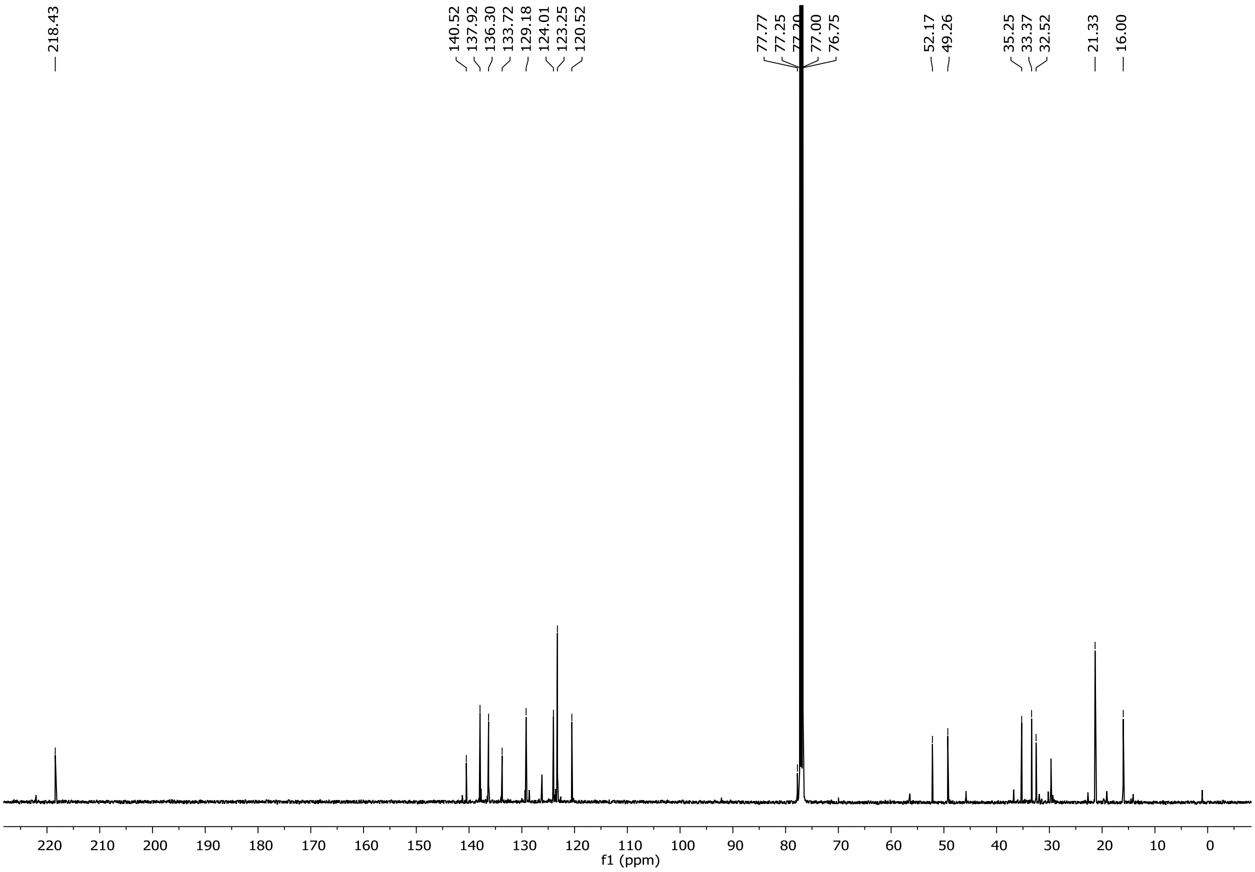
218.43
140.52
137.92
136.30
133.72
129.18
124.01
123.25
120.52
77.77
77.25
77.00
76.75
52.17
49.26
35.25
33.37
32.52
21.33
16.00
220 210 200 190 180 170 160 150 140 130 120 110 100 90 80 70 60 50 40 30 20 10 0
f1 (ppm)

NOESY

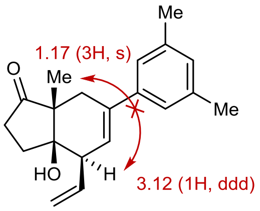

9bb

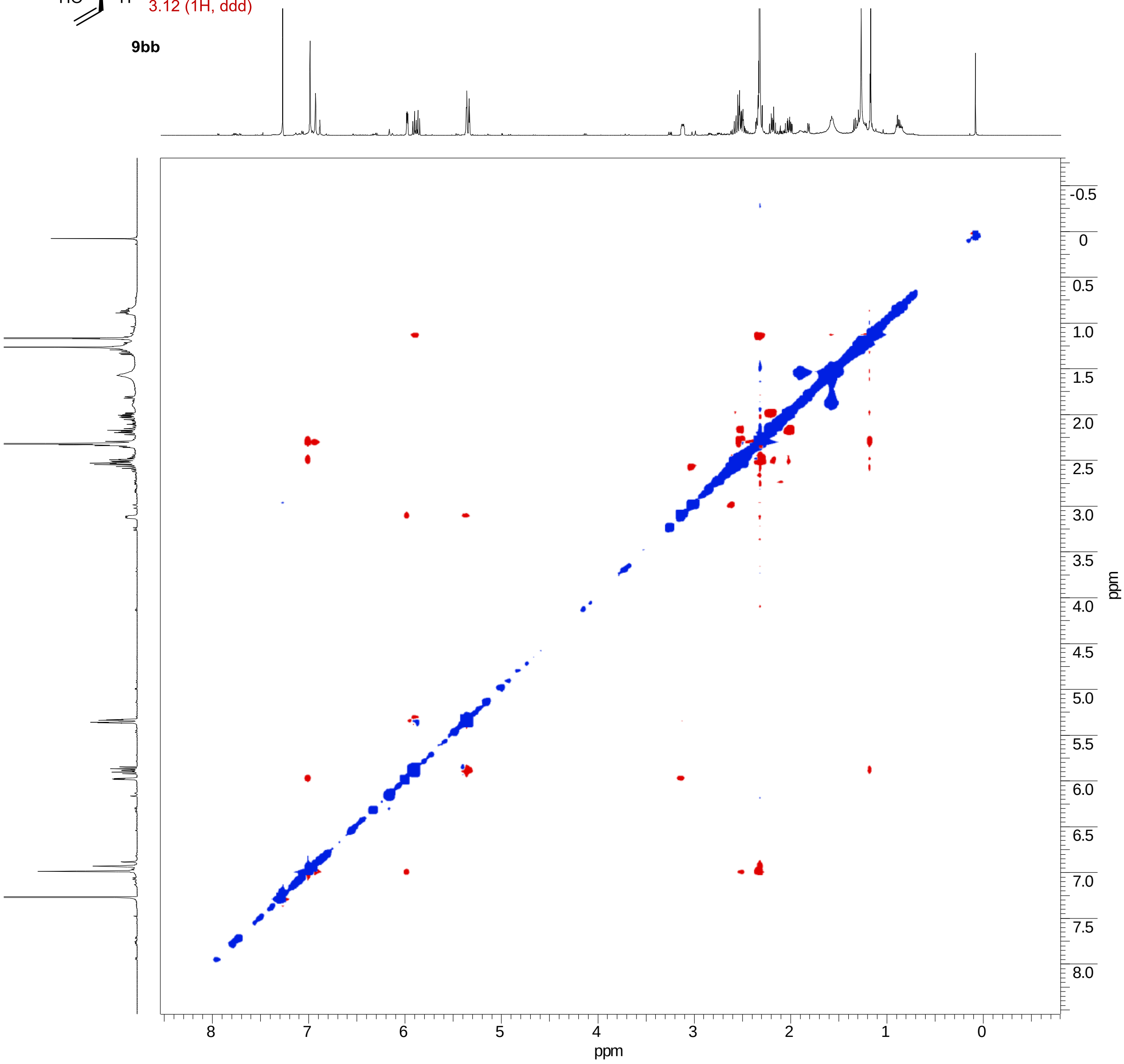

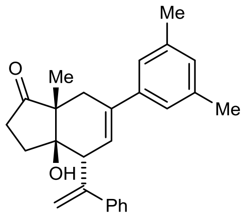
O
Me
Me
Me
OH
Ph

9ca

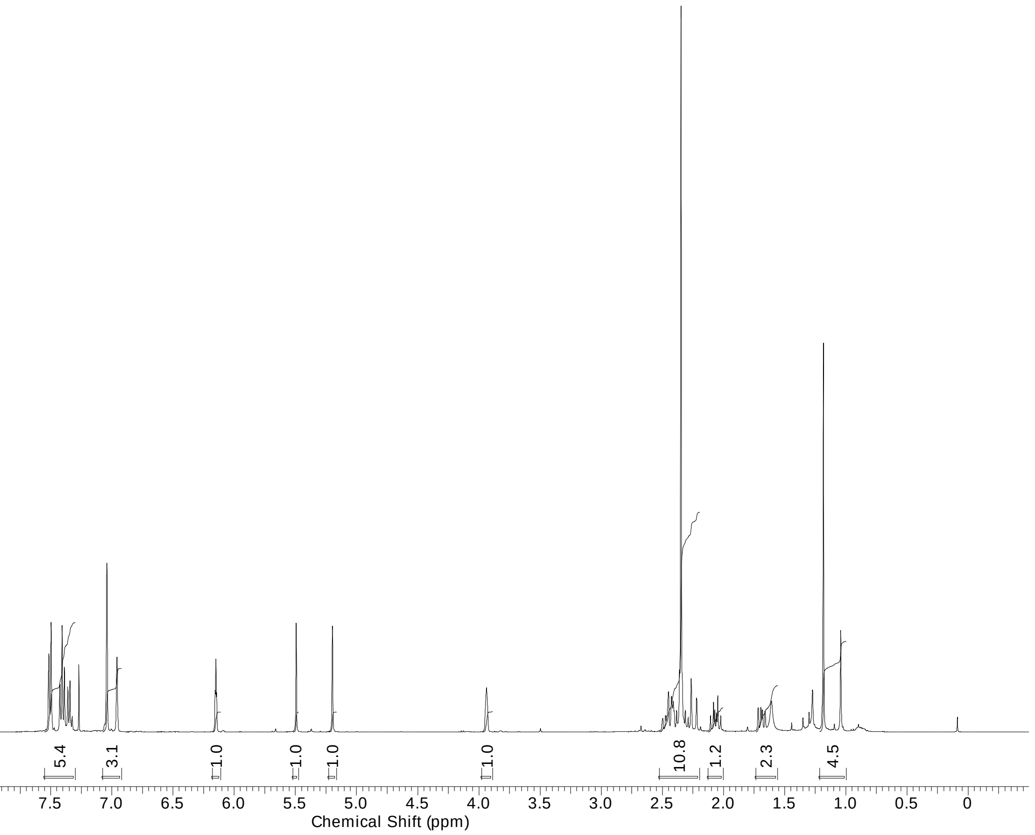
5.4
3.1
1.0
1.0
1.0
1.0
10.8
1.2
2.3
4.5
9.5 9.0 8.5 8.0 7.5 7.0 6.5 6.0 5.5 5.0 4.5 4.0 3.5 3.0 2.5 2.0 1.5 1.0 0.5 0
Chemical Shift (ppm)

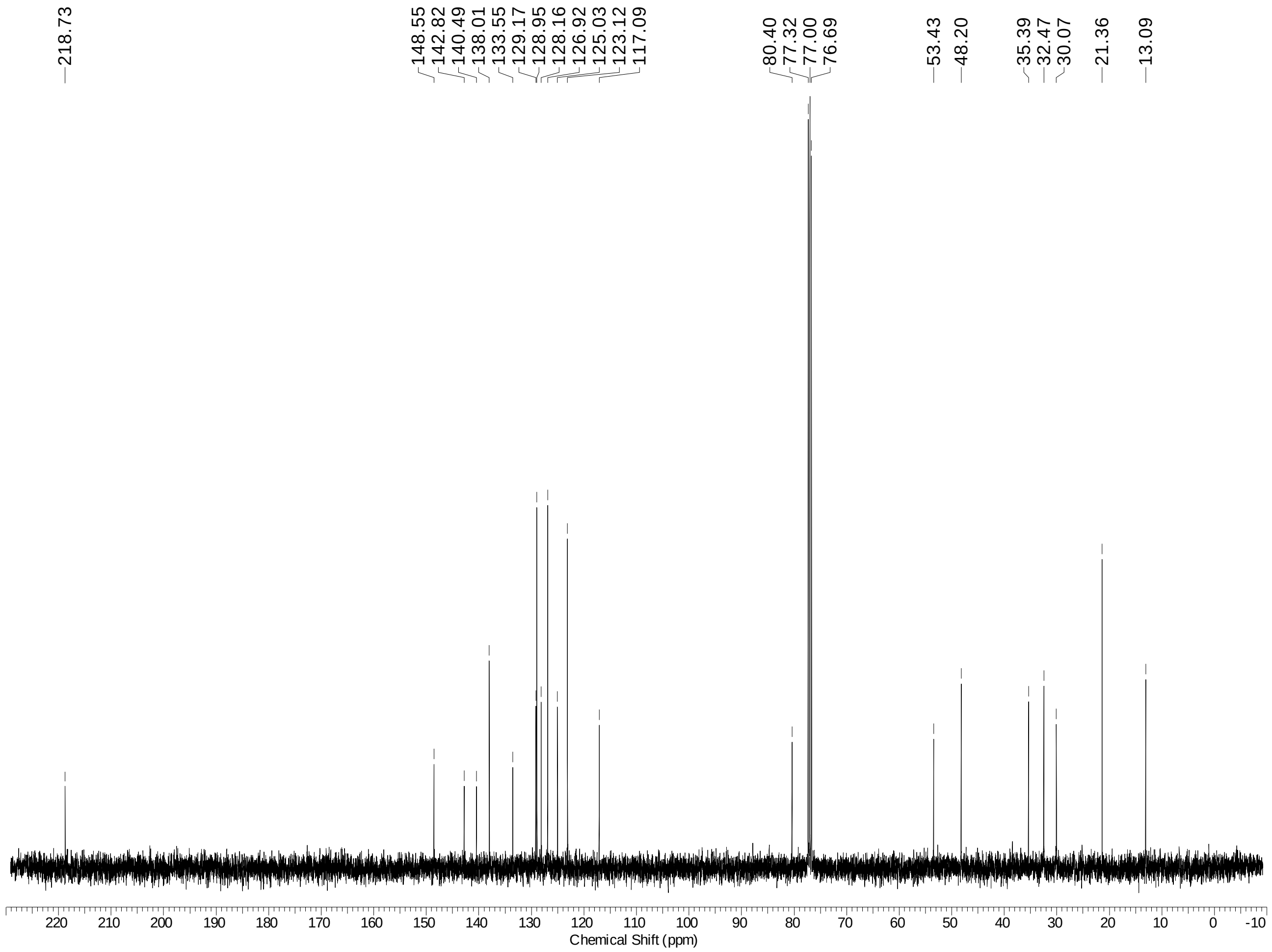
218.73
148.55
142.82
140.49
138.01
133.55
129.17
128.95
128.16
126.92
125.03
123.12
117.09
80.40
77.32
77.00
76.69
53.43
48.20
35.39
32.47
30.07
21.36
13.09
220 210 200 190 180 170 160 150 140 130 120 110 100 90 80 70 60 50 40 30 20 10 0 -10
Chemical Shift (ppm)

NOESY

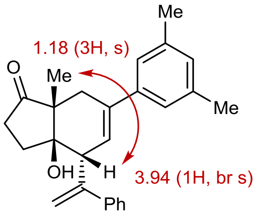
Me
1.18 (3H, s)
O
Me
Me
OH
H
3.94 (1H, br s)
Ph

9ca

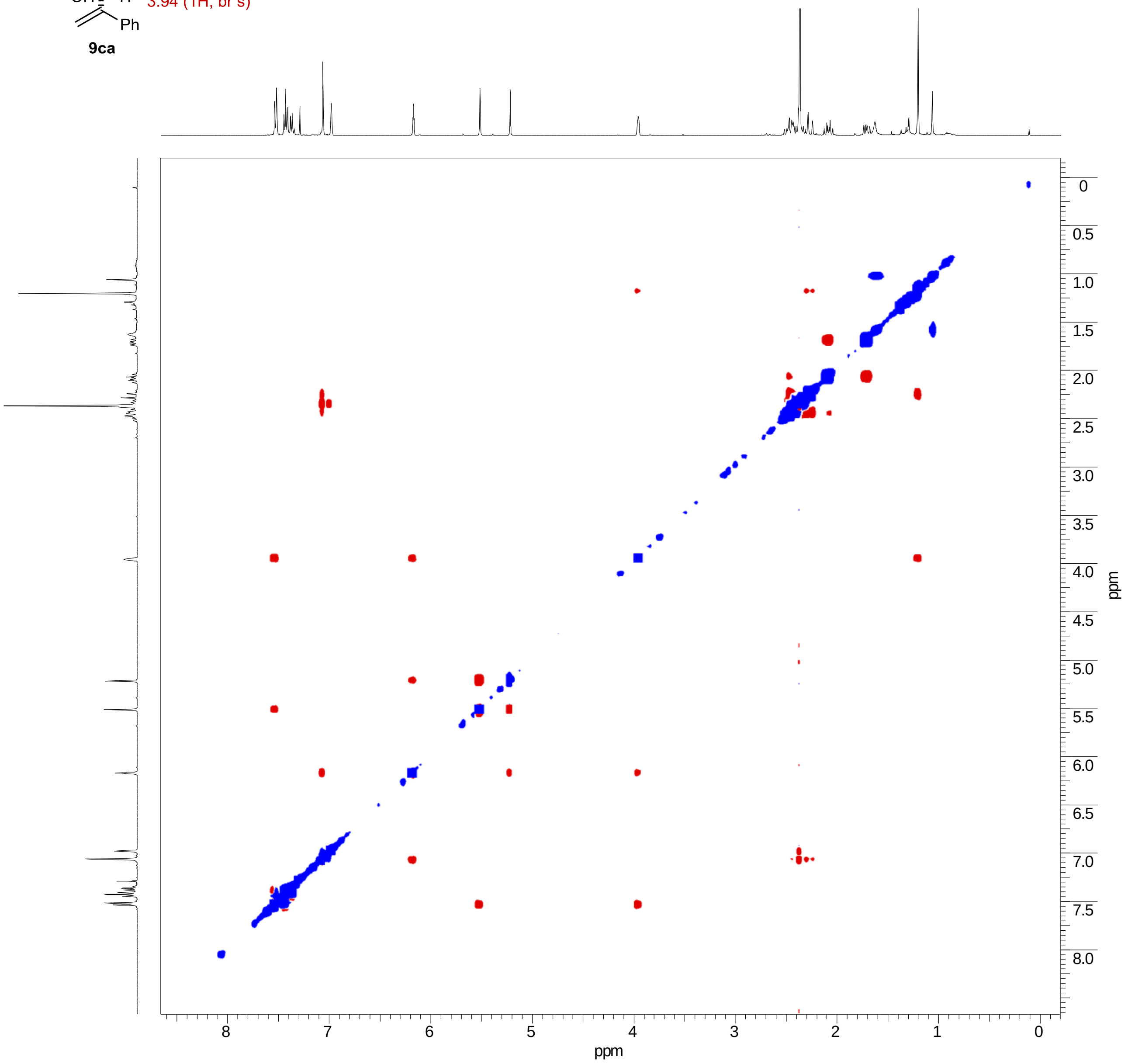
0
0.5
1.0
1.5
2.0
2.5
3.0
3.5
4.0
4.5
5.0
5.5
6.0
6.5
7.0
7.5
8.0
ppm
8
7
6
5
4
3
2
1
0
ppm

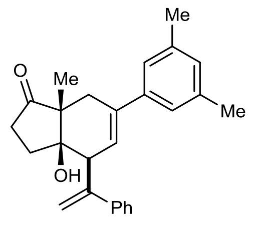
Me
O
Me
Me
OH
Ph

9cb

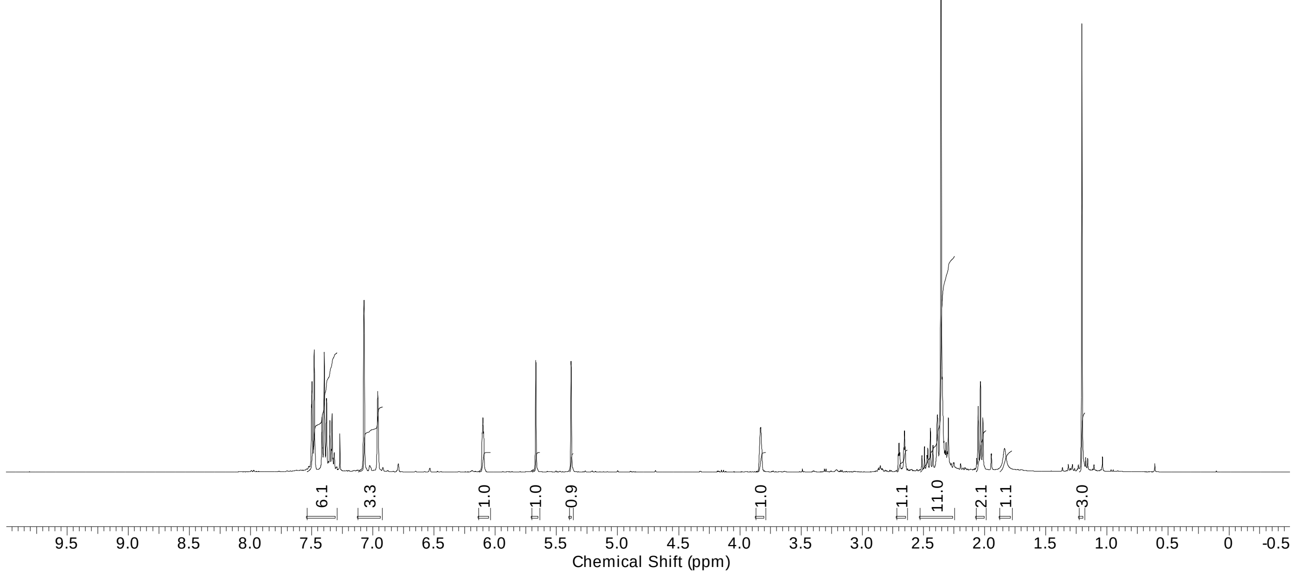
6.1
3.3
1.0
1.0
0.9
1.0
1.1
11.0
2.1
1.1
3.0
9.5 9.0 8.5 8.0 7.5 7.0 6.5 6.0 5.5 5.0 4.5 4.0 3.5 3.0 2.5 2.0 1.5 1.0 0.5 0 -0.5
Chemical Shift (ppm)

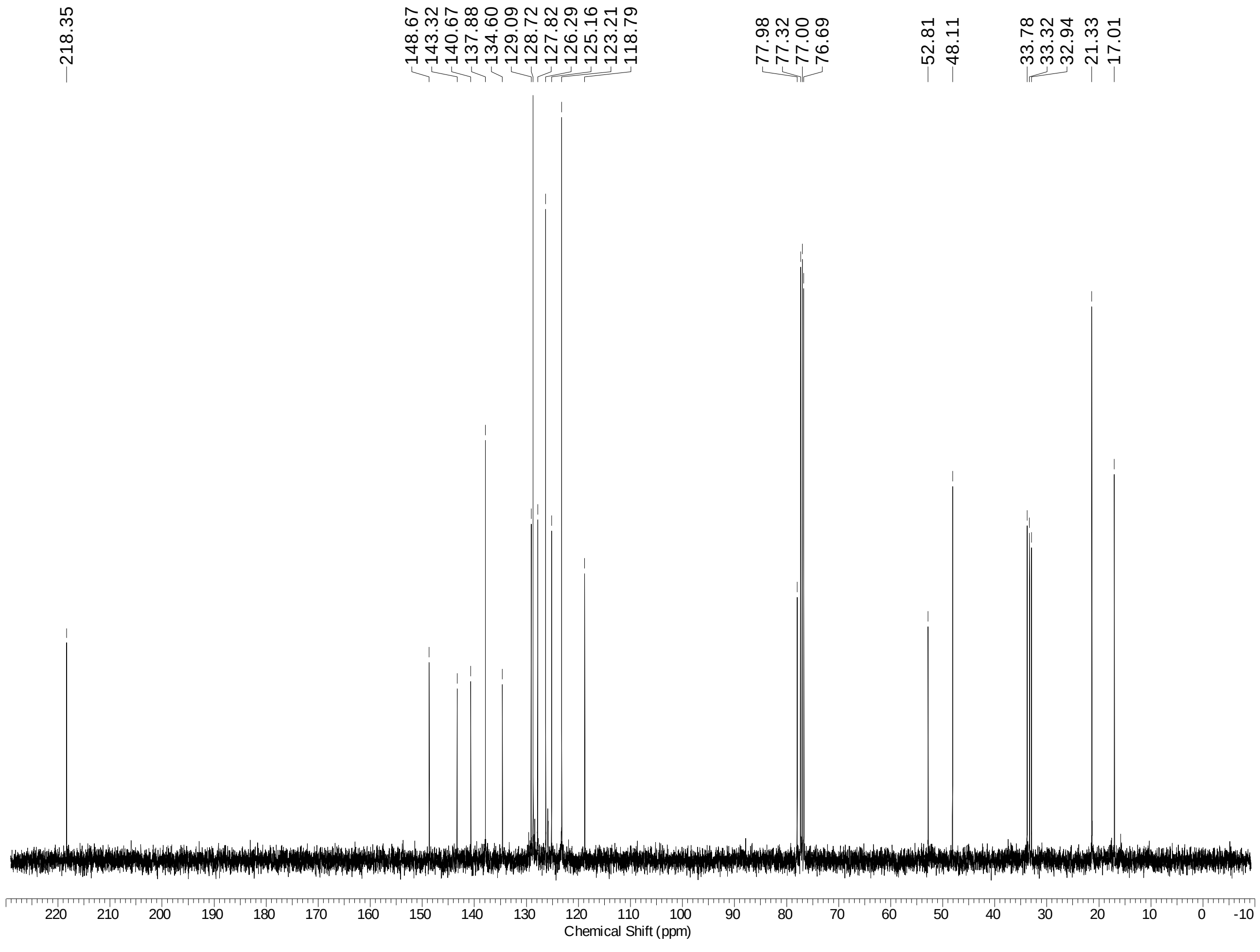
218.35
148.67
143.32
140.67
137.88
134.60
129.09
128.72
127.82
126.29
125.16
123.21
118.79
77.98
77.32
77.00
76.69
52.81
48.11
33.78
33.32
32.94
21.33
17.01
220 210 200 190 180 170 160 150 140 130 120 110 100 90 80 70 60 50 40 30 20 10 0 -10
Chemical Shift (ppm)

NOESY

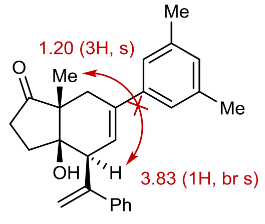

9cb

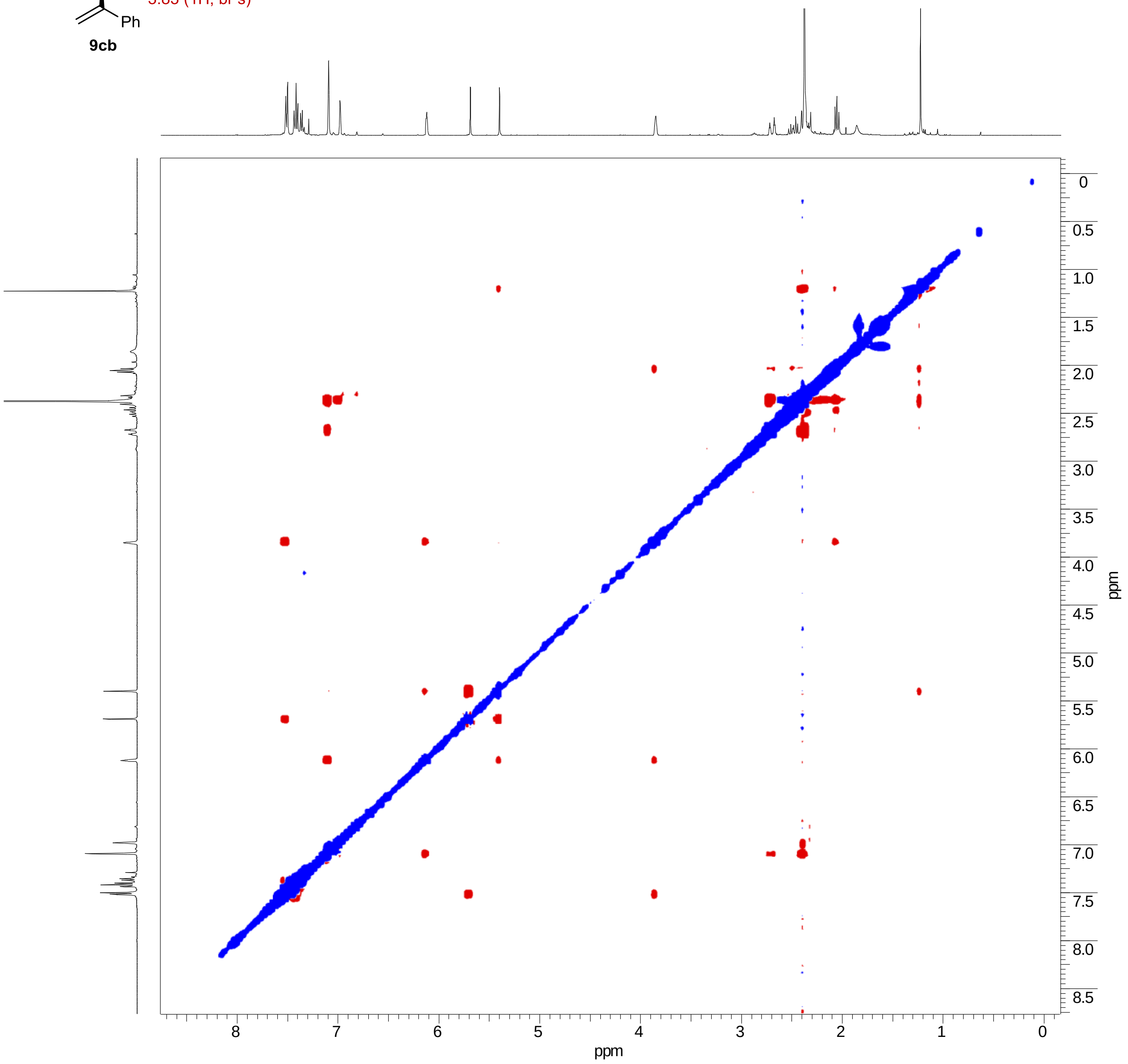

This NOESY NMR spectrum does not constitute strong proof of the relative configuration of **9cb**. The relative configuration of **9cb** was determined by X-ray crystallography (see page 21).

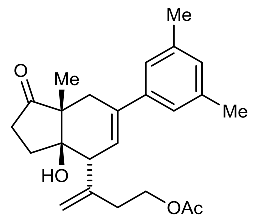
O
Me
Me
Me
HO
OAc

9da

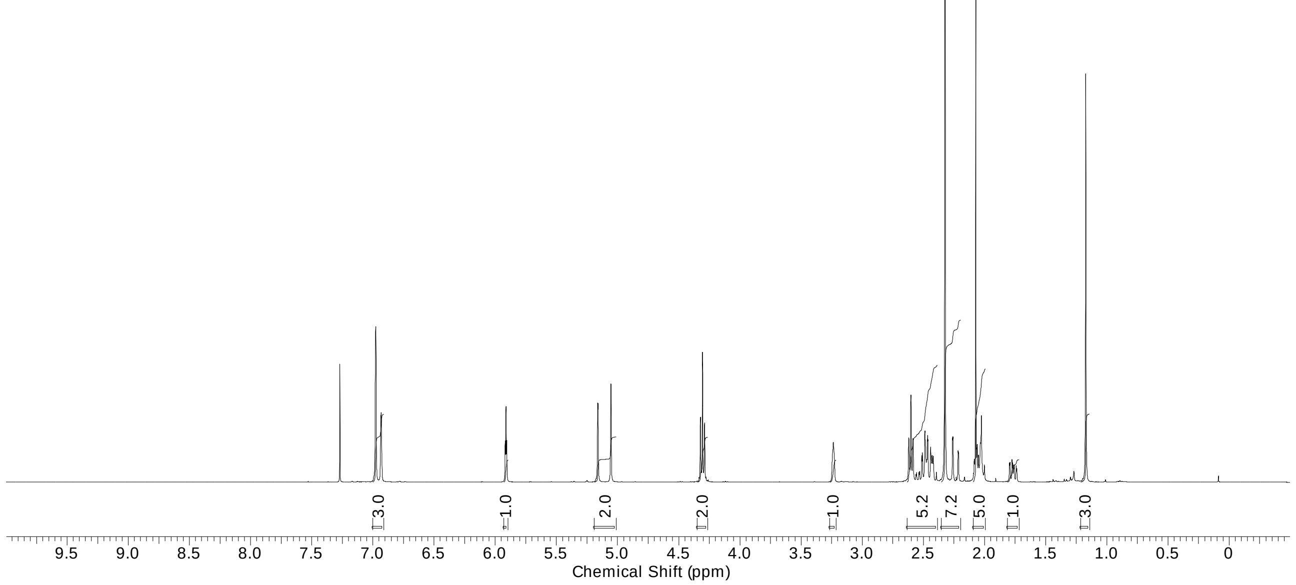
3.0
1.0
2.0
2.0
1.0
5.2
7.2
5.0
1.0
3.0
9.5 9.0 8.5 8.0 7.5 7.0 6.5 6.0 5.5 5.0 4.5 4.0 3.5 3.0 2.5 2.0 1.5 1.0 0.5 0
Chemical Shift (ppm)

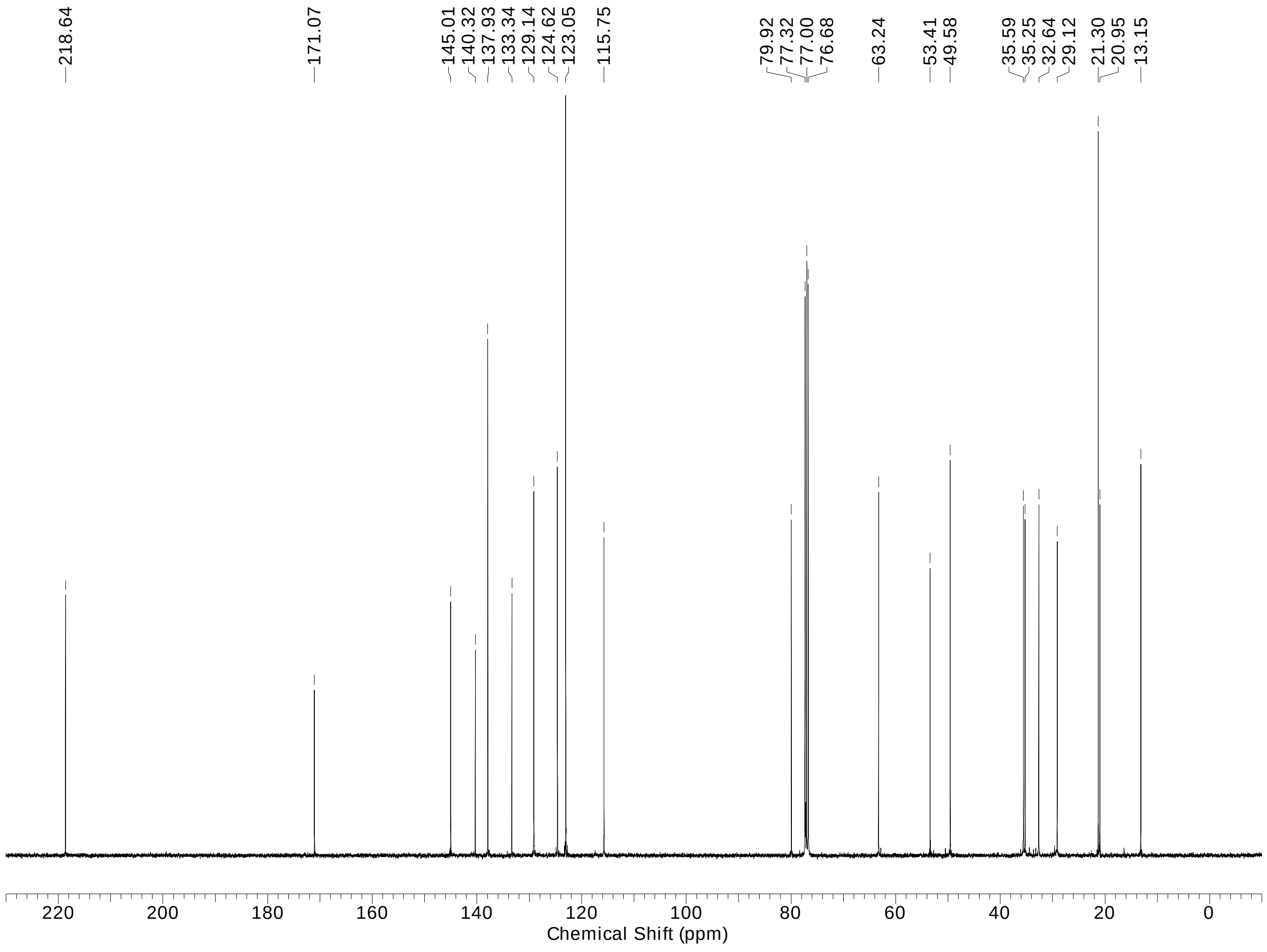
218.64
171.07
145.01
140.32
137.93
133.34
129.14
124.62
123.05
115.75
79.92
77.32
77.00
76.68
63.24
53.41
49.58
35.59
35.25
32.64
29.12
21.30
20.95
13.15
220 200 180 160 140 120 100 80 60 40 20 0
Chemical Shift (ppm)

NOESY

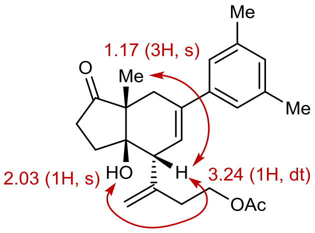

9da

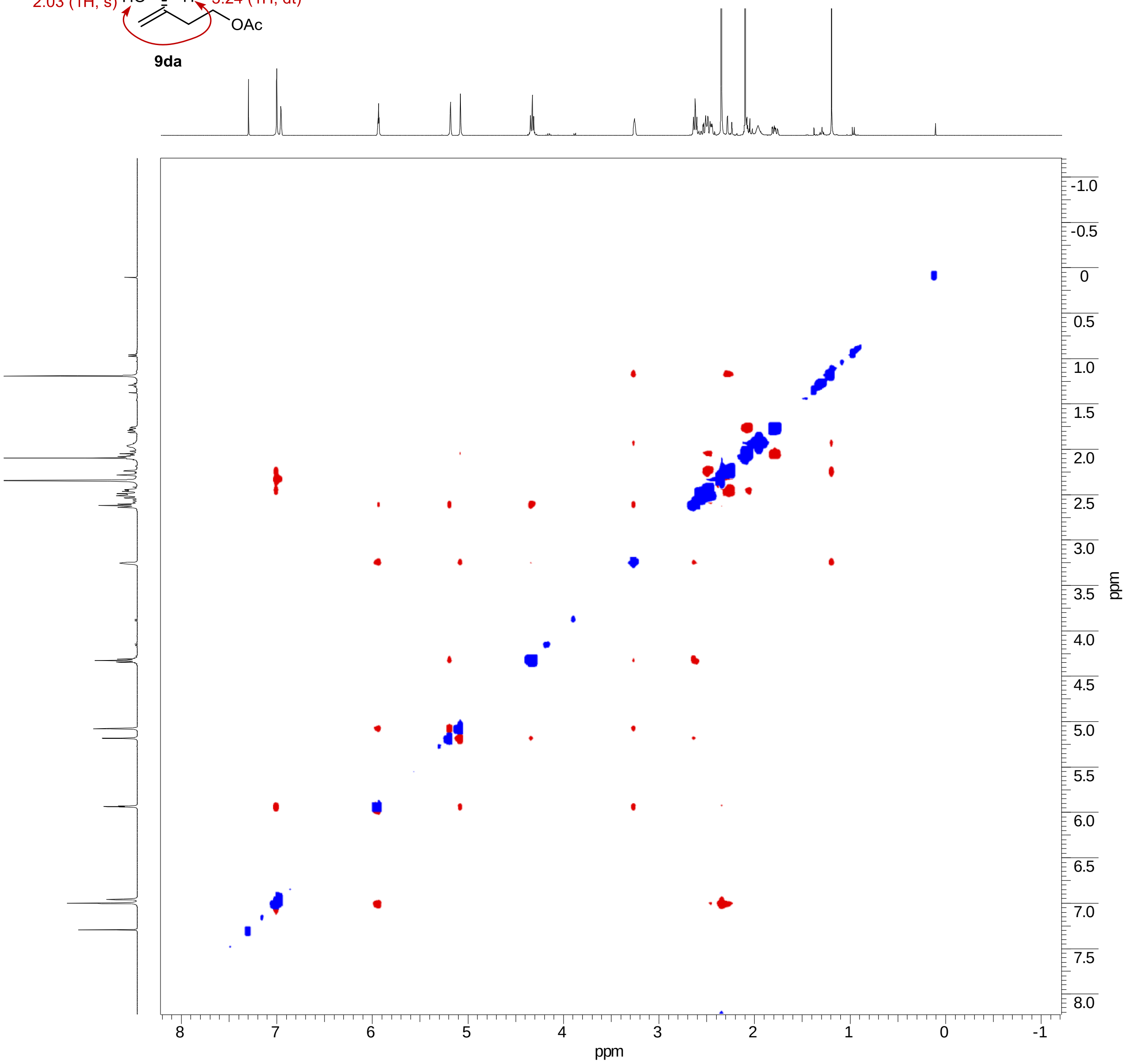

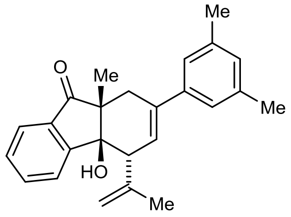
O
Me
HO
Me
Me
Me

9ea

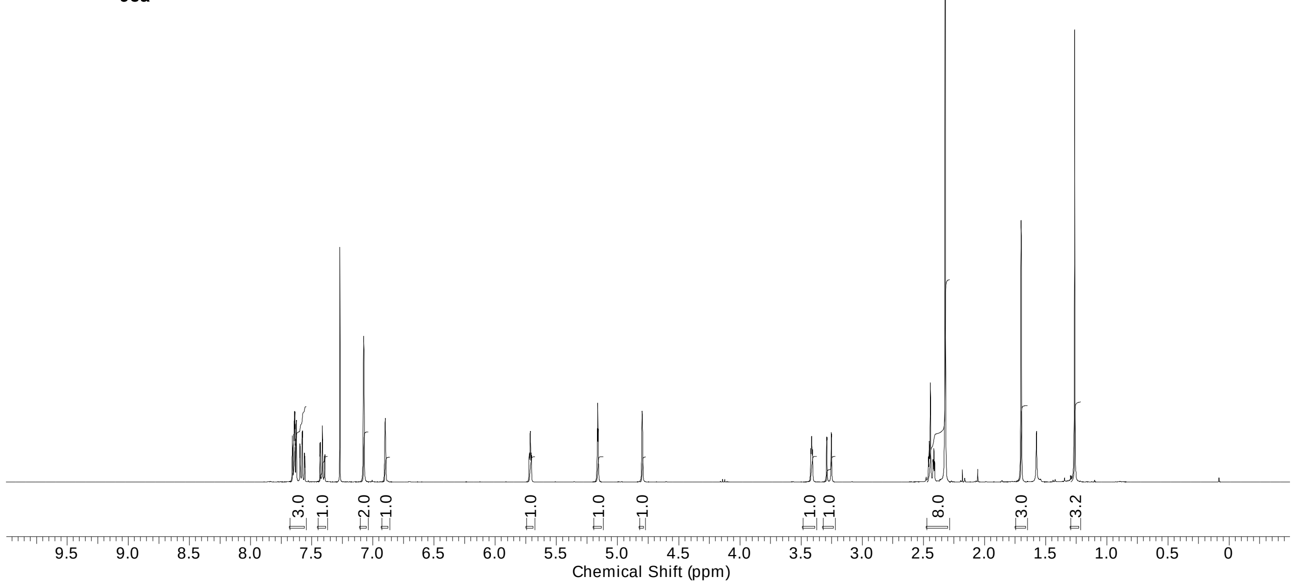
3.0
1.0
2.0
1.0
1.0
1.0
1.0
1.0
1.0
8.0
3.0
3.2
9.5
9.0
8.5
8.0
7.5
7.0
6.5
6.0
5.5
5.0
4.5
4.0
3.5
3.0
2.5
2.0
1.5
1.0
0.5
0
Chemical Shift (ppm)

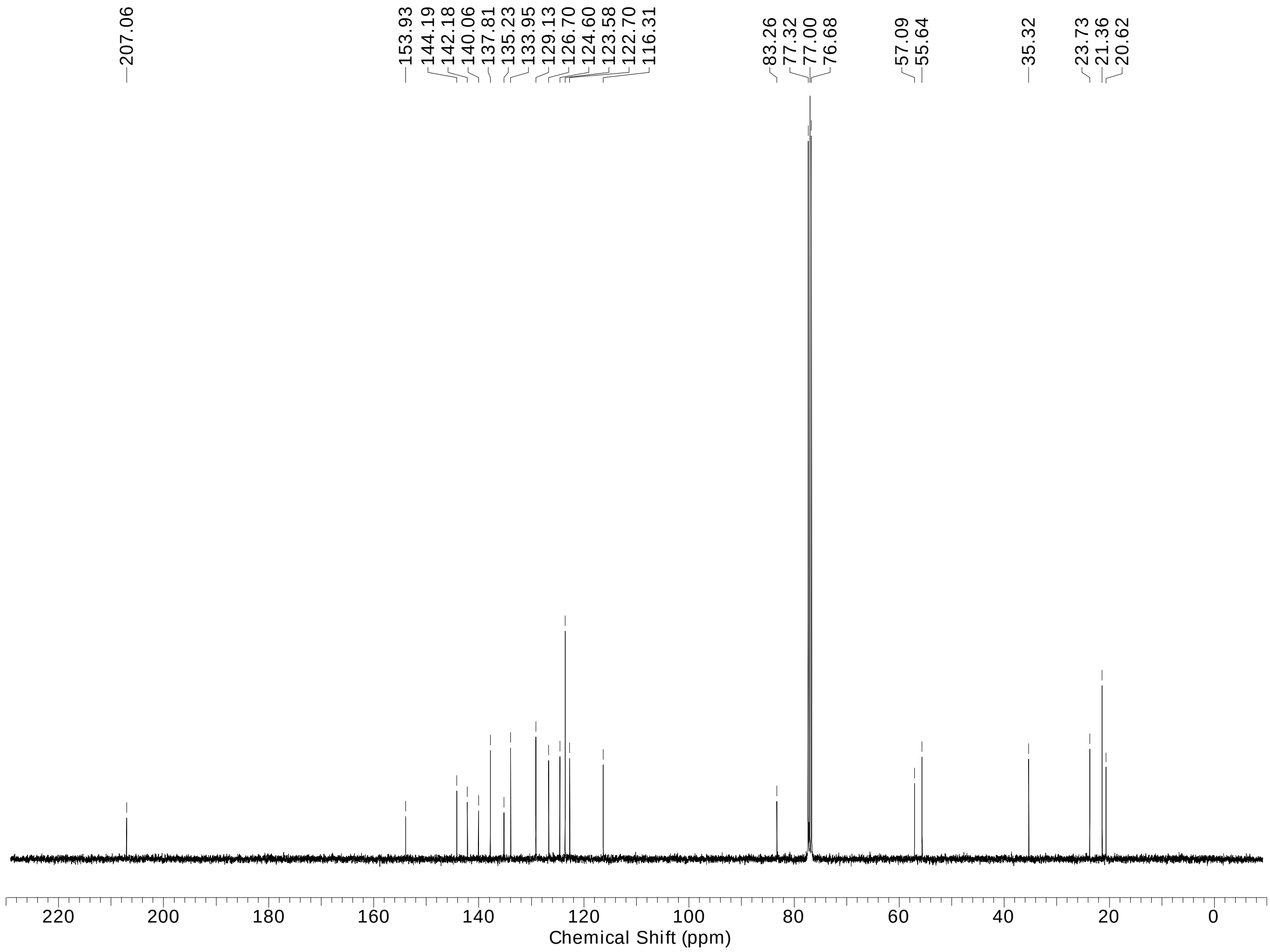
207.06
153.93
144.19
142.18
140.06
137.81
135.23
133.95
129.13
126.70
124.60
123.58
122.70
116.31
83.26
77.32
77.00
76.68
57.09
55.64
35.32
23.73
21.36
20.62
220
200
180
160
140
120
100
80
60
40
20
0
Chemical Shift (ppm)

NOESY

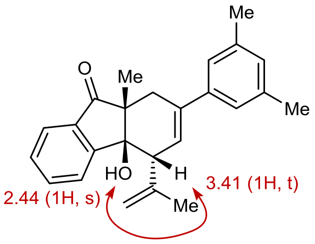

9ea

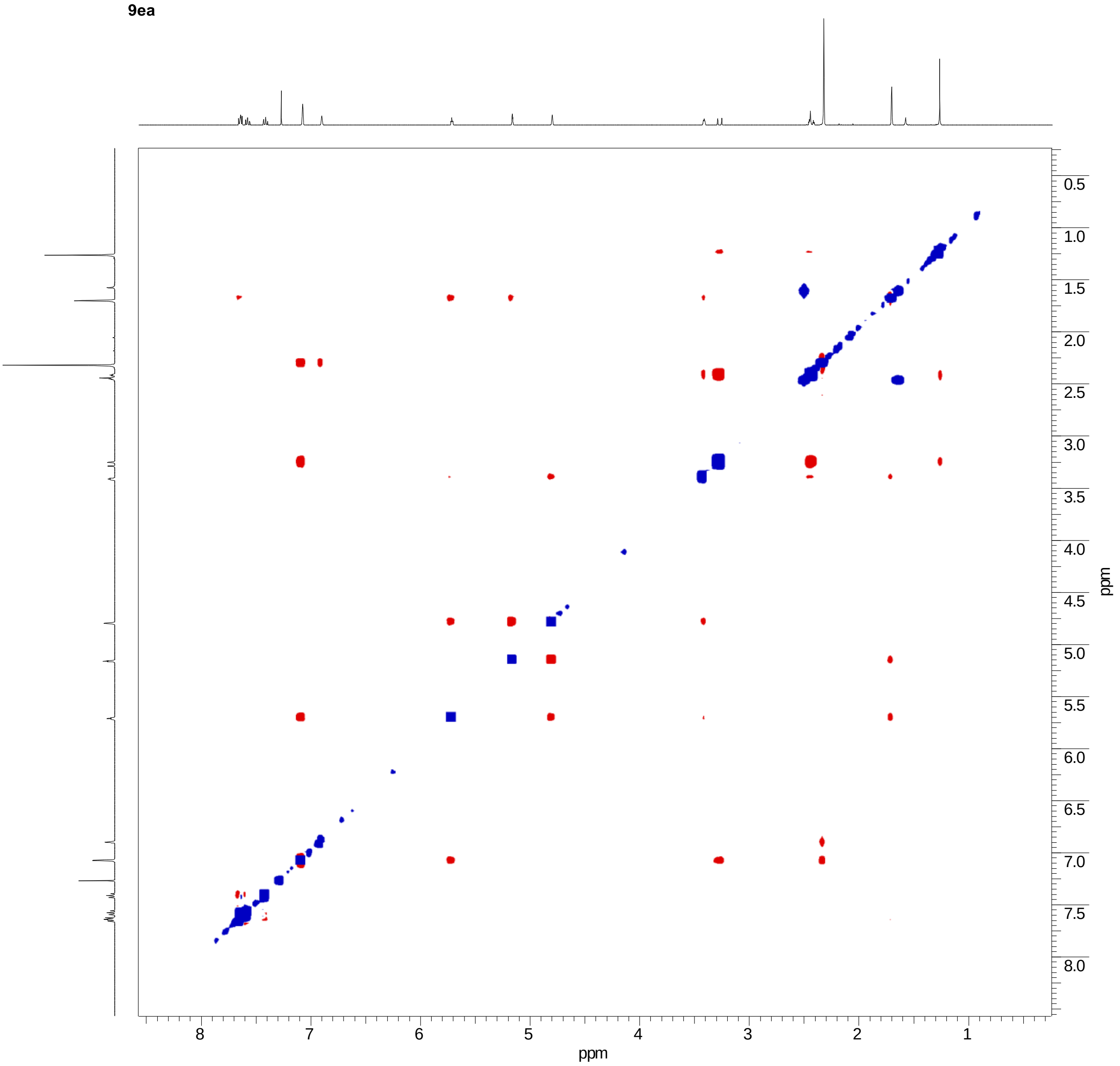

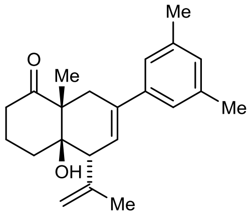
O
Me
Me
Me
OH
Me

9fa

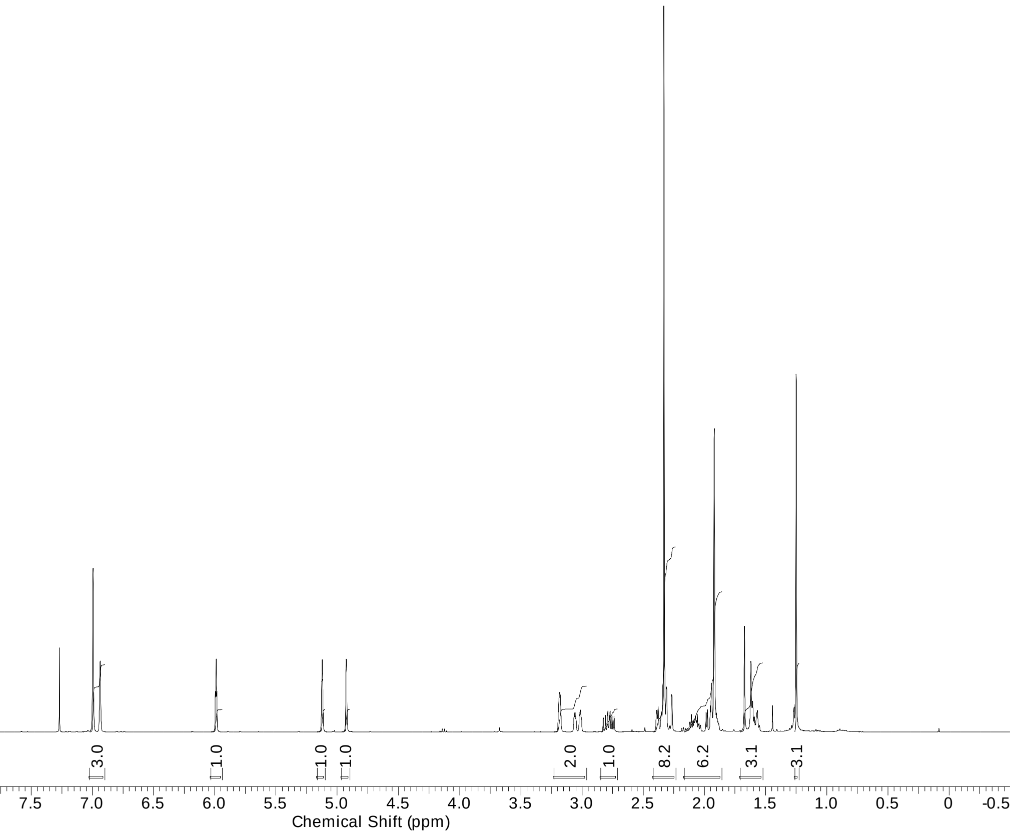
3.0
1.0
1.0
1.0
2.0
1.0
8.2
6.2
3.1
3.1
9.5 9.0 8.5 8.0 7.5 7.0 6.5 6.0 5.5 5.0 4.5 4.0 3.5 3.0 2.5 2.0 1.5 1.0 0.5 0 -0.5
Chemical Shift (ppm)

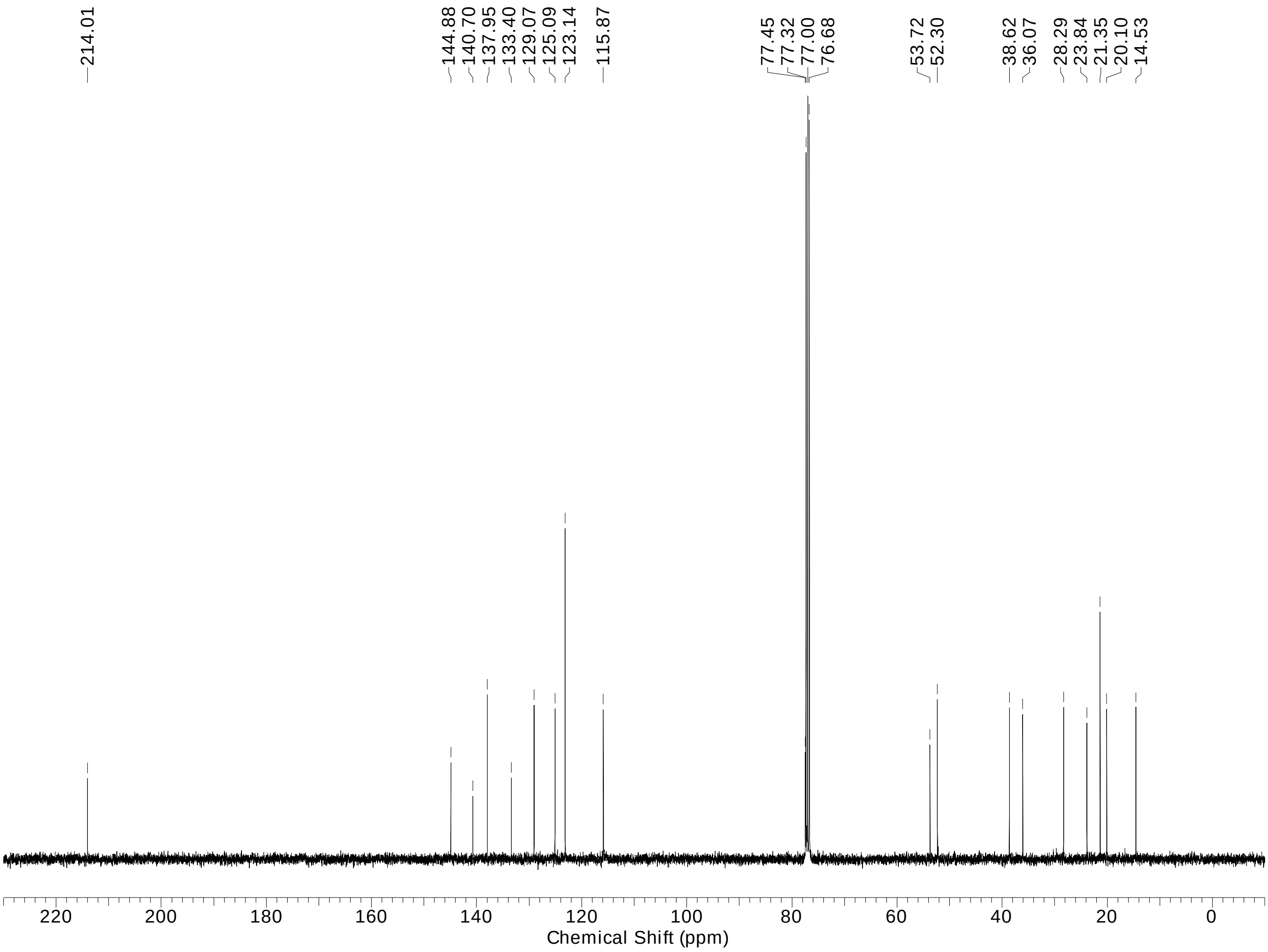
214.01
144.88
140.70
137.95
133.40
129.07
125.09
123.14
115.87
77.45
77.32
77.00
76.68
53.72
52.30
38.62
36.07
28.29
23.84
21.35
20.10
14.53
220 200 180 160 140 120 100 80 60 40 20 0
Chemical Shift (ppm)

NOESY

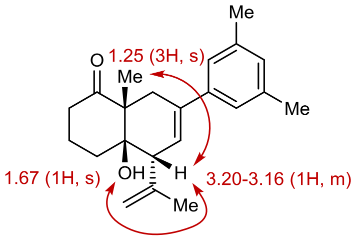
Me
O
1.25 (3H, s)
Me
Me
1.67 (1H, s)
OH
H
3.20-3.16 (1H, m)
Me

9fa

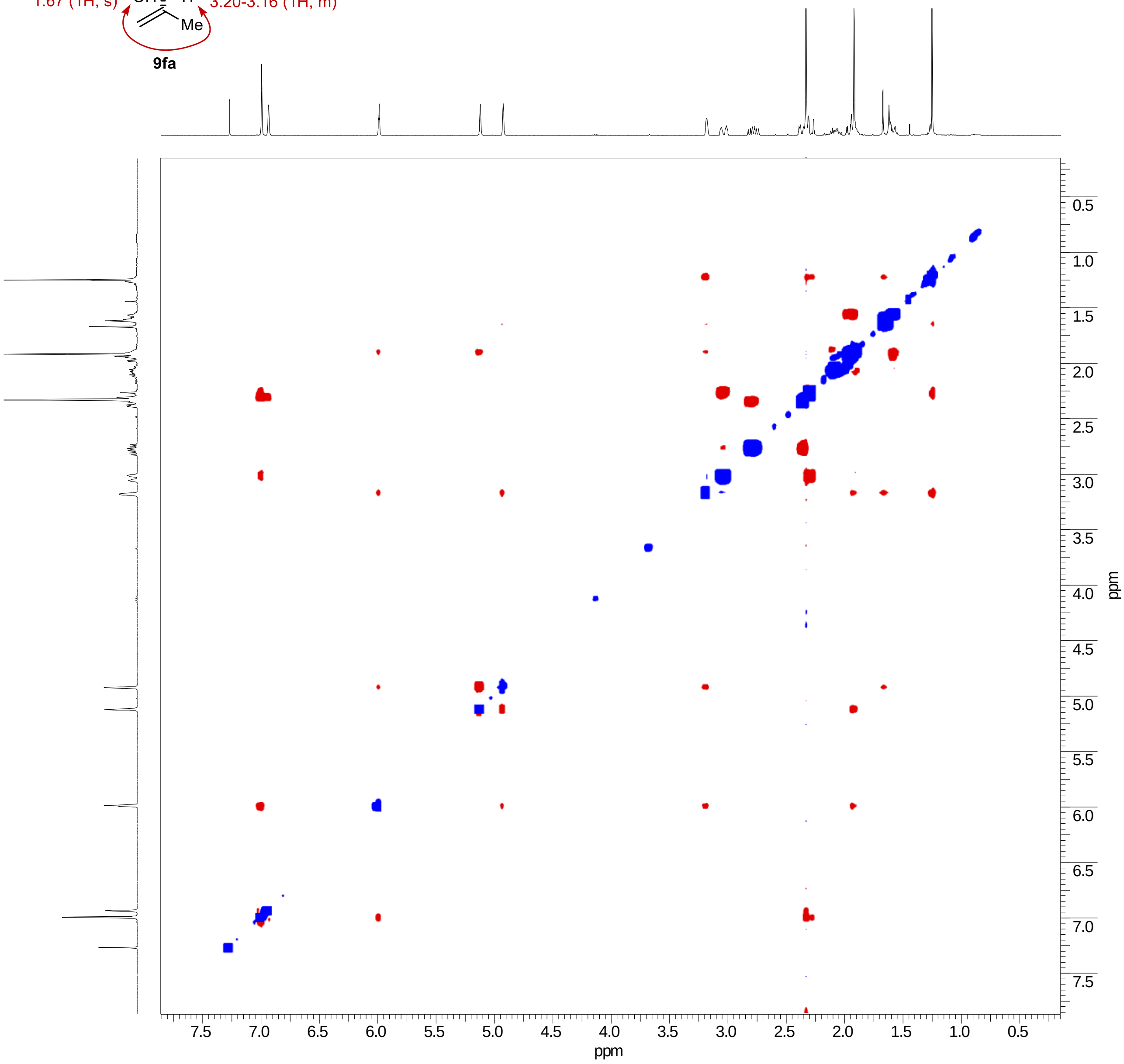
7.5 7.0 6.5 6.0 5.5 5.0 4.5 4.0 3.5 3.0 2.5 2.0 1.5 1.0 0.5
ppm
0.5 1.0 1.5 2.0 2.5 3.0 3.5 4.0 4.5 5.0 5.5 6.0 6.5 7.0 7.5
ppm

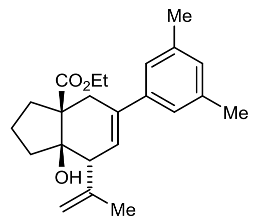
Me
CO_2Et
Me
OH
Me

11

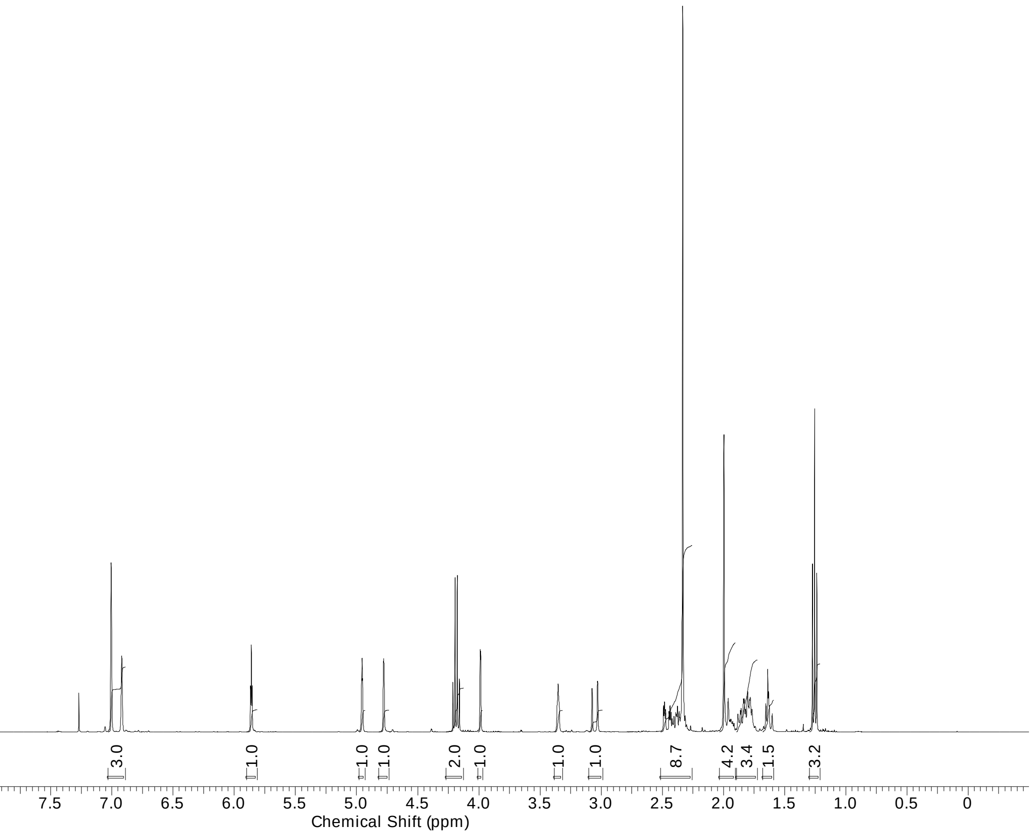
3.0
1.0
1.0
1.0
2.0
1.0
1.0
1.0
8.7
4.2
3.4
1.5
3.2
9.5 9.0 8.5 8.0 7.5 7.0 6.5 6.0 5.5 5.0 4.5 4.0 3.5 3.0 2.5 2.0 1.5 1.0 0.5 0
Chemical Shift (ppm)

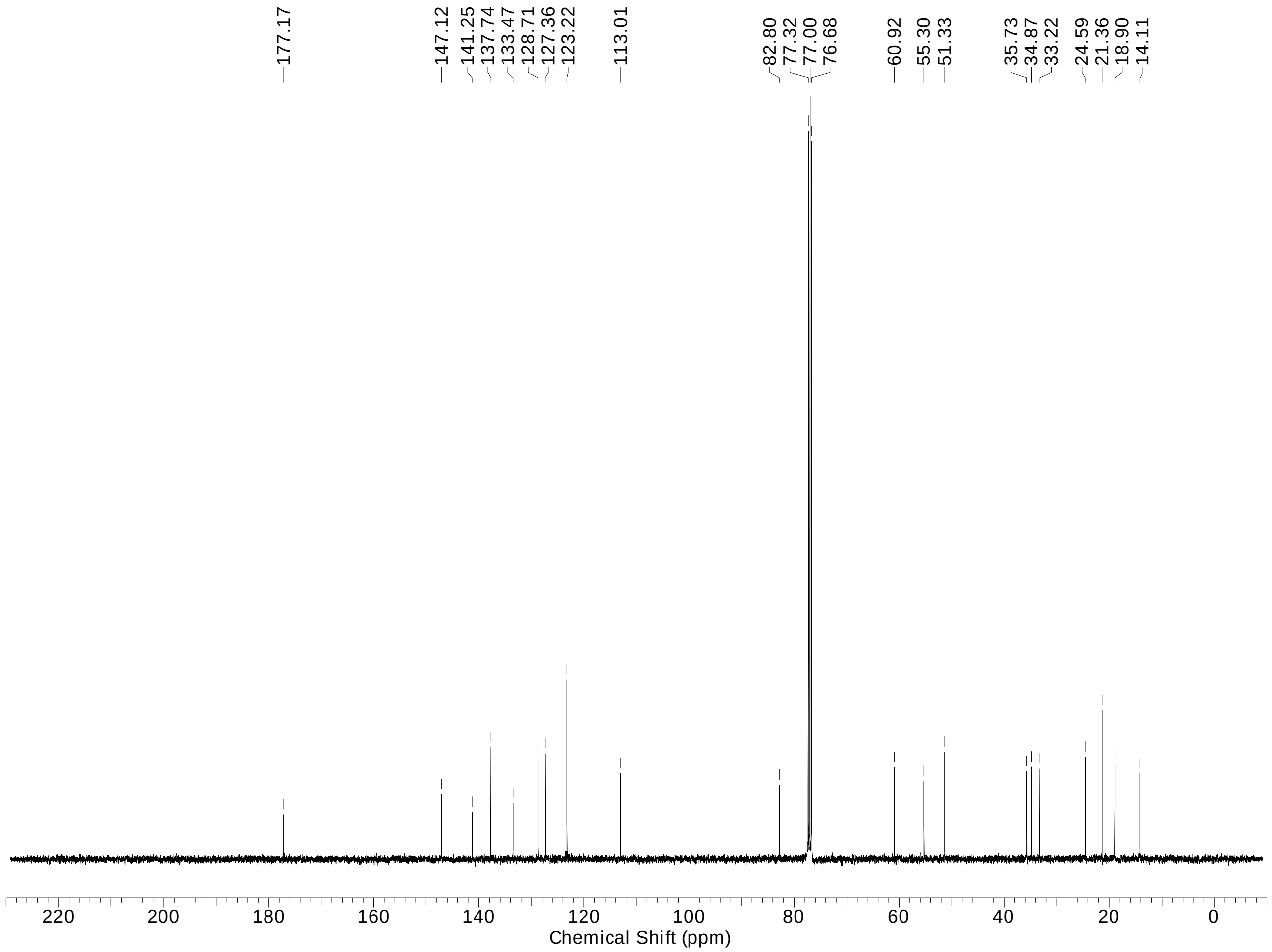
177.17
147.12
141.25
137.74
133.47
128.71
127.36
123.22
113.01
82.80
77.32
77.00
76.68
60.92
55.30
51.33
35.73
34.87
33.22
24.59
21.36
18.90
14.11
220 200 180 160 140 120 100 80 60 40 20 0
Chemical Shift (ppm)

NOESY

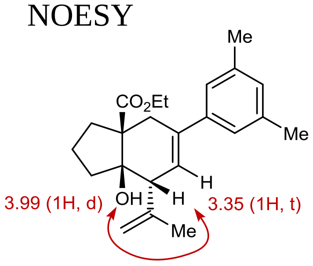

11

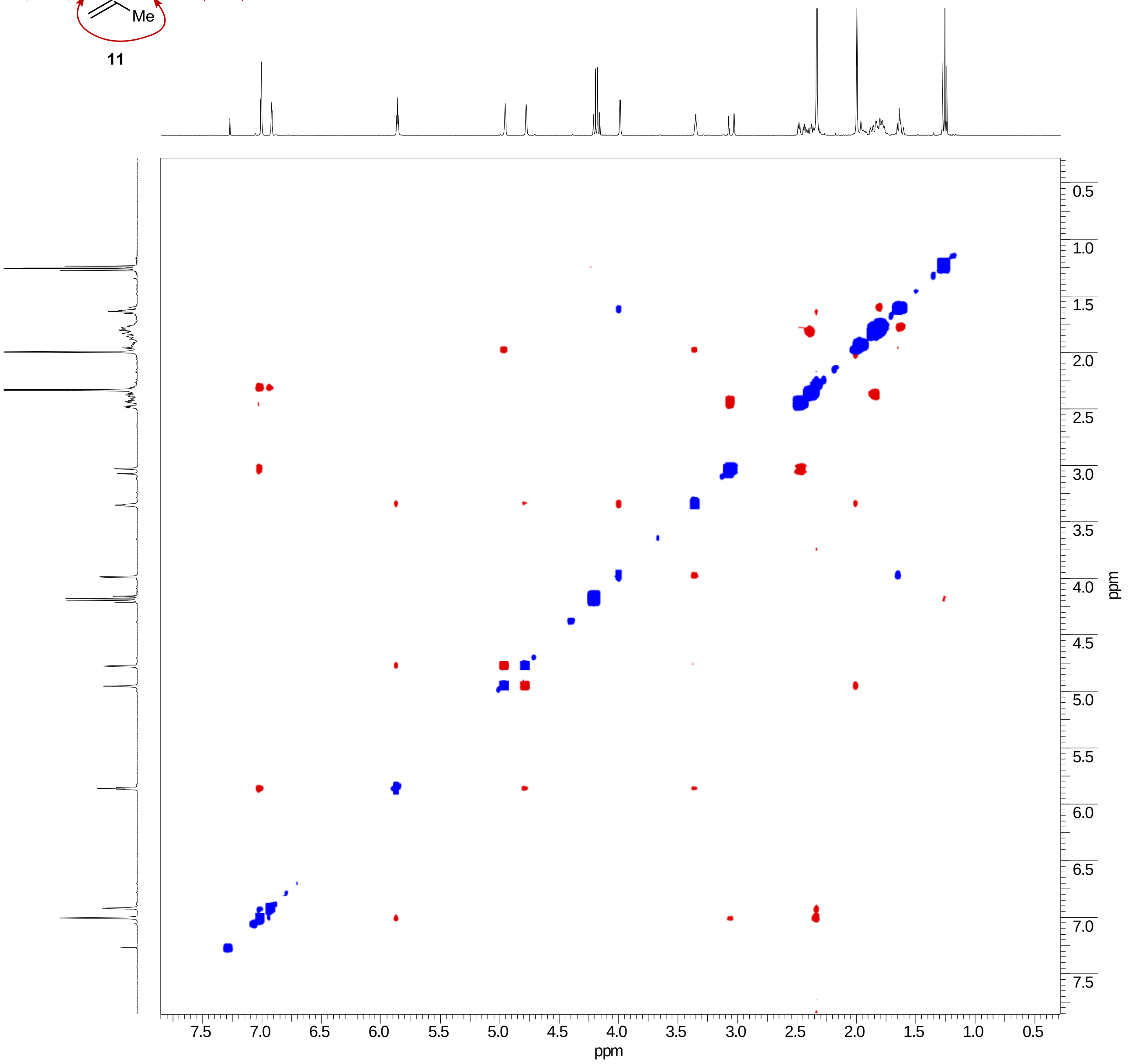

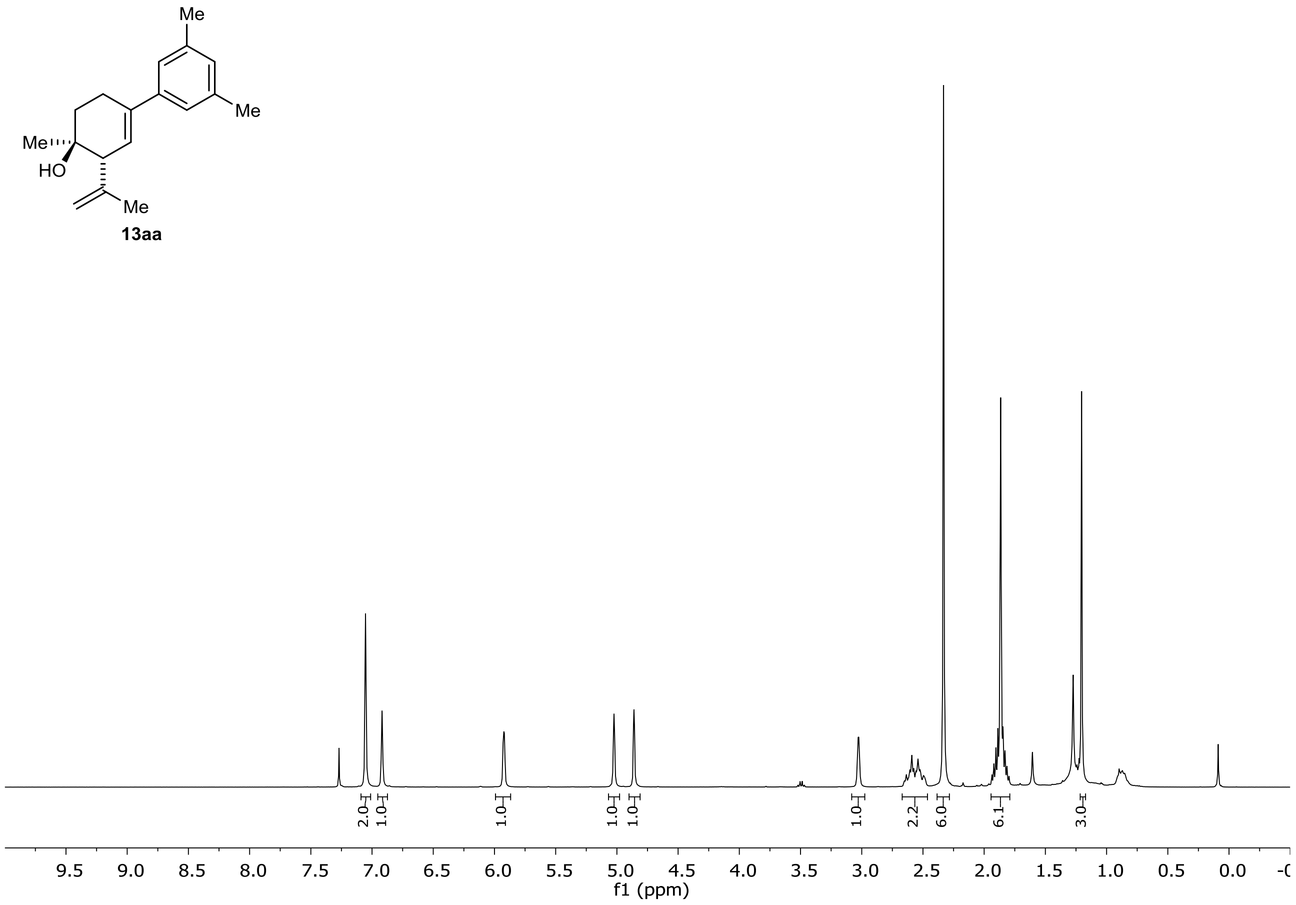
Me
Me
Me
HO
Me
2.0
1.0
1.0
1.0
1.0
1.0
2.2
6.0
6.1
3.0
f1 (ppm)

13aa

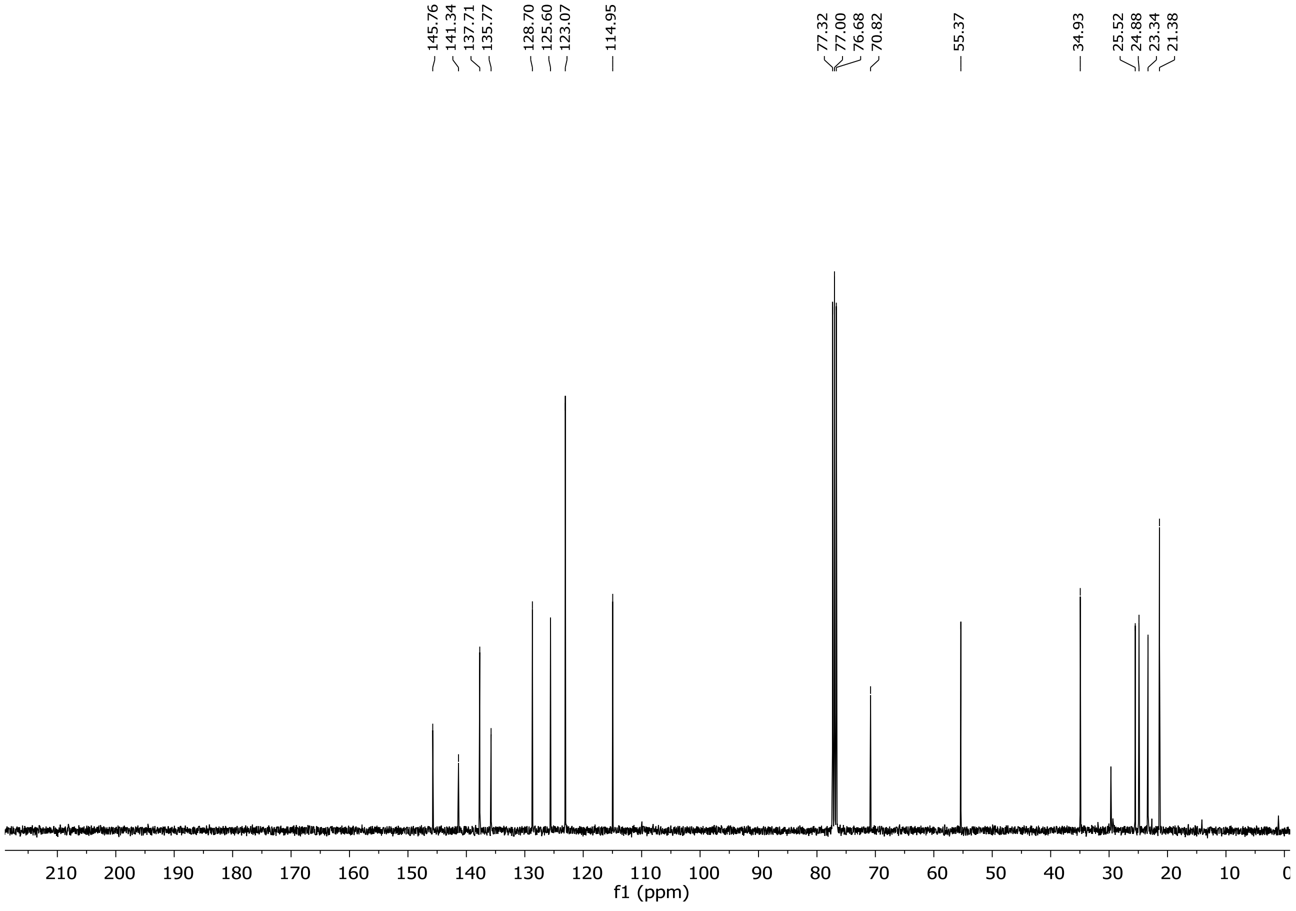
145.76
141.34
137.71
135.77
128.70
125.60
123.07
114.95
77.32
77.00
76.68
70.82
55.37
34.93
25.52
24.88
23.34
21.38
f1 (ppm)

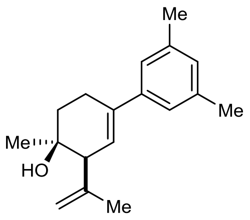
Me
Me
Me
HO
Me

13ab

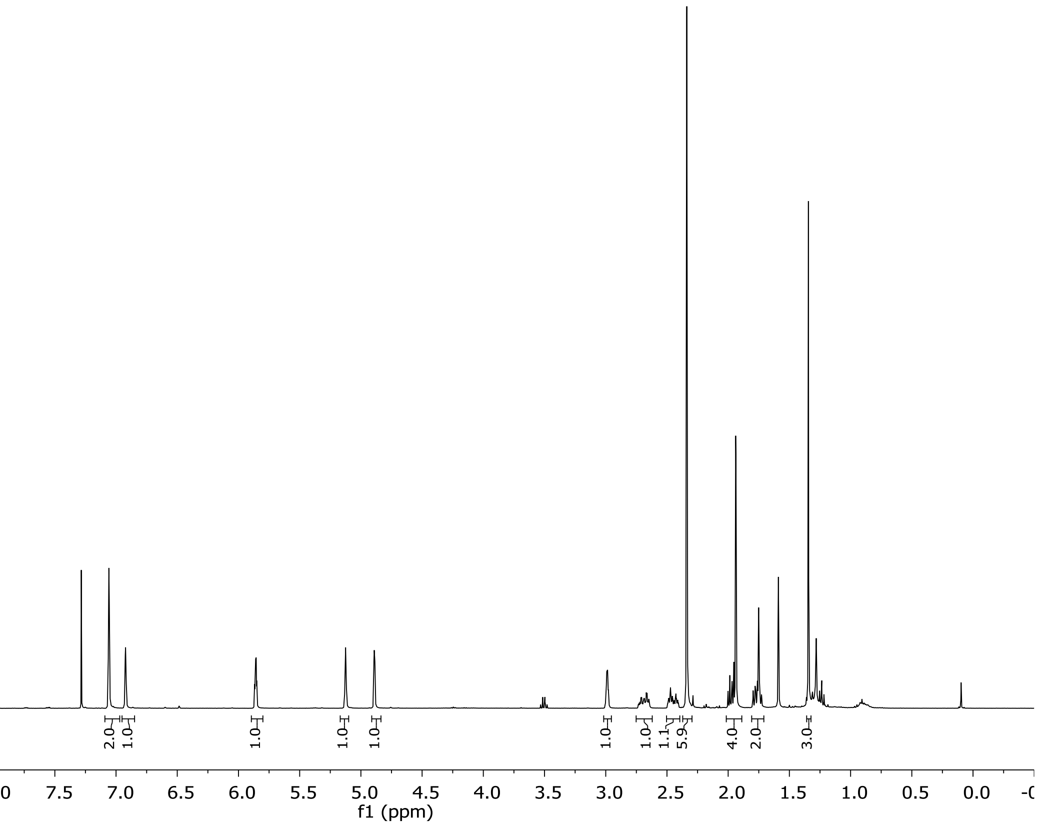
2.0
1.0
1.0
1.0
1.0
1.0
1.0
1.1
5.9
4.0
2.0
3.0
f1 (ppm)

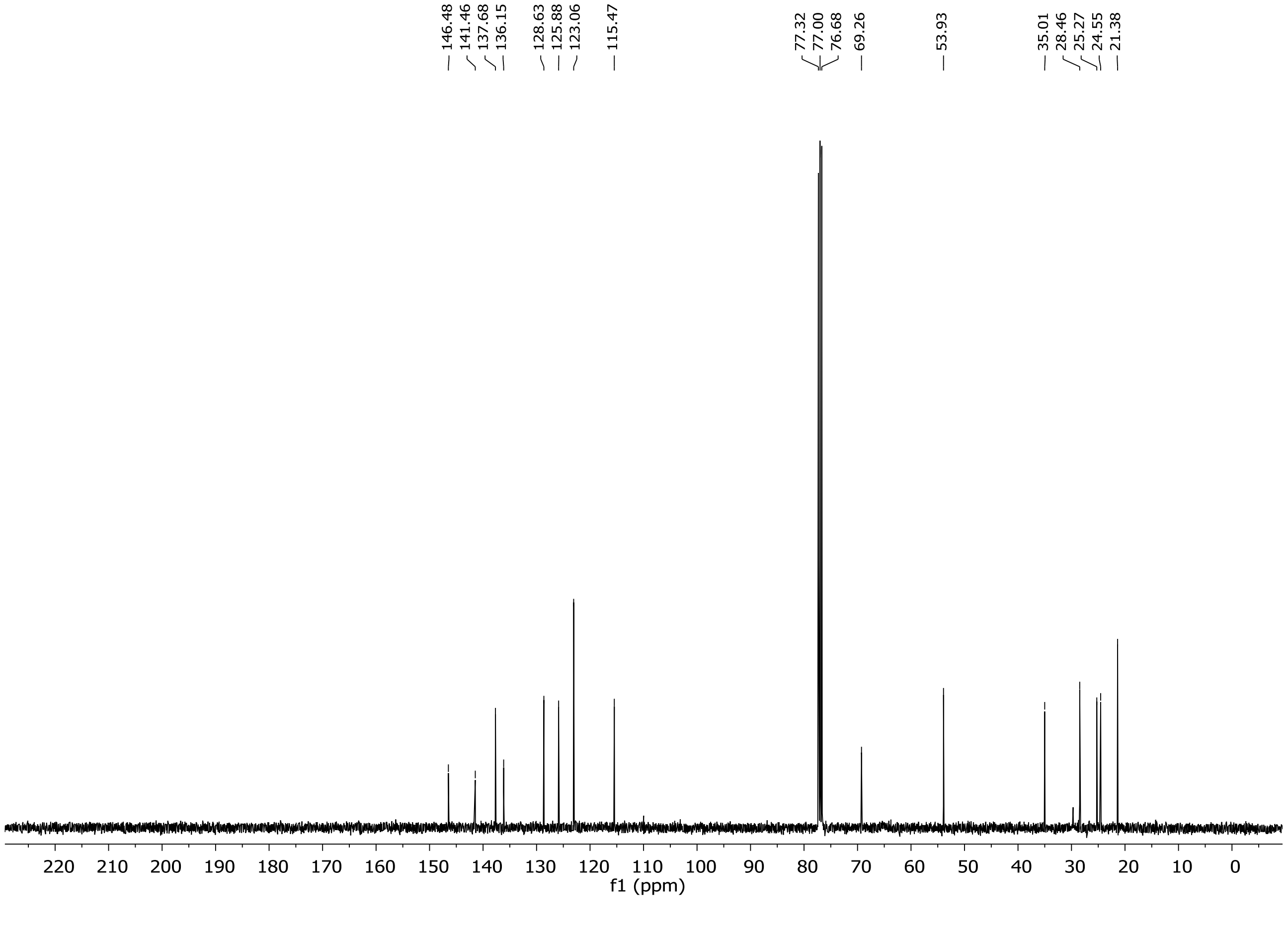
146.48
141.46
137.68
136.15
128.63
125.88
123.06
115.47
77.32
77.00
76.68
69.26
53.93
35.01
28.46
25.27
24.55
21.38
f1 (ppm)

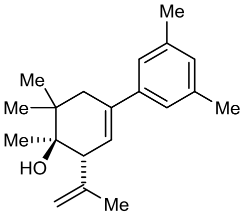
Me
Me
Me
Me
Me
HO
Me

13ba

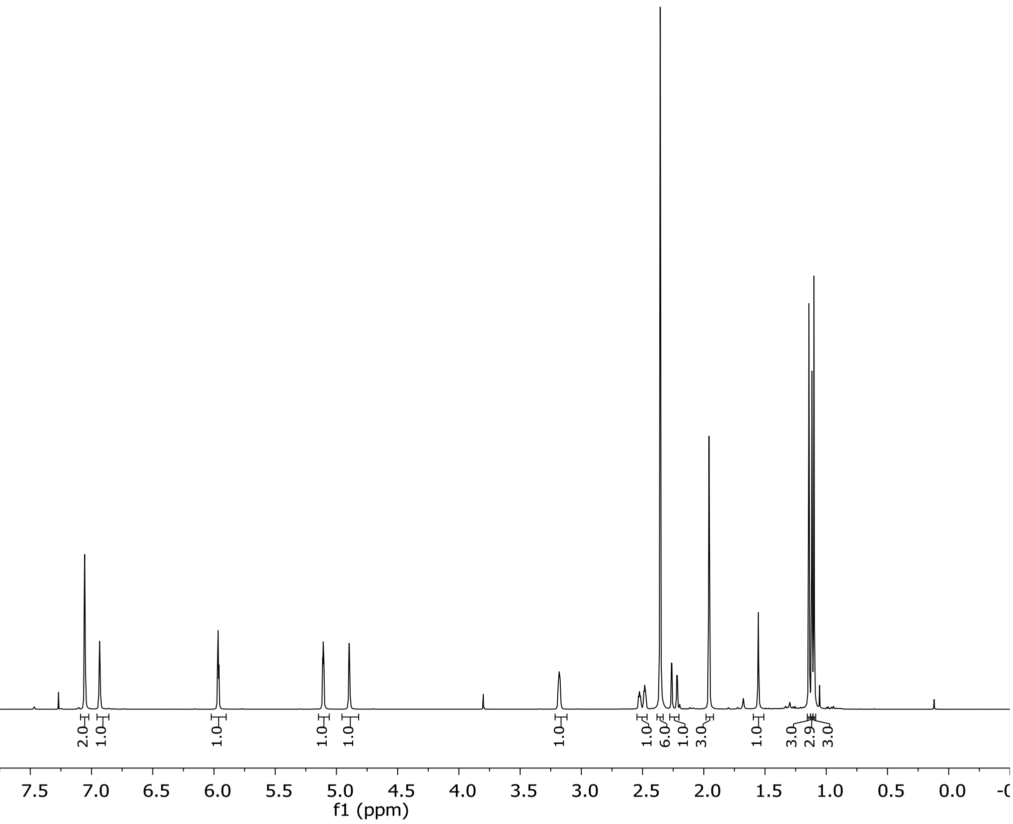
2.0
1.0
1.0
1.0
1.0
1.0
1.0
6.0
1.0
3.0
1.0
3.0
2.9
3.0
9.5 9.0 8.5 8.0 7.5 7.0 6.5 6.0 5.5 5.0 4.5 4.0 3.5 3.0 2.5 2.0 1.5 1.0 0.5 0.0
f1 (ppm)

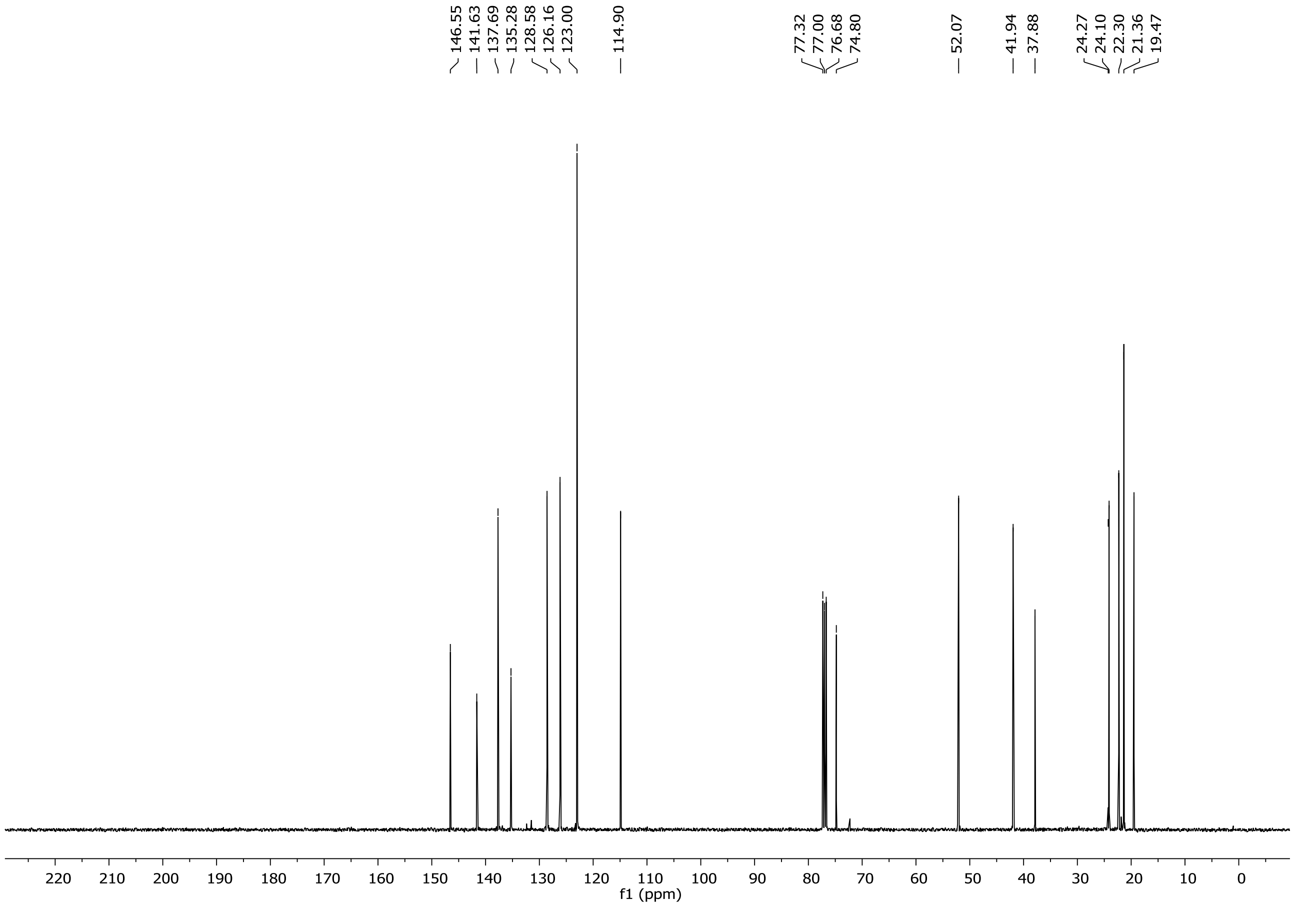
146.55
141.63
137.69
135.28
128.58
126.16
123.00
114.90
77.32
77.00
76.68
74.80
52.07
41.94
37.88
24.27
24.10
22.30
21.36
19.47
220 210 200 190 180 170 160 150 140 130 120 110 100 90 80 70 60 50 40 30 20 10 0
f1 (ppm)

NOESY

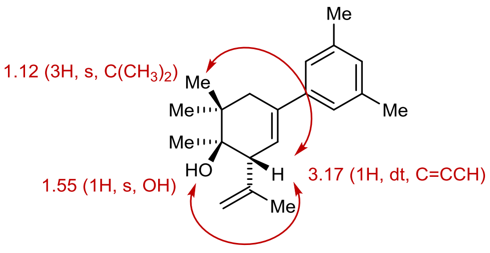
Me
1.12 (3H, s, C(CH3)2)
Me
Me
Me
Me
HO
H
1.55 (1H, s, OH)
3.17 (1H, dt, C=CCH)
Me

13ba

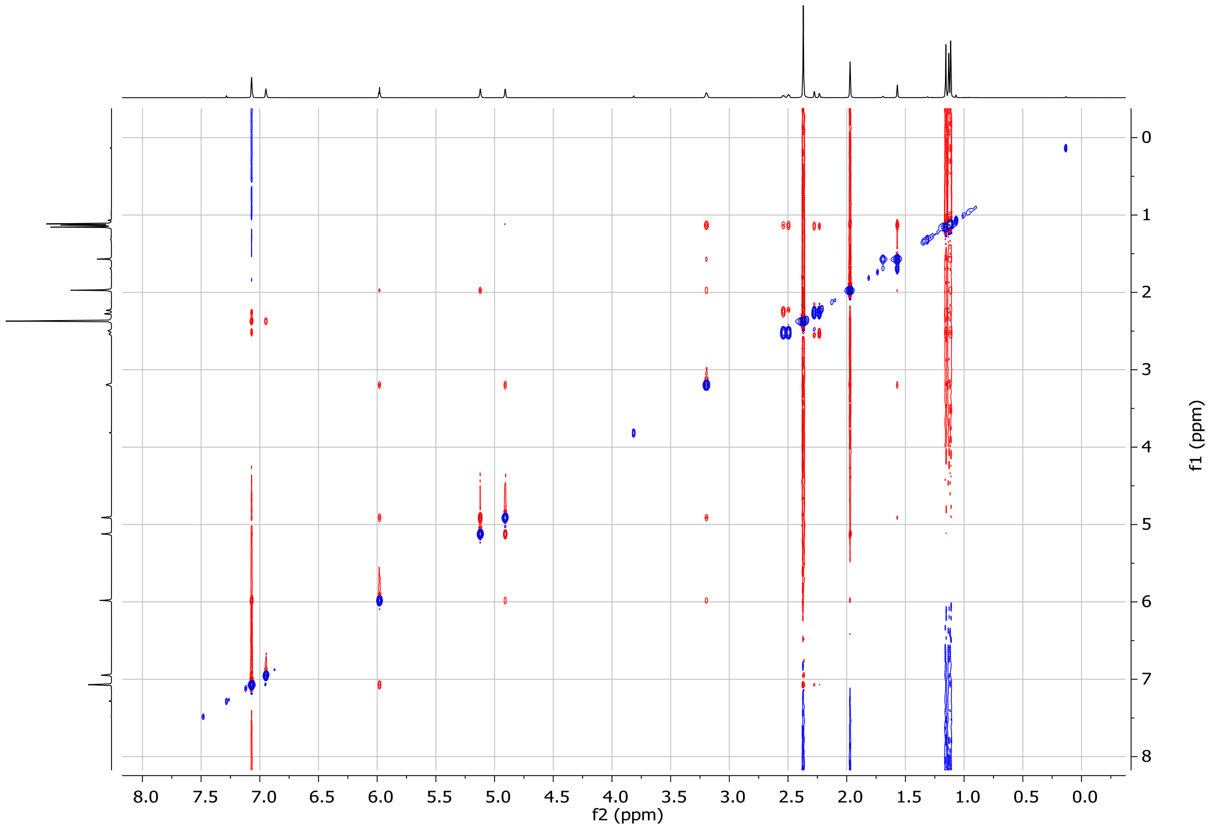
f2 (ppm)
f1 (ppm)

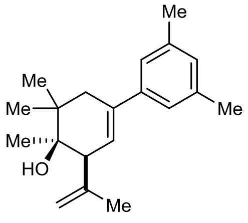
Me
Me
Me
Me
Me
HO
Me

13bb

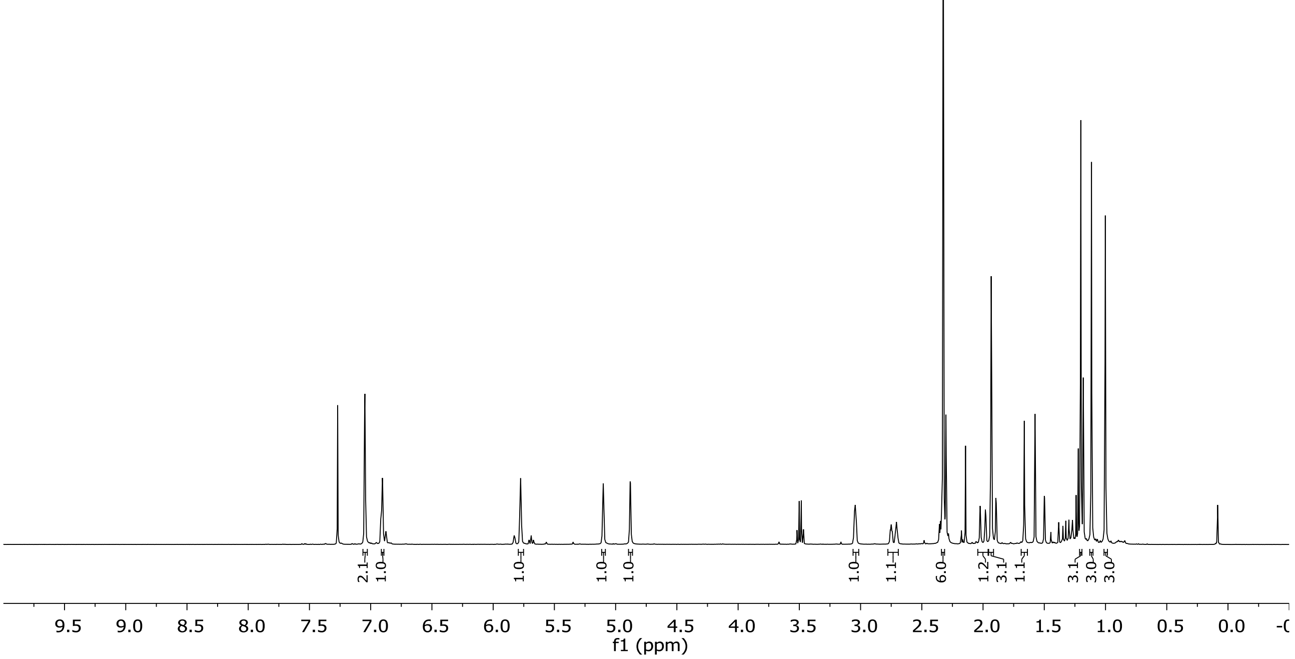
2.1
1.0
1.0
1.0
1.0
1.0
1.1
6.0
1.2
3.1
1.1
3.1
3.0
3.0
9.5 9.0 8.5 8.0 7.5 7.0 6.5 6.0 5.5 5.0 4.5 4.0 3.5 3.0 2.5 2.0 1.5 1.0 0.5 0.0
f1 (ppm)

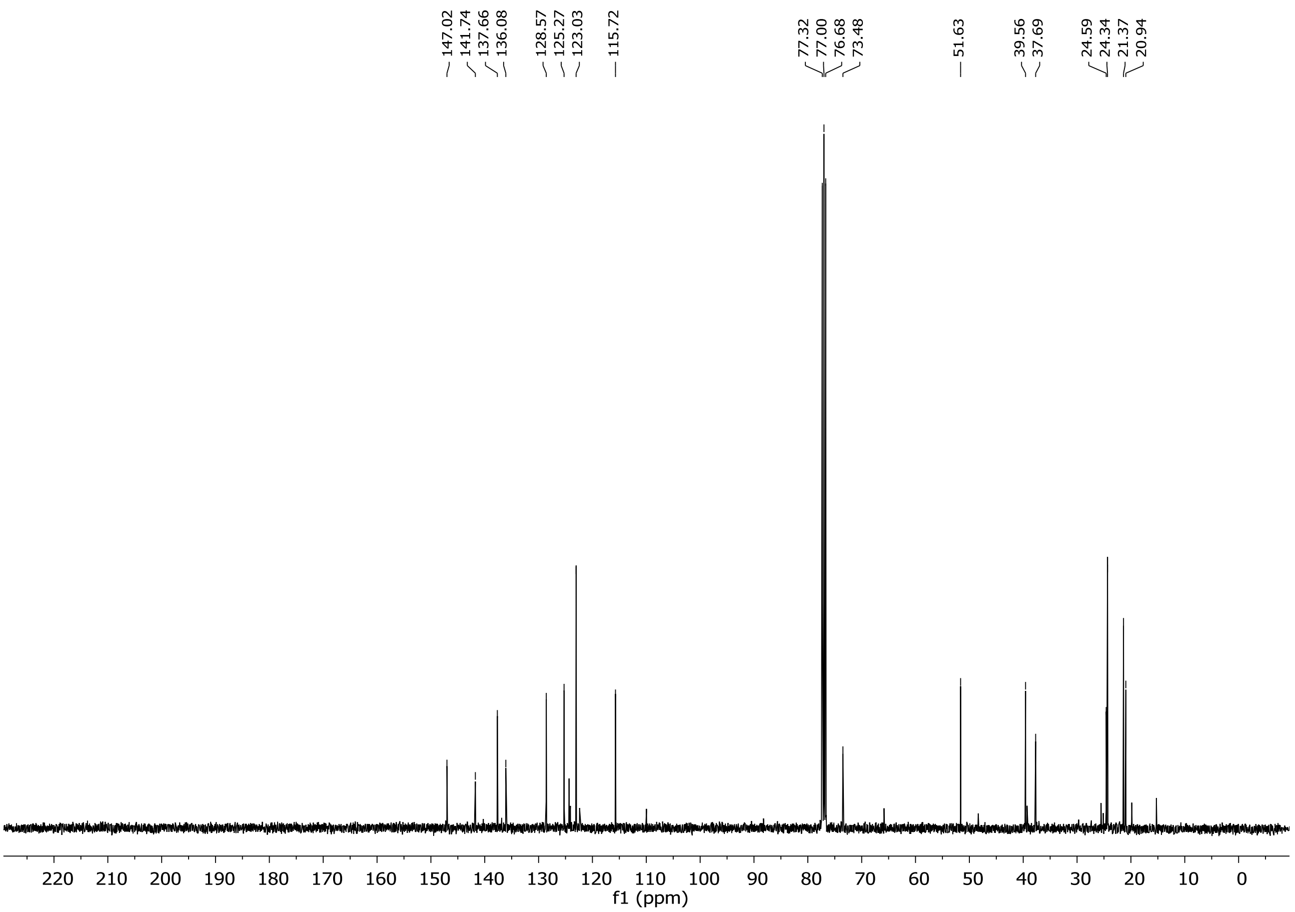
147.02
141.74
137.66
136.08
128.57
125.27
123.03
115.72
77.32
77.00
76.68
73.48
51.63
39.56
37.69
24.59
24.34
21.37
20.94
220 210 200 190 180 170 160 150 140 130 120 110 100 90 80 70 60 50 40 30 20 10 0
f1 (ppm)

NOESY

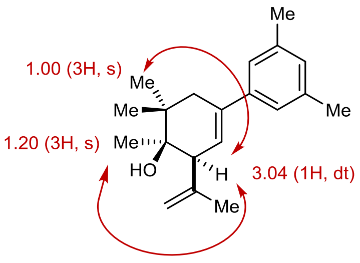

13bb

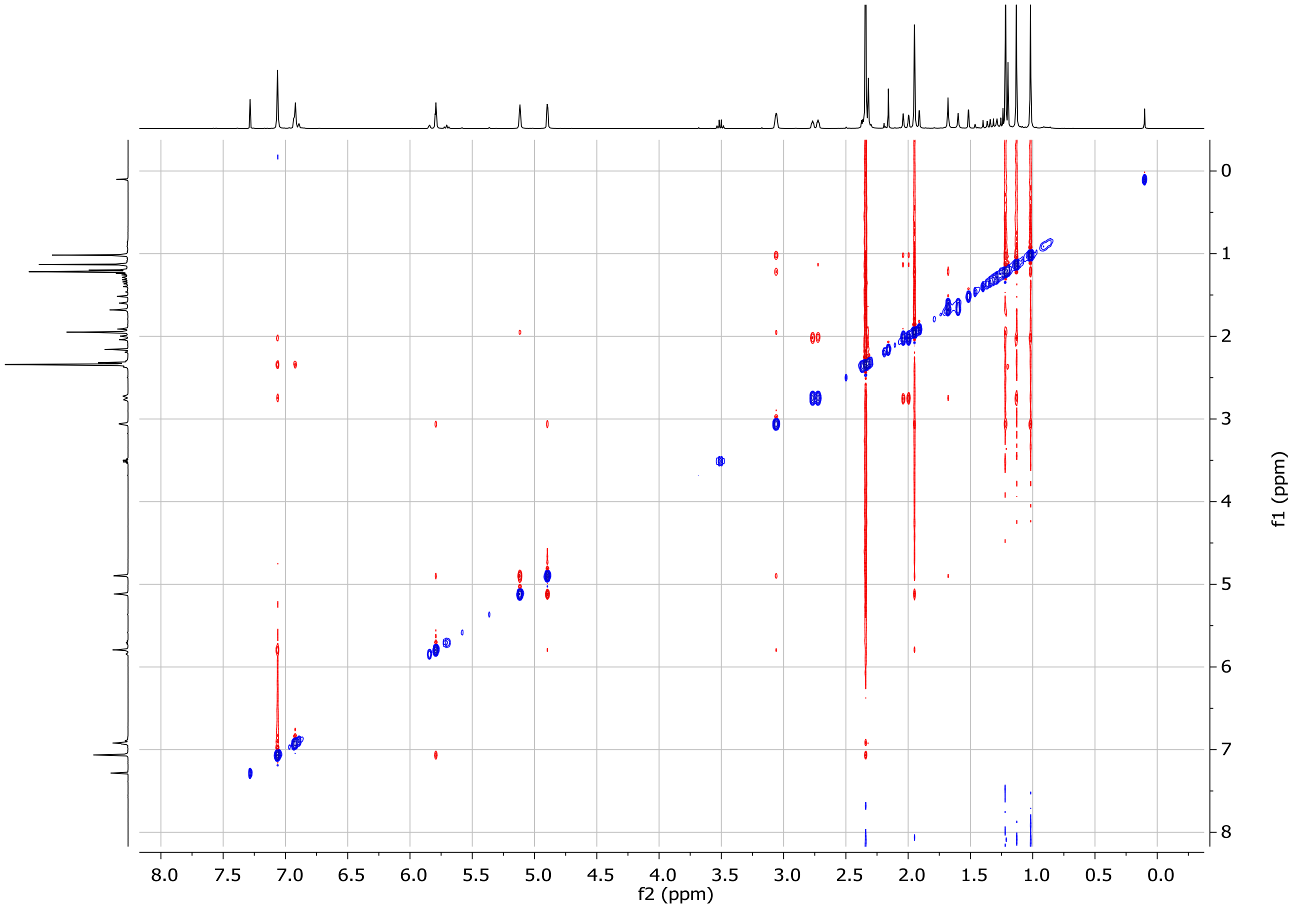

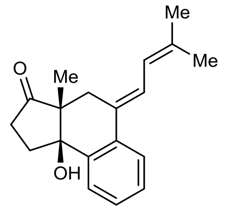
Me
Me
O
Me
OH

15

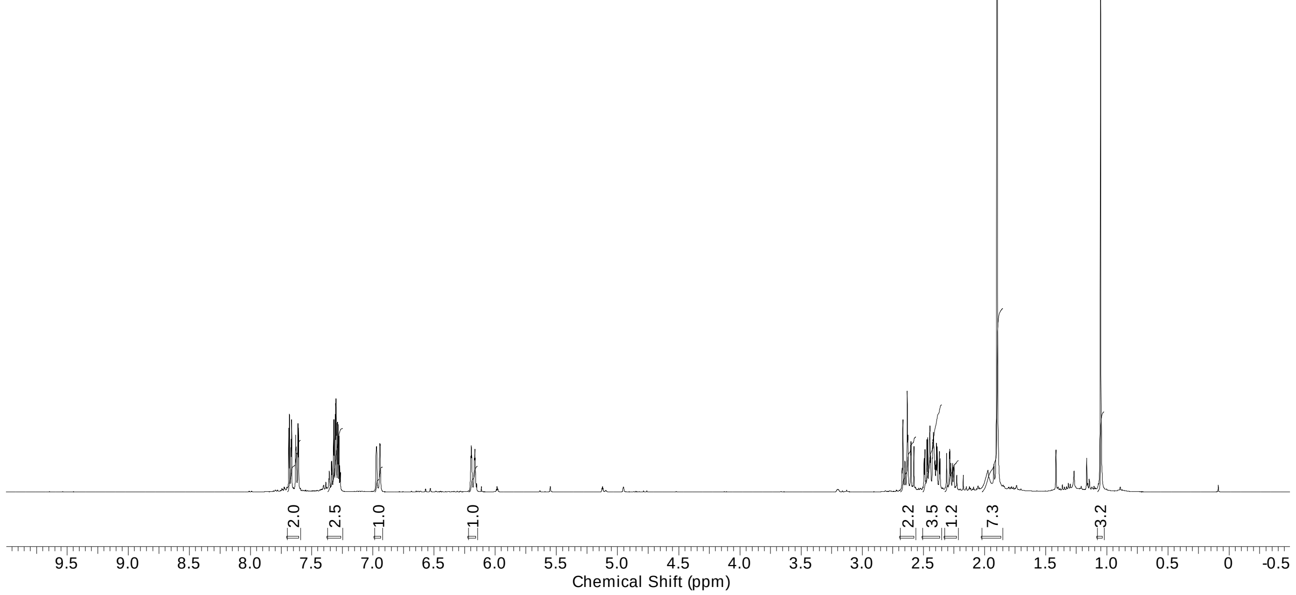
2.0
2.5
1.0
1.0
2.2
3.5
1.2
7.3
3.2
9.5 9.0 8.5 8.0 7.5 7.0 6.5 6.0 5.5 5.0 4.5 4.0 3.5 3.0 2.5 2.0 1.5 1.0 0.5 0 -0.5
Chemical Shift (ppm)

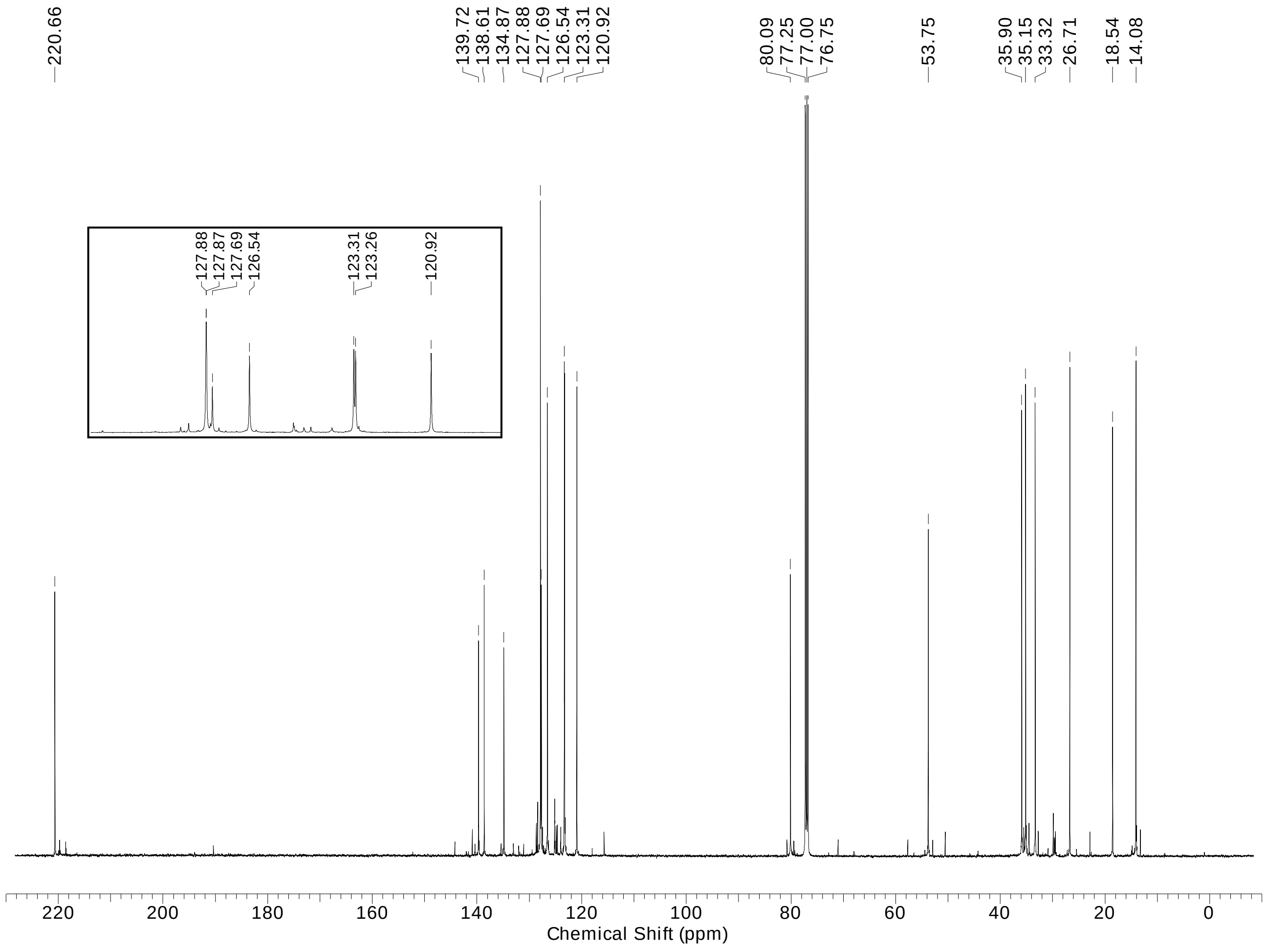
220.66
139.72
138.61
134.87
127.88
127.69
126.54
123.31
120.92
80.09
77.25
77.00
76.75
53.75
35.90
35.15
33.32
26.71
18.54
14.08
127.88
127.87
127.69
126.54
123.31
123.26
120.92
220 200 180 160 140 120 100 80 60 40 20 0
Chemical Shift (ppm)

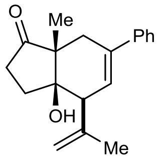
O
Me
Ph
OH
Me

2jb

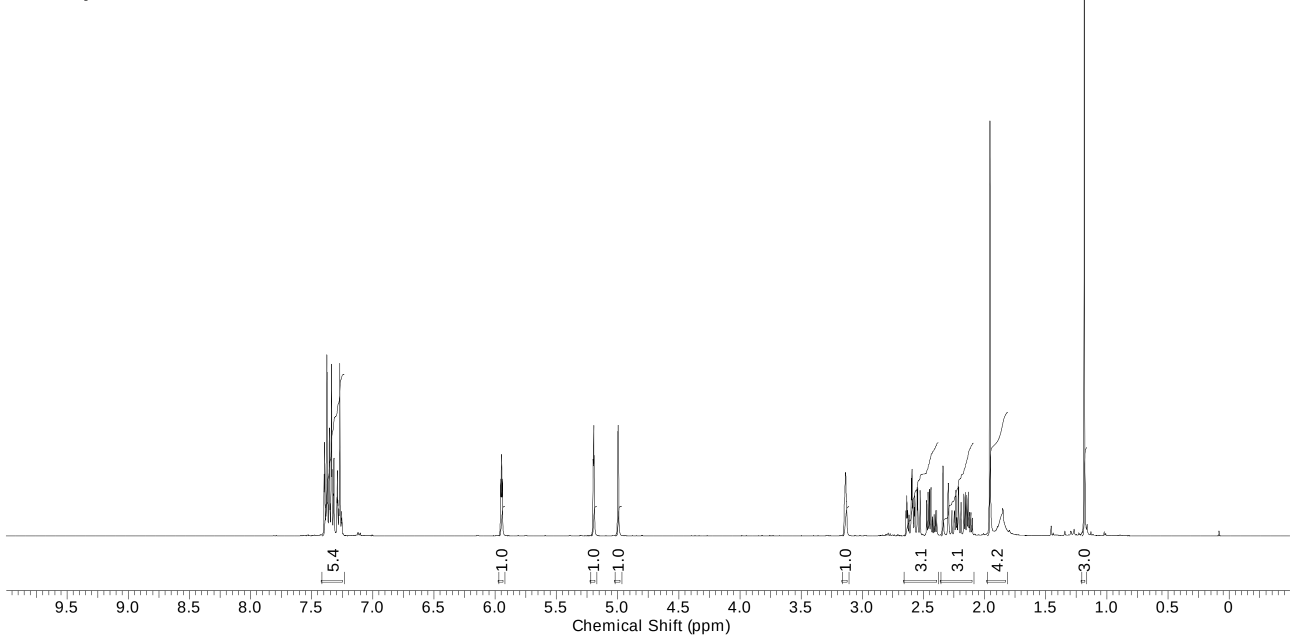
5.4
1.0
1.0
1.0
1.0
3.1
3.1
4.2
3.0
9.5 9.0 8.5 8.0 7.5 7.0 6.5 6.0 5.5 5.0 4.5 4.0 3.5 3.0 2.5 2.0 1.5 1.0 0.5 0
Chemical Shift (ppm)

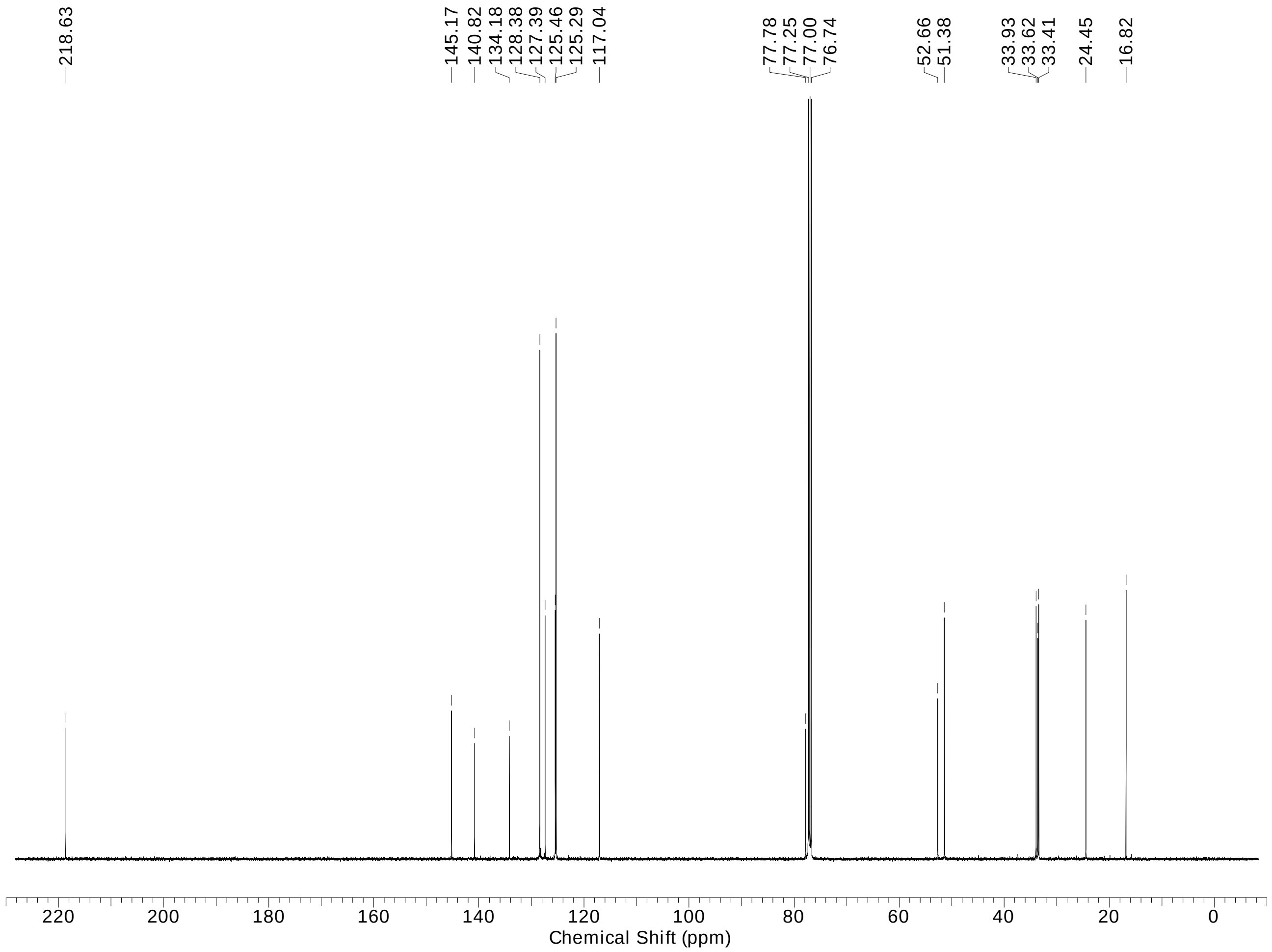
218.63
145.17
140.82
134.18
128.38
127.39
125.46
125.29
117.04
77.78
77.25
77.00
76.74
52.66
51.38
33.93
33.62
33.41
24.45
16.82
220 200 180 160 140 120 100 80 60 40 20 0
Chemical Shift (ppm)

NOESY

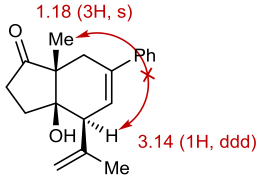

2jb

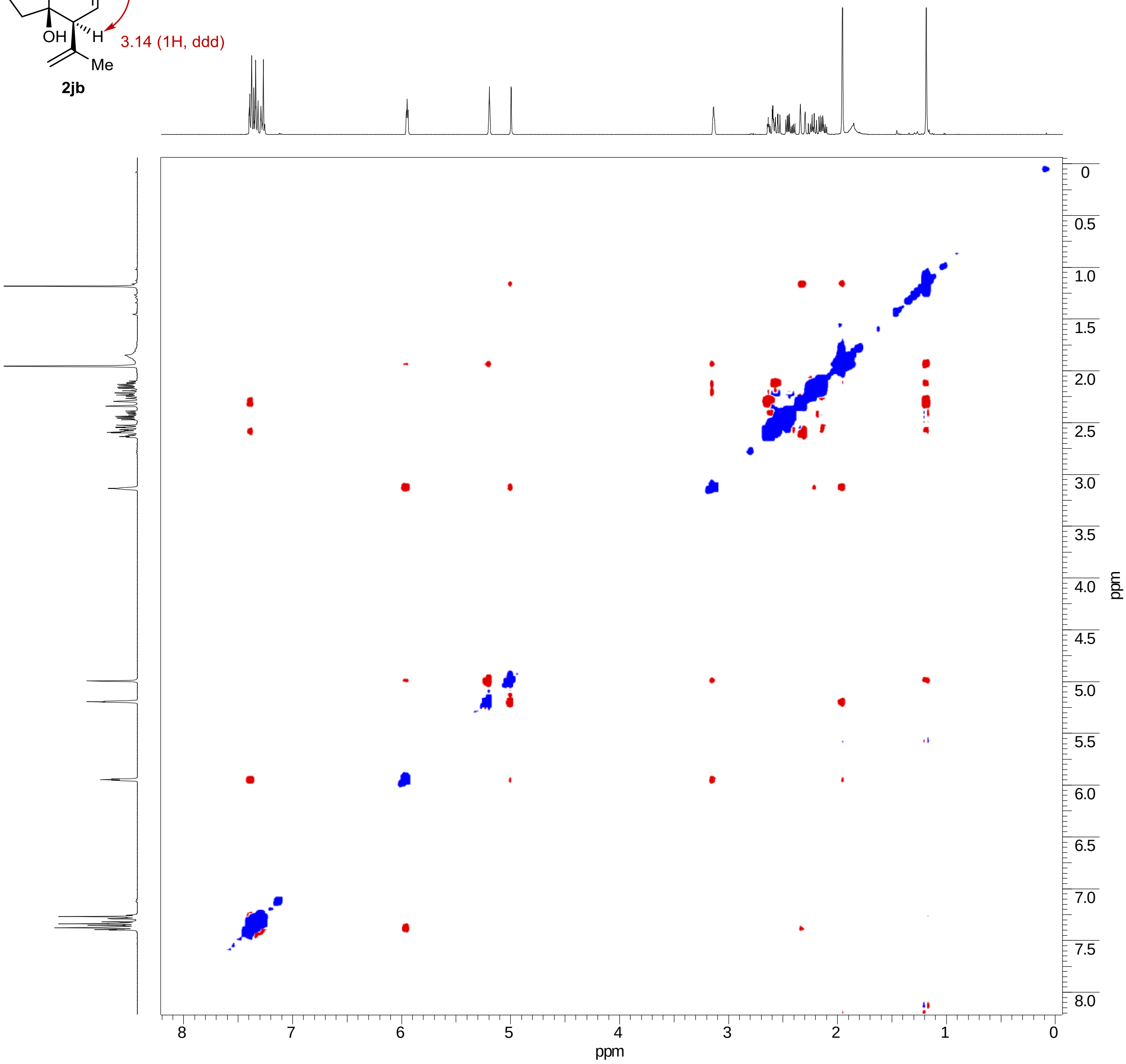

This NOESY NMR spectrum does not constitute strong proof of the relative configuration of **2jb**. The relative configuration of **2jb** was assigned tentatively by analogy with **9cb** (NOESY NMR spectrum on page 82), the relative configuration of which was determined by X-ray crystallography (see page 21).

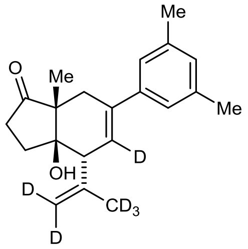
Me
O
Me
Me
D
OH
D
CD_3
D

$[D]_6$-**2aa**

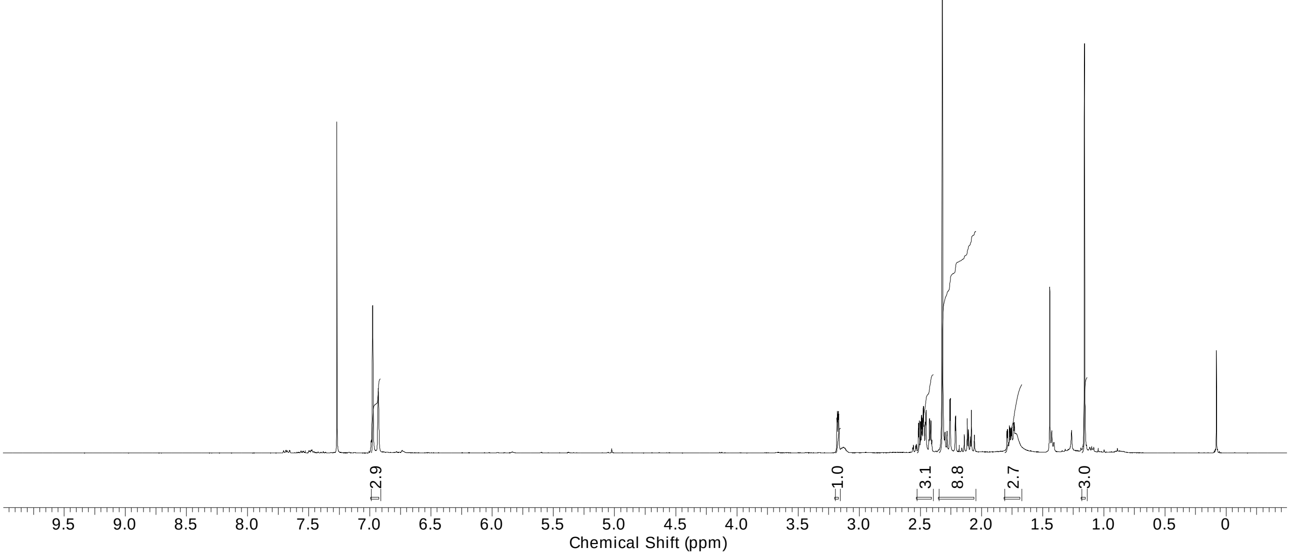
2.9
1.0
3.1
8.8
2.7
3.0
9.5 9.0 8.5 8.0 7.5 7.0 6.5 6.0 5.5 5.0 4.5 4.0 3.5 3.0 2.5 2.0 1.5 1.0 0.5 0
Chemical Shift (ppm)

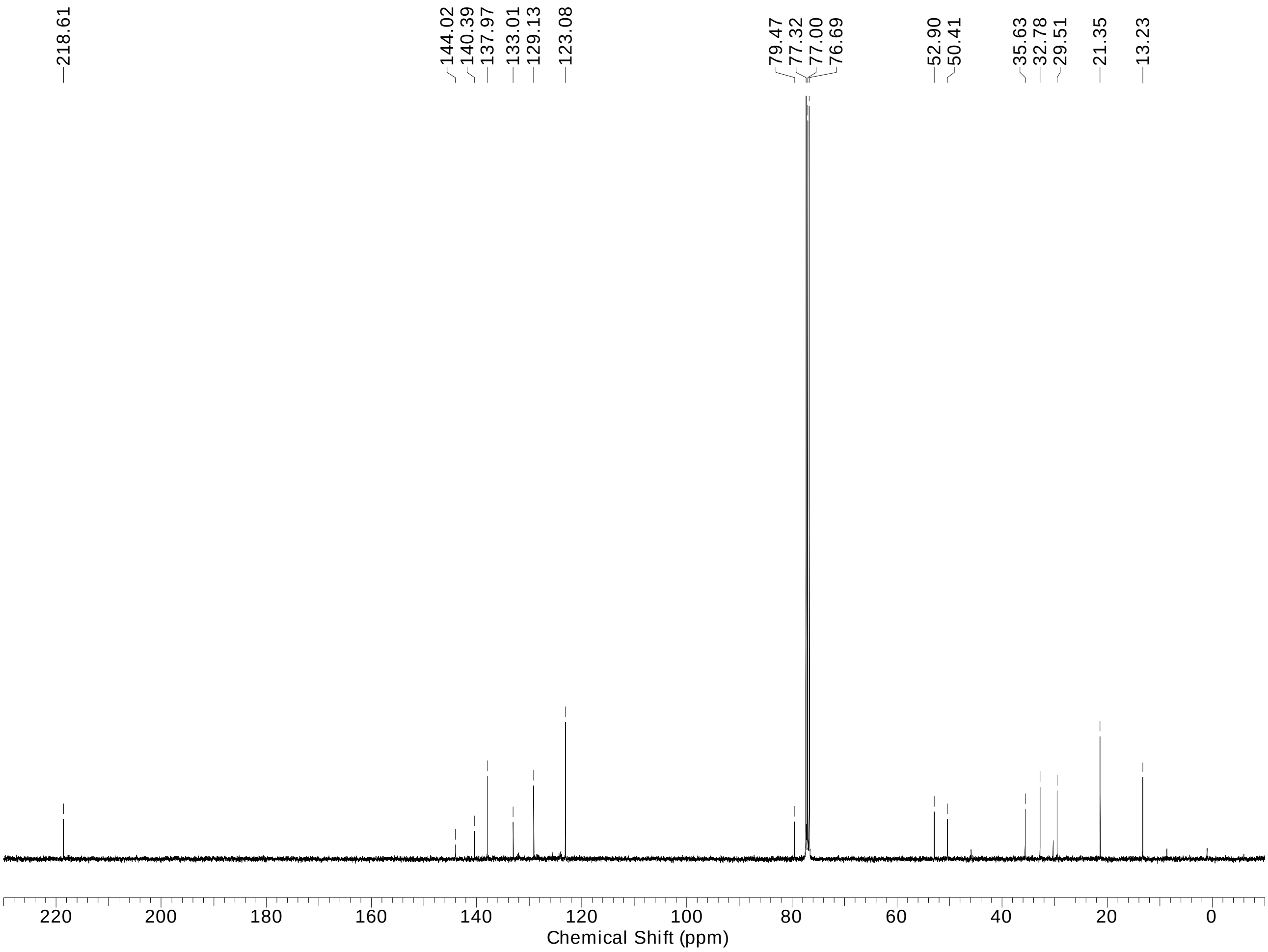
218.61
144.02
140.39
137.97
133.01
129.13
123.08
79.47
77.32
77.00
76.69
52.90
50.41
35.63
32.78
29.51
21.35
13.23
220 200 180 160 140 120 100 80 60 40 20 0
Chemical Shift (ppm)

^{2}H NMR spectrum

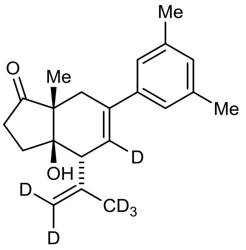

[D]$_6$-**2aa**

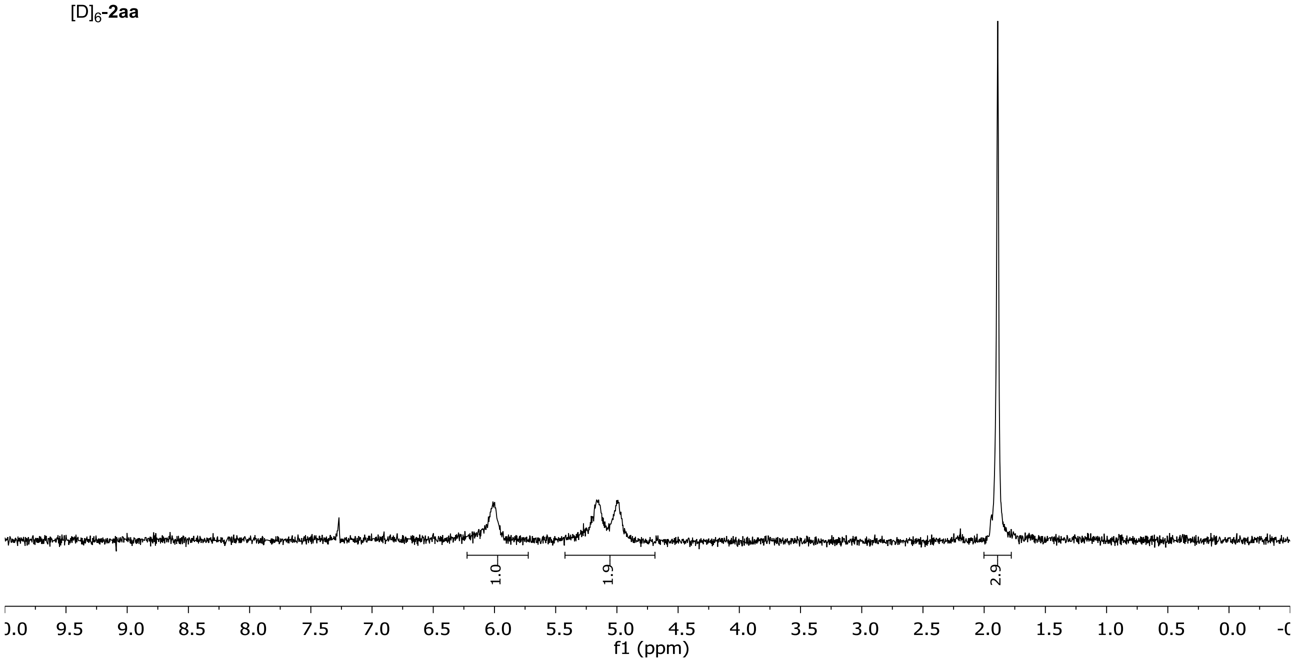

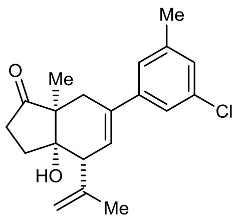
Me
O
Me
Cl
HO
Me

(+)–**2bb**

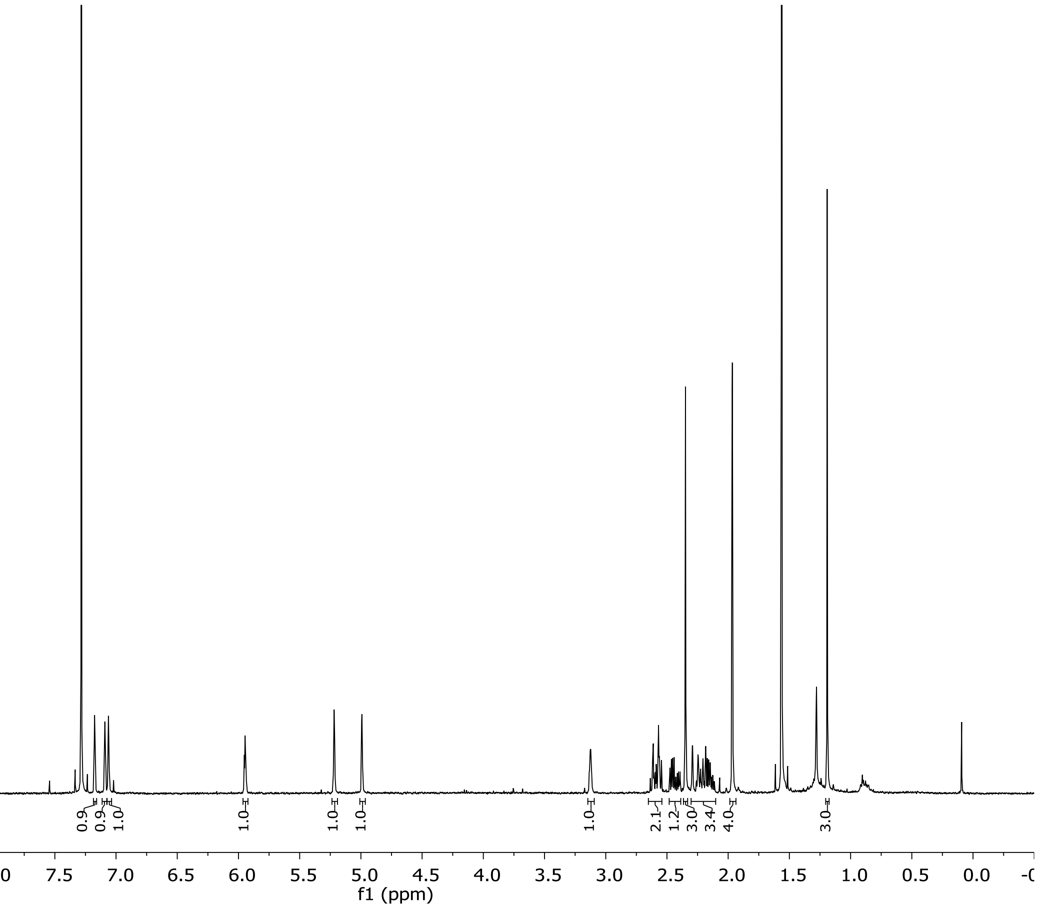
0.9
0.9
1.0
1.0
1.0
1.0
1.0
2.1
1.2
3.0
3.4
4.0
3.0
f1 (ppm)

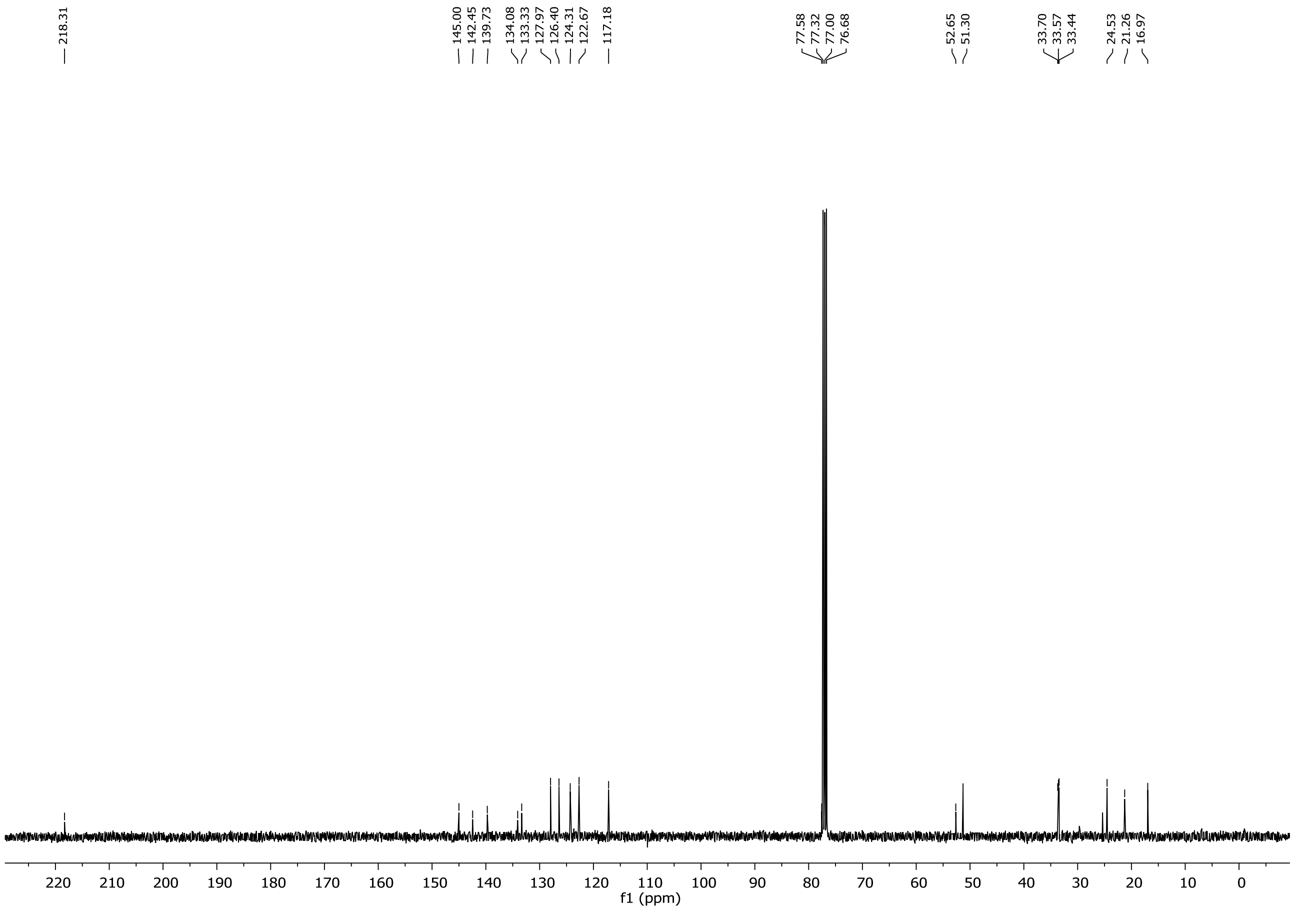
218.31
145.00
142.45
139.73
134.08
133.33
127.97
126.40
124.31
122.67
117.18
77.58
77.32
77.00
76.68
52.65
51.30
33.70
33.57
33.44
24.53
21.26
16.97
f1 (ppm)
